# Synthesis and Anticancer Evaluation of *O*-Alkylated (*E)*-Chalcone Derivatives: A Focus on Estrogen Receptor Inhibition

**DOI:** 10.3390/ijms26020833

**Published:** 2025-01-20

**Authors:** Alwah R. Al-Ghamdi, Wahid U. Ahmed, Reem I. Al-Wabli, Maha S. Al-Mutairi, A. F. M. Motiur Rahman

**Affiliations:** 1 Department of Pharmaceutical Chemistry, College of Pharmacy, King Saud University, Riyadh 11451, Saudi Arabia; 439204477@student.ksu.edu.sa (A.R.A.-G.); ralwabli@ksu.edu.sa (R.I.A.-W.); 2 School of Pharmaceutical Sciences, Zhengzhou University, Zhengzhou 450001, China; ahmeduddin.live@gmail.com

**Keywords:** chalcone derivatives, anticancer agents, cytotoxicity, protein kinase inhibition, anti-estrogenic activity, breast cancer

## Abstract

Cancer remains a leading cause of morbidity and mortality worldwide, highlighting the urgent need for novel therapeutic agents. This study investigated the synthesis and biological evaluation of *O*-alkyl (*E*)-chalcone derivatives (**4a**–**4v**) as potential anticancer agents. The compounds were synthesized via aldol condensation of substituted aldehydes and acetophenones, with structures confirmed by IR, NMR, and mass spectrometry. In vitro cytotoxicity assays revealed varying effectiveness, with compounds **4a**, **4b**, **4q**, and **4v** exhibiting potent activity against MDA-MB-231 and MCF-7, showing IC_50_ values between 2.08 and 13.58 µM, besides HCT-116 and HeLa cancer cell lines (IC_50_ values between 6.59 and 22.64 µM). Notably, compound **4b** displayed remarkable selectivity, with an IC_50_ of 54.59 µM against the non-cancerous WI-38 cell line. Additionally, protein kinase inhibition assays indicated that compounds **4b** and **4q** effectively inhibited EGFR and VEGFR-2, with **4b** outperforming the standard inhibitor erlotinib. Molecular docking studies of compound **4q** showed strong binding affinities in the ATP-binding pockets of EGFR, HER2, VEGFR2, and CDK2. In silico analyses further highlighted the favorable pharmacokinetic properties of compound **4q**, underscoring its potential as a selective tyrosine kinase inhibitor. These findings suggest the therapeutic promise of *O*-alkyl (*E*)-chalcone derivatives in cancer treatment.

## 1. Introduction

**Figure 1:**
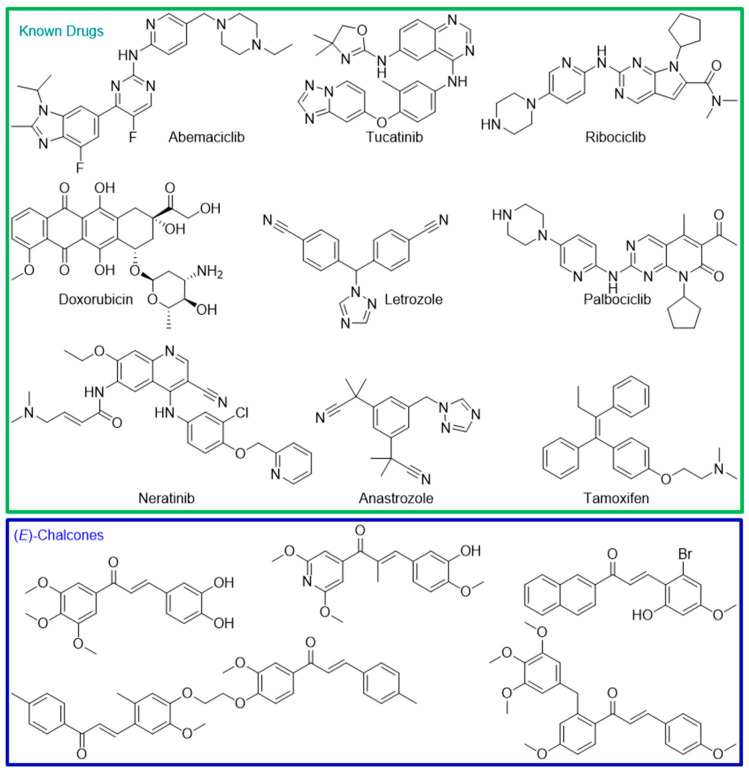
Structures of some anticancer/anti-breast cancer drugs and some reported chalcones with good IC_50_ values.

Breast cancer has emerged as a significant global health challenge and is projected to be one of the leading causes of death in the twenty-first century. According to the World Health Organization (WHO), cancer is the second leading cause of death worldwide, with breast cancer being the most commonly diagnosed cancer among women, accounting for approximately 2.3 million new cases in 2022 [1]. This alarming statistic underscores the need for increased awareness, research, and resources dedicated to combating this formidable disease. The impact of breast cancer extends beyond individual patients, affecting families, communities, and healthcare systems, thereby necessitating a comprehensive approach to its prevention and treatment. The burden of breast cancer is particularly pronounced in Saudi Arabia, where the incidence has dramatically risen in recent years, especially among younger females. In 2022, Saudi Arabia reported 13,400 cancer deaths and 28,000 new cancer cases [2], highlighting the pressing need for effective prevention and treatment strategies tailored to the local context. Alarmingly, breast cancer cases among females in Saudi Arabia have increased by 95%, a surge that is compounded by societal shifts toward a more Westernized lifestyle [3]. These lifestyle changes have led to alterations in reproductive patterns, socioeconomic status, and health behaviors, which can all influence cancer risk. The median age of diagnosis has also risen, suggesting a demographic transition that requires further investigation into the underlying factors driving this trend. Notably, a significant proportion of breast cancer cases among Arab women are diagnosed before the age of 50, in stark contrast to lower rates in Western countries, where earlier detection and screening may contribute to better outcomes. Breast cancer is a complex disease influenced by various risk factors, including age, hormonal factors, and lifestyle choices such as alcohol consumption and obesity [4]. Current treatment options for breast cancer primarily include surgery, chemotherapy, and radiotherapy, but ongoing clinical trials are exploring novel approaches to improve efficacy and reduce side effects [5,6,7]. Metastasis, which is primarily responsible for treatment failure and mortality, is linked to metabolic reprogramming in breast cancer cells [8]. These cells adapt their metabolism to support rapid growth and survival, making it crucial to understand the metabolic mechanisms and the regulatory pathways involved. This understanding can pave the way for developing novel therapeutic strategies specifically aimed at combating metastatic breast cancer, which poses a significant challenge to successful treatment. Breast cancer can be categorized into distinct subtypes based on hormone receptor status, which greatly influences treatment approaches and prognoses [5]. Hormone receptor-positive breast cancers express estrogen receptors (ERs) and/or progesterone receptors (PRs), allowing for targeted therapies that can improve patient outcomes [9,10]. In contrast, triple-negative breast cancers (TNBCs) are characterized by a lack of these receptors and HER2 expression, representing a significant challenge in treatment due to their aggressive clinical behavior and heterogeneous nature [9,10]. Estrogen plays a crucial role in various physiological processes, but its overactivity is a significant factor in the development and progression of hormone-dependent cancers, particularly breast cancer [11,12]. The binding of estrogen to its receptor can stimulate cell proliferation, leading to tumor growth [13]. Therefore, anti-estrogenic agents, such as tamoxifen, anastrozole, and letrozole, are essential in the prevention and treatment of estrogen receptor-positive breast cancers [14] (Figure 1). These drugs work by either blocking estrogen from binding to its receptor or by inhibiting the synthesis of estrogen, effectively reducing its stimulatory effects on cancer cells [14,15]. The development of effective anti-estrogenic drugs is vital for improving therapeutic outcomes and reducing the recurrence of breast cancer, particularly in high-risk populations. Recently developed anti-breast cancer molecules demonstrate promising potential in targeting various breast cancer cell lines. Notable compounds include tucatinib, a HER2-targeted therapy that has shown efficacy in combination with other agents for HER2-positive breast cancer [16,17]. Abemaciclib, a CDK4/6 inhibitor, has also emerged as a crucial treatment option, improving outcomes in hormone receptor-positive cancers by interfering with cancer cell cycle progression [18]. Additionally, neratinib, another HER2 inhibitor, has been effective in extending disease-free survival in certain patient populations [19,20]. The use of CDK4/6 inhibitors such as palbociclib and ribociclib has become increasingly common for advanced hormone receptor-positive breast cancer treatment, providing additional options for managing this disease [21,22]. Furthermore, chalcones have exhibited significant cytotoxicity against various breast cancer lines [23,24,25,26]. Some chalcones are known to effectively inhibit tubulin polymerization and induce G2/M phase arrest [24], while others promote apoptosis by increasing caspase levels—important mechanisms that can lead to cancer cell death [25]. The diverse activities of chalcones highlight their potential as therapeutic agents and warrant further exploration in the context of breast cancer treatment [27,28,29,30,31,32] (Figure 1). A chalcone, namely, 4-hydroxychacone (it is also a secondary metabolite of *E*-chalcone), shows estrogenic activity at 1 × 10^−4^ Mole [33]. Overall, these findings underscore the potential of both novel targeted therapies and chalcones as valuable candidates for further development in the fight against breast cancer. Given the increasing incidence of breast cancer and the complexities associated with its treatment, there is an urgent need for comprehensive strategies aimed at prevention, early detection, and effective management of this disease. This need is particularly critical in regions like Saudi Arabia, where access to healthcare and cancer screening may vary significantly. Continued research and development of new therapeutic molecules, alongside public health initiatives that promote awareness and education, are essential to address this pressing health challenge and improve outcomes for patients globally. In this study, we designed and synthesized a series of both known (**4a**–**4o**, **4r**–**4s**) and novel (**4p**–**4q**, **4t**–**4v**) *O*-alkylated (*E*)-chalcone derivatives, evaluating their anticancer properties against various cancer cell lines, including HCT-116, MDA-MB-231, Hela, and MCF-7, and a non-cancer cell line, WI-38, as well as their effects on specific enzymes (EGFR, HER2, VEGFR-2, and CDK2) and anti-estrogenic activity. This research aimed to contribute to the development of effective anti-breast cancer therapeutics.

## 2. Results and Discussion

### 2.1. Synthesis of (E)-Chalcone Derivatives ***4a***–***4v***

**Scheme 1:**
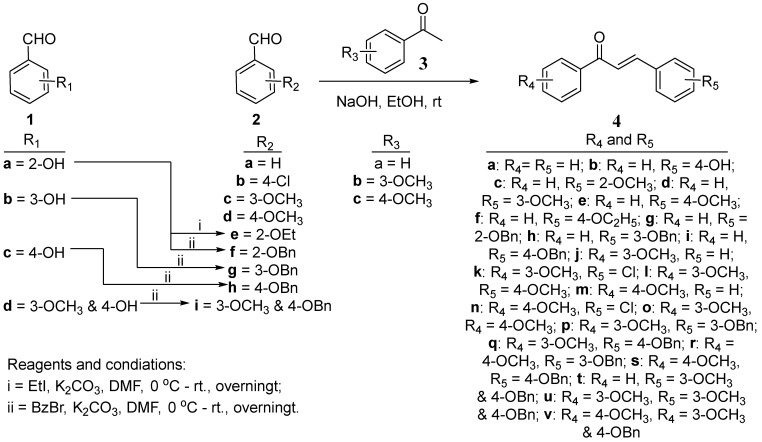
Synthesis of *O*-alkyl (*E*)-chalcones.

*O*-Alkyl (*E)*-chalcones **4a**–**4v** were synthesized using a well-established aldol condensation reaction, which involved reacting substituted aldehydes (**2a**–**2h**) with substituted acetophenones (**3a**–**3c**) in the presence of 10% NaOH in ethanol [34]. Initially, *O*-ethyl aldehydes (**2e**) and *O*-benzyl aldehydes (**2f**–**2h**) were prepared through a Williamson ether synthesis method [35]. This involved the use of K_2_CO_3_ in DMF to react hydroxybenzaldehydes (**1a**–**1d**) with ethyl iodide/benzyl bromide (Scheme 1). The structures of the synthesized chalcones (**4a**–**4v**) were elucidated using IR, NMR, and mass spectrometry. The spectral data were compared to known values where available.

### 2.2. Biological Evaluation of (E)-Chalcone Derivatives ***4a***–***4v***

#### 2.2.1. In Vitro Cytotoxicity Evaluation of **4a**–**4v**

The in vitro cytotoxicity analysis of (*E*)-chalcone derivatives reveals varying degrees of effectiveness, particularly among those classified as low cytotoxicity (Table 1). Compounds such as **4i** and **4k**, which show IC_50_ values greater than 100 µM against all the cell lines (HCT-116, MDA-MB-231, HeLa, and MCF-7, as well as WI-38) tested, contain substituents that likely hinder their therapeutic potential. For instance, **4i** has a bulky 4-benzyloxy group in ring B, which may create steric hindrance, preventing effective interaction with cancer cell targets. Similarly, **4k** features a 3-methoxy group in ring A and a 4-chloro group in ring B, but the combination does not enhance potency, suggesting that the electronic or steric properties of these groups may not favor cytotoxic activity. Other compounds, like **4l**, **4o**, and **4s**, exhibit IC_50_ values against all the cell lines tested, ranging from 61.85 to 91.72 µM, indicating that while they are somewhat more active, they still fall within the low cytotoxicity range. **4l** and **4o** both contain methoxy groups in both rings A and B, yet their overall activity remains limited, possibly due to the specific arrangement of these groups. **4s**, with a 4-methoxy in ring A and a 4-benzyloxy group in ring B, illustrates that the presence of additional bulky substituents may further reduce cytotoxicity. Interestingly, the three compounds (**4l**, **4o**, and **4s**) show strong cytotoxicity against the normal cell line WI-38, with a range between 16.24 and 24.18 µM. Compounds **4t** and **4u**, with IC_50_ values between 55.62 to 82.75 µM, also reflect the trend that certain substituents, like the 3-methoxy, 4-benzyloxy groups in ring B (**4t**), may not provide sufficient electronic or steric advantages to enhance interaction with target cells. Furthermore, **4u**’s combination of multiple methoxy and benzyloxy groups suggests that while these groups can contribute to solubility, they may also interfere with the compound’s overall cytotoxicity. However, the low cytotoxicity observed in these compounds appears to stem from a combination of steric hindrance, unfavorable electronic interactions, and the specific positioning of substituents on the chalcone structure. However, these two compounds also showed toxicity against the WI-38 cell line at lower micromolar concentrations (39.95–40.06 µM).

The moderate cytotoxicity observed in the chalcone derivatives indicates a more favorable interaction with cancer cells compared to the low cytotoxicity compounds. Specifically, derivatives such as **4c**, **4d**, **4f**, **4g**, **4h**, **4m**, **4n**, **4p**, and **4r** show IC_50_ values ranging from 23.51 to 74.51 µM, suggesting that these compounds have enhanced biological activity. For instance, **4c** and **4d**, with IC_50_ values of 23.51 to 42.46 µM and 29.67 to 43.59 µM, respectively, contain methoxy groups in ring B. Both **4c** and **4d** showed better IC_50_ values against the MCF-7 and HCT-116 cell lines compared with the MDA-MB-231 and HeLa cell lines. Importantly, against WI-38, they showed higher IC_50_ values (77.26–100 µM). This configuration indicates that the presence of methoxy groups in ring B can positively influence cytotoxicity, likely due to their electron-donating properties, which may enhance the compounds’ reactivity toward biological targets. **4f**, featuring a 4-ethoxy group in ring B, demonstrates similar moderate activity (35.28 to 57.59 µM). The ethoxy group may provide a balance between steric effects and electronic properties that support effective binding to target sites. Compounds **4g** and **4h**, with 2-benzyloxy and 3-benzyloxy groups, respectively, in ring B, also show moderate potency (35.28 to 53.80 µM and 25.63 to 53.80 µM). Here, the positioning of the benzyloxy group appears to be crucial; the 2-position in ring B may offer better spatial orientation for interaction with the target compared with bulkier substituents at the 4-position in ring B observed in lower potency compounds. Among compounds **4f**, **4g**, and **4h**, compound **4h** shows better cytotoxicity (25.63 µM) against HeLa. However, against the normal cell line WI-38, the IC_50_ value is much higher (83.39 µM). **4m** and **4n**, with 4-methoxy in ring A, exhibit IC_50_ values of 33.04 to 63.65 µM and 36.97 to 74.51 µM. The presence of the methoxy group in ring A suggests that this position can also enhance cytotoxicity, while the additional 4-chloro group in ring B (**4n**) may introduce electronic effects that further influence activity, albeit with a slight decrease in potency compared with **4m**. Finally, compounds **4p** and **4r**, which contain combinations of methoxy and benzyloxy groups in both rings A and B, yield IC_50_ values ranging from 39.34 to 68.42 µM. The best IC_50_ value was obtained for compound **4p** at 39.84 µM against HCT-116. On the other hand, compound **4r** showed an IC_50_ value of 39.34 µM against MCF-7 cell lines. This indicates that while these substituents enhance cytotoxicity, their specific positions and interactions remain critical for maximizing their effectiveness. However, the moderate cytotoxicity of these chalcone derivatives is largely attributed to the beneficial effects of methoxy and benzyloxy groups, which improve their reactivity and binding to cancer cell targets. The careful positioning of these substituents is essential for optimizing their biological activity, suggesting potential pathways for further structural modifications to enhance potency.

The chalcone derivatives exhibiting good to excellent cytotoxicity, namely **4a**, **4b**, **4e**, **4j**, **4q**, and **4v**, demonstrate IC_50_ values ranging from 2.08 to 34.91 µM, indicating their strong potential against cancer cell lines. **4a**, with IC_50_ values of 5.16 to 15.38 µM, stands out as a highly effective compound despite having no substitutions in either ring. Excellent IC_50_ values were shown against MCF-7 (5.16 µM) and MDA-MB-231 (7.06 µM) cell lines. This suggests that the core chalcone structure itself possesses significant inherent activity, potentially due to optimal geometric and electronic properties that facilitate interaction with cellular targets. **4b**, containing a 4-hydroxy group in ring B, shows even greater potency, with IC_50_ values of 2.08 to 13.07 µM. In the case of **4b**, the IC_50_ values are the best among all the compounds in this series against the MCF-7 cell line (2.08 µM), as well as against the MDA-MB-231 (4.93 µM) and HCT-116 (6.59 µM) cell lines. The presence of the hydroxy group likely enhances the compound’s reactivity through hydrogen bonding or improved solubility, facilitating stronger interactions with biological targets and contributing to its excellent cytotoxicity. **4e** and **4j** exhibit IC_50_ values of 16.90 to 29.52 µM and 18.83 to 34.91 µM, respectively; both feature methoxy groups in ring B and ring A, respectively. The methoxy substitution in ring B of **4e** and in ring A of **4j** indicates that these groups can enhance activity through electron-donating effects, which may improve the compounds’ reactivity and binding affinity in cancer cells. Overall, these two compounds demonstrate similar cytotoxicity against all the cell lines tested. **4q** and **4v** illustrate how a combination of functional groups can further optimize cytotoxicity. **4q**, with a 3-methoxy in ring A and a 4-benzyloxy group in ring B, shows IC_50_ values of 11.56 to 17.06 µM. The presence of both methoxy and benzyloxy groups suggests synergistic effects that enhance its biological activity. Similarly, **4v**, which has a 4-methoxy group in ring A and both 3-methoxy and 4-benzyloxy groups in ring B, demonstrates IC_50_ values of 8.67 to 18.16 µM, indicating that the structural diversity and positioning of these substituents contribute positively to its cytotoxic efficacy. Compound **4q** shows better IC_50_ values against MCF-7 (11.56 µM), while compound **4v** is more effective against both MCF-7 (8.67 µM) and MDA-MB-231 (10.24 µM) cell lines. The excellent cytotoxicity of these compounds is attributed to the balance of structural simplicity in **4a**, the beneficial functional groups in **4b**, and the strategic positioning of methoxy and benzyloxy groups in the other derivatives. This highlights the importance of both the core chalcone structure and the presence of specific substituents in maximizing cytotoxic activity against cancer cells.

The analysis of the chalcone derivatives reveals that compounds **4a**, **4b**, **4q**, and **4v** exhibit excellent cytotoxicity against all the cancer cell lines tested, with IC_50_ values ranging from 2.08 to 22.64 µM. This potent activity suggests that these compounds effectively target and inhibit cancer cell proliferation, making them promising candidates for further development in cancer therapy. Selectivity is a critical factor in evaluating the therapeutic potential of these compounds. Among the highly active derivatives, **4b** shows an IC_50_ value of 54.59 µM against the non-cancer cell line WI-38, while **4q** (>100 µM) and **4v** (75.17 µM) demonstrate even greater selectivity. This indicates that these compounds can effectively distinguish between cancerous and non-cancerous cells, potentially minimizing side effects and enhancing safety in clinical applications. In contrast, the standard chemotherapeutic agents doxorubicin and sorafenib exhibit IC_50_ values between 3.18 and 8.04 µM against the same cancer cell lines but show no selectivity against WI-38. This lack of selectivity raises concerns about their potential toxicity to healthy cells, highlighting the advantage of the chalcone derivatives, which not only possess potent anticancer activity but also exhibit a more favorable selectivity profile. However, the findings suggest that chalcone derivatives **4a**, **4b**, **4q**, and **4v** represent a promising avenue for developing new anticancer agents with enhanced selectivity, thereby reducing the risk of adverse effects associated with traditional chemotherapy. Further studies are warranted to elucidate the mechanisms of action of these compounds and to optimize their structures for even greater efficacy and selectivity in cancer treatment.

The selectivity index (SI) values presented in Table 1 provide valuable insights into the selectivity of the synthesized compounds (**4a**–**4v**) against the various cancer cell lines tested. The SI values represent the ratio between the IC_50_ of the normal WI-38 cell line and the respective cancer cell lines. Higher SI values indicate greater selectivity of the compounds for the cancer cells over normal cells [36]. The data reveal that several of the synthesized compounds exhibited exceptional selectivity profiles. Compounds **4a**, **4b**, **4e**, **4q**, and **4v** demonstrated the highest SI values against the cancer cell lines tested. Specifically, these compounds showed SI values ranging from 4.8 to 8.3 for HCT-116, 4.1 to 11.1 for MDA-MB-231, 4.1 to 4.4 for HeLa, and 4.2 to 26.2 for MCF-7 cells. In contrast, the standard chemotherapeutic agents’ doxorubicin and sorafenib had significantly lower SI values, ranging from 1.2 to 2.1 and 1.3 to 1.9, respectively. This suggests that the synthesized compounds **4a**, **4b**, **4e**, **4q**, and **4v** possess a superior ability to selectively target the cancer cells while sparing the normal WI-38 cells compared with the standard drugs. The exceptional selectivity profiles exhibited by these compounds highlight their potential as targeted anticancer agents, as they may be able to effectively eliminate cancer cells while minimizing the adverse effects on healthy tissues. These findings warrant further investigation into the underlying mechanisms and potential clinical applications of these promising compounds.

#### 2.2.2. In Vitro Protein Kinase Inhibition Assays of **4a**, **4b**, **4q** and **4v**

The in vitro protein kinase inhibition IC_50_ values for chalcone derivatives **4a**, **4b**, **4q**, and **4v** were evaluated against key targets, EGFR, HER2, VEGFR-2, and CDK2, and compared to the standard control inhibitors erlotinib, sorafenib, and dinaciclib (Table 2). Compound **4b** exhibited exceptional potency, with 54% inhibition at the concentration of 0.1 µM (IC_50_ ≈ 0.066 ± 0.002 µM) for EGFR and IC_50_ at 0.163 µM for VEGFR-2, outperforming all tested chalcone derivatives and demonstrating superior activity compared with erlotinib, which has an IC_50_ of 0.056 ± 0.002 µM for EGFR. Compound **4q** also showed strong inhibition across multiple targets, with IC_50_ values of 0.151 µM for EGFR, 48% inhibition at the concentration of 0.1 µM (IC_50_ ≈ 0.144 ± 0.006 µM) for HER2, IC_50_ at 0.287 µM for VEGFR-2, and 41.9% inhibition at the concentration of 0.1 µM (IC_50_ ≈ 0.17 ± 0.005 µM) for CDK2. This profile indicates effective broad-spectrum activity, particularly against HER2, where it closely approaches the potency of **4b**. In contrast, **4a** displayed moderate inhibition across all targets, while **4v** had notably higher IC_50_ values, suggesting reduced potency. Sorafenib was effective against VEGFR-2, with 63.9% inhibition at the concentration of 0.1 µM (IC_50_ ≈ 0.058 ± 0.002 µM), but it lacked activity against EGFR and HER2, unlike the chalcone derivatives. Dinaciclib exhibited high potency against CDK2, with 60% inhibition at the concentration of 0.1 µM (IC_50_ ≈ 0.044 ± 0.001 µM). The correlation coefficients presented indicate an average strength of relationship between the variables, suggesting that while the IC_50_ values (Table 2) demonstrate a clear inhibitory effect (as illustrated in Figures S1–S21), they should be interpreted as approximate rather than definitive. Nevertheless, these values serve as valuable guidelines for data interpretation. This further highlights the need for optimization of the chalcone compounds to enhance their efficacy compared with these established therapeutic agents.

#### 2.2.3. In Vitro Anti-Estrogenic Activity Assays of **4a**, **4b**, **4q**, and **4v**

**Figure 2:**
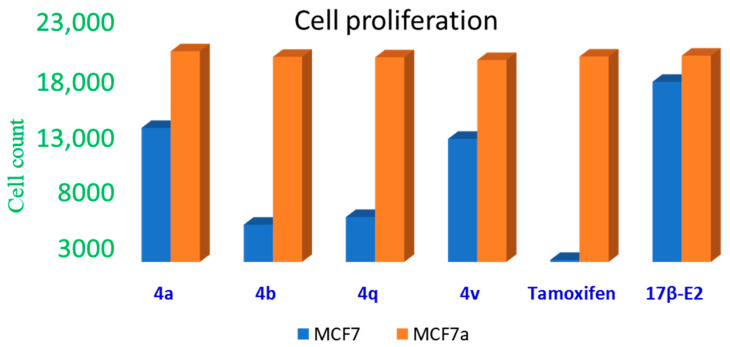
Cell count chart of in vitro anti-estrogenic activity **4a**, **4b**, **4q**, and **4v** on MCF-7 and MCF-7a cell lines.

Table 3 and Figure 2 present data on the anti-estrogenic activity of chalcone derivatives **4a**, **4b**, **4q**, and **4v** against two breast cancer cell lines. MCF-7 is a well-established breast cancer cell line known for its estrogen receptor (ER) positivity. It is commonly used in research to study hormone-responsive breast cancer, particularly how estrogen influences cancer cell growth and behavior. MCF-7 cells generally require estrogen for optimal growth and proliferation, making them a valuable model for examining estrogen signaling pathways and potential therapeutic interventions. On the other hand, MCF-7a, a variant of the MCF-7 cell line, is also estrogen-dependent, but it may exhibit distinct biological behaviors compared to the original MCF-7. The specific adaptations in MCF-7a can include differences in growth rates, drug sensitivity, or responses to hormonal stimuli. MCF-7 and MCF-7a were measured by cell counts and the percentage of cell count relative to the control. Compound **4a** exhibited moderate anti-estrogenic activity in MCF-7 cells, reducing growth by 21%, but no inhibition in MCF-7a. In contrast, **4b** showed significant inhibition in MCF-7 cells (66% reduction in counts) while achieving nearly complete proliferation (99%) in MCF-7a, indicating strong selective activity. Similarly, compound **4q** demonstrated effective anti-estrogenic properties, with a 63% reduction in MCF-7 and only 1% inhibition in MCF-7a. Compound **4v** also exhibited moderate activity, reducing MCF-7 growth by 27% and achieving 2% inhibition in MCF-7a. For comparison, tamoxifen showed effective anti-estrogenic activity in MCF-7 (83% inhibition) but no effect on the growth of MCF-7a, while 17β-E2 serves as a control that promotes growth in both cell lines. Overall, the chalcone derivatives exhibit varying degrees of anti-estrogenic activity, particularly **4b** and **4q**, suggesting their potential as therapeutic agents in breast cancer treatment, warranting further investigation into their mechanisms of action.

#### 2.2.4. In Vitro Aromatase Inhibition Assays of **4a**, **4b**, **4q**, and **4v**

The in vitro aromatase inhibition IC_50_ values for chalcone derivatives **4a**, **4b**, **4q**, and **4v** were evaluated against aromatase (CYP19A, EC 1.14.14.14), a member of the cytochrome P450 monooxidase (CYP) family of microsomal xenobiotic metabolism enzymes, and compared to standard control letrozole (Table 4). Compounds **4a** and **4v** exhibited good potency, with IC_50_ values of 0.434 and 0.357 µM, respectively. Compound **4q** also showed good inhibition, with IC_50_ values of 0.901 µM, and compound **4b** displayed moderate inhibition, with 1.634 µM. Letrozole showed good inhibition (48%) at the concentration of 0.1 µM (IC_50_ ≈ 0.102 ± 0.003 µM). As mentioned in Section 2.2.2, the IC_50_ values for anti-aromatase activity were calculated from non-linear curves and should be considered approximate rather than definitive.

#### 2.2.5. In Silico Studies of the Synthesized Compounds **4a**–**4v**

##### Analysis of Binding Mechanism of Compound **4q** and Reference Compounds by Molecular Docking Study

To compare the docking results to the in vitro biological activity data of compound **4q** and establish a possible correlation between the predicted binding interactions and the observed biological responses, a comprehensive docking study was conducted. This approach was undertaken to guide the design and optimization of more potent and selective compounds. Specifically, compound **4q** was meticulously docked into the active sites of EGFR, HER2, VEGFR2, and CDK2. To establish a reference point, the co-crystal ligands erlotinib (against EGFR and Her-2), sorafenib (VEGFR-2), and dinaciclib (CDK2) were utilized as standards for each respective target (Table 5 and Figure S22).

As shown in Table 5, the binding affinities for compound **4q** were calculated to be −10.0, −10.5, −10.1, and −8.5 kcal/mol against erlotinib (against EGFR and HER-2), VEFGR-2, and CDK2, respectively. It is our surprise that erlotinib’s binding affinities with EGFR (−7.3 kcal/mol) and HER-2 (−8.3 kcal/mol) are much less than compounds **4q**. On the other hand, sorafenib and dinaciclib show little more binding affinities than the compound **4q** against VEGFR-2 (−10.7 kcal/mol) and CDK2 (−9.1 kcal/mol). Therefore, synthesized **4q** can effectively bind to the ATP binding pocket and inhibit the activity of EGFR, Her2, VEGFR2, and CDK2 kinase.

**Figure 3:**
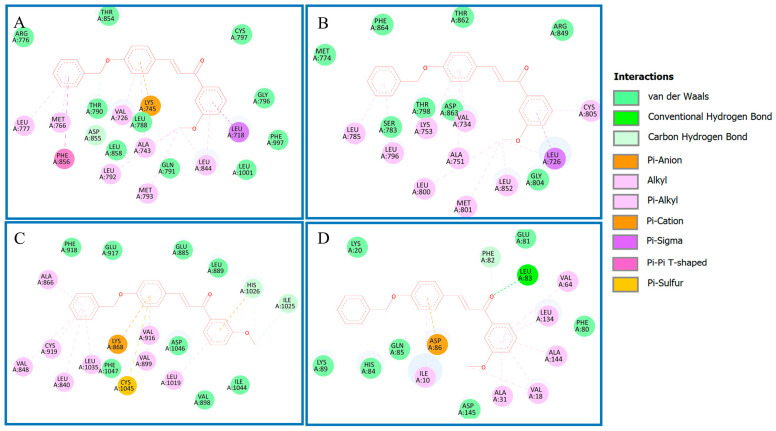
Intermolecular interaction between compound **4q** and enzymes with (**A**) EGFR; (**B**) HER2; (**C**) VEGFR2; and (**D**) CDK2.

The docking study of compound **4q** with EGFR revealed that it binds within the ATP binding pocket of the catalytic tyrosine kinase domain, thereby competing with ATP for binding (Figure 3A). The docking study of compound **4q** with EGFR kinase showed several van der Waals interactions with Arg776, Thr854, Cys797, Thr790, Leu858, Leu788, Gln791, Leu1001, Phe997, and Gly769 in the active site. Our reference standard, erlotinib, exhibited hydrogen bonds with Asp855, Lys745, and Met793 while also revealing some carbon–hydrogen bonds with Gln791, Asn842, and Asp855 (Figures S23 and S24). It also demonstrated van der Waals interactions within the active site. Both **4q** and erlotinib showed similar patterns of hydrophobic interactions with Leu718 and Leu844. However, it is important to note that compound **4q** exhibited hydrophobic and carbon–hydrogen interactions with Asp855 and Met793, respectively, whereas erlotinib formed conventional hydrogen bonds with these residues. A study found that carbon atoms have the potential to establish hydrogen bonds that can be as strong as those generated by conventional donors like oxygen or nitrogen if they experience more polarization from nearby atoms [37,38]. Human epidermal growth factor receptor-2 (HER2) is a membrane tyrosine kinase that can significantly affect cell survival and proliferation when overexpressed [39,40]. Essential amino acids in HER2’s ATP binding pocket include LYS753, VAL734, ALA751, GLN799, MET801, LEU852, LEU726, PHE1004, ASP863, ASN850, GLU770, MET774, LEU785, and LEU796 [41]. Compound **4q** did not form any hydrogen bonds with HER2 (Figure 3B). However, it displayed hydrophobic interactions with LEU785, LEU796, LYS753, VAL734, ALA751, MET801, LEU852, and LEU726, as well as van der Waals interactions with ASP863, which are important residues in the ATP binding site. The reference compound, erlotinib, also did not form any conventional hydrogen bonds. Erlotinib exhibited similar hydrophobic and van der Waals interactions with the ATP binding pocket. Hydrophobic and van der Waals interactions can play a significant role in stabilizing ligands at the binding pocket [42]. Thus, it can be concluded that **4q** has the ability to successfully inhibit HER2 by competing with ATP for the binding pocket. VEGF receptor 2 (VEGFR2, also known as KDR) plays a significant role in regulating tumor angiogenesis through the vascular endothelial growth factor (VEGF) signaling pathway [43,44]. Regarding VEGFR2, compound **4q** did not demonstrate any hydrogen bonds (Figure 3C). In contrast, sorafenib formed hydrogen bonds with CYS919, GLU885, and ASP1046 (Figure S23). Both compound **4q** and sorafenib interacted through similar hydrophobic interactions with Ala866, Leu1035, Leu840, Val848, Val916, Leu1019, and Phe918. Additionally, compound **4q** exhibited carbon–hydrogen interactions with His1026 and Ile1025. Van der Waals interactions are provided in the Supplementary Tables S1–S6. Since compound **4q** and the reference standard displayed identical interactions with the amino acid residues, it can be concluded that compound **4q** has the ability to bind to the active site of VEGFR2 kinase. The most significant function of cyclin-dependent kinase 2 (CDK2) is to regulate the cell cycle. This member of the cyclin-dependent kinase (CDK) family regulates G2 progression, the G1/S phase transition, and DNA synthesis [45]. From the recently published crystal structure of CDK2 in complex with the inhibitor CVT-313 (PDB ID: 6INL), it was visualized that vital residues involved in binding to the ATP-binding site through hydrogen bonds include LEU83, ASP86, and ASP145. Notably, ASP145 is part of the DFG motif, which consists of the residues Asp145-Phe146-Gly147. This DFG motif is essential for the kinase’s active conformation and is crucial for effective inhibition of the kinase’s activity. In CDK2, the N-terminal domain contains the distinct PSTAIRE motif (Pro45–Glu51), which is important for interacting with the cyclin subunit [46]. From the docking analysis of compound **4q** with CDK2, it was observed that the oxygen atoms of the carbonyl group formed a hydrogen bond with the LEU83 residue but did not form any hydrogen bond with ASP145 of the DFG motif (Figure 3D). The reference compound, dinaciclib, formed two hydrogen bonds with Leu83 and Lys89. Unfortunately, the synthesized compound **4q**, as well as the reference compound dinaciclib, did not form any hydrogen bond with ASP145 of the DFG motif. However, they did form hydrogen bonds with other key residues. Therefore, compound **4q** can interact with the ATP binding pocket residues of the CDK2 domain and act as an ATP competitive inhibitor.

**Figure 4:**
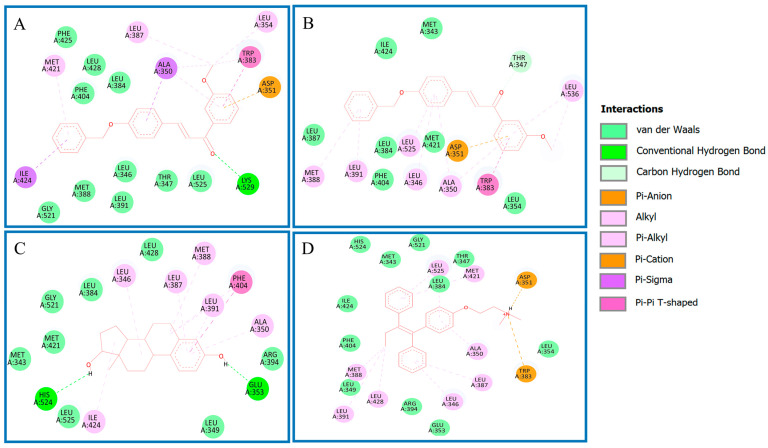
Intermolecular interaction between compound **4q** and estrogen receptors with (**A**) **4q**-estrogen receptors (PDB ID: 1A52), (**B**) **4q**-estrogen receptors (PDB ID: 3ERT), (**C**) estrogen-estrogen receptors (PDB ID: 1A52), and (**D**) tamoxifen-estrogen receptors (PDB ID: 3ERT).

The synthesized compound **4q** demonstrated anti-estrogenic effects in the in vitro activity analysis. Estrogen receptors (ERs) are generally activated by estrogen; however, estrogen signaling is crucial in the development of several malignancies, including ovarian [47], endometrial [48], and breast cancers [49]. Therefore, molecular docking analysis of **4q** was performed with the active site of the estrogen receptor (PDB ID: 1A52), where estradiol binds and activates the signaling pathway (Figure 4A). Estradiol, the most potent estrogen, was also docked to visualize the binding affinity and interactions (Figure 4C). The results revealed that both estradiol and compound **4q** can bind to the active site of the estrogen receptor. However, compound **4q** showed weaker binding affinity than estradiol, which was −7.6 kcal/mol for **4q** and −10.6 kcal/mol for estradiol (Figure S25). Binding interaction analysis demonstrated that both estradiol and **4q** were stabilized by different types of interactions. Compound **4q** established one hydrogen bond with LYS529, while estradiol formed two hydrogen bonds with GLU353 and His524 residues. Both compound **4q** and estradiol showed hydrophobic interactions with Ile424, Leu387, and Ala350, as well as van der Waals interactions with Leu428, Leu384, Gly521, and Leu525, as illustrated in Figure 3. Moreover, to further visualize the inhibitory activity and binding pattern of **4q** in the active site of estrogen receptors (PDB ID: 3ERT), tamoxifen, a selective estrogen receptor modulator and FDA-approved antiestrogen drug, was selected as a reference compound according to our experimental data. Docking analysis showed that both compound **4q** (Figure 4B) and tamoxifen (Figure 4D) did not form any hydrogen bonds in the active site but were stabilized by different types of hydrophobic and van der Waals interactions. Both compounds displayed similar hydrophobic interactions with Met388, Leu391, Leu525, Leu346, and Ala350 residues, as well as pi-anion interactions with ASP351 and van der Waals interactions with Leu354, Leu384, Ile424, Phe404, Met343, and Leu349. Additionally, compound **4q** exhibited carbon–hydrogen interactions with Thr347. The binding affinities for tamoxifen and **4q** with the human estrogen receptor were −9.8 and −9.2 Kcal/mol, respectively, which is consistent with our in vitro analysis. So, compound **4q** has the ability to demonstrate the anti-estrogenic effect. Thus, compound **4q** has the potential to demonstrate an anti-estrogenic effect.

##### In Silico Drug Likeness Property Analysis of **4a**–**4v**

Rational drug design is the most significant part of modern drug discovery methods. We may choose the most effective drug in terms of cost, time, and efficiency by using the advanced computational ADME (absorption, distribution, metabolism, and excretion) and toxicity study. Thanks to recent advancements in computational chemistry, ADME analysis has become more efficient for both in vitro and in vivo studies. This progress enables the pharmaceutical industry to screen many compounds rapidly, streamlining the drug development process [50]. The pkCSM protocol, which utilizes graph-based signatures, was employed to assess the synthesized compounds’ predicted toxicity and pharmacokinetic properties.

We predicted some ADME and toxicity properties using the in silico method in this experiment (Table 6). Here, synthesized compounds (**4a**–**4v**) were screened to predict the ADME and toxicity properties, and the results are summarized in Table 6. Doxorubicin and sorafenib were assigned as our reference standard. The main factor of intestinal absorption is molecular weight. There could be a connection between the high molecular weight and the effectiveness of intestinal absorption. Given that high molecular weight compounds are often less effectively absorbed through the digestive system [51,52]. The molecular weight of our novel compounds (**4a**–**4v**) was kept low. Our reference standard doxorubicin had a relatively higher molecular weight. As a result, it showed a low capability of intestinal absorption. All of our synthesized compounds, as well as sorafenib, displayed a good intestinal absorption rate. Moreover, our compound demonstrated hydrogen bond donor between (0–1), which is within the recommended value (recommended value ≤ 5). However, in the case of doxorubicin, the HBD value was 6, which is higher than the recommended value. Subsequently, it was discovered that the compounds under investigation included less than 10 hydrogen bond acceptors, falling within the recommended range. However, doxorubicin displayed an HBA value of 12, a bit higher than the recommended range. This indicates that our novel compounds are superior to doxorubicin in terms of HBA and HBD values. Jorgensen and Duffy developed a parameter in 2002 to evaluate a drug’s bioavailability using the solubility score. (The Recommended values for solubility scoring are −6.5–0.5 mol/L) [53]. The solubility score of our novel compounds and the reference falls within the recommended value. Additionally, our predictions indicate that most of the derivatives are unlikely to be substrates for the major biotransformation enzymes CYP2D6 and CYP3A4, with the exception of compounds **4g**–**4i**, **4p**–**4v**, sorafenib, and doxorubicin, which may be metabolized by CYP3A4. The estimated toxicity profiles of the synthesized compounds suggest a high maximum tolerated dose and low risk of both acute and chronic oral toxicity. Notably, none of the novel compounds exhibited hepatotoxicity except our reference standard sorafenib, though some were predicted to cause cutaneous sensitivity. These comprehensive in silico analyses provide valuable insights into the pharmacological properties and safety profiles of the synthesized compounds, supporting their potential for further development as tyrosine kinase inhibitors.

## 3. Materials and Methods

### 3.1. General

The experiments were conducted using commercially available reagents and solvents without any additional purification. Melting points were measured using a Barnstead electrothermal digital melting point apparatus (model IA9100, BIBBY scientific limited, Staffordshire, UK). IR spectra were recorded using a Jasco FT/IR-6600 spectrometer (Tokyo, Japan). NMR spectra were obtained using a Bruker 700 MHz NMR spectrometry instrument (Zurich, Switzerland). ^1^H-NMR, ^13^C-NMR, and 2D spectra were determined on Bruker (700 MHz). Chemical shifts were expressed as δ values (ppm) using tetramethyl silane (TMS) as an internal reference. Signals were indicated with the following abbreviations: s = singlet, d = doublet, t = triplet, q = quartet, m = multiple, br = broad, dd = doublet of doublet, ddd = doublet of doublet of doublet, td = triplet of doublet, dt = doublet of triplet, and qd = quartet of doublet. Electrospray ionization (ESI) mass spectrometry (MS) experiments were carried out using an Agilent 6320 ion trap mass spectrometer equipped with an ESI ion source (Agilent Technologies, Palo Alto, CA, USA). The reagents were RPMI-1640 medium, MTT and DMSO (Sigma Co., St. Louis, MA, USA), and Fetal Bo-vine serum (GIBCO, London, UK). Colorectal carcinoma, colon cancer (HCT-116; association number, CCL-247^™^), epithelioid carcinoma cervix cancer (Hela; association number, CCL-2^™^), mammary gland breast cancer (MCF-7; association number, HTB-22) (MDA-MB-231; association number, HTB-26™), and human lung fibroblast (WI-38; association number, CCL-75) cell lines were obtained from ATCC through a holding company for biological products and vaccines (VACSERA), Cairo, Egypt.

### 3.2. General Synthetic Method for the Synthesis of ***2e***–***2i***

General Method. Hydroxybenzaldehyde (0.1 mol) was charged with anhydrous K_2_CO_3_ (0.3 mol) in dry dimethylformamide (DMF, 100 mL) and stirred at 0 °C for 30 min. A total of 1.2 equivalents of ethyl iodide (EtI)/benzyl bromide (BnBr) (0.12 mol) were added to the mixture, which was then stirred at room temperature overnight. The entire reaction mixture was poured into ice-cold water, and the resulting solid precipitate or oil was collected, dried, and used for the next step without further purification. **2e** is characterized as oil (CAS RN: 613-69-4), **2f** as oil (CAS RN: 5896-17-3), **2g** as solid at mp 58 °C (CAS RN: 1700-37-4), **2h** as solid at mp 71–74 °C (CAS RN: 4397-53-9), and **2i** as solid at mp 63 °C (CAS RN: 2426-87-1).

### 3.3. General Synthetic Method for the Synthesis of ***4a***–***4v***

General Method. To a mixture of benzaldehyde (**2a**, 0.01 mol) and acetophenone (**3a**, 0.01 mol) in ethanol (20 mL), 10% NaOH (1 mL) was added, and the mixture was stirred at room temperature overnight (12 h). The formation of solid precipitation was filtered off and washed with cold ethanol. Analytical pure **4a** was obtained.

(*E*)-Chalcone (**4a**). White powder (83%). Mp. 59 °C (lit. [54,55] mp. 56 °C). FT-IR (KBr): ν (cm^−1^) = 3446, 3058, 1661, 1604, 1574, 1496, 1339, 1286, 1035, 1015, 995, 976, 862 and 688 cm^−1^. ^1^H-NMR (700 MHz, CDCl_3_) δ 8.01 (d, *J* = 7.1 Hz, 2H), 7.80 (d, *J* = 15.7 Hz, 1H), 7.63 (dd, *J* = 6.4, 3.2 Hz, 2H), 7.57 (t, *J* = 7.4 Hz, 1H), 7.52 (d, *J* = 15.7 Hz, 1H), 7.49 (t, *J* = 7.6 Hz, 2H) and 7.41 (overlapped, 3H) ppm. ^13^C-NMR (176 MHz, CDCl_3_) δ 190.57, 144.85, 138.20, 134.88, 132.78, 130.54, 128.96, 128.62, 128.50, 128.44 and 122.09 ppm. Mass (ESI): *m*/*z* 209 [M + H]^+^.

(*E*)-3-(4-Hydroxyphenyl)-1-phenylprop-2-en-1-one (**4b**). Brown crystal (73%). Mp. 178 °C (lit. [56] mp. 186–187 °C). FT-IR (KBr): ν (cm^−1^) = 3225, 3019, 2810, 2362, 1895, 1649, 1598, 1557, 1510, 1349, 1283, 1107, 834 and 690 cm^−1^. ^1^H-NMR (700 MHz, DMSO-*d*_6_) δ 10.08 (s, 1H, -OH), 8.10 (d, *J* = 8.4 Hz, 2H), 7.73 (d, *J* = 8.8 Hz, 2H), 7.71 (d, *J* = 15.5 Hz, 1H), 7.67 (d, *J* = 15.5 Hz, 1H), 7.64 (t, *J* = 7.4 Hz, 1H), 7.55 (t, *J* = 7.7 Hz, 2H) and 6.83 (d, *J* = 8.6 Hz, 2H) ppm. ^13^C-NMR (176 MHz, DMSO-*d*_6_) δ 189.39, 160.55, 144.92, 138.34, 133.22, 131.44, 129.12, 128.73, 126.16, 118.87 and 116.21 ppm. Mass (ESI): *m*/*z* 225 [M]^+^; 247 [M + Na]^+^.

(*E*)-3-(2-Methoxyphenyl)-1-phenylprop-2-en-1-one (**4c**). Off-white powder (77%). Mp. 53 °C (lit. mp 53–54 °C [56]; mp 59–61 °C [55]). FT-IR (KBr): ν (cm^−1^) = 3312, 3063, 2965, 2835, 2620, 1683, 1661, 1449, 1313, 1169, 1017, 978, 847and 684 cm^−1^. ^1^H-NMR (700 MHz, CDCl_3_) δ 8.00 (d, *J* = 7.5 Hz, 2H), 7.76 (d, *J* = 15.8 Hz, 1H), 7.57 (t, *J* = 7.3 Hz, 1H), 7.50 (d, *J* = 8.0 Hz, 2H), 7.49 (d, *J* = 15.8 Hz, 1H), 7.32 (t, *J* = 8.0 Hz, 1H), 7.23 (d, *J* = 8.0 Hz, 1H), 7.14 (t, *J* = 2.3 Hz, 1H), 6.95 (d, *J* = 5.4 Hz, 1H) and 3.84 (s, 3H) ppm. ^13^C-NMR (176 MHz, CDCl_3_) δ 190.57, 159.94, 144.77, 138.17, 136.25, 132.79, 129.94, 128.62, 128.50, 122.39, 121.09, 116.30, 113.41 and 55.34 ppm. Mass (ESI): *m/z* 239 [M + H]^+^; 261 [M + Na]^+^.

(*E*)-3-(3-Methoxyphenyl)-1-phenylprop-2-en-1-one (**4d**). Yellow powder (76%). Mp. 62 °C (lit. [55] mp. 58–59 °C). FT-IR (KBr): ν (cm^−1^) = 3060, 3013, 2956, 2836, 1683, 1661, 1575, 1488, 1339, 1248, 1016, 929, 866 and 585 cm^−1^. ^1^H-NMR (700 MHz, CDCl_3_) δ 8.10 (d, *J* = 15.9 Hz, 1H), 8.00 (d, *J* = 7.4 Hz, 2H), 7.62 (d, *J* = 7.1 Hz, 1H), (d, *J* = 15.9 Hz, 1H), 7.56 (t, *J* = 7.5 Hz, 1H), 7.48 (t, *J* = 7.2 Hz, 2H), 7.36 (t, *J* = 7.8 Hz, 1H), 6.98 (t, *J* = 7.4 Hz, 1H), 6.93 (d, *J* = 8.2 Hz, 1H) and 3.90 (s, 3H) ppm. ^13^C-NMR (176 MHz, CDCl_3_) δ 190.98, 158.63, 140.23, 138.35, 132.34, 131.56, 129.06, 128.34, 123.75, 122.71, 120.55, 111.04 and 55.35 ppm. Mass (ESI): *m*/*z* 239 [M + H]^+^; 261 [M + Na]^+^.

(*E*)-3-(4-Methoxyphenyl)-1-phenylprop-2-en-1-one (**4e**). Yellow crystal (86%). Mp. 92 °C (lit. mp. 91 °C [54]; mp. 79–80 °C [55]). FT-IR (KBr): ν (cm^−1^) = 3224, 3019, 2810, 2362, 1649, 1579, 1447, 1349, 1283, 1167, 1021, 941, 834 and 690 cm^−1^. ^1^H-NMR (700 MHz, CDCl_3_) δ 8.04 (d, *J* = 7.5 Hz, 2H), 7.82 (d, *J* = 15.1 Hz, 1H), 7.64 (d, *J* = 7.8 Hz, 2H), 7.61 (t, *J* = 7.9 Hz, 1H), 7.53 (t, *J* = 5.7 Hz, 1H 2H), 7.45 (d, *J* = 15.7 Hz, 1H), 6.97 (d, *J* = 6.4 Hz, 2H) and 3.89 (s, 3H) ppm. ^13^C-NMR (176 MHz, CDCl_3_) δ 190.63, 161.71, 144.73, 138.54, 132.58, 130.26, 128.59, 128.44, 127.65, 119.82, 114.45 and 55.44 ppm. Mass (ESI): *m*/*z* 239 [M + H]^+^; 261 [M + Na]^+^.

(*E*)-3-(4-Ethoxyphenyl)-1-phenylprop-2-en-1-one (**4f**). Yellow powder (74%). Mp. 52 °C (lit. [57] mp. 52–53 °C). FT-IR (KBr): ν (cm^−1^) = 2981, 1653, 1586, 1510, 1475, 1425, 1293, 1174, 1016, 923, 778, 687, 659 and 572 cm^−1^. ^1^H-NMR (700 MHz, CDCl_3_) δ 7.99 (d, *J* = 7.6 Hz, 2H), 7.77 (d, *J* = 15.6 Hz, 1H), 7.58 (d, *J* = 6.4 Hz, 2H), 7.55 (t, *J* = 6.8 Hz, 1H), 7.49 (t, *J* = 7.7 Hz, 2H), 7.39 (d, *J* = 16.1 Hz, 1H), 6.91 (d, *J* = 6.5 Hz, 2H), 4.07 (q, 2H) and 1.42 (t, 3H) ppm. ^13^C-NMR (176 MHz, CDCl_3_) δ 190.82, 161.29, 144.99, 138.72, 132.71, 130.42, 128.73, 128.59, 127.62, 119.84, 115.07, 63.84 and 14.91 ppm. Mass (ESI): *m*/*z* 253 [M + H]^+^; 275 [M + Na]^+^.

(*E*)-3-(2-(Benzyloxy)phenyl)-1-phenylprop-2-en-1-one (**4g**). Yellow powder (74%). Mp. 101 °C (lit. [58] mp. 101 °C). FT-IR (KBr): ν (cm^−1^) = 3055, 3031, 2926, 2875, 2361, 1654, 1566, 1488, 1335, 1281, 1016, 989, 731 and 589 cm^−1^. ^1^H-NMR (700 MHz, CDCl_3_) δ 8.04 (d, *J* = 15.7 Hz, 1H), 7.83 (d, *J* = 7.4 Hz, 2H), 7.73 (d, *J* = 15.8 Hz, 1H), 7.60 (d, *J* = 7.4 Hz, 1H), 7.51 (t, *J* = 7.2 Hz, 1H), 7.48 (d, *J* = 7.3 Hz, 2H), 7.43–7.37 (m, overlapped, 5H), 7.34 (t, *J* = 7.9 Hz, 1H), 7.02 (d, *J* = 9.7 Hz, 2H) and 5.16 (s, 2H) ppm. ^13^C-NMR (176 MHz, CDCl_3_) δ 190.83, 158.03, 140.68, 138.17, 136.22, 132.28, 131.36, 130.92, 128.58, 128.32, 128.27, 128.05, 127.67, 123.95, 123.28, 120.93, 112.22 and 70.36 ppm. Mass (ESI): *m*/*z* 315 [M + H]^+^; 337 [M + Na]^+^.

(*E*)-3-(3-(Benzyloxy)phenyl)-1-phenylprop-2-en-1-one (**4h**). Yellow crystal (77%). Mp. 82 °C (lit. [59] mp. 81–82 °C). FT-IR (KBr): ν (cm^−1^) = 3062, 3033, 2869, 1951, 1661, 1581, 1495, 1314, 1216, 1178, 1018, 990, 738 and 573 cm^−1^. ^1^H NMR (700 MHz, CDCl_3_) δ ^1^H-NMR (700 MHz, CDCl_3_) δ 8.00 (d, *J* = 7.6 Hz, 2H), 7.75 (d, *J* = 15.8 Hz, 1H), 7.57 (t, *J* = 7.4 Hz, 1H), 7.52–7.46 (m, overlapped, 3H), 7.44 (t, overlapped, *J* = 8.0 Hz, 3H), 7.38 (t, *J* = 7.6 Hz, 2H), 7.35–7.30 (overlapped, 2H), 7.23 (d, *J* = 8.4 Hz, 2H), 7.02 (d, *J* = 9.8 Hz, 1H) and 5.10 (s, 2H) ppm. ^13^C-NMR (176 MHz, CDCl_3_) δ 190.31, 158.96, 144.48, 137.98, 136.45, 136.13, 132.63, 129.81, 128.49, 128.45, 128.32, 127.95, 127.33, 122.22, 121.23, 116.91, 114.27 and 69.98 ppm. Mass (ESI): *m*/*z* 315 [M + H]^+^; 337 [M + Na]^+^.

(*E*)-3-(4-(Benzyloxy)phenyl)-1-phenylprop-2-en-1-one (**4i**). Yellow crystal (81%). Mp. 217 °C [lit. [54] mp. 220 °C]. FT-IR (KBr): ν (cm^−1^) = 3087, 3062, 3033, 2884, 1968, 1890, 1651, 1469, 1292, 1117, 920, 826, 606 and 517 cm^−1^. ^1^H-NMR (700 MHz, CDCl_3_) δ 7.99 (d, *J* = 7.5 Hz, 2H), 7.77 (d, *J* = 15.5 Hz, 1H), 7.59 (d, *J* = 8.1 Hz, 2H), 7.55 (t, *J* = 7.5 Hz, 1H), 7.48 (t, *J* = 7.6 Hz, 2H), 7.44–7.36 (overlapped, 5H), 7.33 (t, *J* = 7.3 Hz, 1H), 7.00 (d, *J* = 8.1 Hz, 2H) and 5.10 (s, 2H) ppm. ^13^C-NMR (176 MHz, CDCl_3_) δ 190.61, 160.86, 144.66, 138.52, 136.41, 132.59, 130.26, 128.71, 128.59, 128.44, 128.21, 127.88, 127.50, 119.94, 115.32 and 70.14 ppm. Mass (ESI): *m*/*z* 315 [M + H]^+^; 337 [M + Na]^+^.

(*E*)-1-(3-Methoxyphenyl)-3-phenylprop-2-en-1-one (**4j**). Yellow Crystal (83%). Mp. 75 °C (lit. [60] mp. 75–76 °C). FT-IR (KBr): ν (cm^−1^) = 3051, 1969, 1664, 1607, 1574, 1494, 1447, 1336, 1308, 1287, 1213, 1180, 1014 and 988 cm^−1^. ^1^H-NMR (700 MHz, DMSO-*d*_6_) δ 7.92 (d, *J* = 15.6 Hz, 1H), 7.90 (d, *J* = 8.2 Hz, 1H), 7.89 (d, *J* = 9.0 Hz, 1H), 7.76 (d, *J* = 8.9 Hz, 1H), 7.74 (d, *J* = 15.6 Hz, 1H), 7.61 (s, 1H), 7.49 (t, *J* = 7.9 Hz, 1H), 7.47–7.42 (overlapped, 3H), 7.24 (d, *J* = 8.3 Hz, 1H) and 3.85 (s, 3H) ppm. ^1^H-NMR (176 MHz, DMSO-*d*_6_) δ 189.35, 159.96, 144.53, 139.40, 135.03, 131.07, 130.36, 129.36, 129.31, 122.49, 121.47, 119.64, 113.38 and 55.78 ppm. Mass (ESI): *m*/*z* 239 [M + H]^+^.

(*E*)-3-(4-Chlorophenyl)-1-(3-methoxyphenyl)prop-2-en-1-one (**4k**). Yellow Powder (88%). Mp. 77 °C (lit. [57] mp. 75–76 °C). FT-IR (KBr): ν (cm^−1^) = 3434, 2997, 2938, 2836, 1658, 1575, 1454, 1322, 1198, 1089, 982, 861, 781 and 566 cm^−1^. ^1^H-NMR (700 MHz, DMSO-*d*_6_) δ 7.73 (d, *J* = 15.7 Hz, 1H), 7.57 (d, *J* = 7.6 Hz, 1H), 7.55 (d, *J* = 8.5 Hz, 2H), 7.52 (dd, *J* = 2.8, 1.5 Hz, 1H), 7.46 (d, *J* = 15.7 Hz, 1H), 7.39 (t, *J* = 7.9 Hz, 1H), 7.37 (d, *J* = 8.5 Hz, 2H), 7.12 (ddd, *J* = 8.2, 2.7, 0.9 Hz, 1H) and 3.86 (s, 3H) ppm. ^13^C-NMR (176 MHz, DMSO-*d*_6_) δ 189.94, 159.96, 143.33, 139.43, 136.46, 133.39, 129.64, 129.62, 129.27, 122.50, 121.06, 119.45, 112.90 and 55.52 ppm. Mass (ESI): *m*/*z* 273 [M + H]^+^; 295 [M + Na]^+^.

(*E*)-1-(3-Methoxyphenyl)-3-(4-methoxyphenyl) prop-2-en-1-one (**4l**). Yellowish orange low melting solid (80%); mp. 48 °C (lit. mp. 49 °C [61]; mp. 45–47 °C [62]). FT-IR (KBr): ν (cm^−1^) = 3648, 2995, 2362, 1735, 1683, 1594, 1509, 1456, 1251, 1199, 1171, 1029 and 599 cm^−1^. ^1^H-NMR (700 MHz, DMSO-*d*_6_) δ 7.85 (d, *J* = 6.9 Hz, 2H), 7.78 (d, *J* = 15.5 Hz, 1H), 7.74 (d, *J* = 7.5 Hz, 1H), 7.71 (d, *J* = 15.7 Hz, 1H), 7.59 (s, 1H), 7.47 (t, *J* = 7.9 Hz, 1H), 7.22 (d, *J* = 8.3 Hz, 1H), 7.01 (d, *J* = 6.7 Hz, 2H), 3.84 (s, 3H) and 3.81 (s, 3H) ppm. ^1^H-NMR (176 MHz, DMSO-*d*_6_) δ 161.79, 159.92, 144.50, 139.69, 131.27, 130.26, 121.31, 119.95, 119.35, 114.79, 113.30, 55.77, 55.74, 40.02, 39.90 and 39.78 ppm. Mass (ESI): *m/z*: 269 [M + H]^+^; 291 [M + Na]^+^.

(*E*)-1-(4-Methoxyphenyl)-3-phenylprop-2-en-1-one (**4m**). Yellow crystal (78%). Mp. 91 °C (lit. mp. 61–62 °C [63]; mp. 105–107 °C [64]). FT-IR (KBr): ν (cm^−1^) = 3022, 2973, 2935, 2840, 1654, 1572, 1446, 1337, 1224, 1035, 830, 695, 617 and 563 cm^−1^. ^1^H-NMR (700 MHz, CDCl_3_) δ 8.03 (d, *J* = 8.8 Hz, 2H), 7.79 (d, *J* = 15.6 Hz, 1H), 7.63 (dd, *J* = 7.6, 2.0 Hz, 2H), 7.53 (d, *J* = 15.6 Hz, 1H), 7.40 (d, *J* = 7.0 Hz, 3H), 6.97 (d, *J* = 8.8 Hz, 2H) and 3.87 (s, 3H) ppm. ^13^C-NMR (176 MHz, CDCl_3_) δ 188.53, 163.23, 143.77, 134.89, 130.90, 130.62, 130.13, 128.73, 128.16, 121.69, 113.65 and 55.30 ppm. Mass (ESI): *m*/*z* 239 [M + H]^+^; 261 [M + Na]^+^.

(*E*)-3-(4-Chlorophenyl)-1-(4-methoxyphenyl)prop-2-en-1-one (**4n**). White crystal (83%). Mp. 132 °C (lit. [54] mp. 133 °C). FT-IR (KBr): ν (cm^−1^) = 3012, 2962, 2839, 1917, 1655, 1604, 1491, 1405, 1222, 1177, 1024, 839, 747 and 534 cm^−1^. ^1^H NMR (700 MHz, CDCl_3_) δ ^1^H NMR (700 MHz, CDCl_3_) δ 8.03–8.00 (dt, 2H), 7.73 (d, *J* = 15.6 Hz, 1H), 7.55 (dt, 2H), 7.50 (d, *J* = 15.6 Hz, 1H), 7.37 (dt, 2H), 6.97 (dt, 2H) and 3.88 (s, 3H) ppm. ^13^C-NMR (176 MHz, CDCl_3_) δ 188.57, 163.71, 142.63, 136.35, 133.75, 131.09, 131.00, 129.67, 129.37, 122.46, 114.07 and 55.69 ppm. Mass (ESI): *m*/*z* 273 [M + H]^+^; 295 [M + Na]^+^.

(*E*)-1,3-bis(4-Methoxyphenyl)prop-2-en-1-one (**4o**). Yellow crystal (73%). Mp. 100 °C (lit. [54] mp. 99 °C). FT-IR (KBr): ν (cm^−1^) = 3303, 3014, 2965, 2841, 2029, 1886, 1592, 1419, 1213, 1166, 1014, 845, 749 and 523 cm^−1^. ^1^H-NMR (700 MHz, DMSO-*d*_6_) δ 8.14 (d, *J* = 9.0 Hz, 2H), 7.83 (d, *J* = 8.2 Hz, 2H), 7.79 (d, *J* = 15.4 Hz, 1H), 7.67 (d, *J* = 15.3 Hz, 1H), 7.07 (d, *J* = 9.3 Hz, 2H), 7.00 (d, *J* = 9.1 Hz, 2H), 3.85 (s, 3H) and 3.81 (s, 3H) ppm. ^13^C-NMR (176 MHz, DMSO-*d*_6_) δ 187.73, 163.54, 161.69, 143.60, 131.26, 131.15, 131.14, 127.93, 119.96, 114.85, 114.44, 56.02 and 55.84 ppm. Mass (ESI): *m*/*z* 269 [M + H]^+^; 291 [M + Na]^+^.

(*E*)-3-(3-(Benzyloxy)phenyl)-1-(3-methoxyphenyl)prop-2-en-1-one (**4p**). White powder (78%). Mp. 78 °C. FT-IR (KBr): ν (cm^−1^) = 3062, 3029, 2975, 2871, 1697, 1662, 1461, 1310, 1242, 1054, 1026, 842, 782 and 680 cm^−1^. ^1^H-NMR (700 MHz, DMSO-*d*_6_) δ 7.75 (d, *J* = 15.6 Hz, 1H), 7.58 (d, *J* = 7.5 Hz, 1H), 7.52 (s, 1H), 7.46 (d, overlapped, *J* = 15.6 Hz, 1H), 4.44 (d, *J* = 8.4 Hz, 2H), 7.44 (overlapped, Hz, 1H), 7.39 (m, overlapped, 3H), 7.33 (t, overlapped, *J* = 6.7 Hz, 1H), 7.32 (t, overlapped, 1H), 7.23 (s, 1H), 7.12 (d, *J* = 8.0 Hz, 1H), 7.02 (d, *J* = 8.1 Hz, 1H), 5.10 (s, 2H) and 3.87 (s, 3H) ppm. ^13^C-NMR (176 MHz, DMSO) δ 185.46, 155.19, 154.42, 139.96, 134.83, 131.91, 131.59, 125.27, 124.86, 123.95, 123.41, 122.82, 122.79, 117.69, 116.73, 116.34, 114.63, 112.40, 109.71, 108.13, 65.44 and 50.77 ppm. Mass (ESI): *m*/*z* 345 [M + H]^+^; 367 [M + Na]^+^.

(*E*)-3-(4-(Benzyloxy)phenyl)-1-(3-methoxyphenyl)prop-2-en-1-one (**4q**). Yellowish powder (72%). Mp. 102 °C. FT-IR (KBr): ν (cm^−1^) = 3031, 3008, 2908, 2830, 1945, 1904, 1654, 1509, 1428, 1294, 1033, 858, 790 and 519 cm^−1^. ^1^H-NMR (700 MHz, DMSO-*d*_6_) δ 7.77 (d, *J* = 15.5 Hz, 1H), 7.60–7.56 (overlapped, 3H), 7.52 (s, 1H), 7.44–7.41 (overlapped, 2H), 7.41–7.36 (overlapped, 4H), 7.33 (t, *J* = 7.3 Hz, 1H), 7.11 (d, *J* = 5.4 Hz, 1H), 6.99 (d, *J* = 8.2 Hz, 2H), 5.10 (s, 2H) and 3.86 (s, 3H) ppm. ^13^C-NMR (176 MHz, DMSO-*d*_6_) δ 190.23, 160.82, 159.84, 144.62, 139.87, 136.36, 130.23, 129.48, 128.65, 128.16, 127.81, 127.45, 120.93, 119.89, 119.06, 115.27, 112.77, 70.08 and 55.45 ppm. Mass (ESI): *m/z* 345 [M + H]^+^; 367 [M + Na]^+^.

(*E*)-3-(3-(Benzyloxy)phenyl)-1-(4-methoxyphenyl)prop-2-en-1-one (**4r**). White powder (69%). Mp. 130 °C (lit. [59,65] mp. 136–138 °C). FT-IR (KBr): ν (cm^−1^) = 3064, 2931, 2838, 1655, 1583, 1496, 1311, 1251, 1178, 1018, 985, 835, 798, and 585 cm^−1^. ^1^H-NMR (700 MHz, CDCl_3_) δ 8.02 (d, *J* = 8.9 Hz, 2H), 7.74 (d, *J* = 15.4 Hz, 1H), 7.49 (d, *J* = 15.8 Hz, 1H), 7.44 (d, *J* = 7.5 Hz, 2H), 7.39 (t, *J* = 7.6 Hz, 2H), 7.33 (t, overlapped, *J* = 7.8 Hz, 1H), 7.32 (t, overlapped, *J* = 7.8 Hz, 1H), 7.23 (s, *J* = 8.2 Hz, 2H), 7.01 (d, *J* = 8.2 Hz, 1H), 6.97 (d, *J* = 9.2 Hz, 2H), 5.10 (s, 2H) and 3.88 (s, 3H) ppm. ^13^C-NMR (176 MHz, CDCl_3_) δ 188.47, 163.27, 158.94, 143.59, 136.48, 136.34, 130.88, 130.64, 129.77, 128.48, 127.93, 127.34, 122.03, 121.14, 116.65, 114.23, 113.67, 69.97 and 55.32 ppm. Mass (ESI): *m*/*z* 345 [M + H]^+^.

(*E*)-3-(4-(Benzyloxy)phenyl)-1-(4-methoxyphenyl)prop-2-en-1-one (**4s**). White powder (77%). Mp. 117 °C (lit. [66,67] mp. 135–137 °C). FT-IR (KBr): ν (cm^−1^) = 3423, 1626, 1600, 1572, 1509, 1421, 1306, 1242, 1175, 1079, 1015, 836, 735 and 598 cm^−1^. ^1^H-NMR (700 MHz, CDCl_3_) δ 8.01 (d, *J* = 8.9 Hz, 2H), 7.76 (d, *J* = 15.6 Hz, 1H), 7.58 (d, *J* = 8.9 Hz, 2H), 7.44–7.36 (overlapped, 5H), 7.33 (t, *J* = 7.2 Hz, 1H), 6.99 (d, *J* = 9.1 Hz, 2H), 6.96 (d, *J* = 8.9 Hz, 2H), 5.10 (s, 2H) and 3.87 (s, 3H) ppm. ^13^C-NMR (176 MHz, CDCl_3_) δ 188.58, 163.09, 160.47, 143.56, 136.26, 131.17, 130.52, 129.92, 128.49, 127.99, 127.87, 127.30, 119.50, 115.08, 113.60, 69.92 and 55.30 ppm. Mass (ESI): *m*/*z* 44 [M] ^+^; 345 [M + H]^+^; 367 [M + Na]^+^.

(*E*)-3-(4-(Benzyloxy)-3-methoxyphenyl)-1-phenylprop-2-en-1-one (**4t**). Yellow crystal (78%). Mp. 101 °C. FT-IR (KBr): ν (cm^−1^) = 3430, 3019, 2944, 2860, 1656, 1588, 1518, 1456, 1237, 1135, 1011, 781, 695 and 593 cm^−1^. ^1^H-NMR (700 MHz, CDCl_3_) δ 7.98 (d, *J* = 7.4 Hz, 2H), 7.73 (d, *J* = 15.4 Hz, 1H), 7.56 (t, *J* = 7.4 Hz, 1H), 7.48 (t, *J* = 7.6 Hz, 2H), 7.42 (d, *J* = 7.6 Hz, 2H), 7.37 (s, 1H), 7.36 (d, *J* = 8.8 Hz, 2H), 7.31 (t, *J* = 7.3 Hz, 1H), 7.16 (s, 1H), 7.15 (d, *J* = 8.8 Hz, 1H), 6.89 (d, *J* = 8.1 Hz, 1H), 5.20 (s, 2H) and 3.94 (s, 3H) ppm. ^13^C NMR (176 MHz, CDCl_3_) δ 190.47, 150.38, 149.60, 144.82, 138.29, 136.32, 132.39, 128.47, 128.39, 128.25, 128.02, 127.87, 127.03, 122.73, 120.02, 113.28, 110.51, 70.68 and 55.91 ppm. Mass (ESI): *m/z* 345 [M + H]^+^; 367 [M + Na]^+^.

(*E*)-3-(4-(Benzyloxy)-3-methoxyphenyl)-1-(3-methoxyphenyl)prop-2-en-1-one (**4u**). Yellow powder (81%). Mp. 85 °C. FT-IR (KBr): ν (cm^−1^) = 3423, 3010, 2940, 2863, 2838, 1886, 1656, 1577, 1515, 1430, 1285, 1261, 1132, 1018, 997, 791, 621 and 561 cm^−1^. ^1^H-NMR (700 MHz, CDCl_3_) δ 7.73 (d, *J* = 15.7 Hz, 1H), 7.57 (d, *J* = 7.4 Hz, 1H), 7.52 (s, 1H), 7.42 (d, *J* = 7.5 Hz, 2H), 7.40–7.32 (overlapped, 5H), 7.30 (t, *J* = 7.4 Hz, 1H), 7.16 (s, 1H), 7.14 (d, *J* = 8.8 Hz, 1H), 7.10 (d, *J* = 8.6 Hz, 1H), 6.89 (d, *J* = 8.2 Hz, 1H), 5.19 (s, 2H), 3.94 (s, 3H) and 3.86 (s, 3H) ppm. ^13^C-NMR (176 MHz, CDCl_3_) δ 190.10, 159.65, 150.37, 149.57, 144.81, 139.66, 136.30, 129.30, 128.44, 127.98, 127.84, 127.01, 122.73, 120.75, 119.97, 118.78, 113.24, 112.70, 110.49, 70.64, 55.88 and 55.28 ppm. Mass (ESI): *m/z* 375 [M + H]^+^; 397 [M + Na]^+^.

(*E*)-3-(4-(Benzyloxy)-3-methoxyphenyl)-1-(4-methoxyphenyl)prop-2-en-1-one (**4v**). Yellow powder (76%). Mp. 92 °C. FT-IR (KBr): ν (cm^−1^) =3423, 2959, 2838, 1674, 1650, 1584, 1505, 1464, 1424, 1158, 1026, 807, 698 and 553 cm^−1^. ^1^H-NMR (700 MHz, DMSO-*d*_6_) δ 8.16 (d, *J* = 8.9 Hz, 2H), 7.82 (d, *J* = 15.5 Hz, 1H), 7.65 (d, *J* = 15.5 Hz, 1H), 7.54 (s, 1H), 7.45 (d, *J* = 7.5 Hz, 2H), 7.40 (t, *J* = 7.6 Hz, 2H), 7.34 (overlapped, 2H), 7.10 (d, *J* = 8.3 Hz, 1H), 7.08 (d, *J* = 8.8 Hz, 2H), 5.15 (s, 2H), 3.87 (s, 3H) and 3.86 (s, 3H) ppm. ^13^C-NMR (176 MHz, DMSO-*d*_6_) δ 187.64, 163.46, 150.44, 149.67, 143.96, 137.15, 131.21, 131.06, 128.84, 128.34, 128.31, 128.27, 123.91, 120.10, 114.33, 113.49, 111.46, 70.20, 56.19 and 55.94 ppm. Mass (ESI): *m/z* 375 [M + H]^+^; 397 [M + Na]^+^.

### 3.4. Biological Evaluation Assay

#### 3.4.1. In Vitro Cytotoxicity Assay of **4a**–**4v**

The cytotoxic potential of the synthesized compounds (**4a**–**4v**), along with reference compounds (doxorubicin and sorafenib), was evaluated using the colorimetric MTT assay. This assessment was conducted across multiple cancer cell lines (HCT-116, MDA-MB-231, HeLa, and MCF-7) and one normal cell line (WI-38) in accordance with a previously reported methodology [68,69]. Specifically, the cells were seeded in 96-well plates at a density of 1.0 × 10^4^ cells per well, supplemented with 10% fetal bovine serum (FBS), an antibiotic cocktail containing streptomycin (100 µg/mL) and penicillin (100 units/mL), and RPMI 11,640 medium. Following a 48 h incubation period at 37 °C, 5% CO_2_, and 100% relative humidity, the cells were treated with increasing concentrations of the synthesized compounds, as well as doxorubicin and sorafenib, and further incubated for 24 h. Afterward, 20 µL of MTT solution was added to each well and left in the incubator for 4 h to allow for formazan formation. Subsequently, 100 µL of dimethyl sulfoxide was introduced to dissolve the insoluble formazan. Finally, the absorbance of the samples was measured at 570 nm using a BioTek EXL 800 plate reader (Agilent Technologies, Inc., Santa Clara, CA, USA), and the relative cell viability percentage was calculated as (A570 of treated samples/A570 of untreated sample) × 100.

#### 3.4.2. In Vitro Enzyme Assays of **4a**, **4b**, **4q**, and **4v**

The inhibitory activities of four chalcone derivatives (**4a**, **4b**, **4q**, and **4v**) against the enzymes EGFR, Her2, VEGFR-2, and CDK2 were evaluated using specific human ELISA kits, as described in the referenced methods [68,69]. In the assay, various concentrations of the synthesized compounds were added to a 96-well plate, along with the specific antibodies for each kinase enzyme. The plate was then incubated at room temperature for 2.5 h to allow the binding of the compounds to the respective enzyme targets. After the incubation period, the 96-well plate was washed to remove any unbound components. Subsequently, 100 µL of an in-house prepared biotin antibody was added to each well and incubated at ambient temperature for 1 h. Following another wash step, 100 µL of streptavidin solution was added to each well and left to incubate for 45 min at ambient temperature. After a final wash, 100 µL of 3,3′,5,5′-tetramethylbenzidine (TMB) substrate reagent was applied to initiate a colorimetric reaction, and the plate was incubated at ambient temperature for 30 min. Finally, 50 µL of a stop solution was added to halt the reaction, and the absorbance was directly measured at 450 nm. To determine the concentrations of the compounds, a standard curve was constructed by plotting concentration values on the *X*-axis and corresponding absorbance values on the *Y*-axis. Non-linear regression curve fit (four-parameter logistic regression) was used to plot the dose-response curves and calculate IC_50_ values using GraphPad Prism 8. Dose-response data were obtained from triplicate experiments, and the mean values were fitted to the curve.

#### 3.4.3. In Vitro Anti-Estrogenic Activity Assay of **4a**, **4b**, **4q**, and **4v**

The estrogen receptor-based CALUX mammalian cell bioassay utilizes MCF-7 and estradiol-dependent MCF7 subline MCF7a cell lines, which have been stably transfected with an estrogen-responsive luciferase reporter plasmid. This CALUX cell line responds to estrogenic chemicals by inducing the expression of firefly luciferase. The CALUX bioassay has advantages over the yeast-based YES bioassay commonly used to detect estrogenic chemicals in that it expresses mRNA for ER isoforms alpha and beta, in contrast to the YES assay, which is usually only transfected with ER alpha. The CALUX bioassay was used to determine the concentration-dependent agonist (pesticide alone) and antagonist (pesticide in the presence of estradiol) effects of pyrethroids used in these studies, and chemical exposure and luciferase analyses were carried out as described in detail [70]. The source of 17*β* estradiol used as a positive control in all CALUX bioassays was from Sigma Chemical Co. (St. Louis, MO, USA).

#### 3.4.4. In Vitro Aromatase Assay of **4a**, **4b**, **4q**, and **4v**

In vitro aromatase assay was performed using the BioVision Aromatase Activity Assay Kit (Catalog # K983-100; BioVision, Milpitas, CA, USA). Microsomal samples were isolated from tissue using the Microsome Isolation Kit (Cat. #K249; BioVision, Milpitas, CA, USA). A standard curve was established by diluting the Fluorescence Standard provided in the kit to create a 0.5 pmole/µL solution, followed by setting up a series of standards in a white 96-well plate, adjusting the total volume to 100 µL with aromatase assay buffer. For each reaction well, a 2X concentrated mix was prepared by adding 2–48 µL of the microsomal sample and 2 µL of the NADPH generating system, bringing the final volume to 50 µL with aromatase assay buffer. Background controls (no enzyme) and positive controls (with recombinant aromatase) were included. To assess inhibition, 1 µM of letrozole, the aromatase inhibitor included in the kit, was added to designated wells. After a 10 min incubation at 37 °C, 30 µL of the substrate/NADP+ mixture was added to each well, and fluorescence was measured immediately in kinetic mode for 60 min at 37 °C. Finally, the change in fluorescence was calculated to determine the specific activity of aromatase using the formula provided in the kit protocol.

### 3.5. In Silico Studies of ***4q***

#### 3.5.1. Molecular Docking of **4q**

ChemOffice application (ChemDraw 16.0) was used to draw the structure of compound **4q** with the proper 2D orientation. The structure of the reference standards was obtained from PubChem. ChemBio3D was used to minimize the energy of each molecule. The X-ray crystal structures of the main kinase domain in complex with other molecules were collected from the RCSB Protein Data Bank. (PDB id = 3 pp0 for HER2, 4ASD for VEGFR2, 3POZ for EGFR, 6INL for CDK2 and 3ERT and 1A52 for Estrogen receptor). The PyMol software package (version 2.5.2) was utilized to remove all water molecules, heteroatoms, and inhibitors from the structure [71]. After that, the crystal structures of the receptor were optimized and evaluated based on their lowest energy using the Swiss-PDB Viewer software (version 4.1.0). Docking studies were performed using Autodock Vina [72] with the assistance of UCSF Chimera [73]. Domains and ligands that had previously been minimized were then used as input in Autodock vina. Hydrogens and Gasteiger charges were added to the ligand before performing a docking analysis. The grid box in AutoDock Vina was set up to focus on the active site of the main domain. Following docking, UCSF ChimeraX and Discovery Studio Visualizer were used to visualize the interactions. The ligands were shown in a variety of colors while interacting residues and hydrogen bonds were shown to establish the hypothesis.

#### 3.5.2. In Silico ADME Studies of the Compounds **4a**–**4v**

The ADMET (Absorption, Distribution, Metabolism, Excretion, and Toxicity) profiles of the selected synthesized compounds were predicted using the in silico pkCSM descriptors algorithm protocol according to the reported method [68,69,74].

## 4. Conclusions

Synthesized *O*-alkyl (*E)*-chalcone derivatives (**4a**–**4v**) demonstrate significant potential as anticancer agents, particularly in targeting breast cancer cell lines. The variation in cytotoxicity observed among the compounds highlights the importance of structural modifications, with compounds **4b** and **4q** standing out for their potent activity and selectivity against cancer cells while minimizing effects on non-cancerous cells. The ability of these derivatives to inhibit key protein kinases, such as EGFR and VEGFR-2, further supports their role as effective therapeutic agents. Additionally, the notable anti-estrogenic activity observed in several derivatives suggests that they may offer dual therapeutic benefits in the treatment of hormone-dependent cancers. We interpret the IC_50_ values for enzyme inhibitory and anti-aromatase activity, derived from non-linear curves, as approximate, emphasizing the importance of cautious analysis in our findings. Compound **4q** demonstrated a higher binding affinity and similar interactions as estrogen and tamoxifen. Overall, this study establishes a promising foundation for the further development of *O*-alkyl (*E)*-chalcone derivatives as novel cancer therapeutics and emphasizes the need for continued research into their mechanisms of action and optimization for enhanced efficacy and selectivity in clinical applications.

## Figures and Tables

**Table 1:** In vitro cytotoxicity of compounds **4a**–**4v** against selected cancer cell lines.

Compound	Cytotoxicity IC_50_ (µM)								
	HCT-116	SI	MDA-MB-231	SI	HeLa	SI	MCF-7	SI	WI-38
**4a**	9.72 ± 0.7	3.7	7.06 ± 0.5	5.1	15.38 ± 1.3	2.4	5.16 ± 0.4	7.0	36.28 ± 2.2
**4b**	6.59 ± 0.3	8.3	4.93 ± 0.3	11.1	13.07 ± 1.1	4.2	2.08 ± 0.1	26.2	54.59 ± 3.1
**4c**	28.35 ± 1.8	3.5	35.86 ± 2.1	2.8	42.46 ± 2.5	2.4	23.51 ± 1.6	4.3	>100
**4d**	36.01 ± 2.2	2.1	43.59 ± 2.3	1.8	39.43 ± 2.3	2.0	29.67 ± 1.9	2.6	77.26 ± 3.8
**4e**	20.73 ± 1.5	4.8	24.30 ± 1.8	4.1	29.52 ± 2.0	3.4	16.90 ± 1.3	5.9	>100
**4f**	57.59 ± 3.3	1.1	46.12 ± 2.4	1.4	49.60 ± 2.8	1.3	35.28 ± 2.1	1.8	64.65 ± 3.6
**4g**	53.96 ± 3.0	0.5	59.19 ± 3.5	0.5	36.47 ± 2.4	0.8	48.43 ± 2.6	0.6	27.81 ± 1.9
**4h**	46.81 ± 2.7	1.8	53.80 ± 3.3	1.6	25.63 ± 1.8	3.3	42.07 ± 2.4	2.0	83.39 ± 4.2
**4i**	>100	1.0	>100	1.0	>100	1.0	85.54 ± 4.3	1.2	>100
**4j**	24.60 ± 1.7	3.2	29.34 ± 1.9	2.7	34.91 ± 2.2	2.2	18.83 ± 1.5	4.2	78.22 ± 3.9
**4k**	>100	1.0	>100	1.0	>100	1.0	>100	1.0	>100
**4l**	83.96 ± 4.2	0.2	91.72 ± 4.5	0.2	88.83 ± 4.6	0.2	75.38 ± 3.8	0.2	16.24 ± 1.3
**4m**	59.62 ± 3.5	1.5	45.93 ± 2.6	1.9	63.65 ± 3.7	1.4	33.04 ± 2.0	2.7	87.62 ± 4.6
**4n**	66.08 ± 3.8	0.9	48.59 ± 2.8	1.3	74.51 ± 3.9	0.8	36.97 ± 2.3	1.7	61.53 ± 3.4
**4o**	89.17 ± 4.5	0.3	73.48 ± 4.0	0.3	>100	0.2	61.85 ± 3.4	0.4	24.18 ± 1.7
**4p**	39.84 ± 2.4	2.3	68.42 ± 3.7	1.3	45.21 ± 2.4	2.0	57.11 ± 3.2	1.6	92.06 ± 4.9
**4q**	17.06 ± 1.3	5.9	13.58 ± 1.1	7.4	22.64 ± 1.7	4.4	11.56 ± 0.9	8.7	>100
**4r**	62.60 ± 3.7	1.6	51.84 ± 2.9	1.9	57.03 ± 3.2	1.8	39.34 ± 2.4	2.5	>100
**4s**	73.53 ± 3.9	0.3	82.37 ± 4.1	0.3	86.96 ± 4.3	0.3	64.19 ± 3.5	0.3	22.34 ± 1.6
**4t**	68.91 ± 3.7	0.6	64.09 ± 3.8	0.6	59.28 ± 3.4	0.7	55.62 ± 2.9	0.7	40.06 ± 2.5
**4u**	95.27 ± 4.9	0.4	>100	0.4	>100	0.4	82.75 ± 4.1	0.5	38.95 ± 2.4
**4v**	14.53 ± 1.1	5.2	10.24 ± 0.8	7.3	18.16 ± 1.5	4.1	8.67 ± 0.6	8.7	75.17 ± 3.9
**Doxorubicin**	5.23 ± 0.3	1.3	3.18 ± 0.1	2.1	5.57 ± 0.4	1.2	4.17 ± 0.2	1.6	6.72 ± 0.5
**Sorafenib**	5.47 ± 0.3	1.9	7.64 ± 0.4	1.4	8.04 ± 0.5	1.3	7.26 ± 0.3	1.5	10.65 ± 0.8

IC_50_ values are the mean ± SD of triplicate measurements; SI = selectivity index = IC_50_ of normal cell line (WI-38)/IC_50_ of cancer cell line. Average values were used to calculate the selectivity index.

**Table 2:** In vitro protein kinase inhibition assay of most active compounds **4a**, **4b**, **4q**, and **4v** against EGFR, Her2, VEGFR-2, and CDK2.

Compound	Protein Kinase Inhibition [IC_50_ (µM) ^a^/Inhibition % at 0.1 µM ^b^]			
	EGFR	HER2	VEGFR-2	CDK2
**4a**	0.367 ± 0.012	0.691 ± 0.027	1.548 ± 0.053	0.84 ± 0.026
**4b**	54 ^c^	0.387 ± 0.015	0.163 ± 0.006	0.288 ± 0.009
**4q**	0.151 ± 0.005	48 ^c^	0.287 ± 0.01	41.9 ^c^
**4v**	0.671 ± 0.022	1.568 ± 0.061	1.824 ± 0.062	1.032 ± 0.032
**Erlotinib**	0.056 ± 0.002	63.9 ^c^	-	-
**Sorafenib**	-	-	57 ^c^	-
**Dinaciclib**	-	-	-	60 ^c^

^a^ Inhibition concentration (µM) at 50%; ^b^ Inhibition % at the concentration of 0.1 µM; ^c^ The data between the highest and lowest values; IC_50_ values are the mean ± SD of triplicate measurements.

**Table 3:** In vitro anti-estrogenic activity assay of most active compounds **4a**, **4b**, **4q**, and **4v** on MCF-7 and MCF-7a cell lines.

Compound	Cell Proliferation			
	MCF-7		MCF-7a	
	**Cell Count**	**%**	**Cell Count**	**%**
**4a**	14,553 ± 518	79	21,155 ± 611	102
**4b**	6213 ± 221	34	20,669 ± 597	99
**4q**	6874 ± 245	37	20,630 ± 595	99
**4v**	13,611 ± 485	73	20,397 ± 589	98
**Tamoxifen**	3185 ± 113	17	20,698 ± 597	100
**17β-E2** (Control)	18,524 ± 659	100	20,776 ± 600	100

Cell count is the mean ± SD of triplicate measurements.

**Table 4:** In vitro aromatase assay of most active compounds **4a**, **4b**, **4q**, and **4v**.

Compound	Aromatase Inhibition [IC_50_ (µM) ^a^/Inhibition % at 0.1 µM ^b^]
**4a**	0.434 ± 0.014
**4b**	1.634 ± 0.054
**4q**	0.801 ± 0.03
**4v**	0.357 ± 0.012
**Letrozole**	55 ^c^

^a^ Inhibition concentration (µM) at 50%; ^b^ Inhibition % at the concentration of 0.1 µM; ^c^ The data indicated median of three individual experimental values; IC_50_ values are the mean ± SD of triplicate measurements.

**Table 5:** Binding affinity, interacting residues, and types of interaction mediated by compound **4q** and reference compounds.

Entry	Binding Affinity (kcal/mol)				Hydrogen Bonds			
	EGFR	HER2	VEGFR2	CDK2	EGFR	HER2	VEGFR2	CDK2
**4q**	−10.0	−10.5	−10.1	−8.5	-	-	-	Leu83
Erlotinib	−7.3	−8.3			Met793, Lys745, Asp855	-	-	-
Sorafenib			−10.7		-	-	Glu885, Asp1046, Cys919	-
Dinaciclib				−9.1	-	-	-	Leu83, Lys89

**Table 6:** In silico drug-likeness property analysis of compounds **4a**–**4v**.

Entry	MW ^a^	LogP	HBA ^b^	HBD ^c^	SA ^d^	WS ^e^	IA ^f^	SP ^g^	BBB ^h^	CNS ^i^	CYP ^j^	CYP ^k^	TC ^l^	MTD ^m^	ORAT ^n^	ORCT ^o^	SS ^p^
**4a**	208.3	3.6	1.0	0.0	95.1	−4.6	99.1	−1.8	0.6	−0.9	No	No	0.2	1.8	1.9	1.6	Yes
**4b**	224.3	3.3	2.0	1.0	99.8	−4.0	96.2	−2.3	0.2	−1.2	No	No	0.1	1.7	2.0	2.5	Yes
**4c**	238.3	3.6	2.0	0.0	106.5	−4.6	99.1	−2.1	0.4	−1.0	No	No	0.3	1.7	2.0	2.4	Yes
**4d**	238.3	3.6	2.0	0.0	106.5	−4.6	99.1	−2.1	0.4	−1.0	No	No	0.2	1.7	2.0	2.4	Yes
**4e**	238.3	3.6	2.0	0.0	106.5	−4.6	99.1	−2.1	0.4	−1.0	No	No	0.2	1.7	2.0	2.4	Yes
**4f**	252.3	4.0	2.0	0.0	112.9	−4.9	98.7	−2.2	0.5	−1.0	No	No	0.2	1.7	2.0	2.5	Yes
**4g**	314.4	5.2	2.0	0.0	141.6	−5.8	98.3	−2.6	0.6	−0.6	No	Yes	0.2	1.6	2.1	2.7	No
**4h**	314.4	5.2	2.0	0.0	141.6	−5.8	98.3	−2.6	0.6	−0.6	No	Yes	0.2	1.6	2.1	2.7	No
**4i**	314.4	5.2	2.0	0.0	141.6	−5.8	98.3	−2.6	0.6	−0.6	No	Yes	0.2	1.6	2.1	2.7	No
**4j**	238.3	3.6	2.0	0.0	106.5	−4.6	99.1	−2.1	0.4	−1.0	No	No	0.2	1.7	2.0	2.4	Yes
**4k**	272.7	4.2	2.0	0.0	116.8	−5.3	97.4	−2.2	0.4	−1.0	No	No	0.1	1.6	2.2	2.3	Yes
**4l**	268.3	3.6	3.0	0.0	118.0	−4.6	99.1	−2.5	0.3	−1.0	No	No	0.2	1.6	2.0	2.3	No
**4m**	238.3	3.6	2.0	0.0	106.5	−4.6	99.1	−2.1	0.4	−1.0	No	No	0.2	1.7	2.0	2.4	Yes
**4n**	272.7	4.2	2.0	0.0	116.8	−5.4	97.4	−2.2	0.4	−1.0	No	No	0.0	1.6	2.2	2.3	No
**4o**	268.3	3.6	3.0	0.0	118.0	−4.6	99.1	−2.5	0.3	−1.0	No	No	0.2	1.6	2.0	2.3	No
**4p**	344.4	5.2	3.0	0.0	153.1	−5.9	98.3	−2.7	0.3	−0.7	No	Yes	0.2	1.5	2.1	2.5	No
**4q**	344.4	5.2	3.0	0.0	153.1	−5.9	98.3	−2.7	0.3	−0.7	No	Yes	0.2	1.5	2.1	2.5	No
**4r**	344.4	5.2	3.0	0.0	153.1	−5.9	98.3	−2.7	0.3	−0.7	No	Yes	0.2	1.5	2.1	2.5	No
**4s**	344.4	5.2	3.0	0.0	153.1	−5.9	98.3	−2.7	0.3	−0.7	No	Yes	0.2	1.5	2.1	2.5	No
**4t**	344.4	5.2	3.0	0.0	153.1	−5.9	98.3	−2.7	0.3	−0.7	No	Yes	0.2	1.5	2.1	2.5	No
**4u**	374.4	5.2	4.0	0.0	164.5	−6.0	98.3	−2.8	0.1	−0.8	No	Yes	0.2	1.4	2.2	2.4	No
**4v**	374.4	5.2	4.0	0.0	164.5	−6.0	98.3	−2.8	0.1	−0.8	No	Yes	0.2	1.4	2.2	2.4	No
**So ^q^**	464.8	5.5	4.0	3.0	185.1	−6.6	88.5	−3.0	−1.4	−1.9	No	Yes	−0.2	0.7	3.2	1.1	No
**Do ^r^**	543.5	0.0	12.0	6.0	222.1	−3.9	55.8	−2.8	−1.6	−4.1	No	Yes	1.0	−0.1	1.8	2.1	No

^a^ Molecular weight in Dalton (acceptable range: <500); ^b^ Hydrogen bond acceptor (acceptable range: ≤10); ^c^ Hydrogen bond donor (acceptable range: ≤5); ^d^ Surface area; ^e^ Water solubility (log mol/L); ^f^ Intestinal absorption (human)%; ^g^ Skin permeability; ^h^ BBB permeability; ^i^ CNS permeability; ^j^ CYP2D6 substrate; ^k^ CYP3A4 substrate; ^l^ Total clearance (log ml/min/kg); ^m^ Max. tolerated dose (human) (log mg/kg/day); ^n^ Oral rat acute toxicity (LD50) (mol/kg); ^o^ Oral rat chronic toxicity (LOAEL) (log mg/kg, bw/day); ^p^ Skin sensitization; ^q^ Sorafenib; ^r^ Doxorubicin.

## Data Availability

Data are contained within the article.

## Supplementary Materials

The following supporting information can be downloaded at https://www.mdpi.com/article/10.3390/ijms26020833/s1.
