# Supplementary material for: Synthesis and Anticancer Evaluation of O-Alkylated (E)-Chalcone Derivatives: A Focus on Estrogen Receptor Inhibition
Source: Int J Mol Sci. 2025 Jan 20;26(2):833. doi: 10.3390/ijms26020833 (PMC11766267; doi:10.3390/ijms26020833)

Synthesis and Anticancer Evaluation of *O*-Alkylated (*E*)-Chalcone Derivatives: A Focus on Estrogen Receptor Inhibition

Alwah R. Al-Ghamdi[1], Wahid U. Ahmed[2], Reem I. Al-Wabli[1], Maha S. Al-Mutairi[1],* and A. F. M. Motiur Rahman[1],*

[1]Department of Pharmaceutical Chemistry, College of Pharmacy, King Saud University, Riyadh 11451, Saudi Arabia

[2]School of Pharmaceutical Sciences, Zhengzhou University, Zhengzhou, Henan 450001, China

*Correspondence: Maha S. Al-Mutairi (malmutbiri@ksu.edu.sa) A F M Motiur Rahman (afmrahman@ksu.edu.sa)

Entry	Content	Page no.	Entry	Content	Page no.
**4a**	IR Spectrum of **4a**	5	**4f**	^{13}C-NMR Spectrum of **4f**	27
**4a**	^{1}H-NMR Spectrum of **4a**	6	**4f**	Mass Spectrum of **4f**	28
**4a**	^{13}C-NMR Spectrum of **4a**	7	**4g**	IR Spectrum of **4g**	29
**4a**	Mass Spectrum of **4a**	8	**4g**	^{1}H-NMR Spectrum of **4g**	30
**4b**	IR Spectrum of **4b**	9	**4g**	^{13}C-NMR Spectrum of **4g**	31
**4b**	^{1}H-NMR Spectrum of **4b**	10	**4g**	Mass Spectrum of **4g**	32
**4b**	^{13}C-NMR Spectrum of **4b**	11	**4h**	IR Spectrum of **4h**	33
**4b**	Mass Spectrum of **4b**	12	**4h**	^{1}H-NMR Spectrum of **4h**	34
**4c**	IR Spectrum of **4c**	13	**4h**	^{13}C-NMR Spectrum of **4h**	35
**4c**	^{1}H-NMR Spectrum of **4c**	14	**4h**	Mass Spectrum of **4h**	36
**4c**	^{13}C-NMR Spectrum of **4c**	15	**4i**	IR Spectrum of **4i**	37
**4c**	Mass Spectrum of **4c**	16	**4i**	^{1}H-NMR Spectrum of **4i**	38
**4d**	IR Spectrum of **4d**	17	**4i**	^{13}C-NMR Spectrum of **4i**	39
**4d**	^{1}H-NMR Spectrum of **4d**	18	**4i**	Mass Spectrum of **4i**	40
**4d**	^{13}C-NMR Spectrum of **4d**	19	**4j**	IR Spectrum of **4j**	41
**4d**	Mass Spectrum of **4d**	20	**4j**	^{1}H-NMR Spectrum of **4j**	42
**4e**	IR Spectrum of **4e**	21	**4j**	^{13}C-NMR Spectrum of **4j**	43
**4e**	^{1}H-NMR Spectrum of **4e**	22	**4j**	Mass Spectrum of **4j**	44
**4e**	^{13}C-NMR Spectrum of **4e**	23	**4k**	IR Spectrum of **4k**	45
**4e**	Mass Spectrum of **4e**	24	**4k**	^{1}H-NMR Spectrum of **4k**	46
**4f**	IR Spectrum of **4f**	25	**4k**	^{13}C-NMR Spectrum of **4k**	47
**4f**	^{1}H-NMR Spectrum of **4f**	26	**4k**	Mass Spectrum of **4k**	48

Entry	Content	Page no.	Entry	Content	Page no.
**4l**	IR Spectrum of **4l**	49	**4q**	^{13}C-NMR Spectrum of **4q**	71
**4l**	^{1}H-NMR Spectrum of **4l**	50	**4q**	Mass Spectrum of **4q**	72
**4l**	^{13}C-NMR Spectrum of **4l**	51	**4r**	IR Spectrum of **4r**	73
**4l**	Mass Spectrum of **4l**	52	**4r**	^{1}H-NMR Spectrum of **4r**	74
**4m**	IR Spectrum of **4m**	53	**4r**	^{13}C-NMR Spectrum of **4r**	75
**4m**	^{1}H-NMR Spectrum of **4m**	54	**4r**	Mass Spectrum of **4r**	76
**4m**	^{13}C-NMR Spectrum of **4m**	55	**4s**	IR Spectrum of **4s**	77
**4m**	Mass Spectrum of **4m**	56	**4s**	^{1}H-NMR Spectrum of **4s**	78
**4n**	IR Spectrum of **4n**	57	**4s**	^{13}C-NMR Spectrum of **4s**	79
**4n**	^{1}H-NMR Spectrum of **4n**	58	**4s**	Mass Spectrum of **4s**	80
**4n**	^{13}C-NMR Spectrum of **4n**	59	**4t**	IR Spectrum of **4t**	81
**4n**	Mass Spectrum of **4n**	60	**4t**	^{1}H-NMR Spectrum of **4t**	82
**4o**	IR Spectrum of **4o**	61	**4t**	^{13}C-NMR Spectrum of **4t**	83
**4o**	^{1}H-NMR Spectrum of **4o**	62	**4t**	Mass Spectrum of **4t**	84
**4o**	^{13}C-NMR Spectrum of **4o**	63	**4u**	IR Spectrum of **4u**	85
**4o**	Mass Spectrum of **4o**	64	**4u**	^{1}H-NMR Spectrum of **4u**	86
**4p**	IR Spectrum of **4p**	65	**4u**	^{13}C-NMR Spectrum of **4u**	87
**4p**	^{1}H-NMR Spectrum of **4p**	66	**4u**	Mass Spectrum of **4u**	88
**4p**	^{13}C-NMR Spectrum of **4p**	67	**4v**	IR Spectrum of **4v**	89
**4p**	Mass Spectrum of **4p**	68	**4v**	^{1}H-NMR Spectrum of **4v**	90
**4q**	IR Spectrum of **4q**	69	**4v**	^{13}C-NMR Spectrum of **4v**	91
**4q**	^{1}H-NMR Spectrum of **4q**	70	**4v**	Mass Spectrum of **4v**	92

Entry	Content	Page no.
**Table S1**	Binding affinity, interacting residues and types of interaction mediated by compound **4q** and Erlotinib with EGFR	93
**Table S2**	Binding affinity, interacting residues and types of interaction mediated by compound **4q** and Erlotinib with HER2	94
**Table S3**	Binding affinity, interacting residues and types of interaction mediated by compound **4q** and Sorafenib with VEGFR2	95
**Table S4**	Binding affinity, interacting residues and types of interaction mediated by compound **4q** and Dinaciclib with CDK2	96
**Table S5**	Binding affinity, interacting residues and types of interaction mediated by compound **4q** and Estrogen (Estradiol) with Estrogen receptor (1A52)	97
**Table S6**	Binding affinity, interacting residues and types of interaction mediated by compound **4q** and Tamoxifen with Estrogen receptor (3ERT)	98
**Figure S1**	Intermolecular interaction between (A) Erlotinib with EGFR (B) Erlotinib with HER2 (C) Dinaciclib with CDK2 (D) Sorafenib with VEGFR2	99
**Figure S2**	Alignment of (A) compound 4q (yellow) and dibaciclib (blue) in the active site of CDK2; (B) compound 4q (green) and erlotinib (blue) in the active site of EGFR	100
**Figure S3**	(A) compound **4q** (green) and erlotinib (blue) in the active site of EGFR. (B) compound **4q** (red) and sorafenib (blue) in the active site of VEGFR2	101
**Figure S4**	Alignment of (A) compound 4q (red) and estradiol (blue) in the active site of Estrogen receptor (PDB id= 1a52) ; (B) compound 4q (green) and tamoxifen (red) in the active site of Estrogen receptor (PDB id = 3ERT). Red dotted circle = Active site	102
**Figure S5-S21**	IC_{50} of compound **4a**, **4b**, **4q** and **4v** with various enzymes	103-123
**Figure S22-S25**	Compound **4a**, **4b**, **4q**, **4v** and letrozole with Aromatase	124-127

IR Spectrum of **4a**

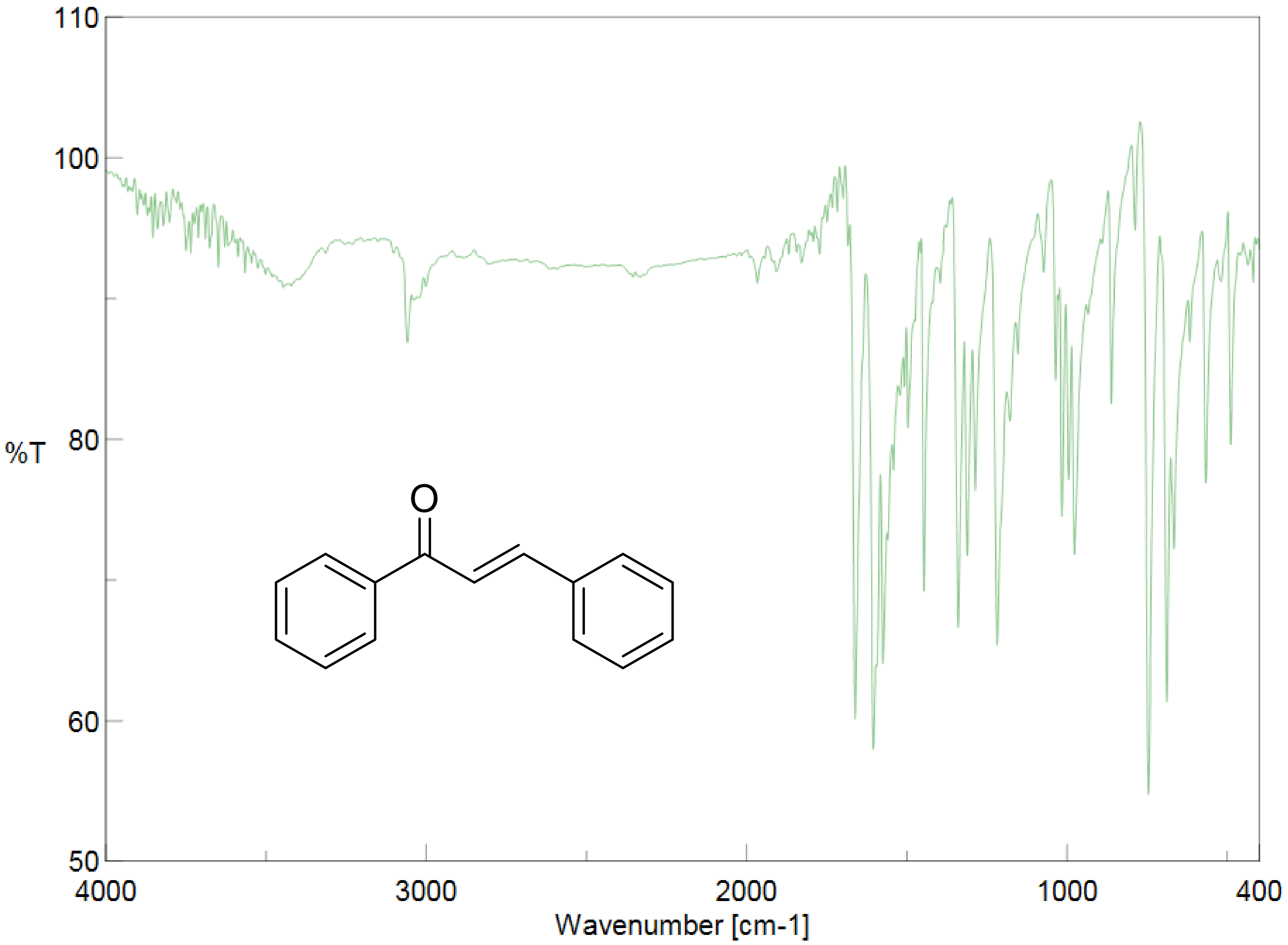

^{1}H-NMR Spectrum of **4a**

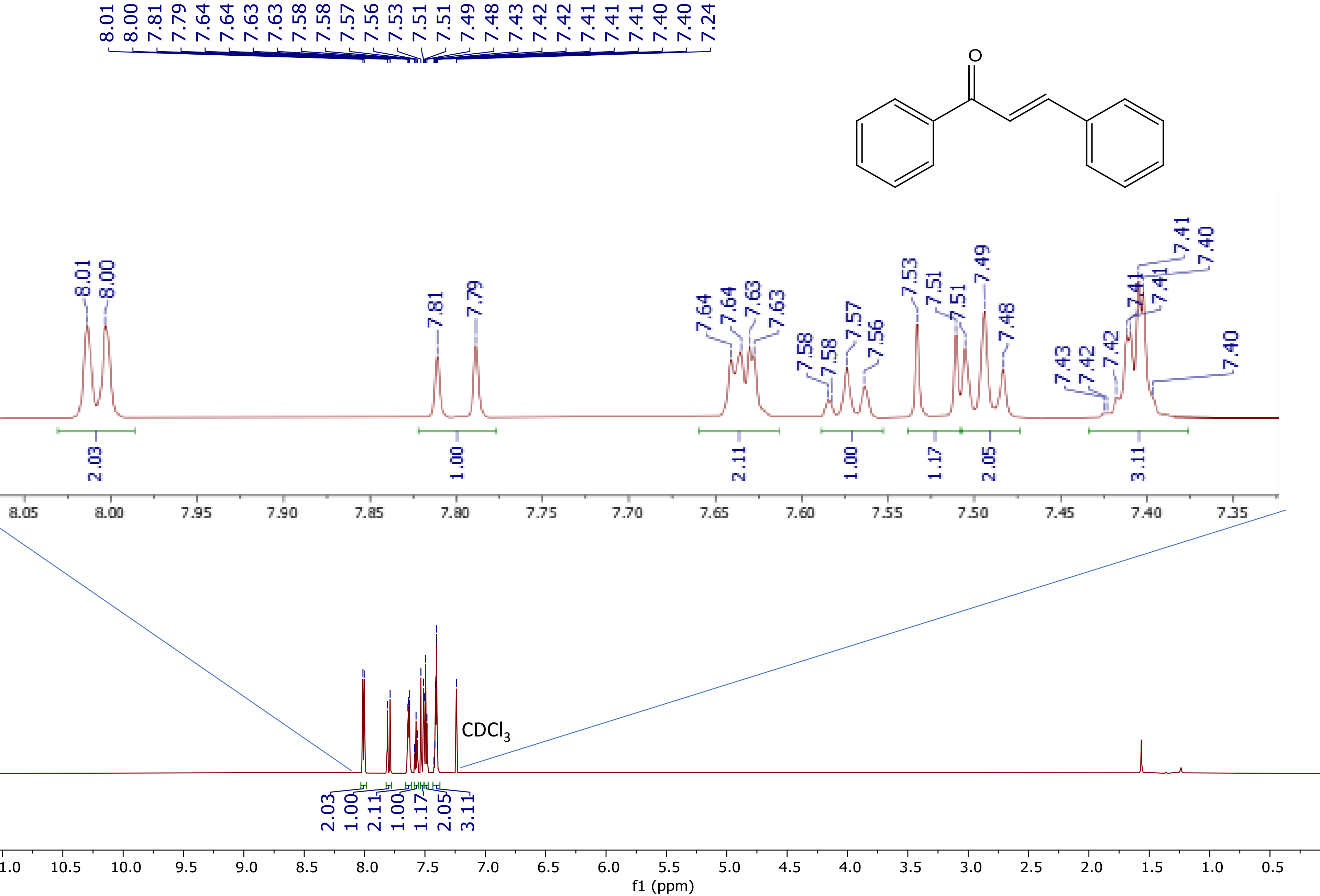

^{13}C-NMR Spectrum of **4a**

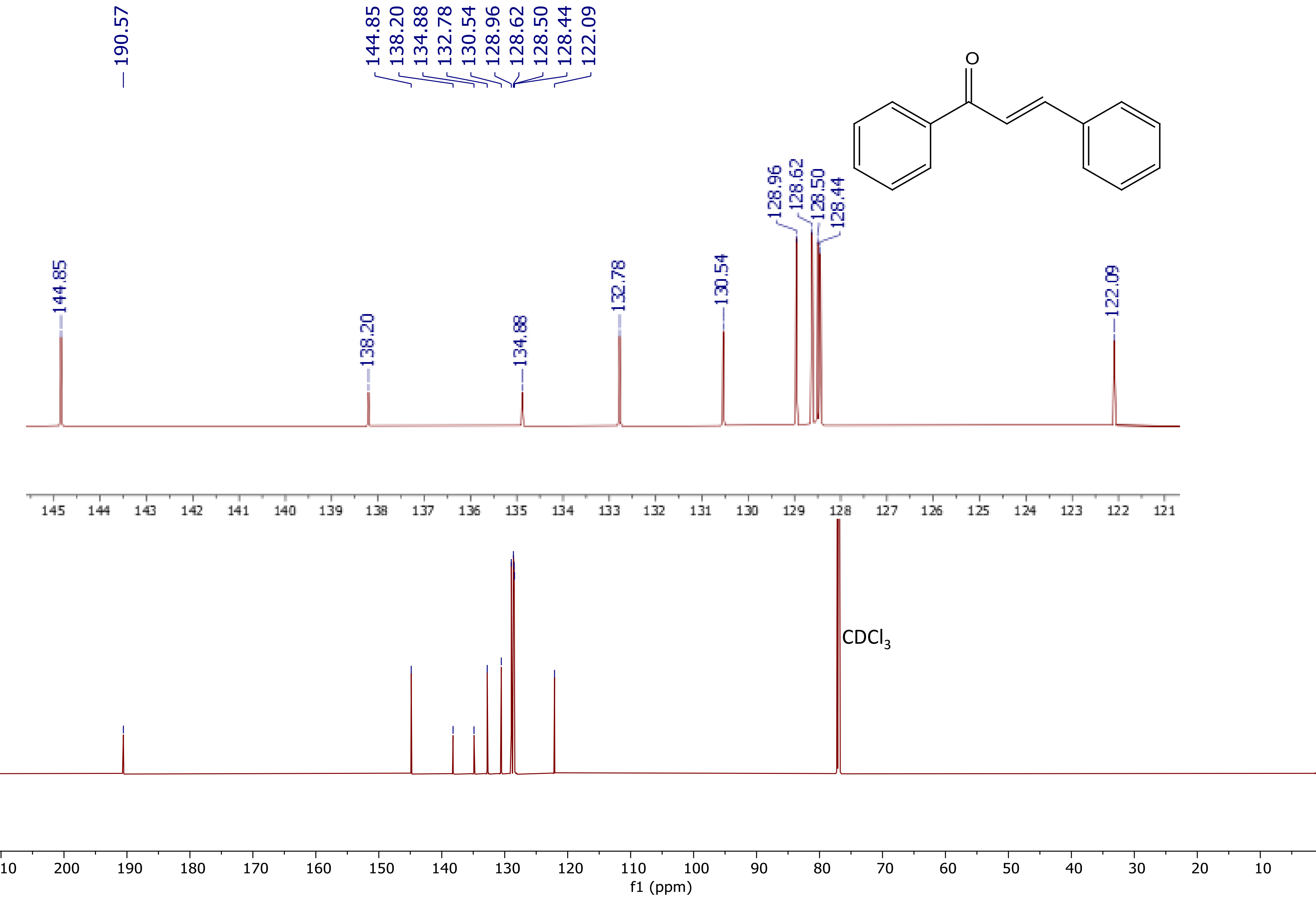

Mass Spectrum of **4a**

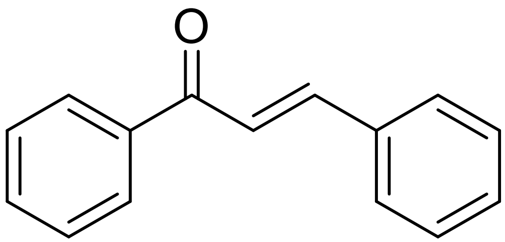

Chemical Formula: $C_{15}H_{12}O$

Exact Mass: 208.09

m/z: 209 $[M+H]^+$

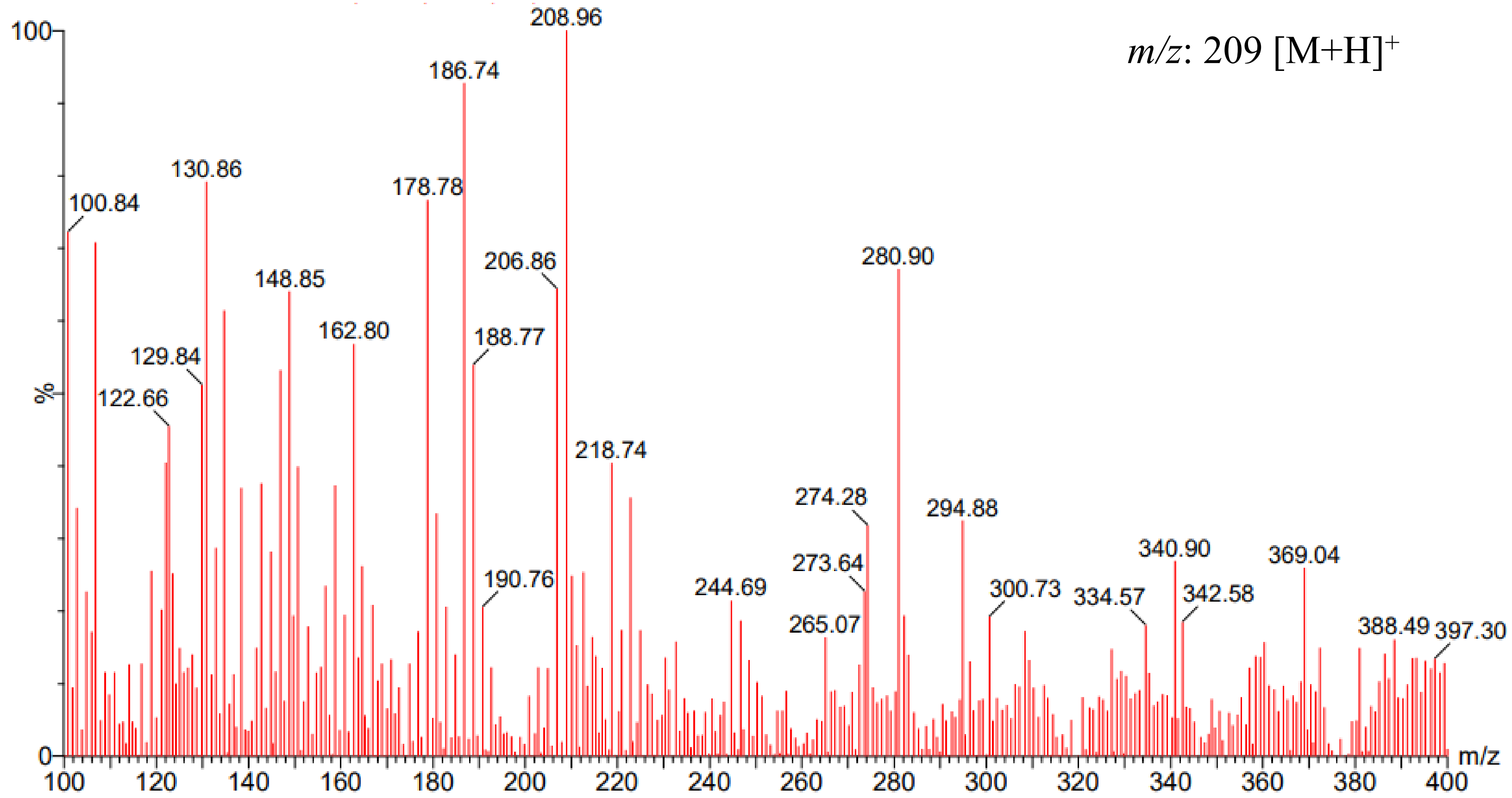

IR Spectrum of **4b**

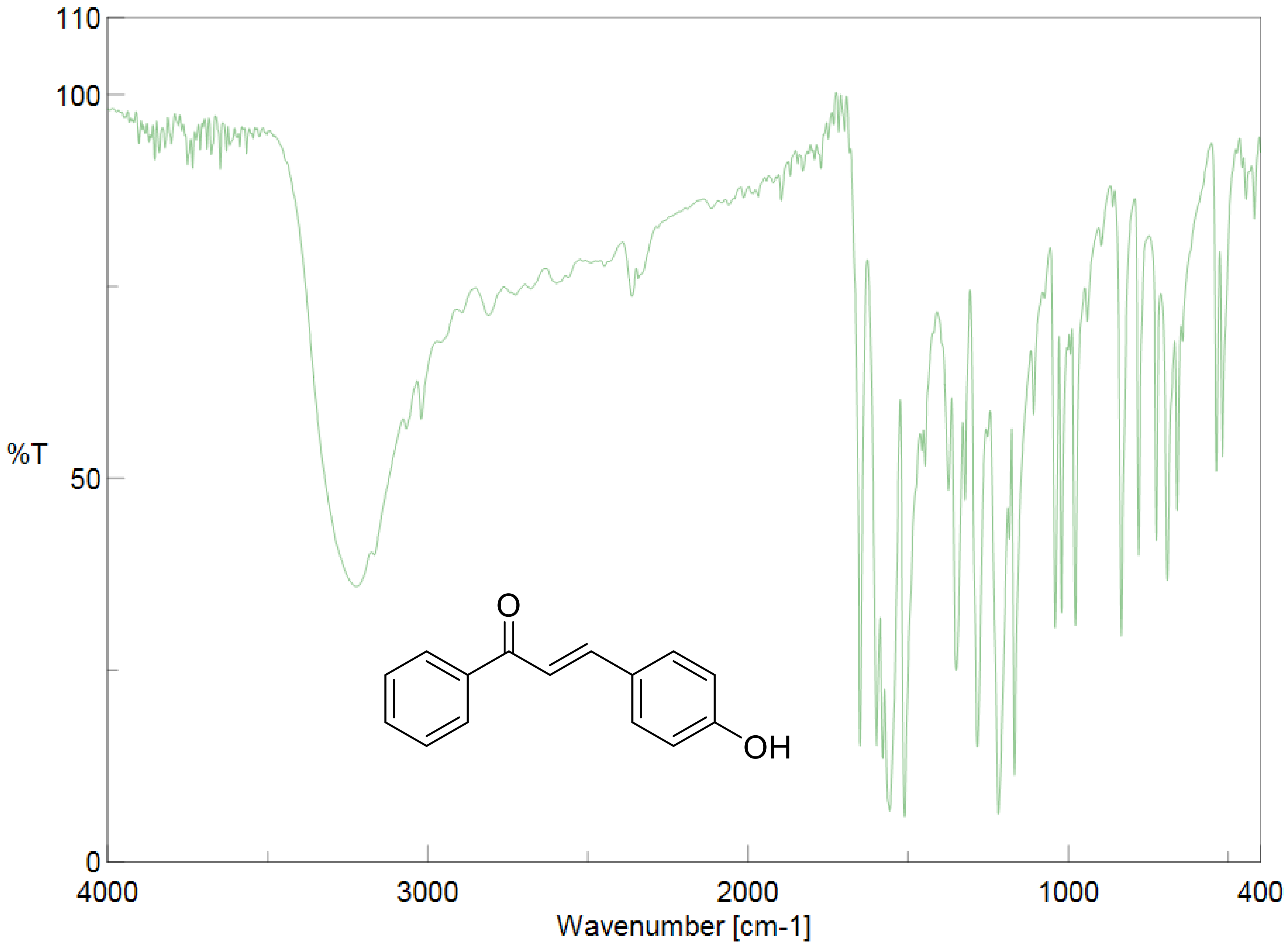

^{1}H-NMR Spectrum of **4b**

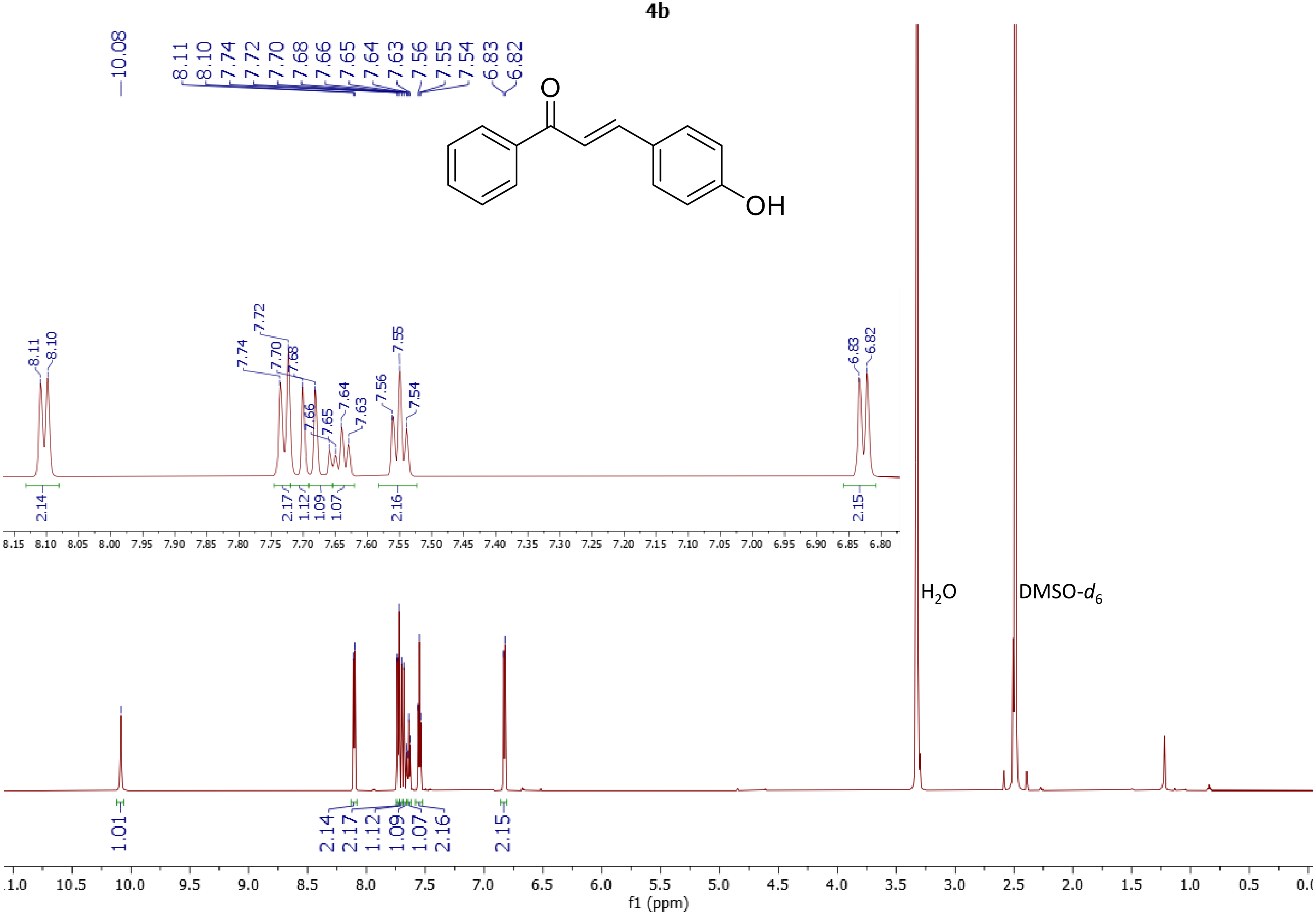

^{13}C-NMR Spectrum of **4b**

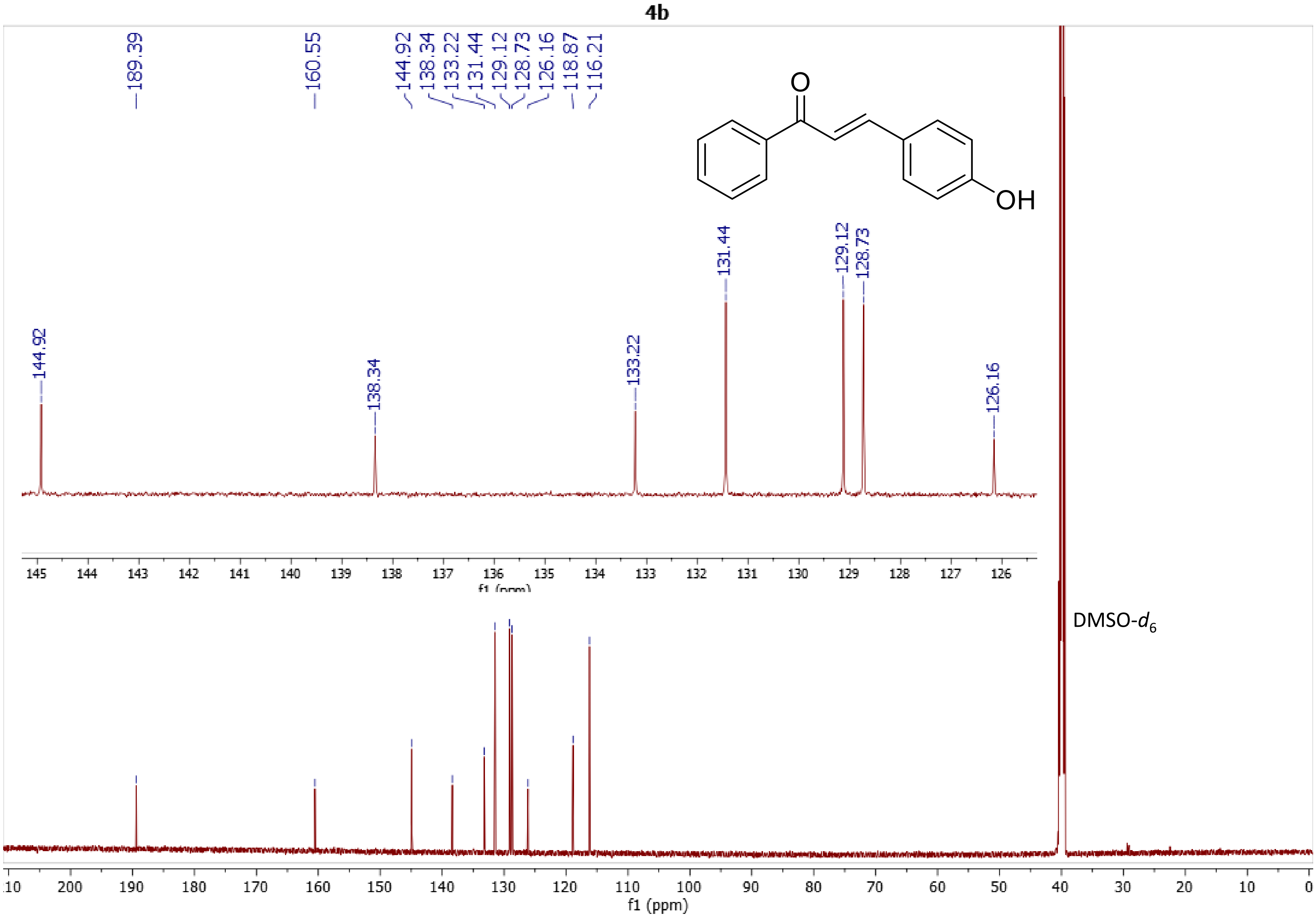

Mass Spectrum of **4b**

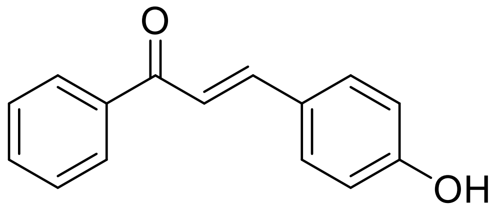

Chemical Formula: $C_{15}H_{12}O_2$

Exact Mass: 224.08

m/z: 225 $[M+H]^+$, 246.96 $[M+Na]^+$

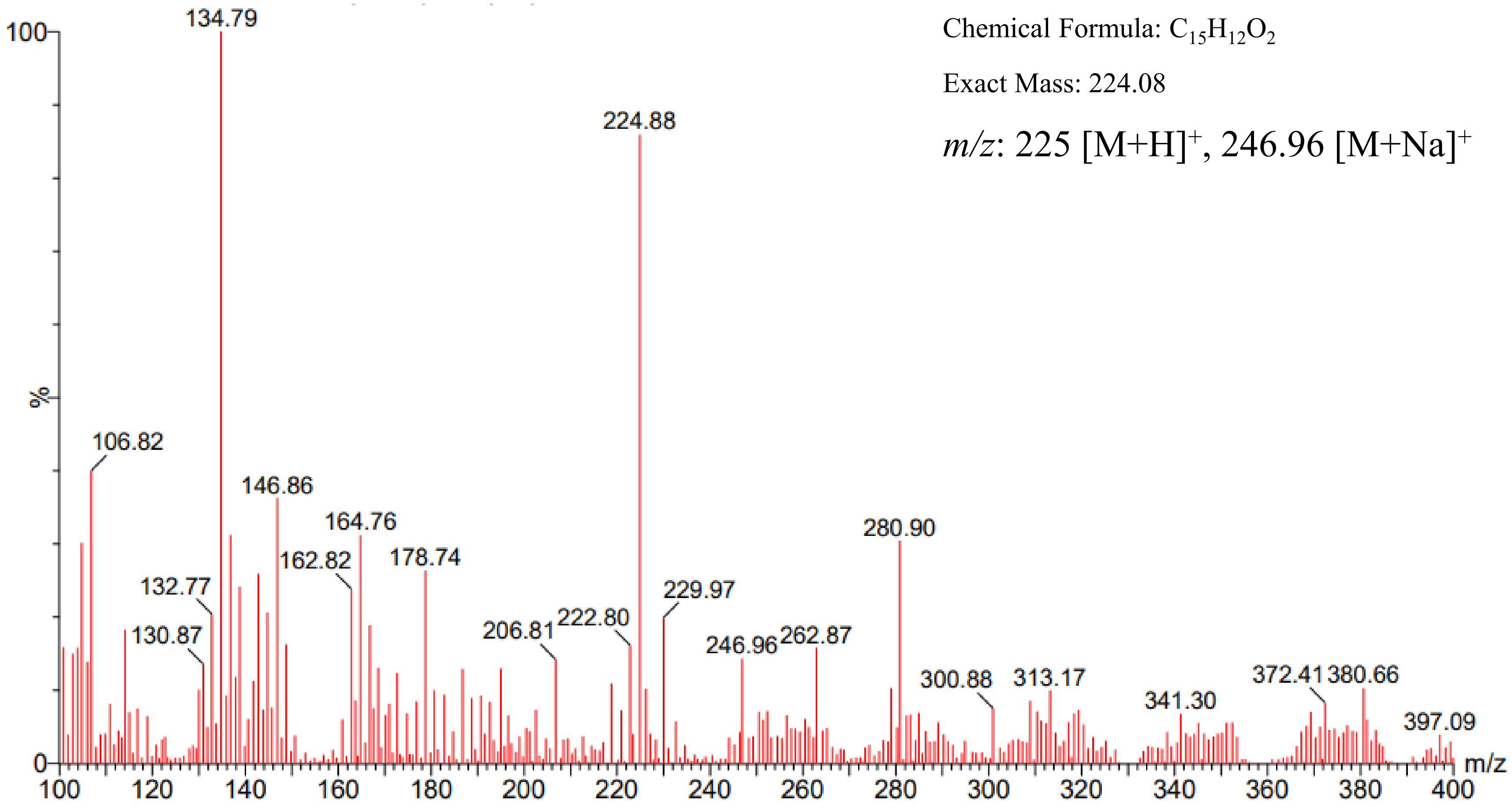

IR Spectrum of **4c**

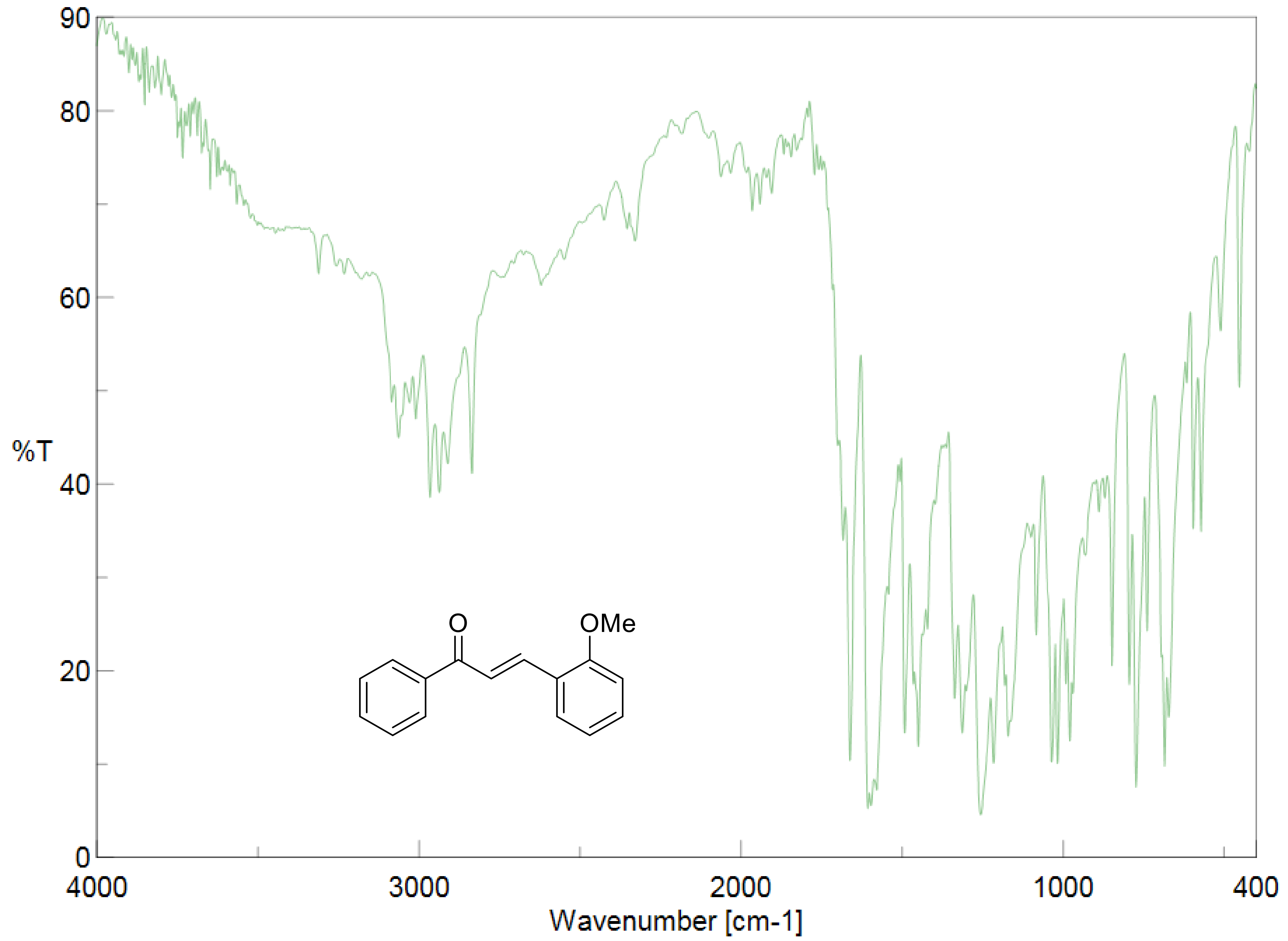

^{1}H-NMR Spectrum of **4c**

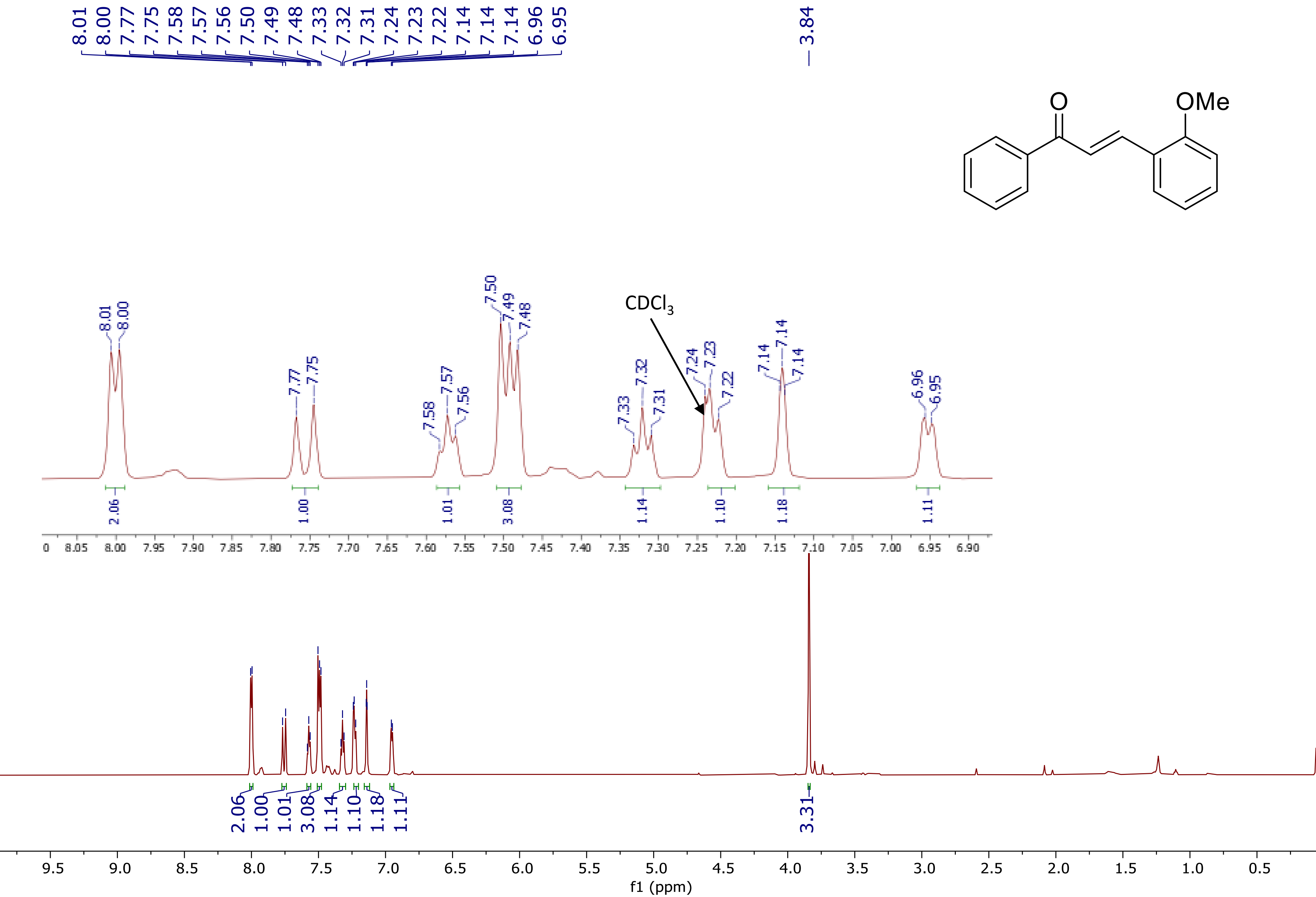

^{13}C-NMR Spectrum of **4c**

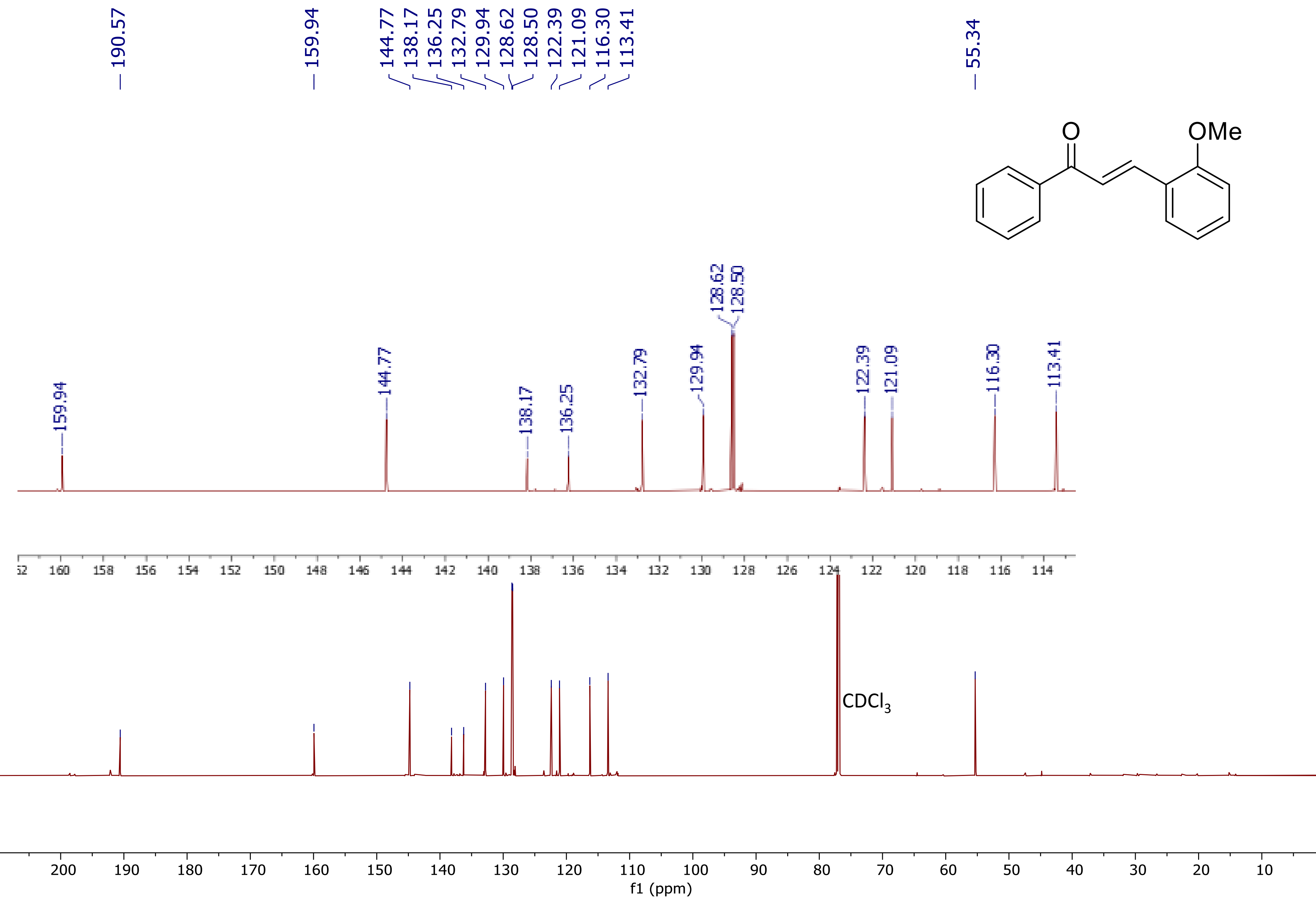

Mass Spectrum of **4c**

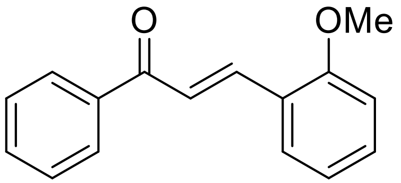

Chemical Formula: $C_{16}H_{14}O_2$

Exact Mass: 238.10

m/z: 239.1 $[M+H]^+$, 261.1 $[M+Na]^+$

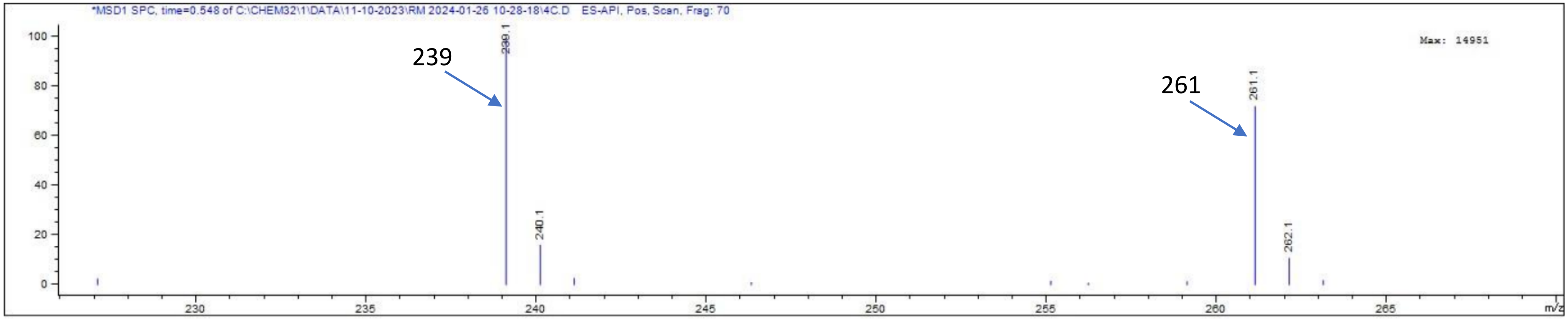

IR Spectrum of **4d**

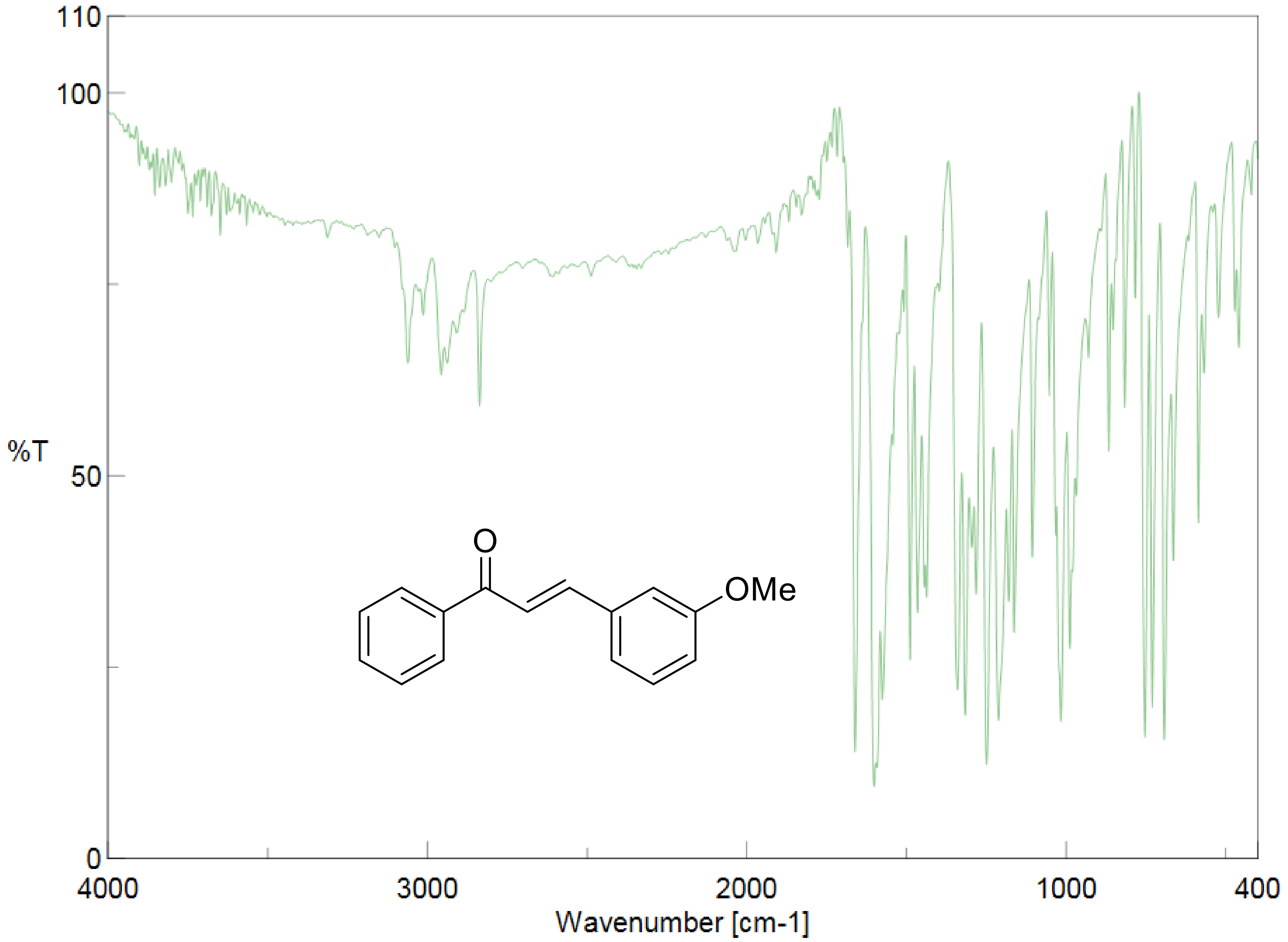

^{1}H-NMR Spectrum of **4d**

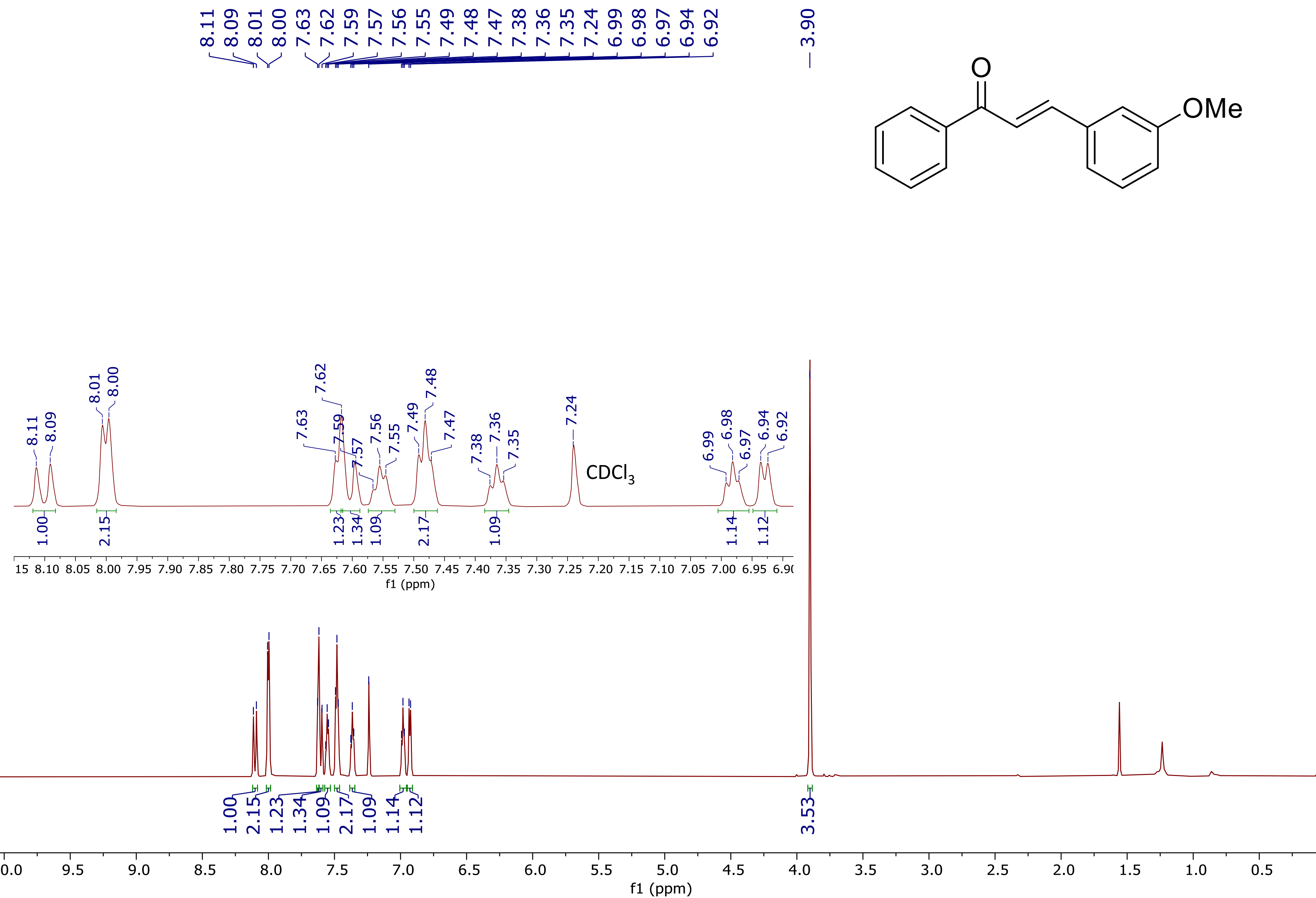

^{13}C-NMR Spectrum of **4d**

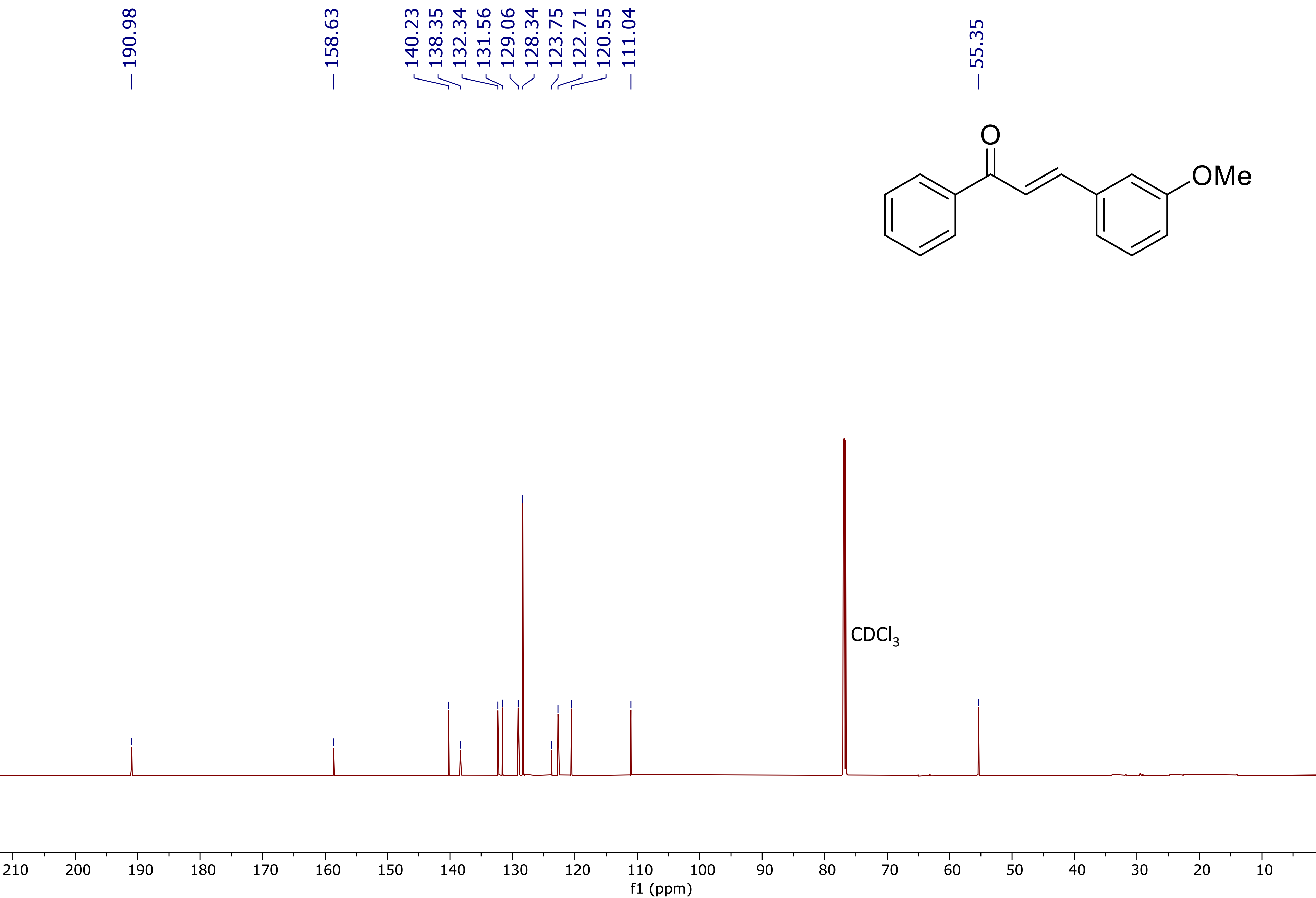

Mass Spectrum of **4d**

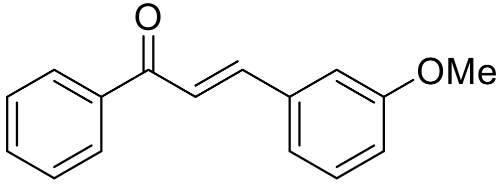

Chemical Formula: $C_{16}H_{14}O_2$

Exact Mass: 238.10

m/z: 238.80 $[M+H]^+$, 260.8 $[M+Na]^+$

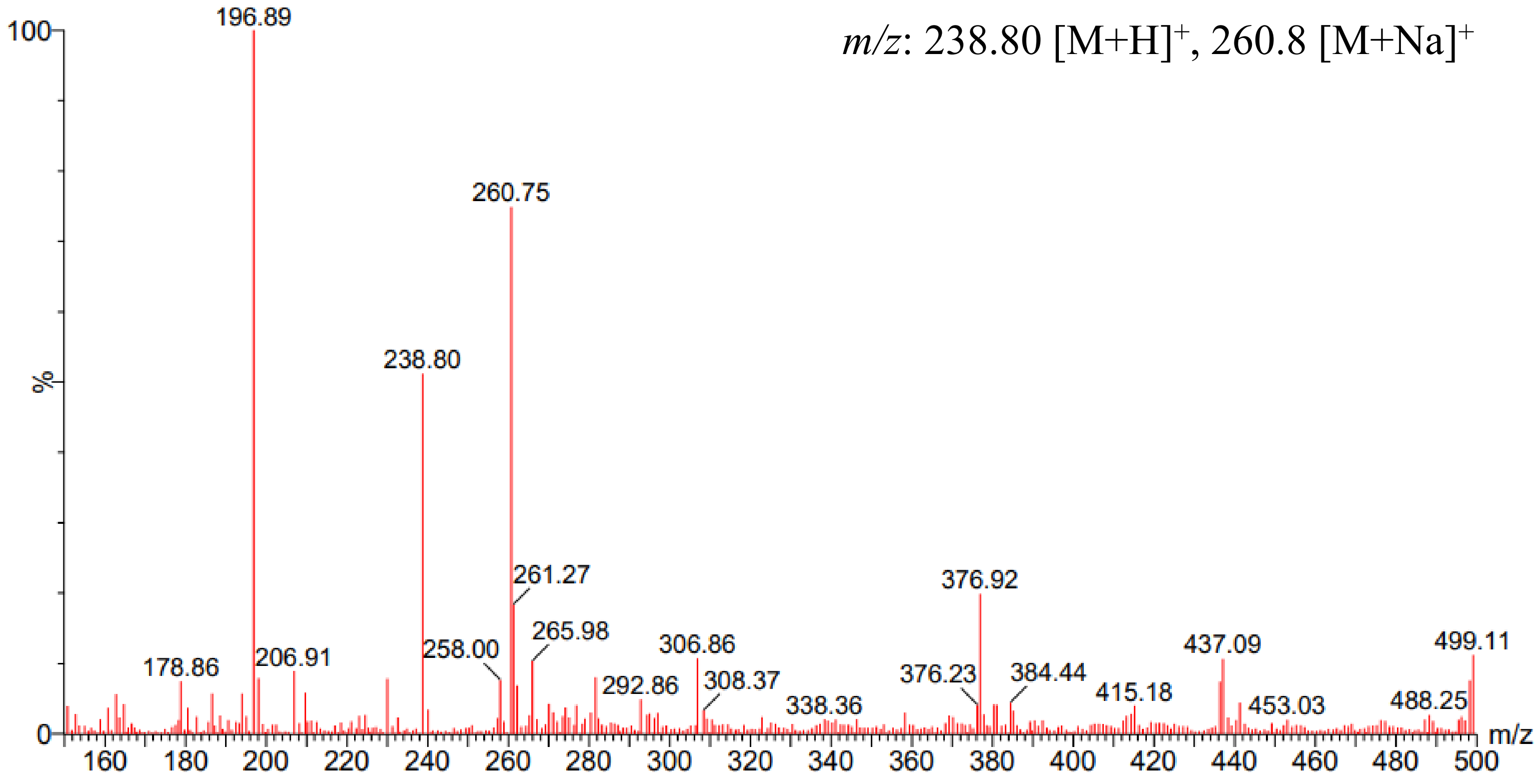

IR Spectrum of **4e**

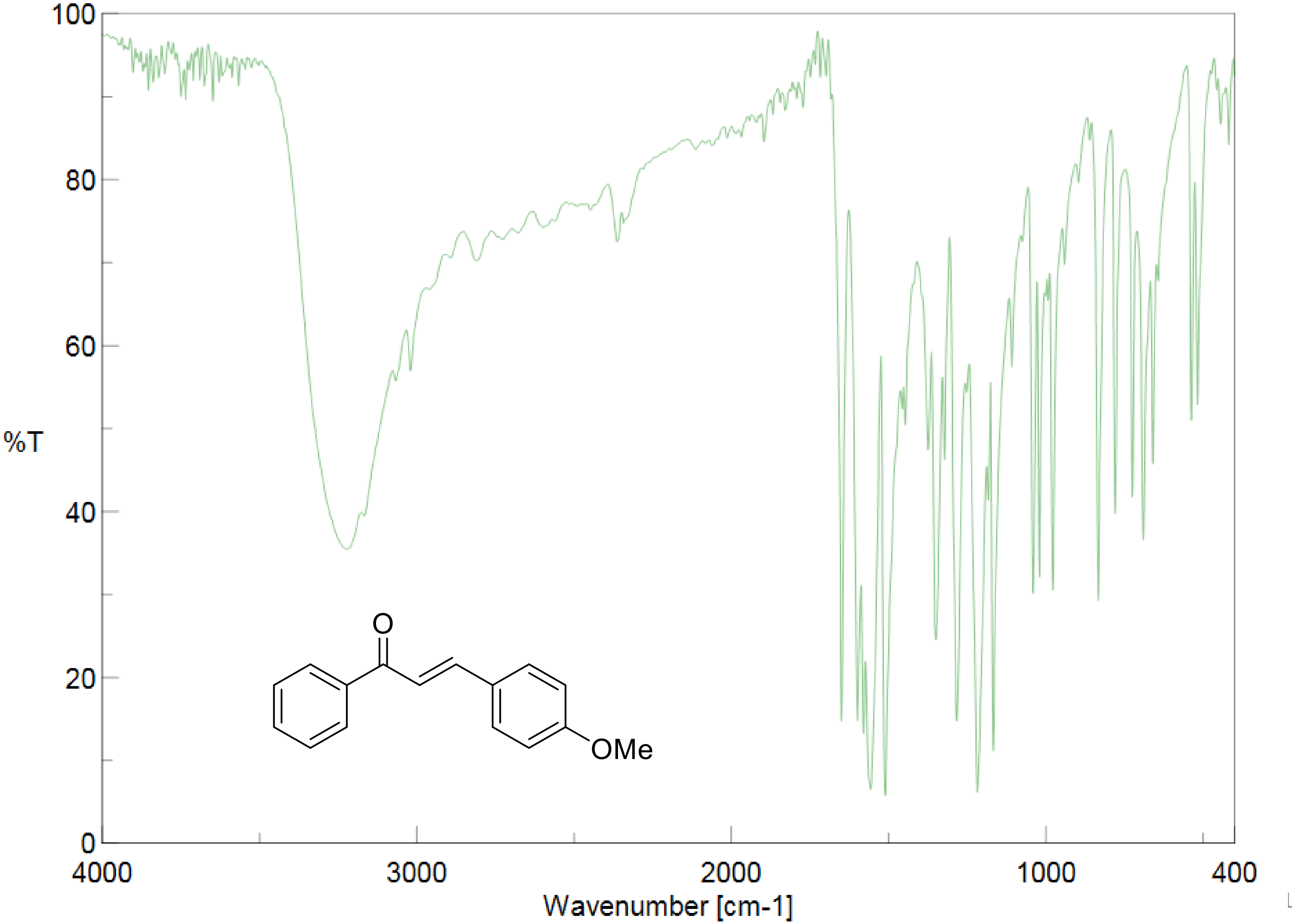

^{1}H-NMR Spectrum of **4e**

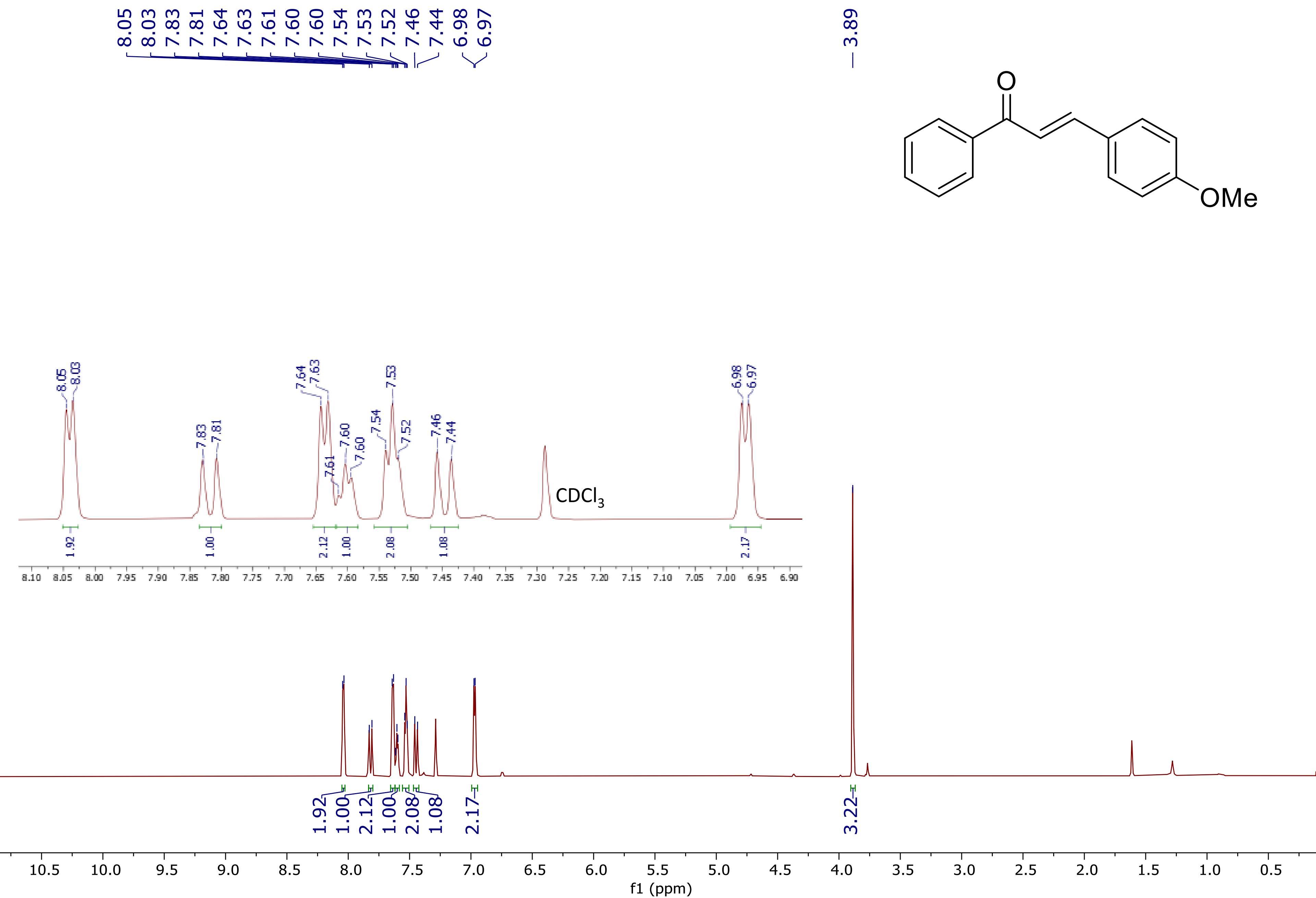

^{13}C-NMR Spectrum of **4e**

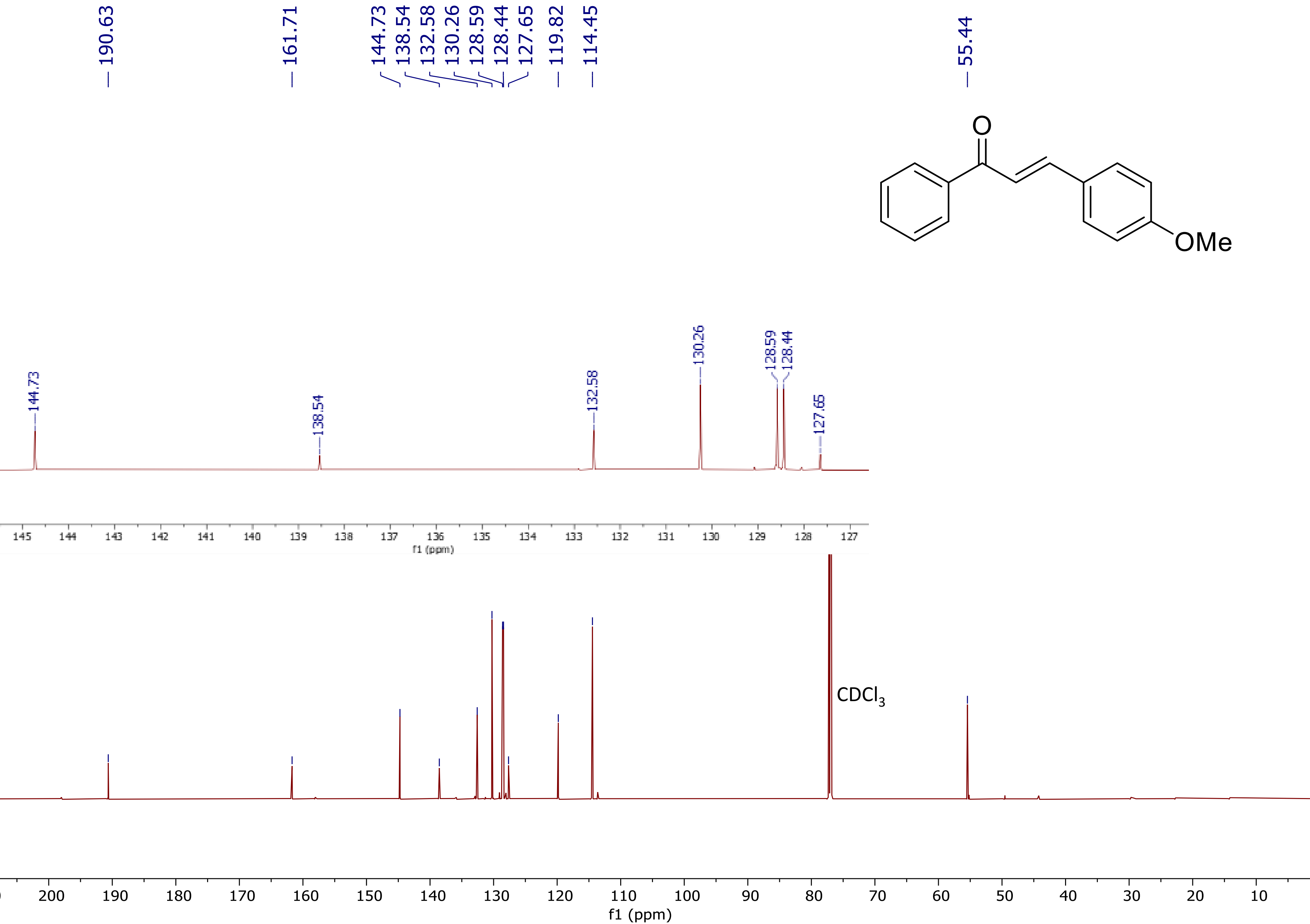

Mass Spectrum of **4e**

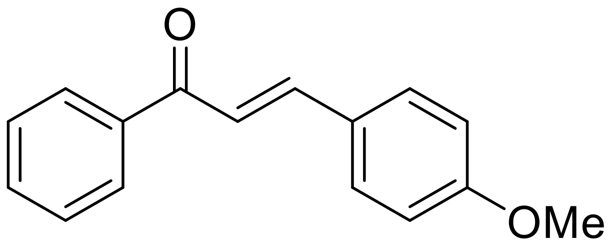

Chemical Formula: $C_{16}H_{14}O_2$

Exact Mass: 238.10

m/z: 239 $[M+H]^+$; 261 $[M+H]^+$

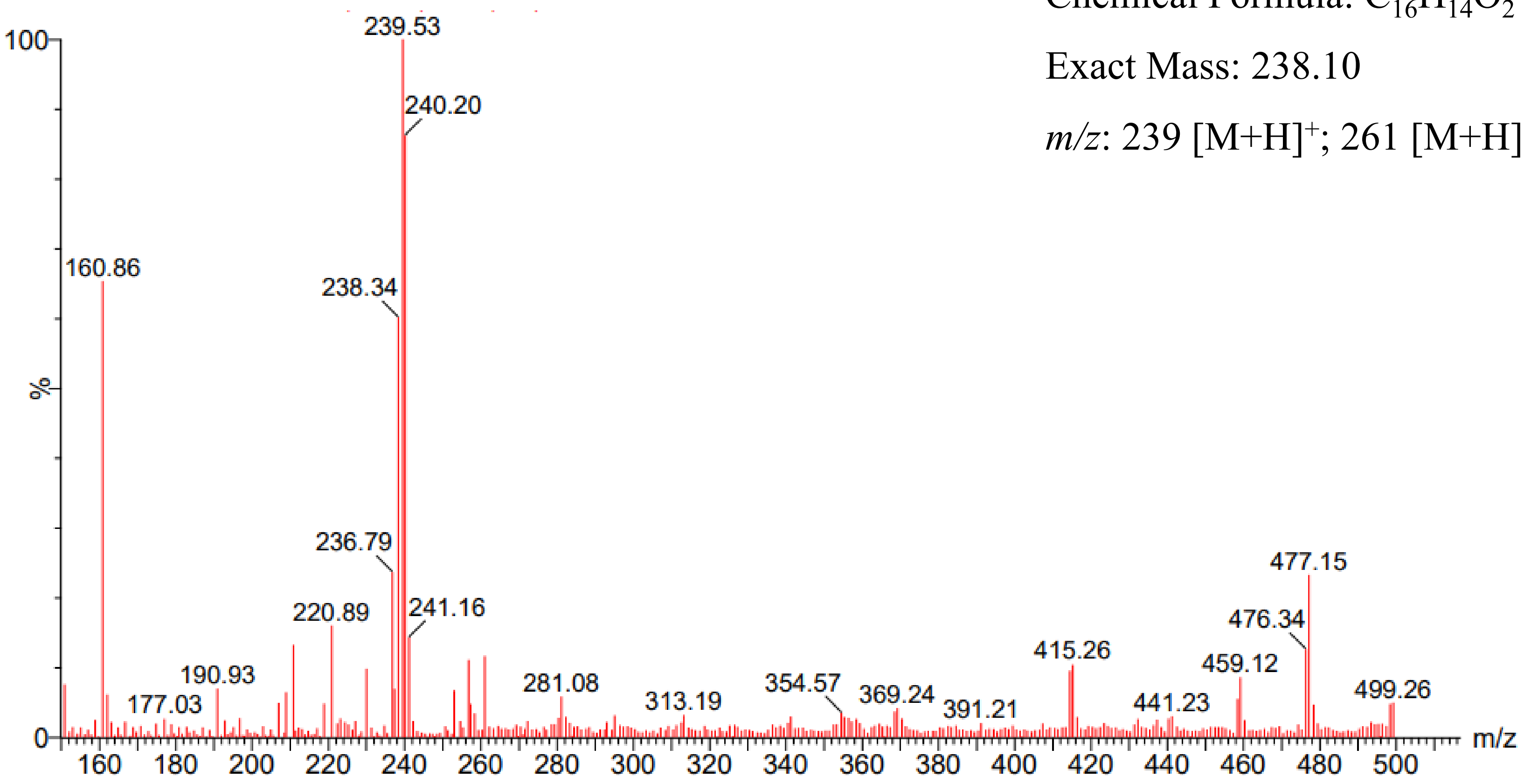

IR Spectrum of **4f**

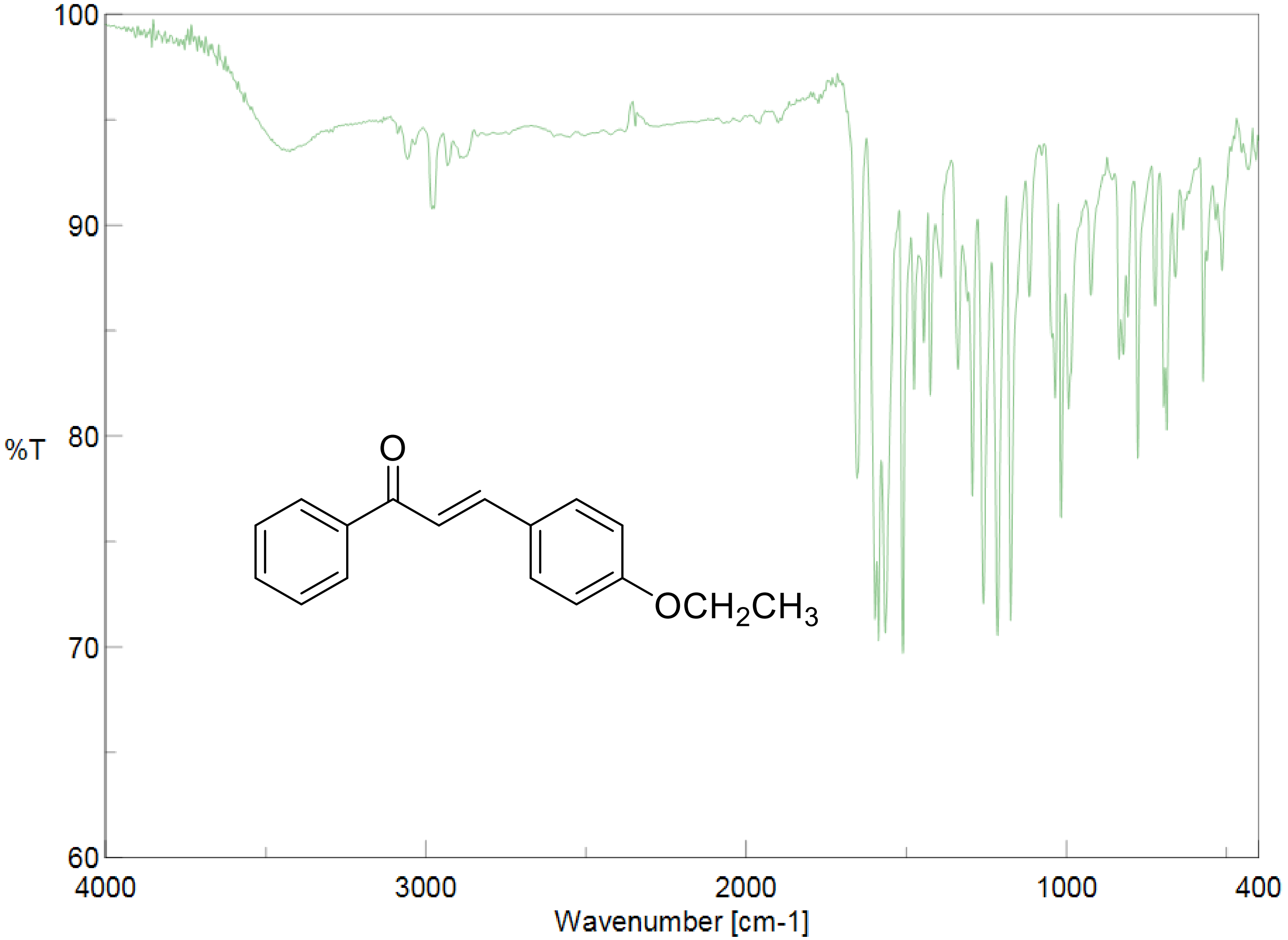

^{1}H-NMR Spectrum of **4f**

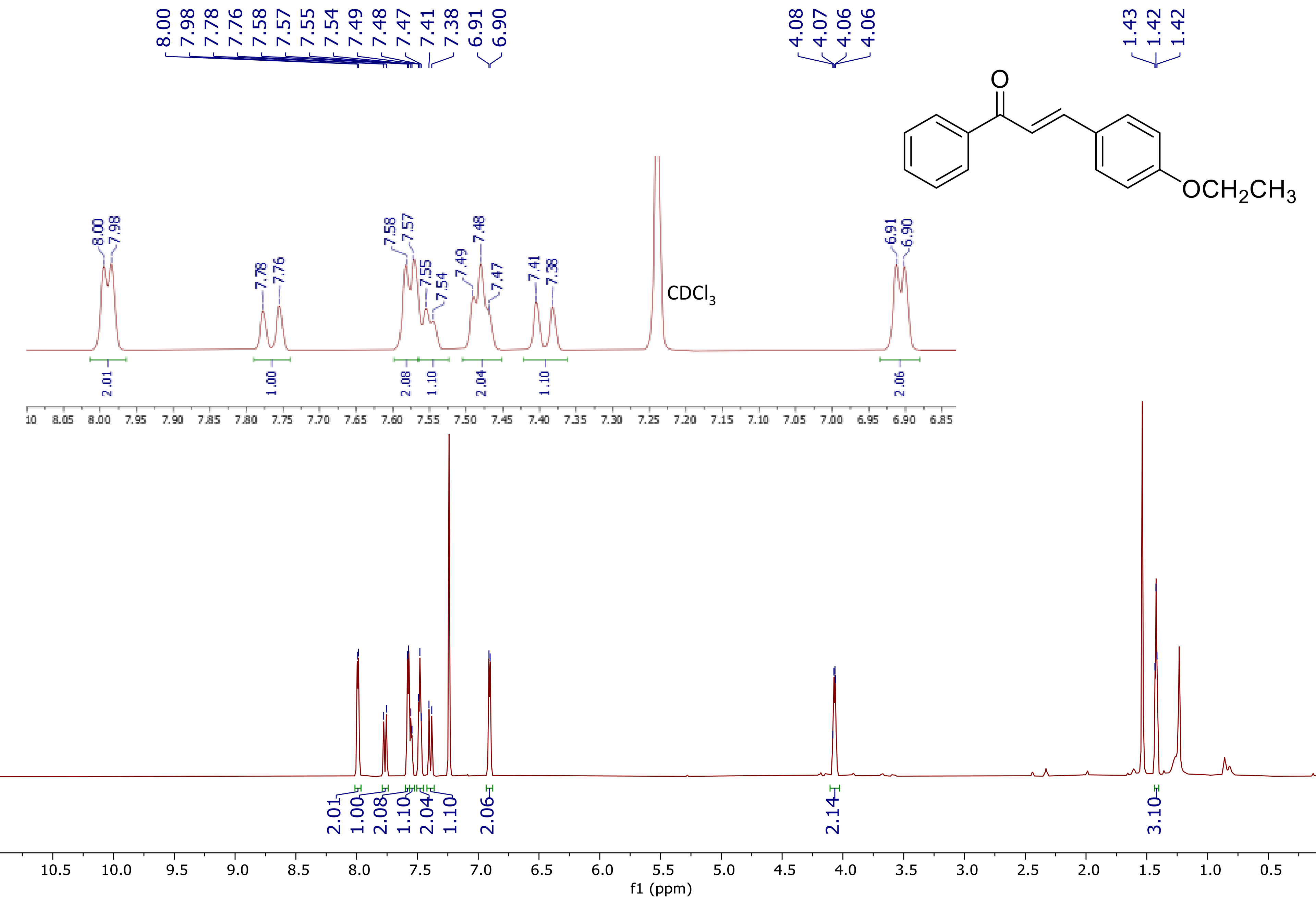

^{13}C-NMR Spectrum of **4f**

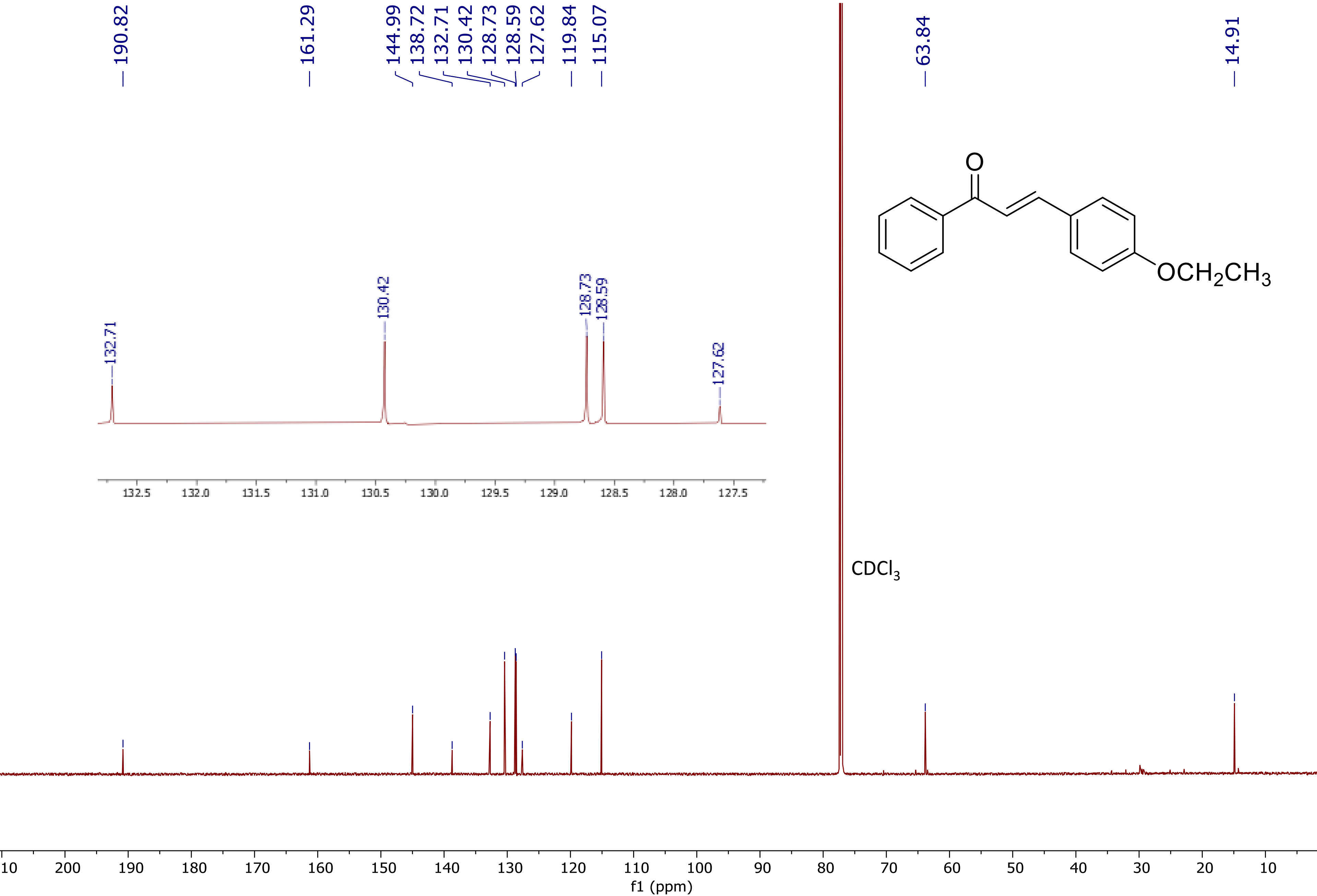

Mass Spectrum of **4f**

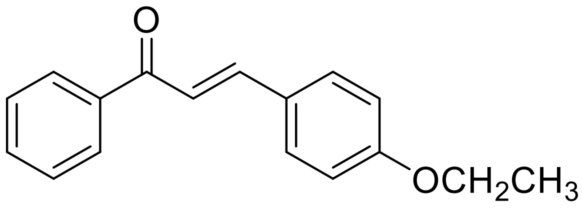

Chemical Formula: $C_{17}H_{16}O_2$

Exact Mass: 252.12

m/z: 253 $[M+H]^+$, 275 $[M+Na]^+$

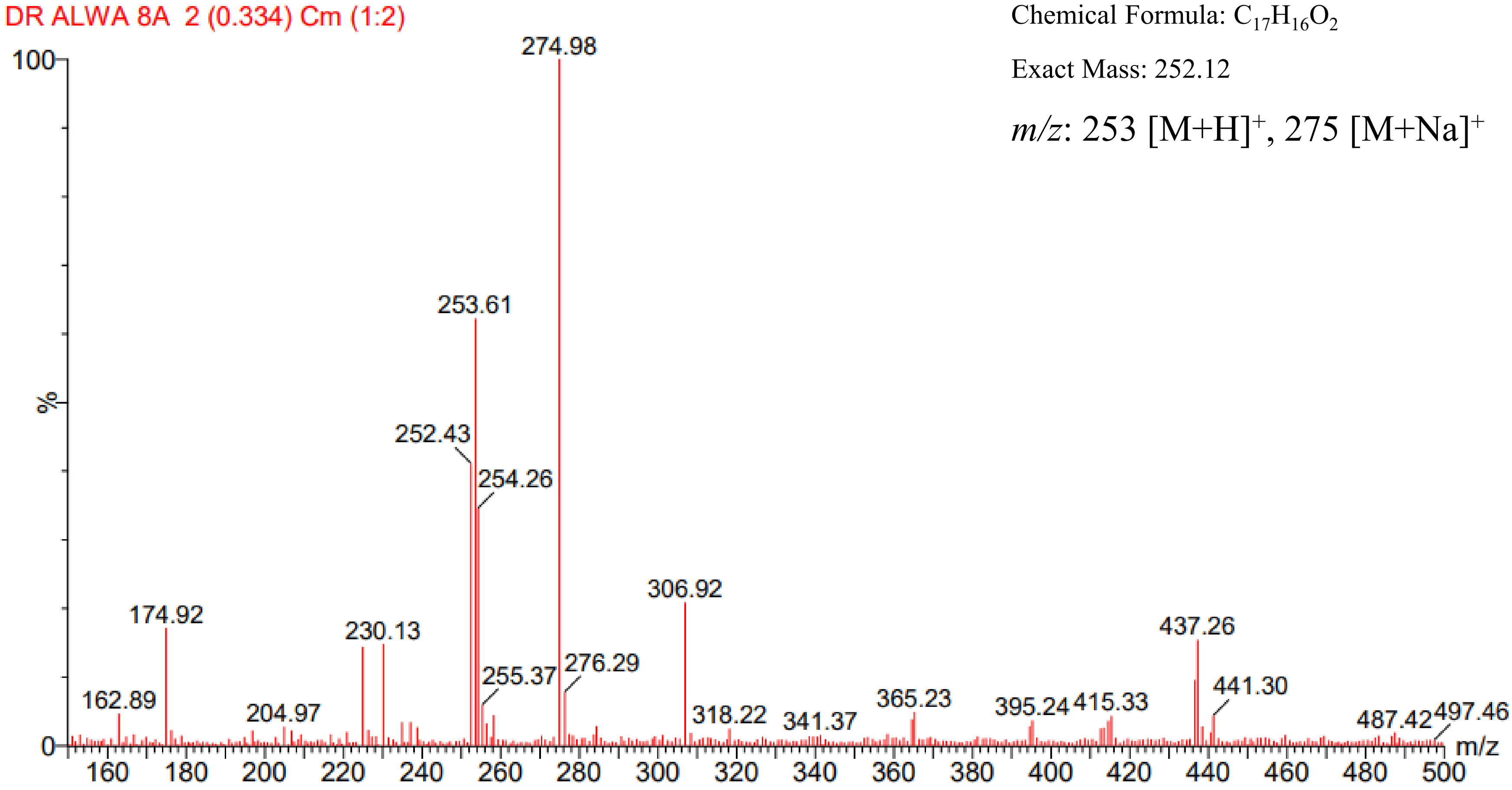

IR Spectrum of **4g**

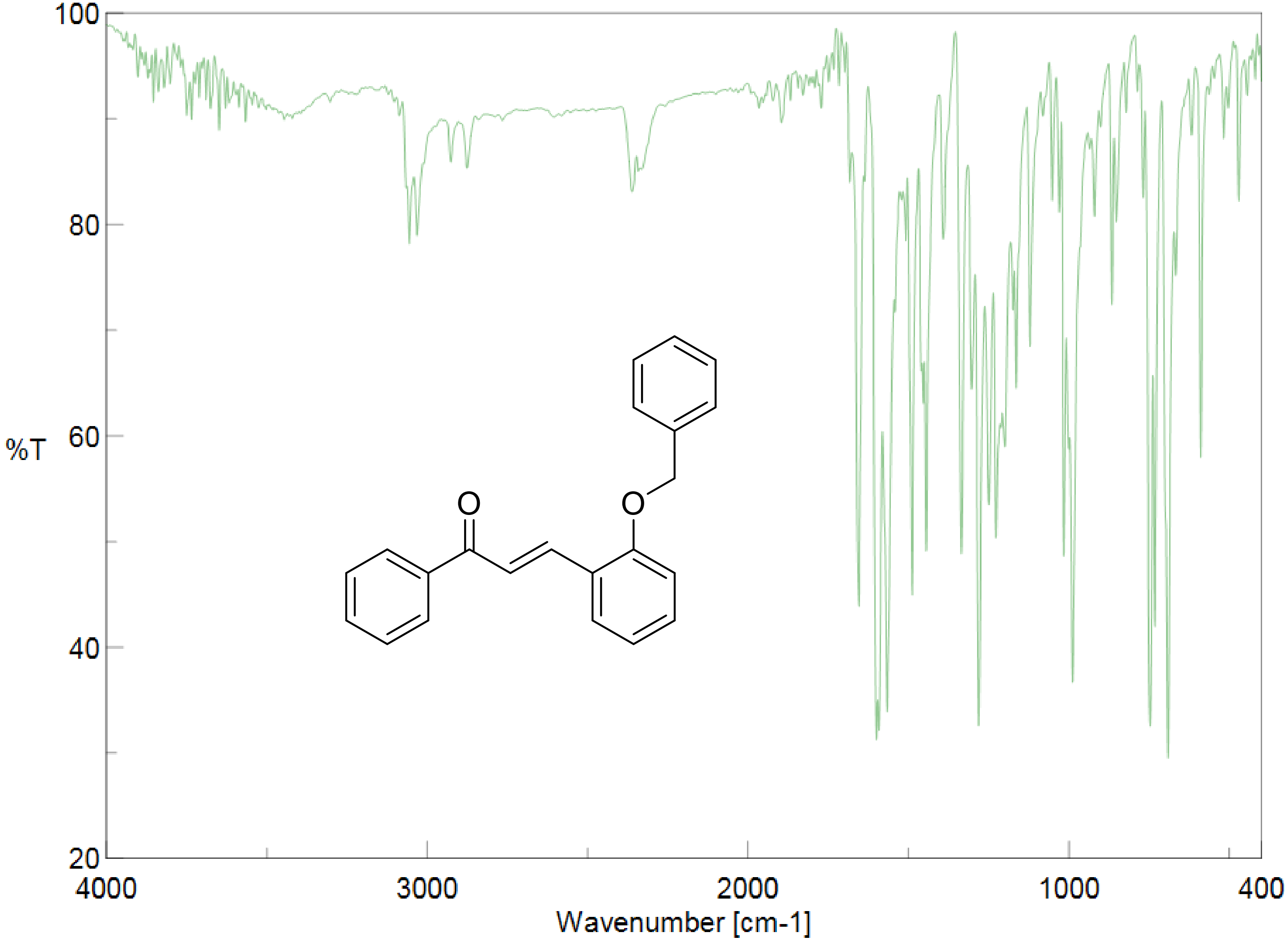

^{1}H-NMR Spectrum of **4g**

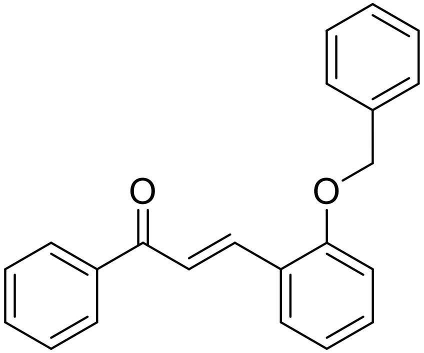

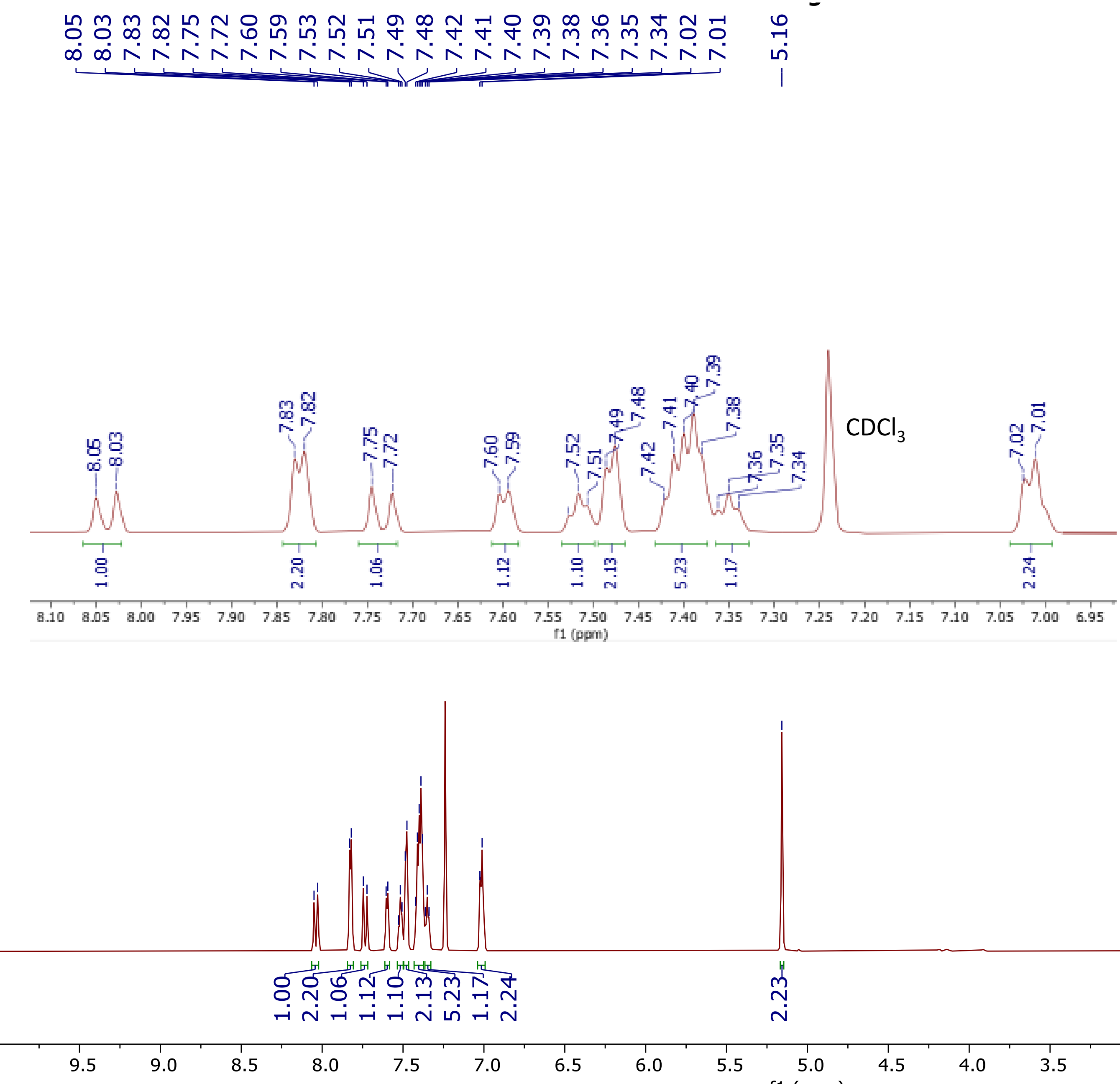

^{13}C-NMR Spectrum of **4g**

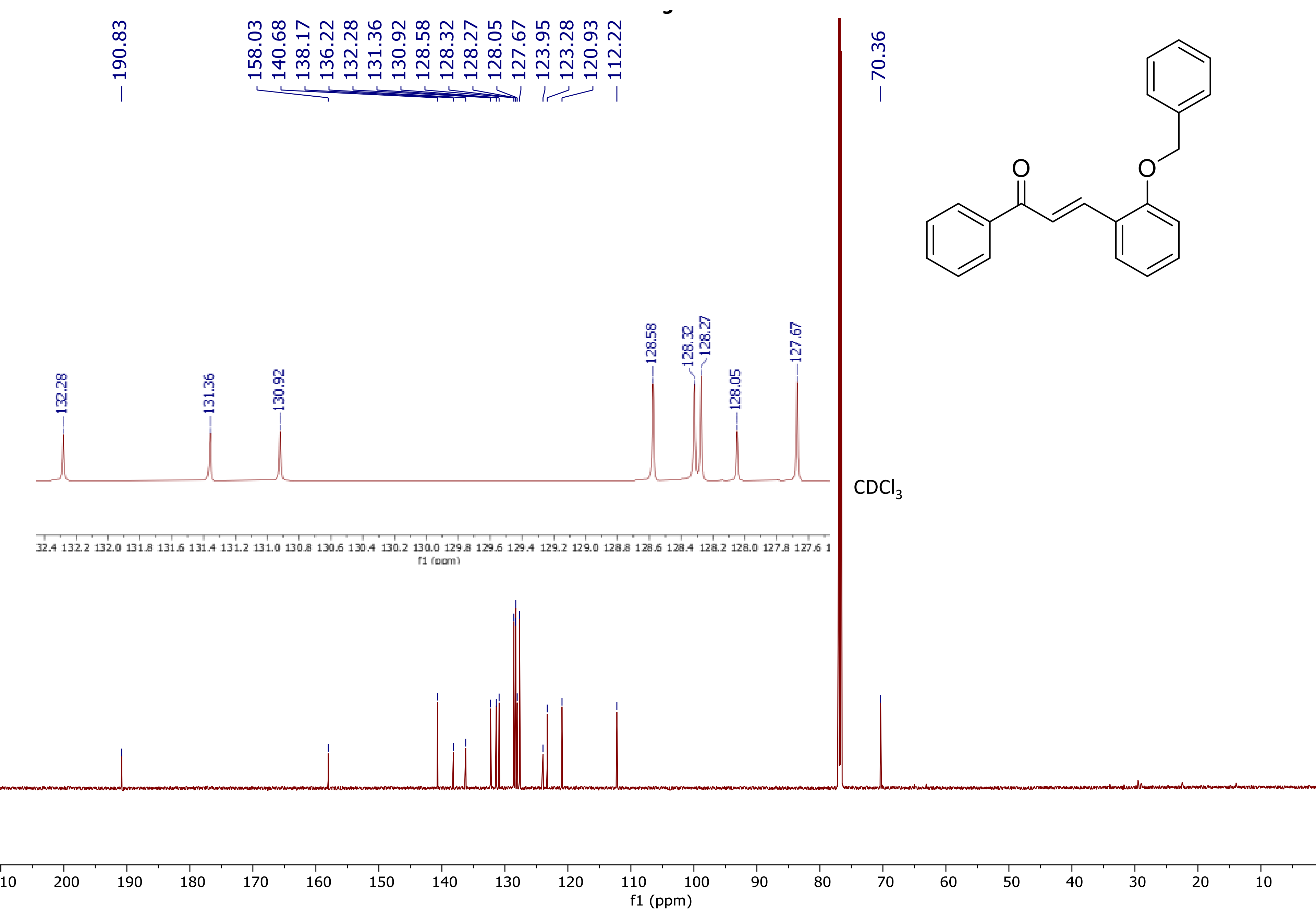

Mass Spectrum of **4g**

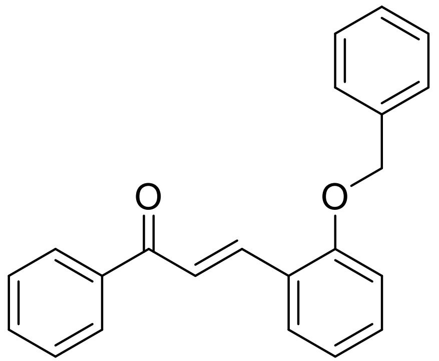

Chemical Formula: $C_{22}H_{18}O_2$

Exact Mass: 314.13

m/z: 315 $[M+H]^+$, 337 $[M+Na]^+$

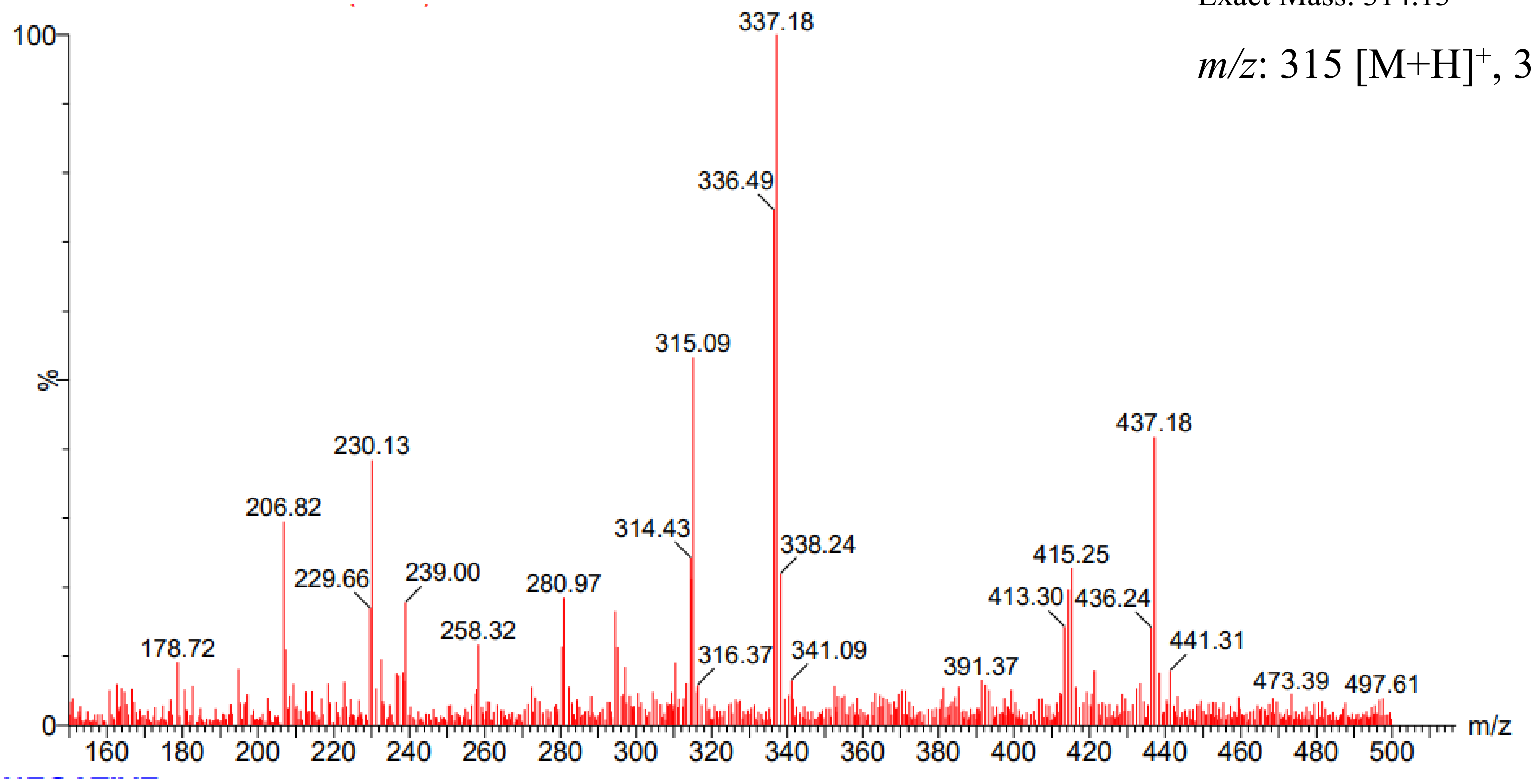

IR Spectrum of **4h**

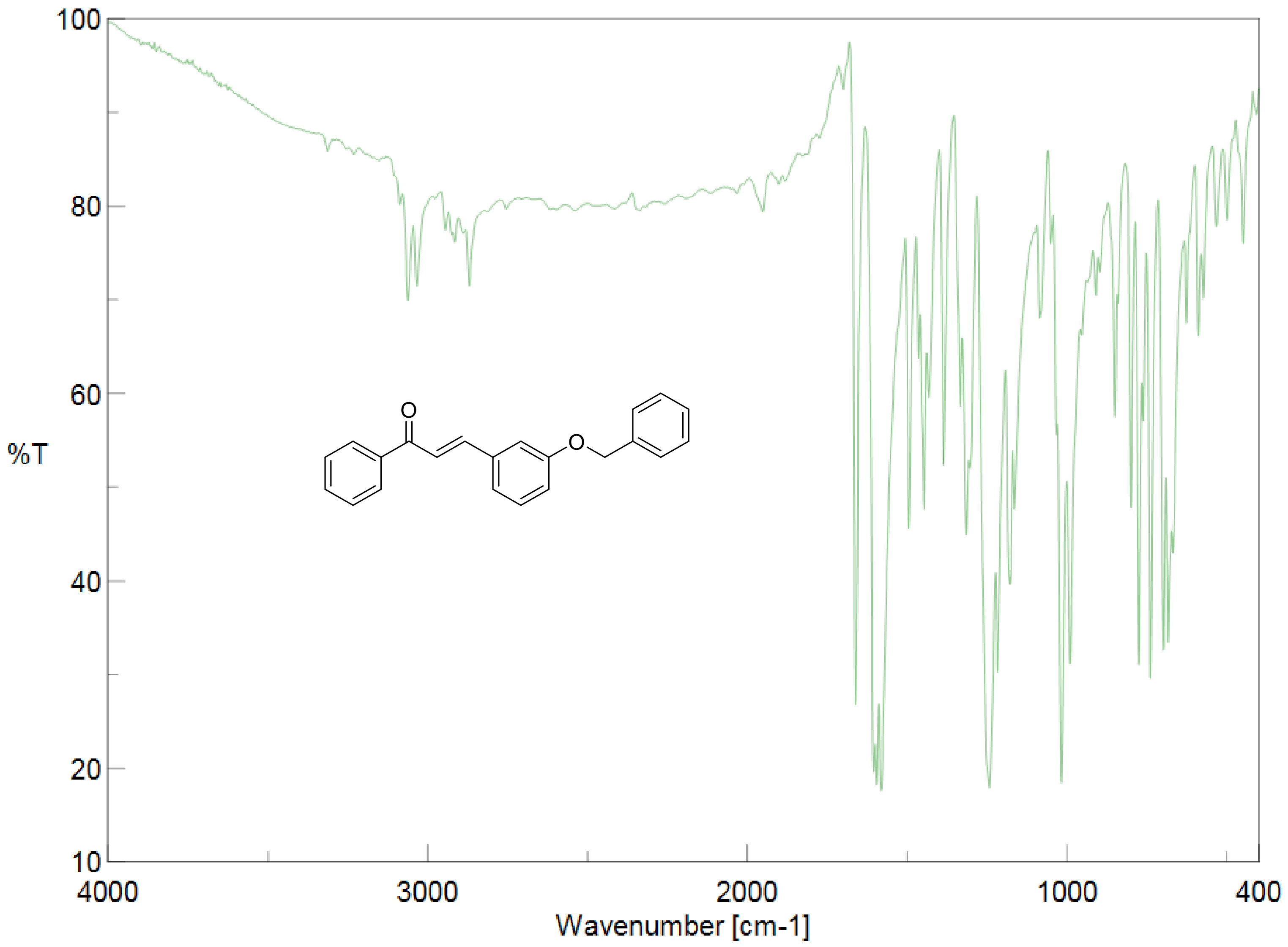

^{1}H-NMR Spectrum of **4h**

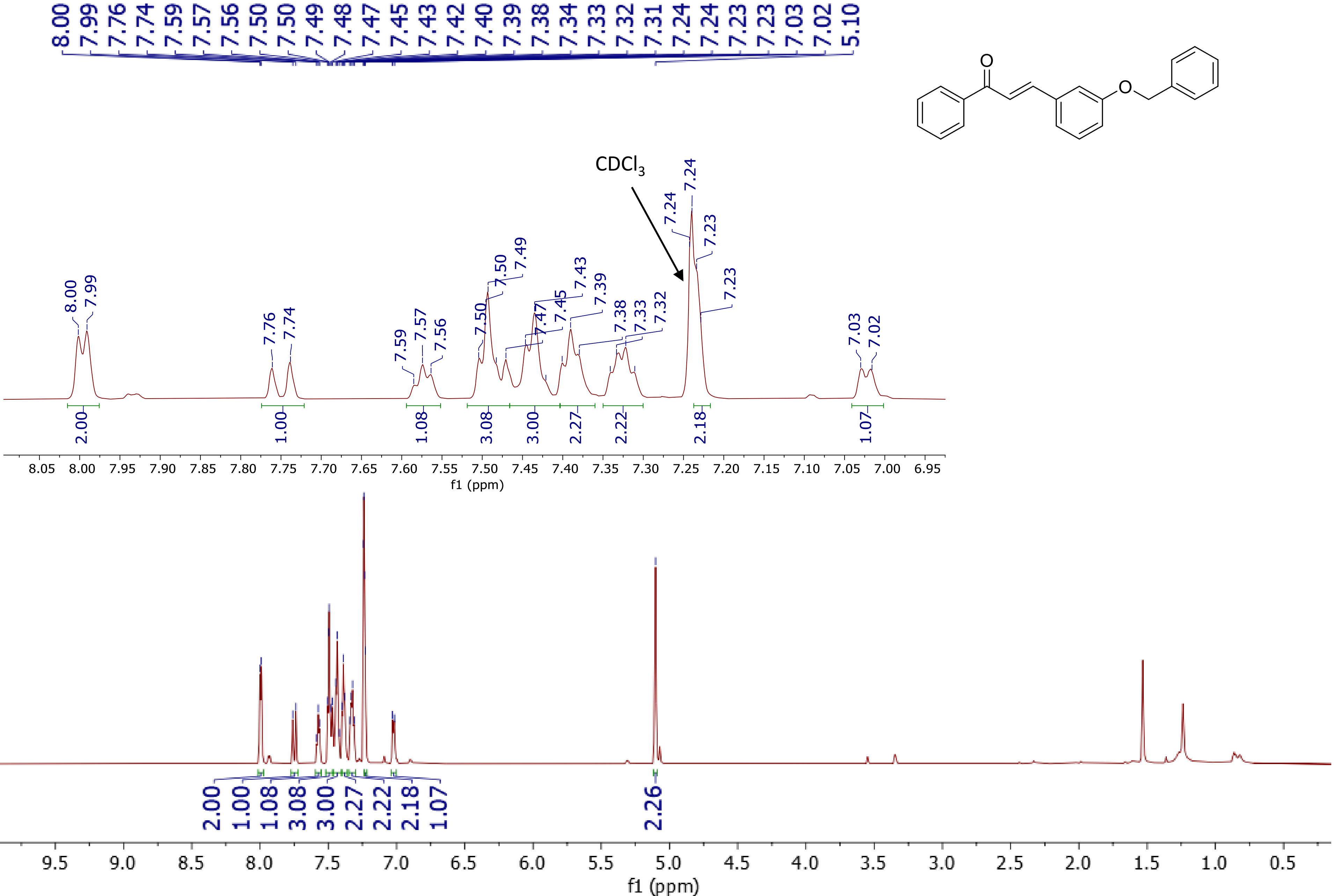

^{13}C-NMR Spectrum of **4h**

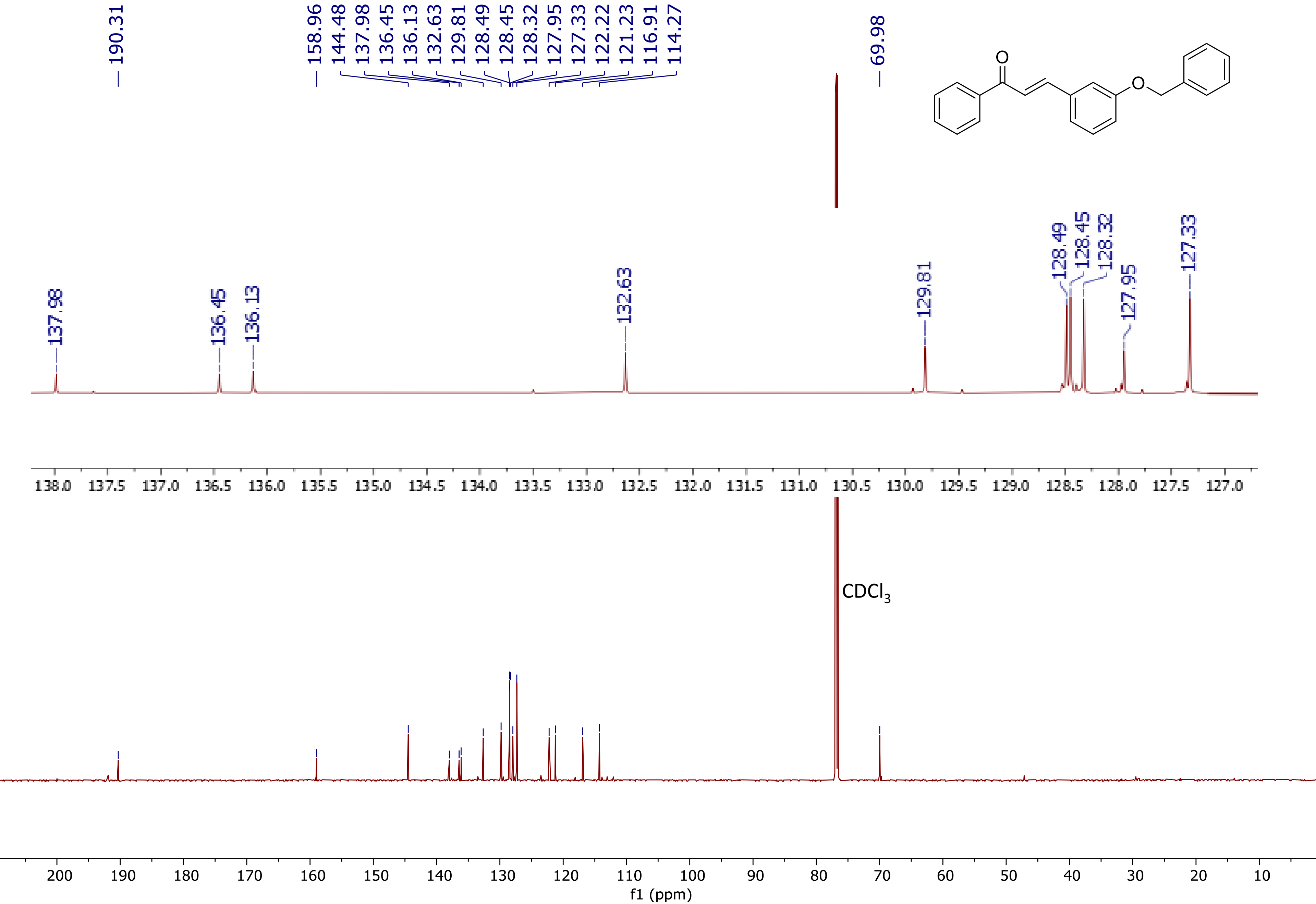

Mass Spectrum of **4h**

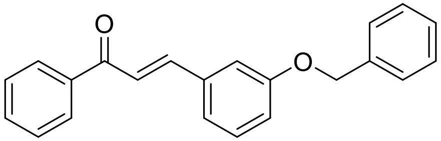

Chemical Formula: $C_{22}H_{18}O_2$

Exact Mass: 314.13

m/z: 315 $[M+H]^+$; 337 $[M+Na]^+$

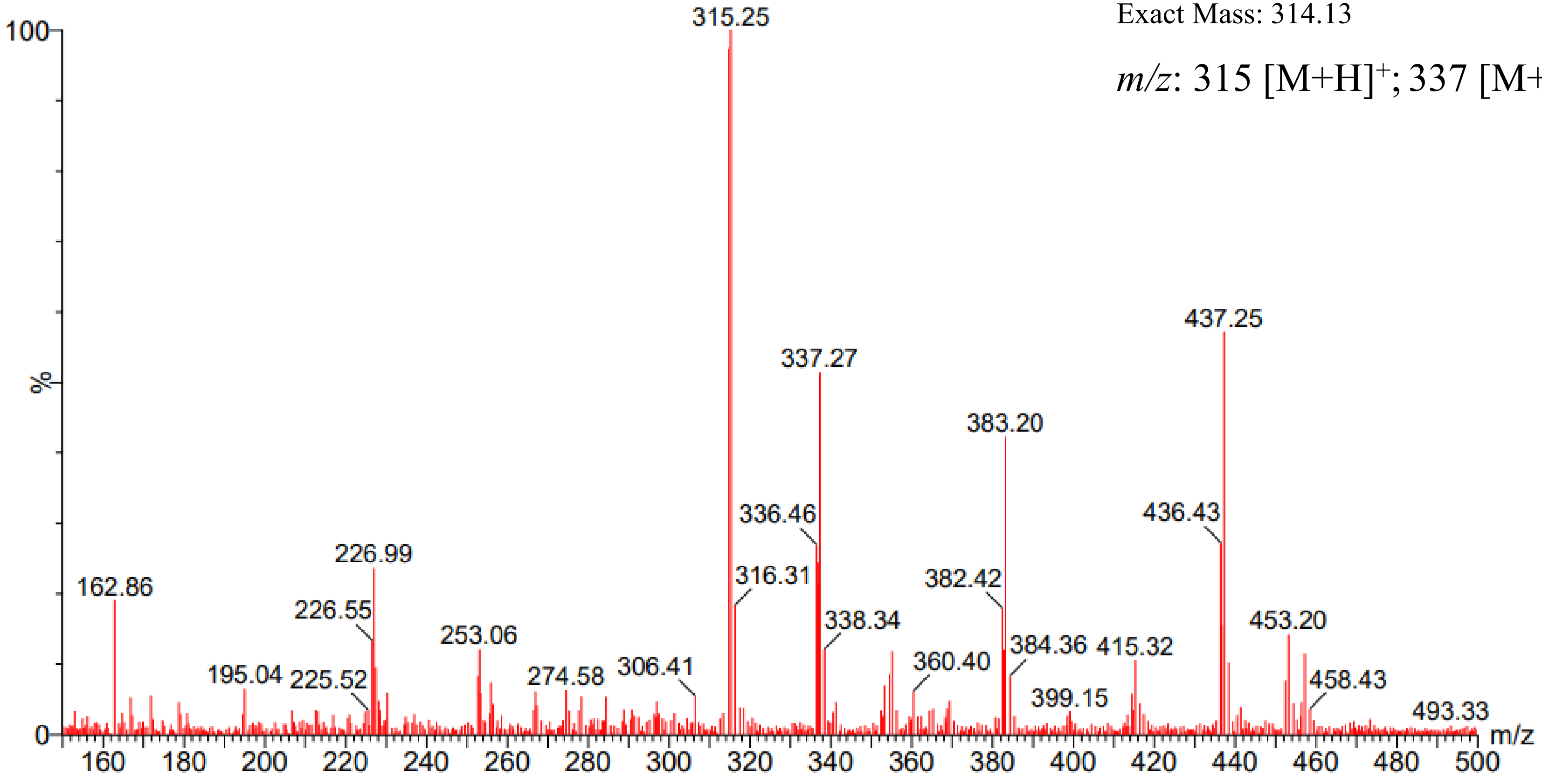

IR Spectrum of **4i**

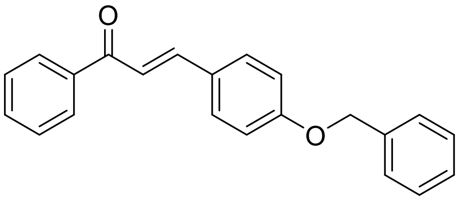

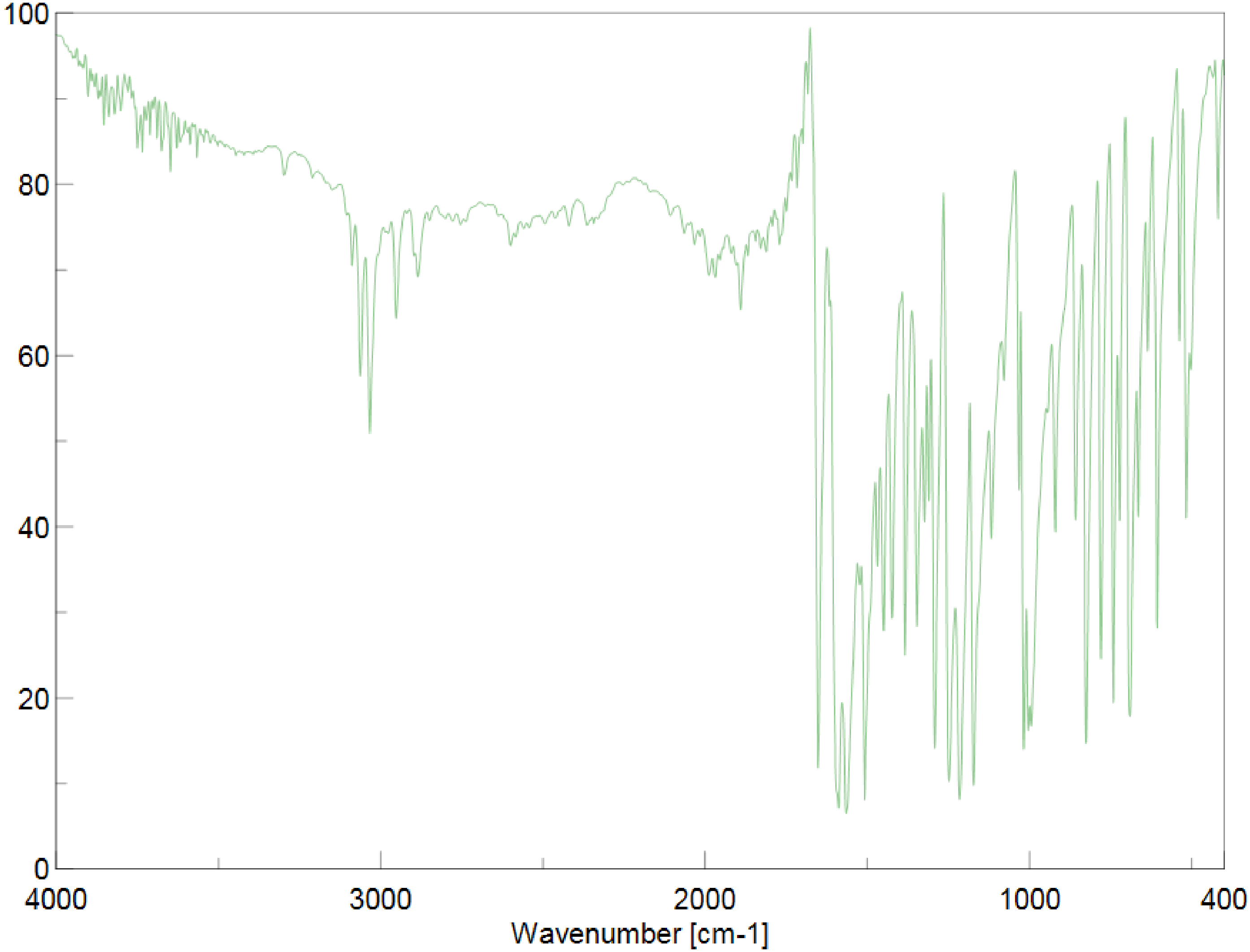

^{1}H-NMR Spectrum of **4i**

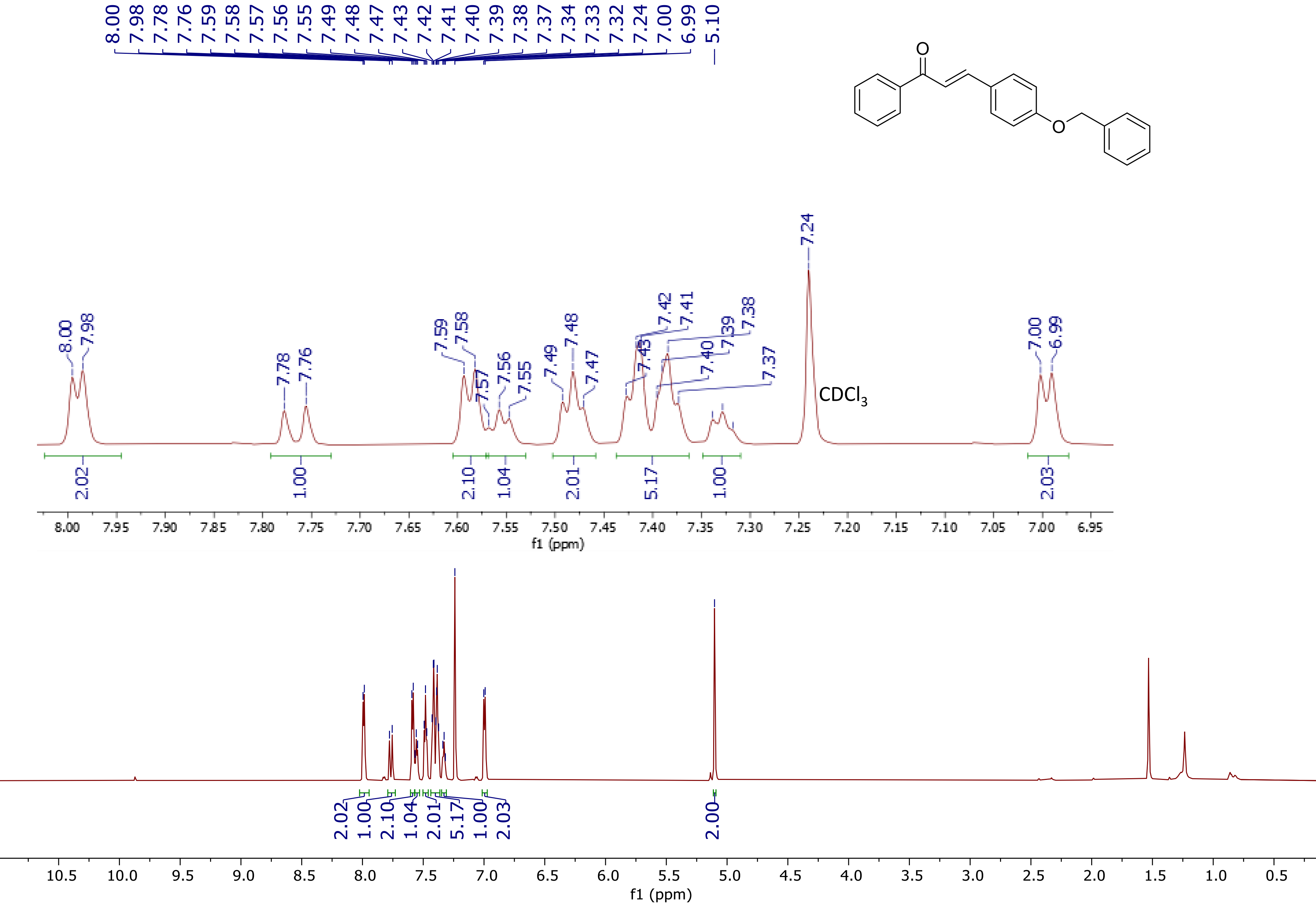

^{13}C-NMR Spectrum of **4i**

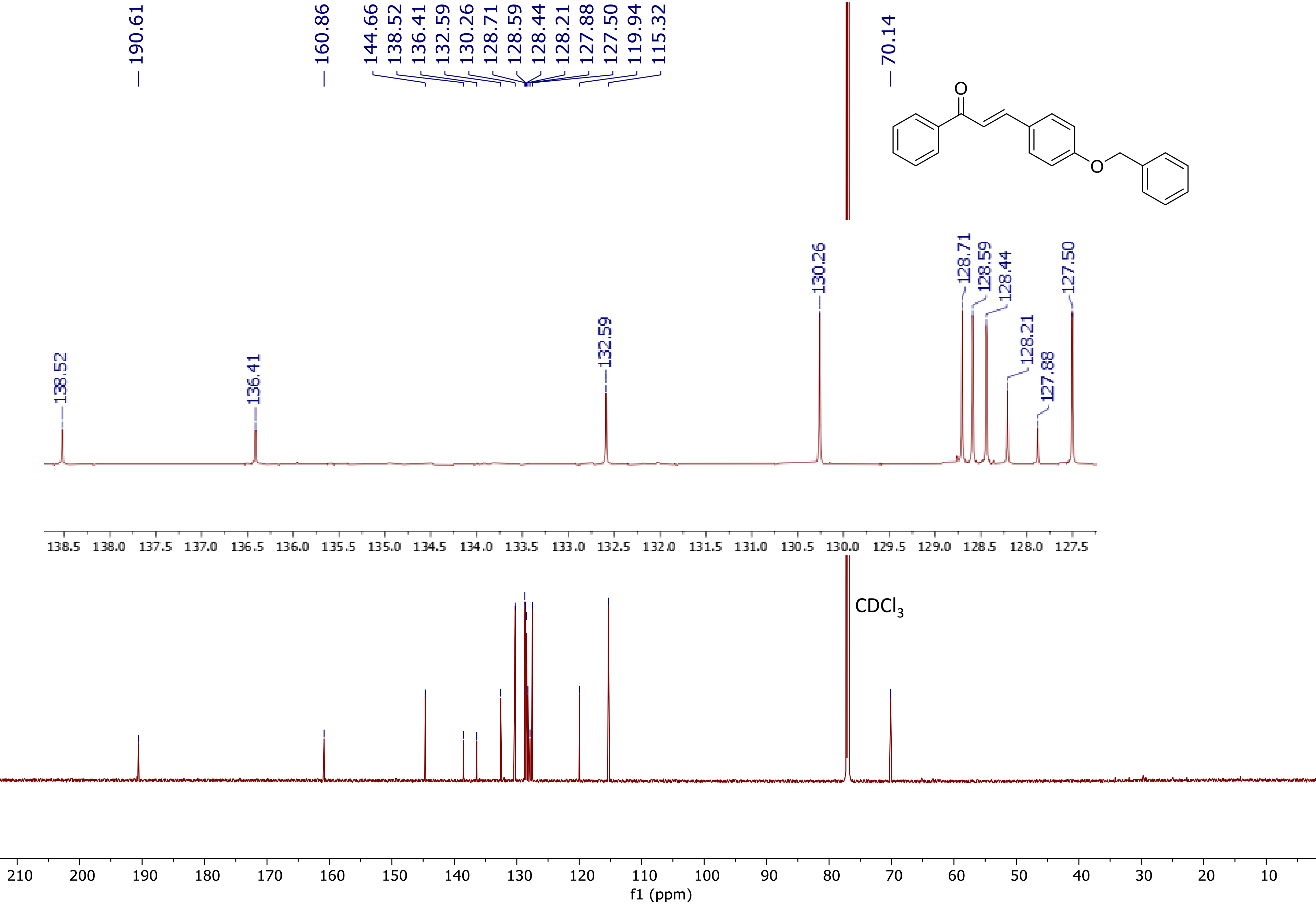

Mass Spectrum of **4i**

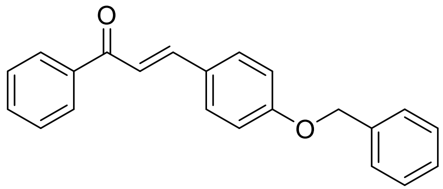

Chemical Formula: $C_{22}H_{18}O_2$

Exact Mass: 314.13

m/z: 315 $[M+H]^+$; 337 $[M+Na]^+$

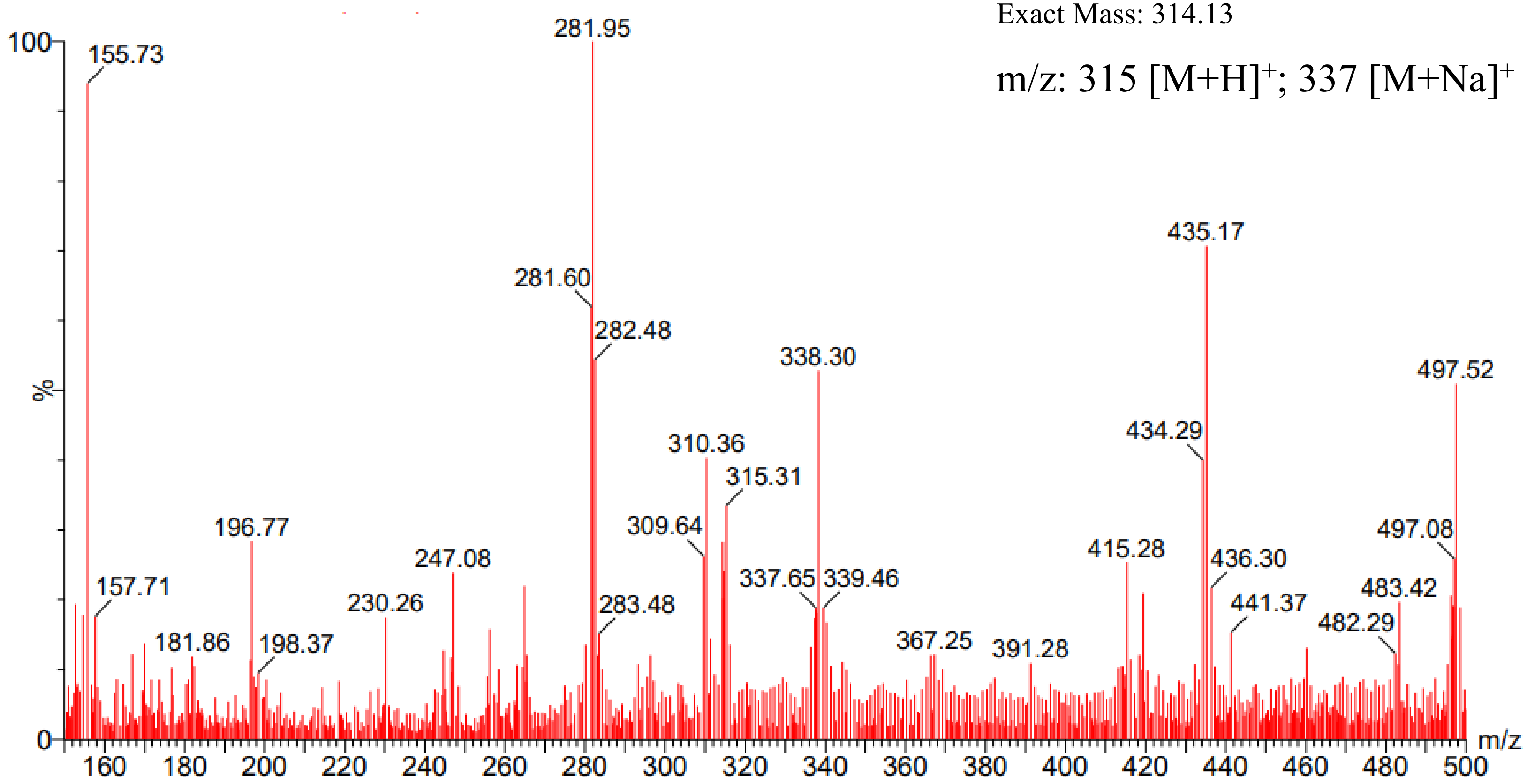

IR Spectrum of **4j**

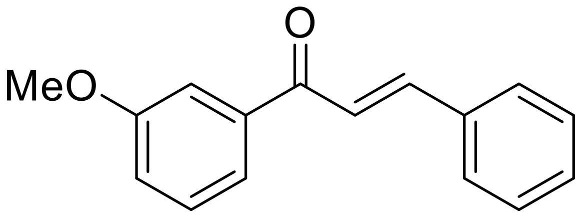

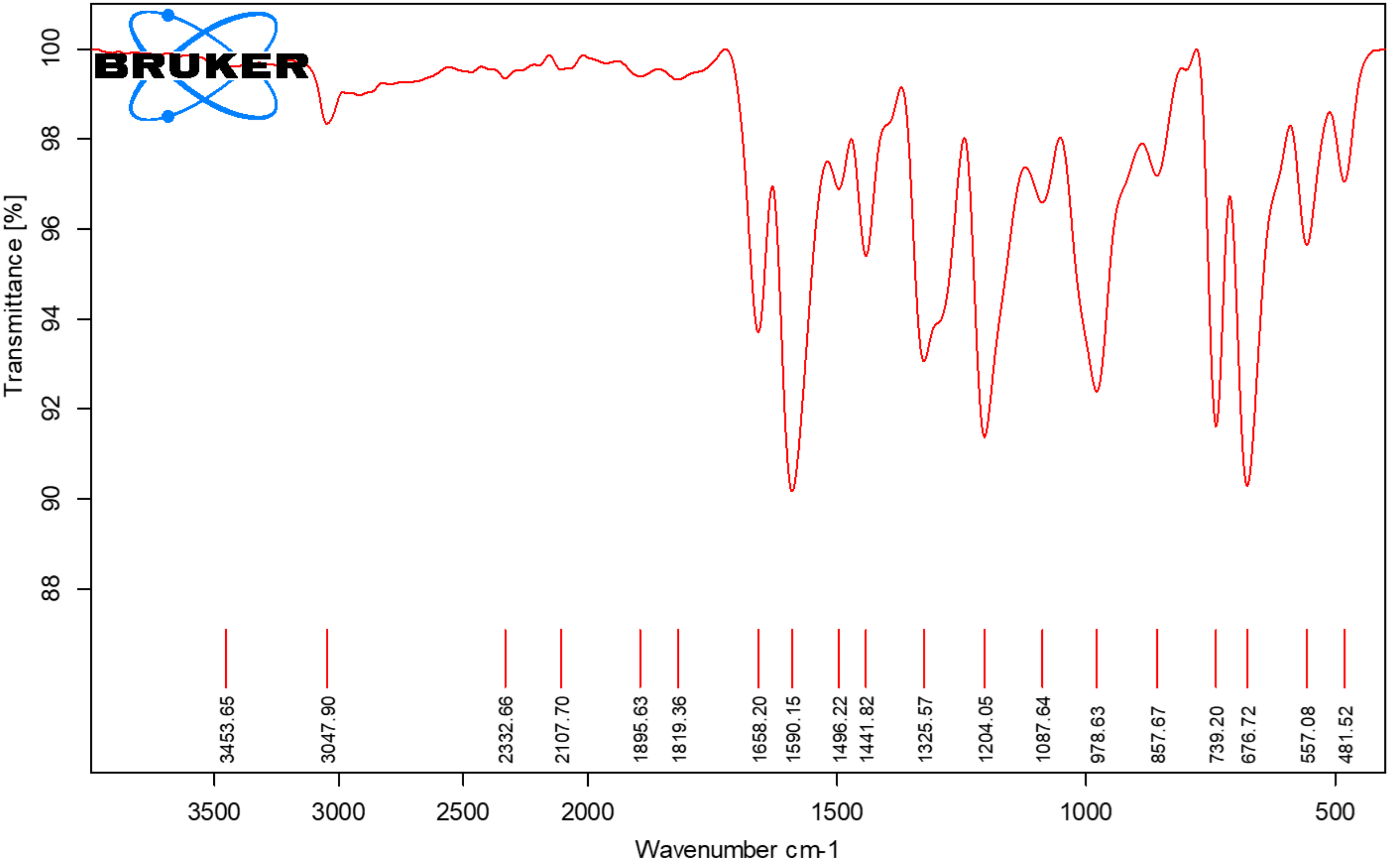

^{1}H-NMR Spectrum of **4j**

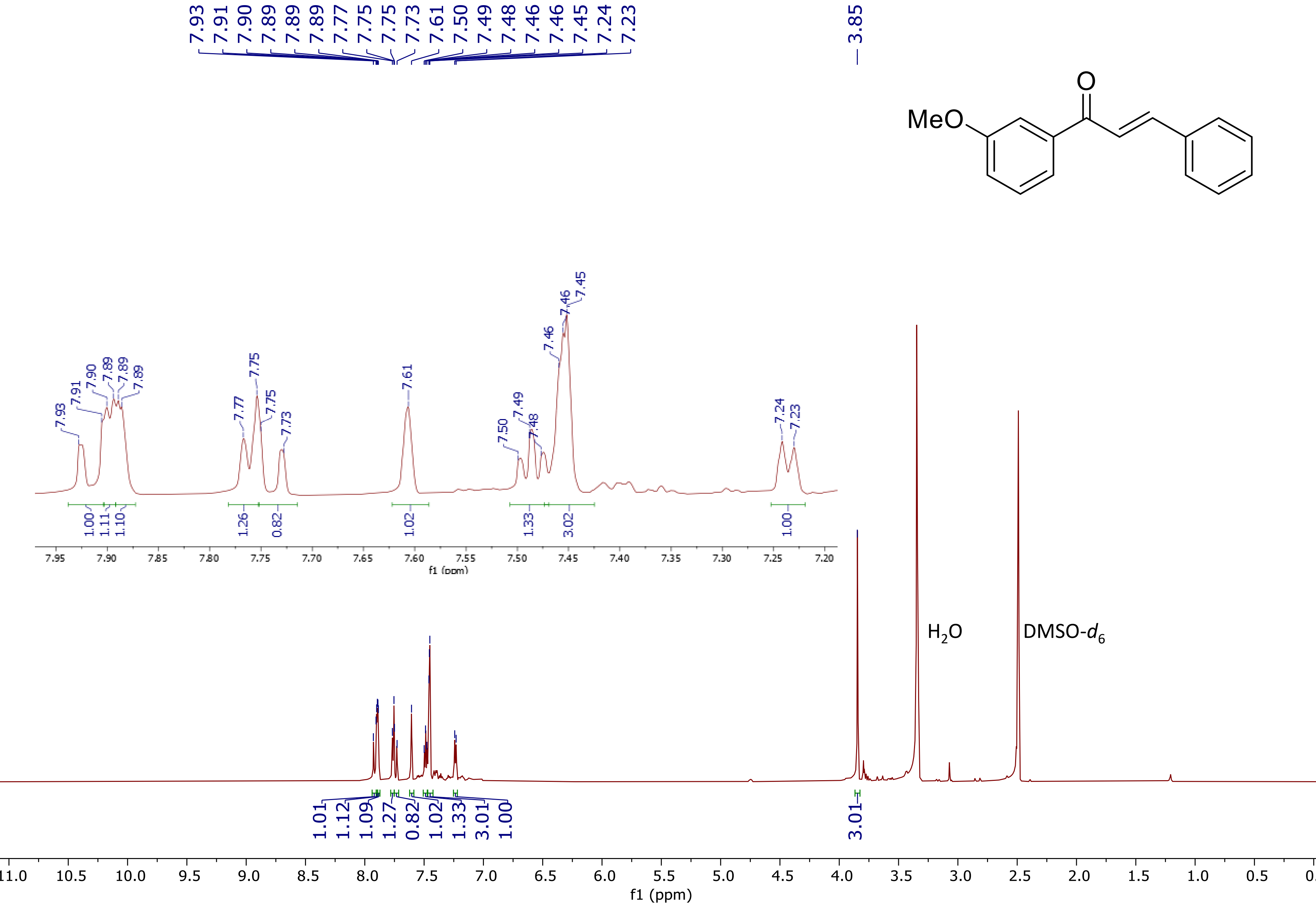

^{13}C-NMR Spectrum of **4j**

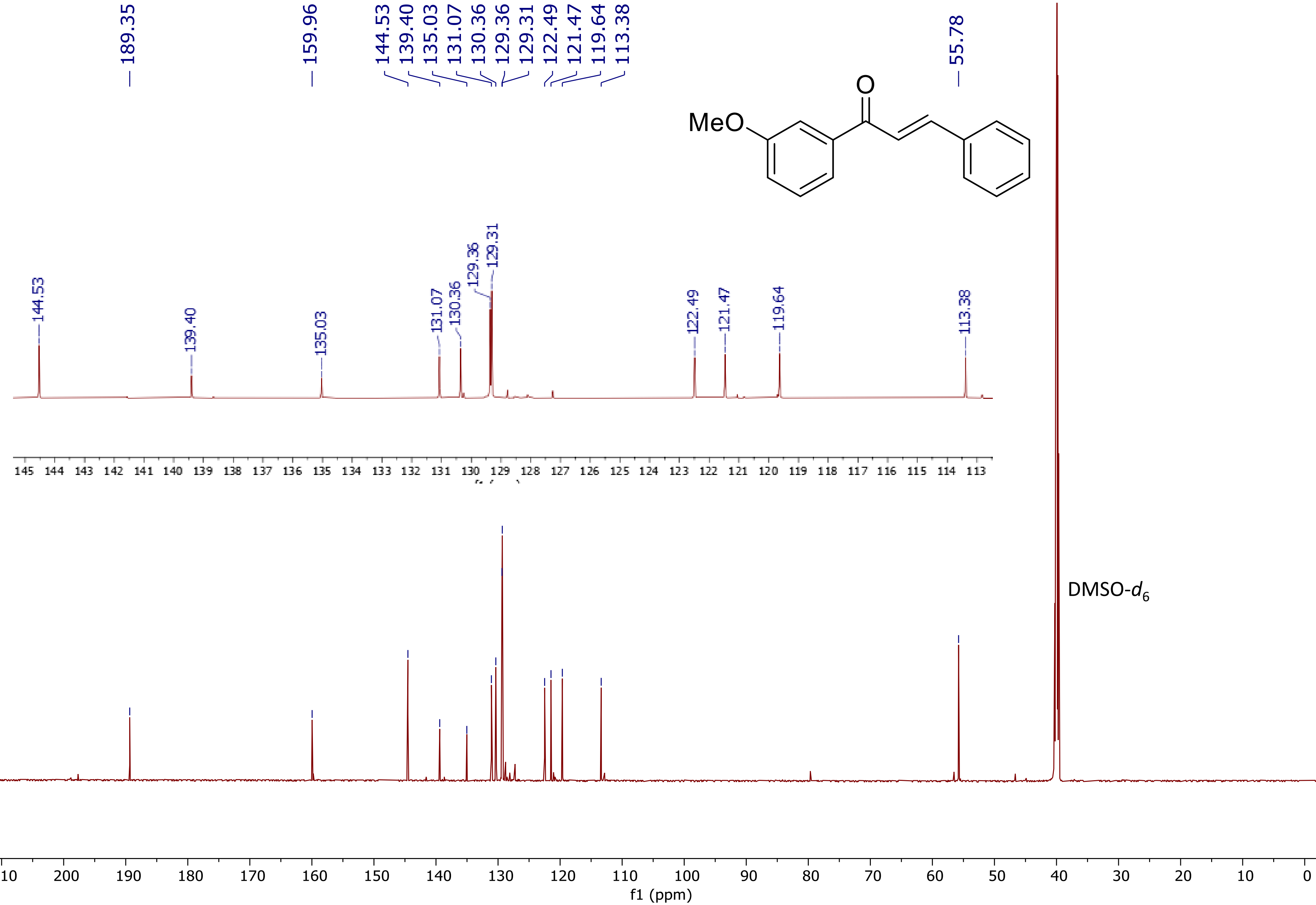

Mass Spectrum of **4j**

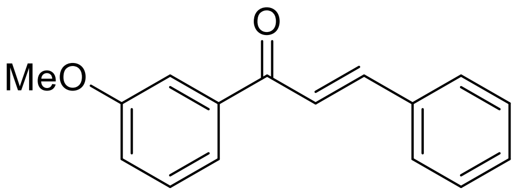

Chemical Formula: $C_{16}H_{14}O_2$

Exact Mass: 238.10

m/z: 239 $[M+H]^+$

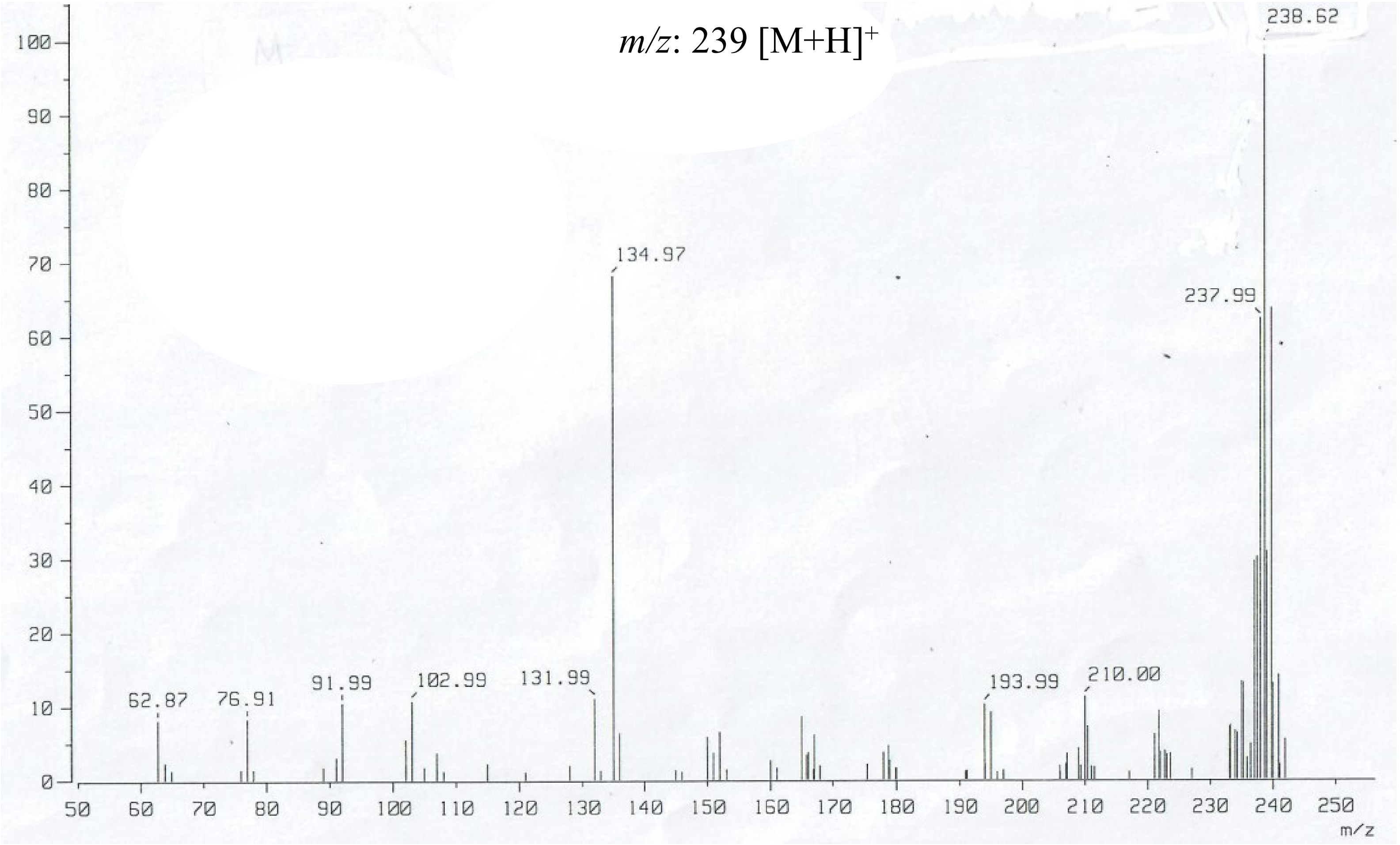

IR Spectrum of **4k**

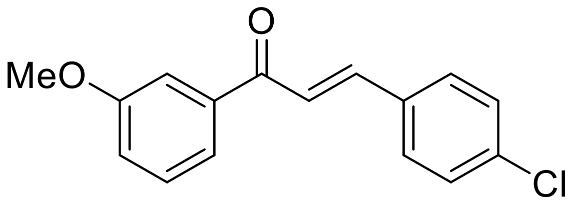

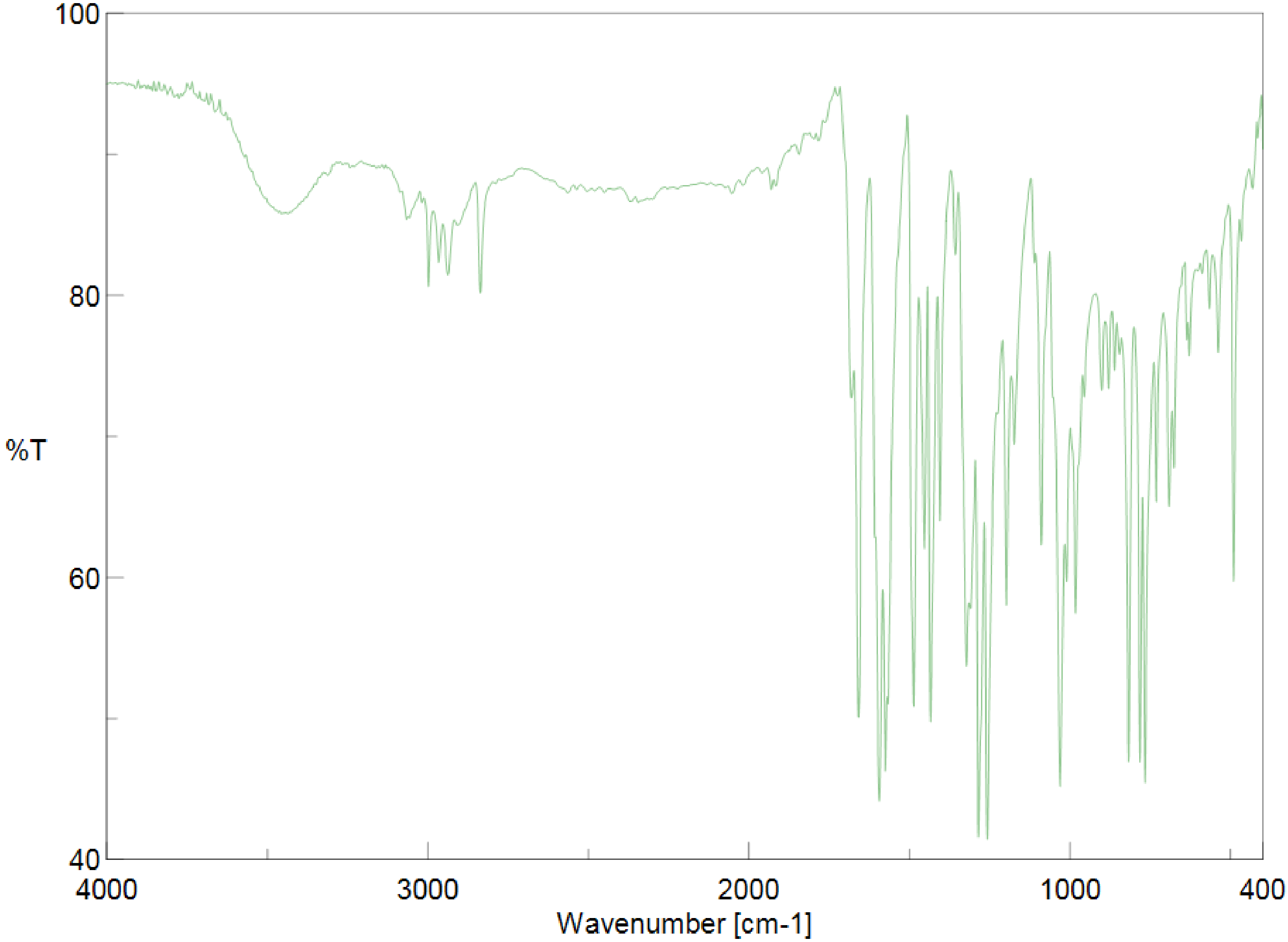

^{1}H-NMR Spectrum of **4k**

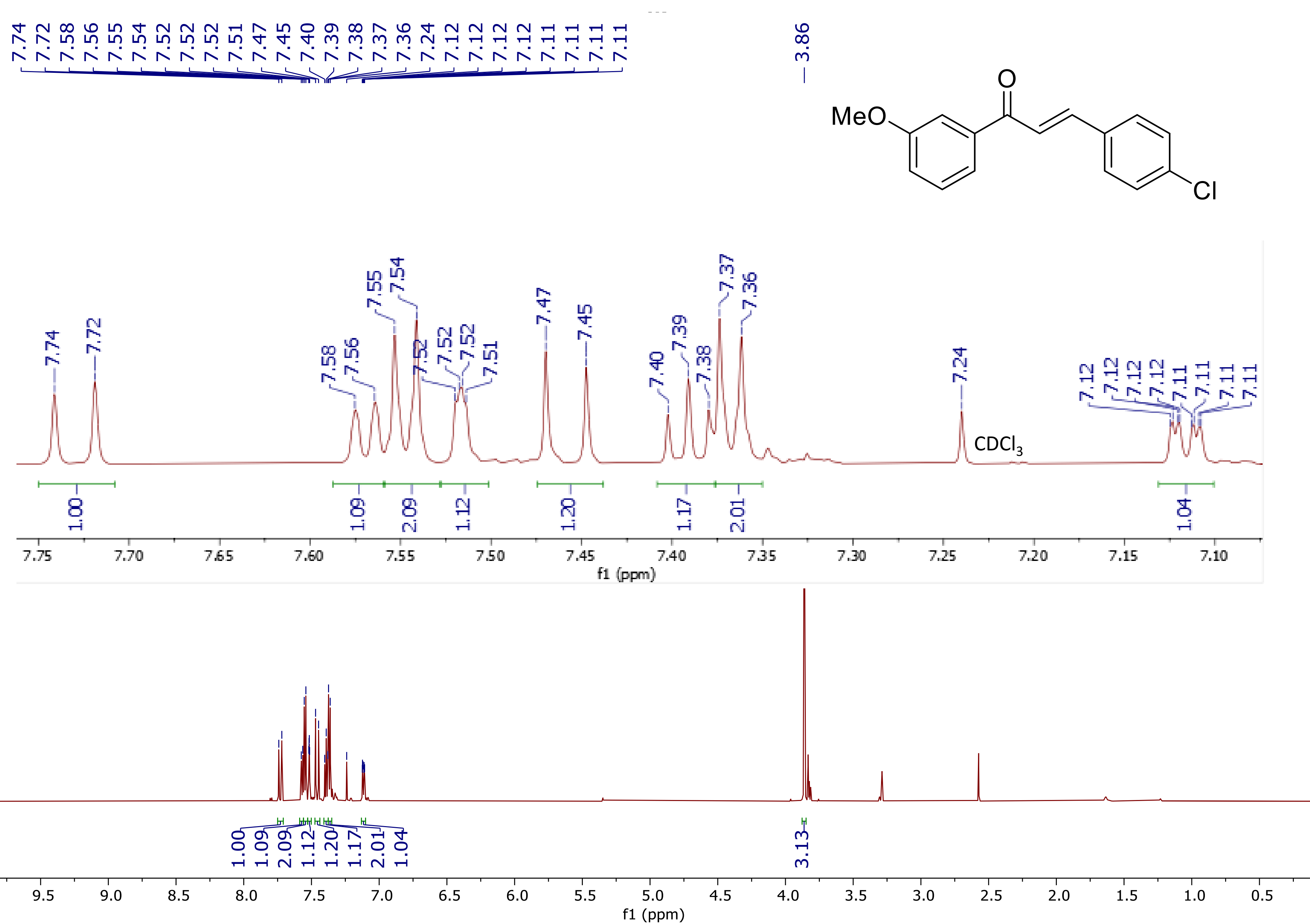

^{13}C-NMR Spectrum of **4k**

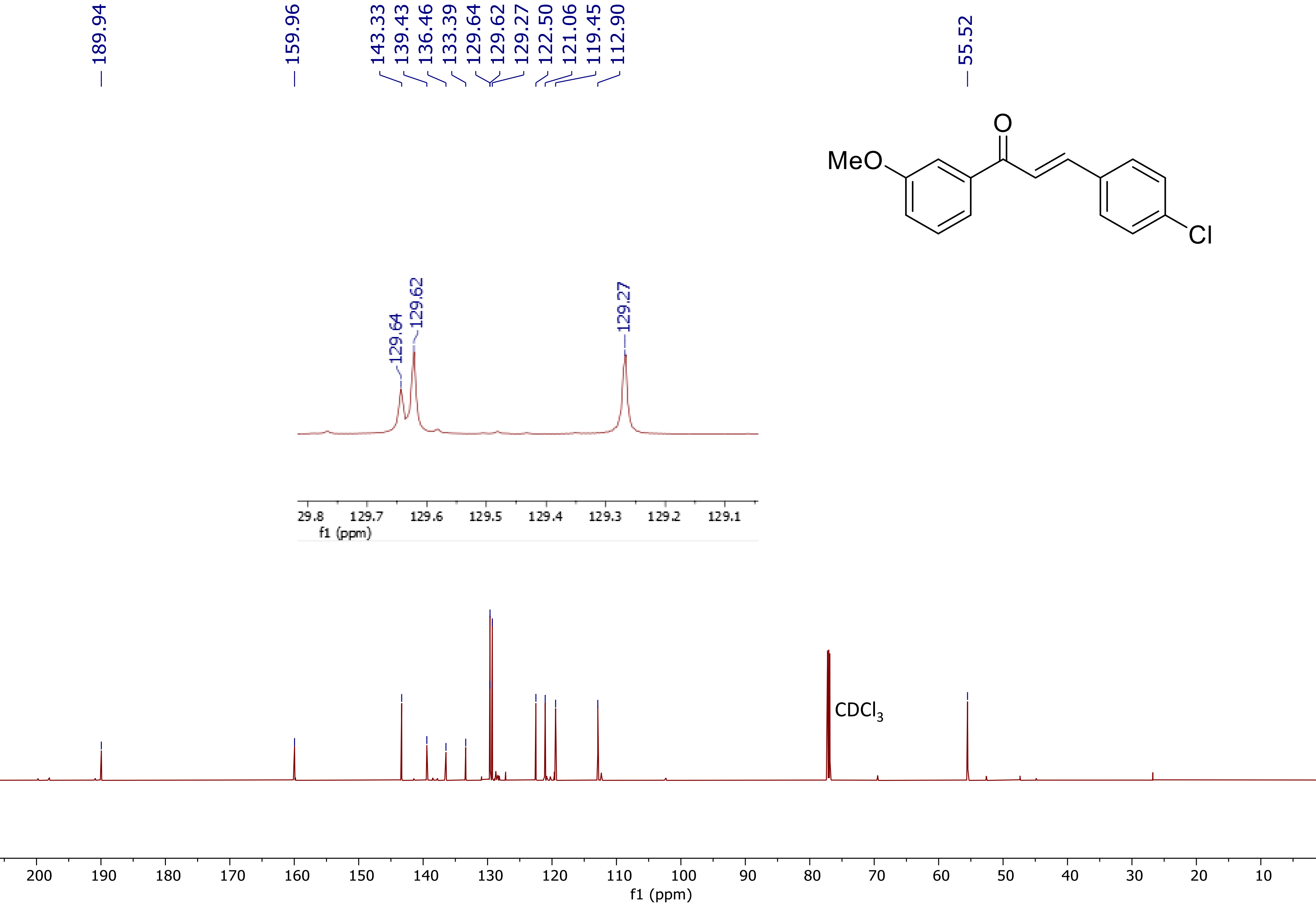

Mass Spectrum of **4k**

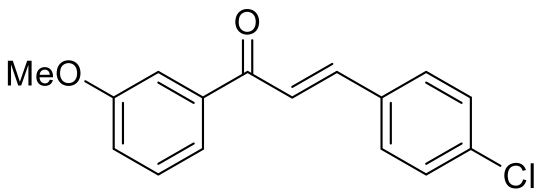

Chemical Formula: $C_{16}H_{13}ClO_2$

Exact Mass: 272.06

m/z: 273 $[M+H]^+$; 295 $[M+Na]^+$

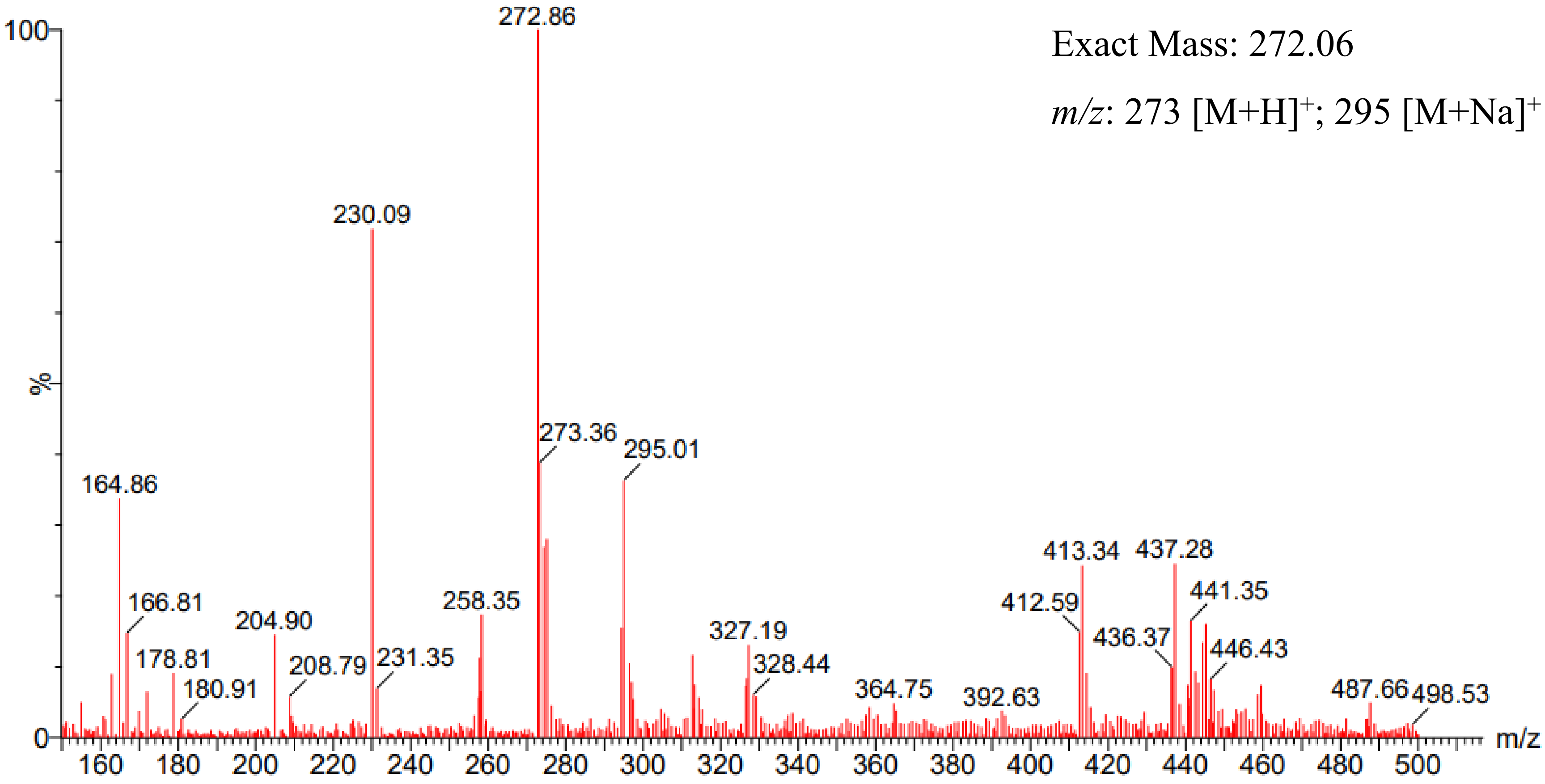

IR Spectrum of **4l**

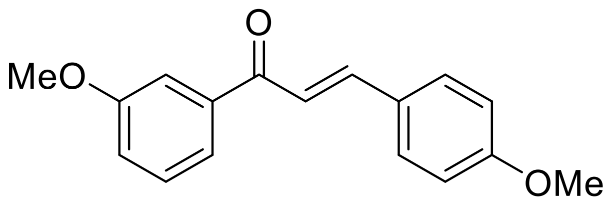

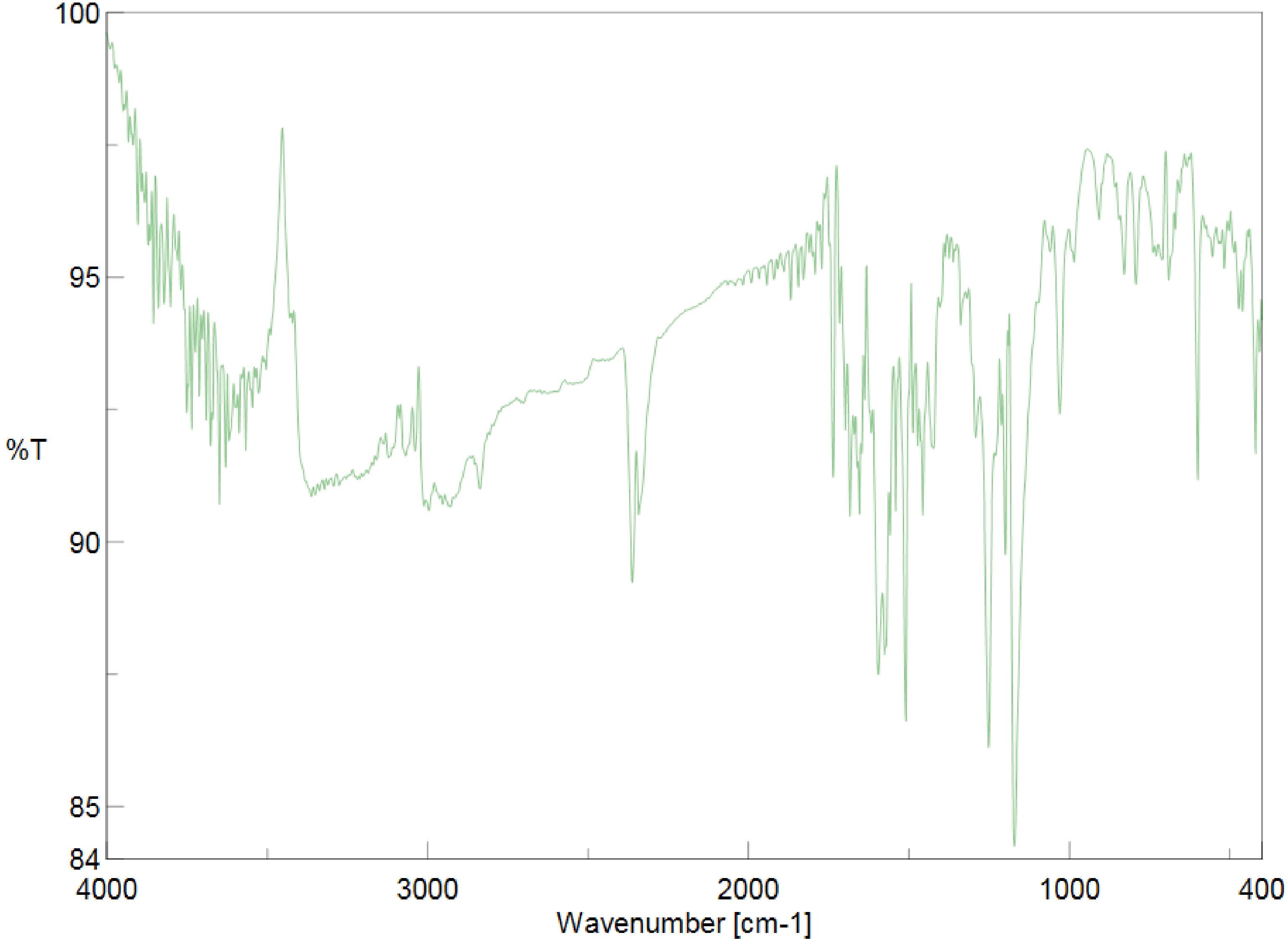

^{1}H-NMR Spectrum of **4l**

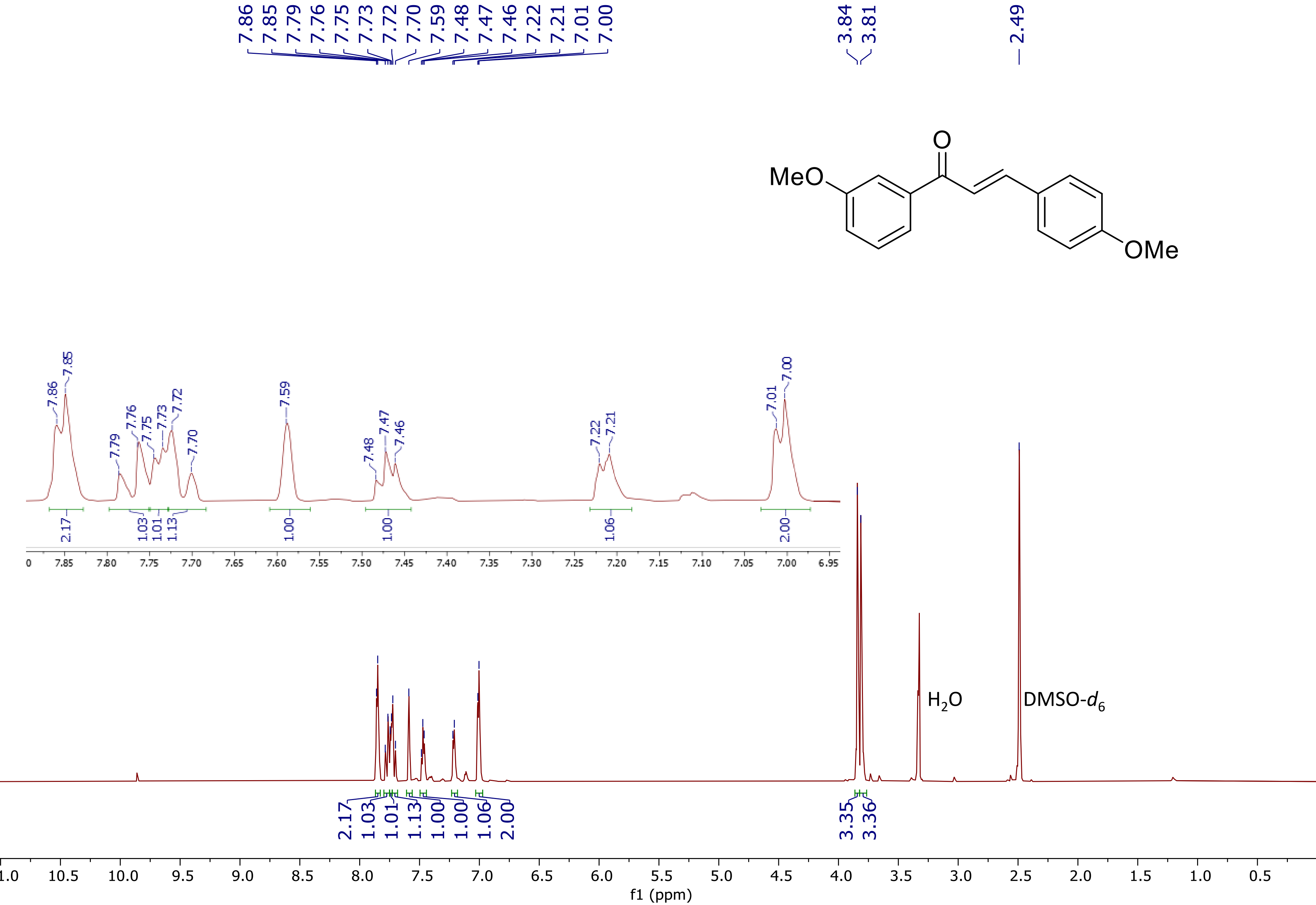

^{13}C-NMR Spectrum of **4l**

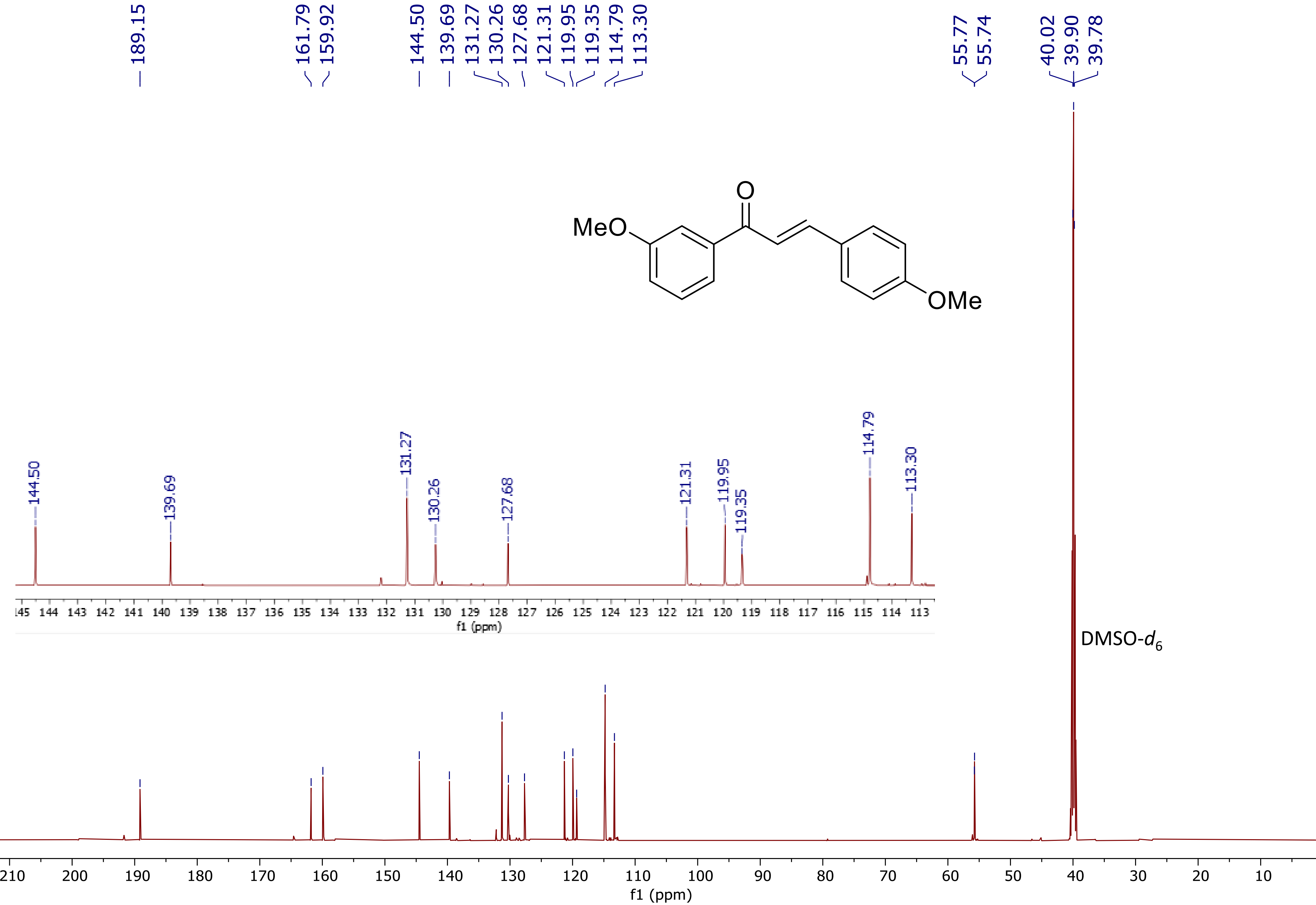

Mass Spectrum of **4l**

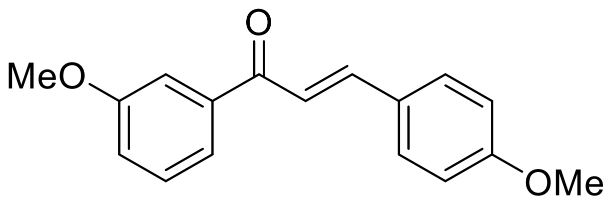

Chemical Formula: $C_{17}H_{16}O_3$

Exact Mass: 268.11

m/z: 269 $[M+H]^+$; 291 $[M+Na]^+$

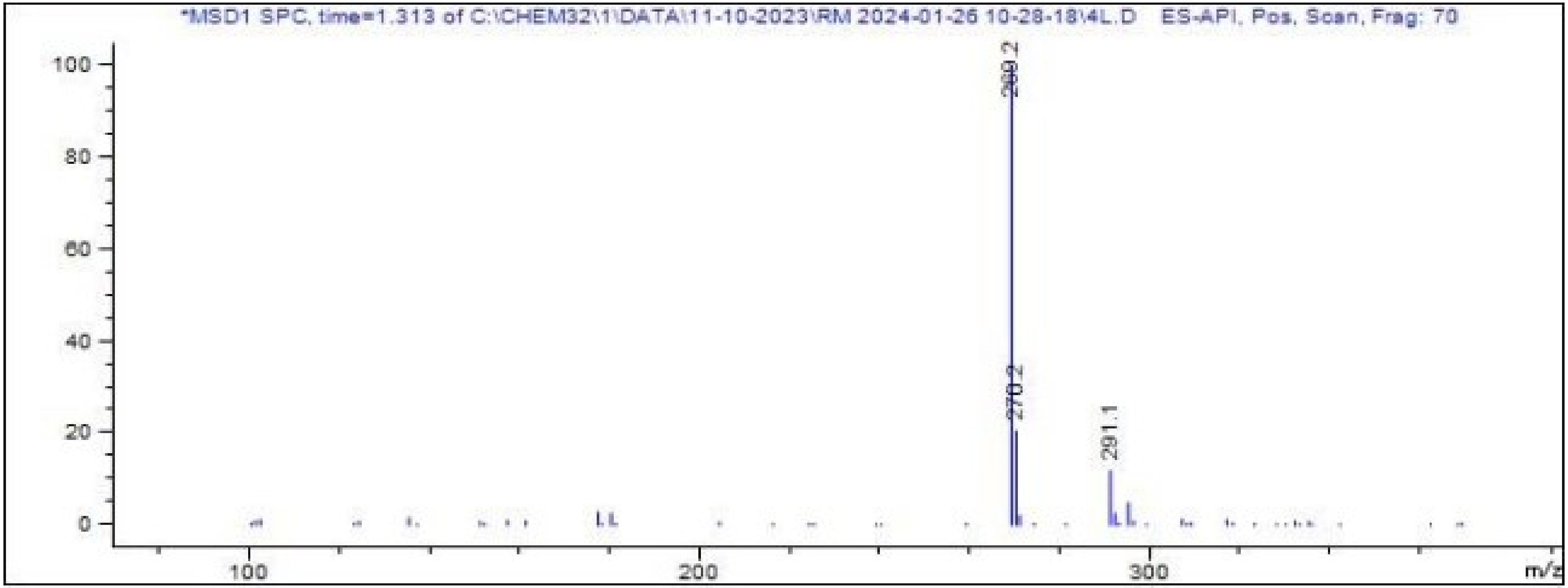

IR Spectrum of **4m**

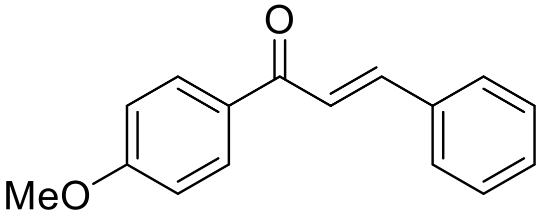

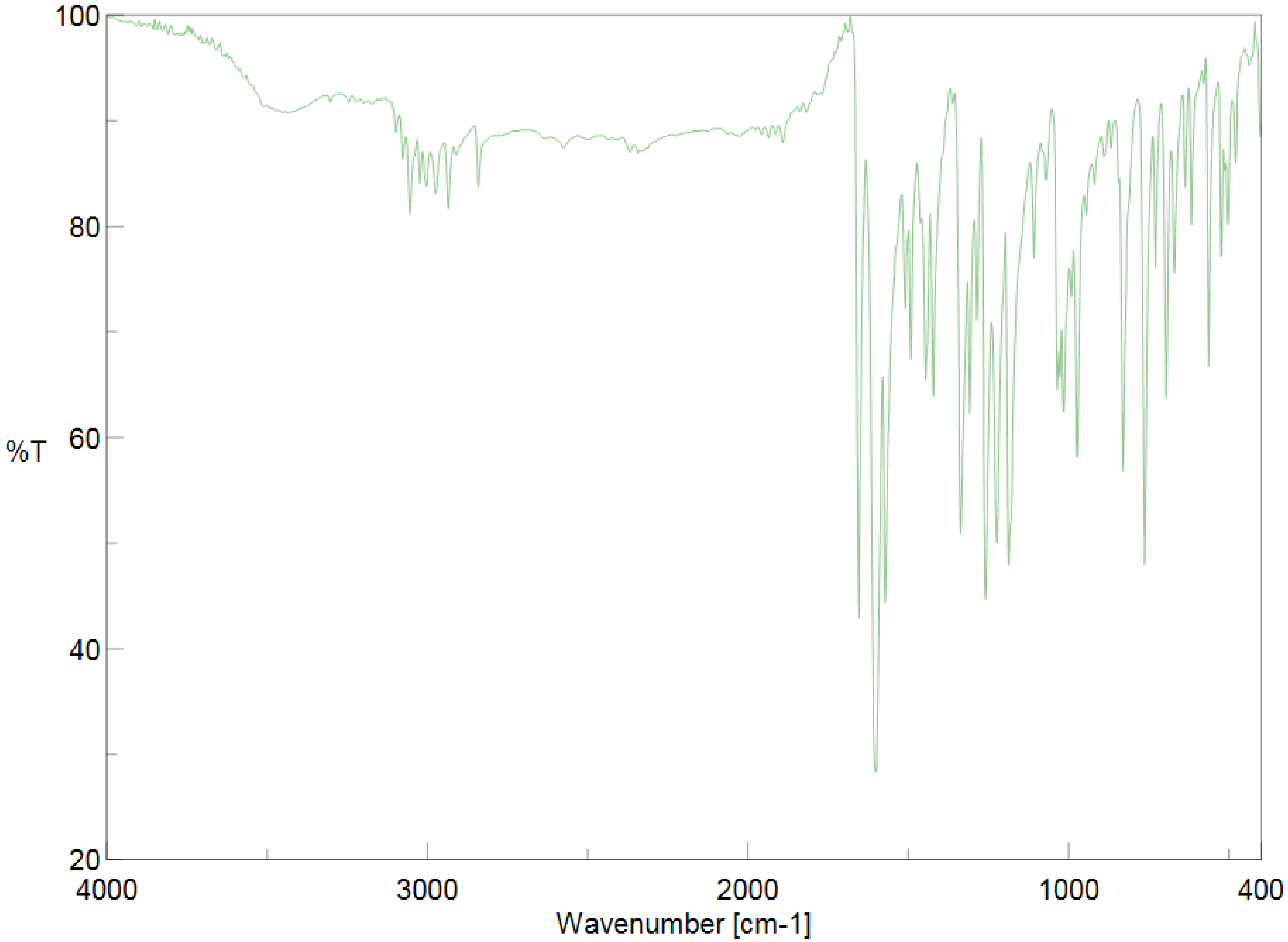

^{1}H-NMR Spectrum of **4m**

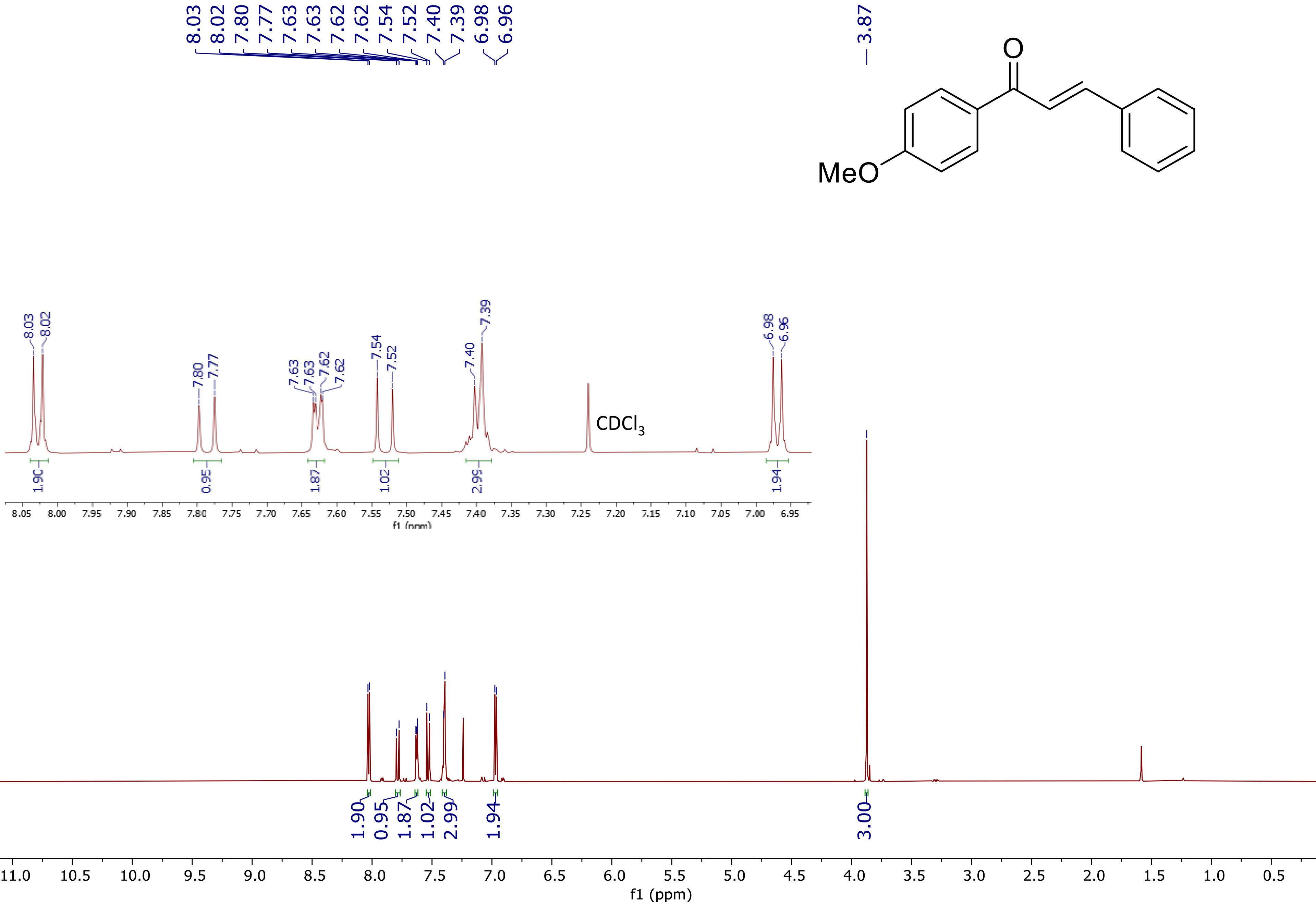

^{13}C-NMR Spectrum of **4m**

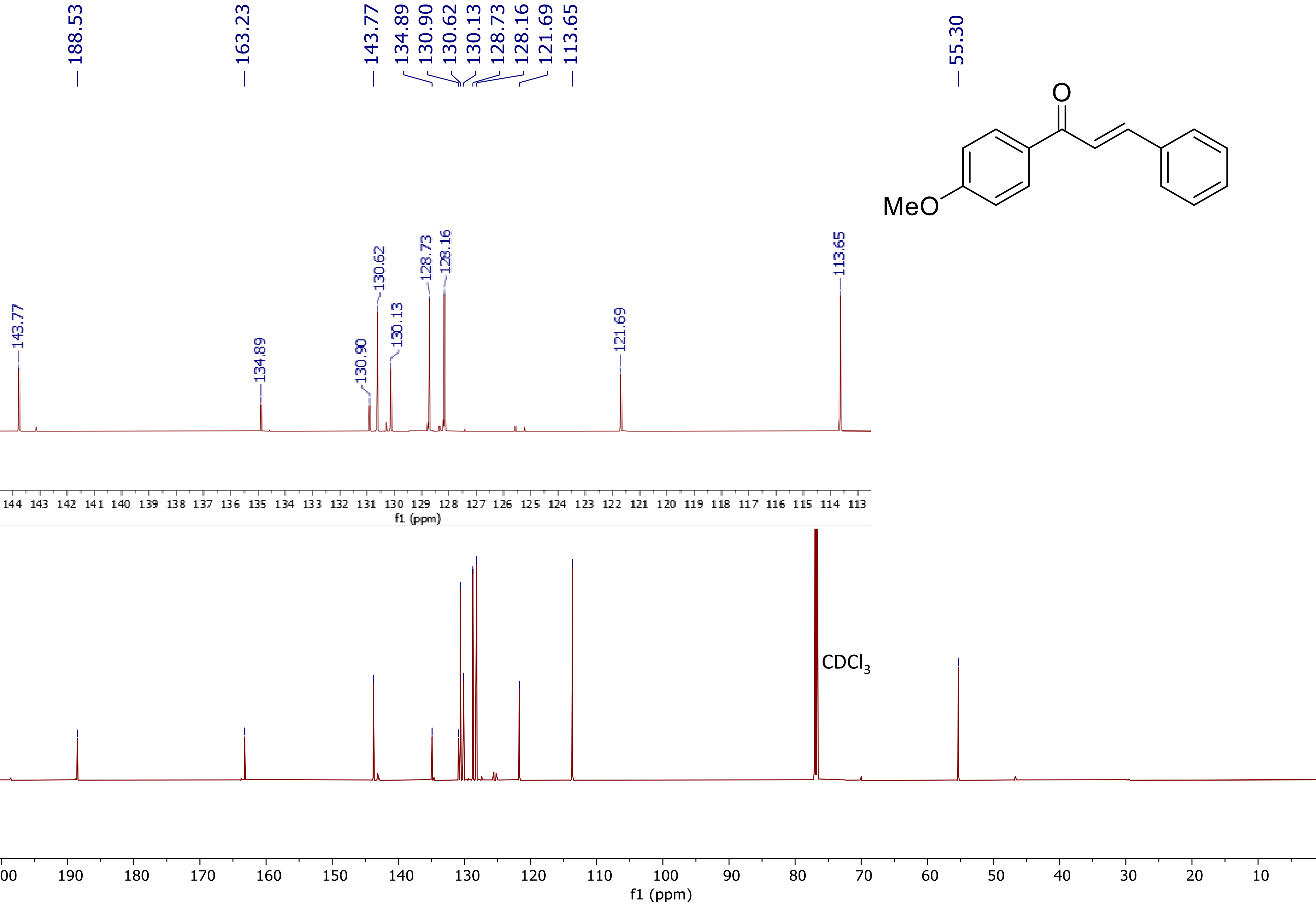

Mass Spectrum of **4m**

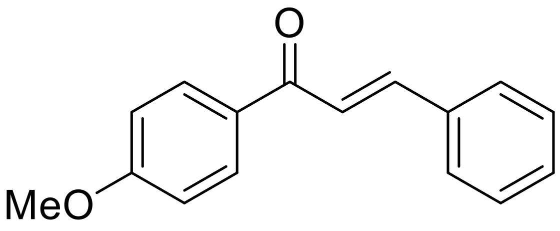

Chemical Formula: $C_{16}H_{14}O_2$

Exact Mass: 238.10

m/z: 239 $[M+H]^+$; 261 $[M+Na]^+$

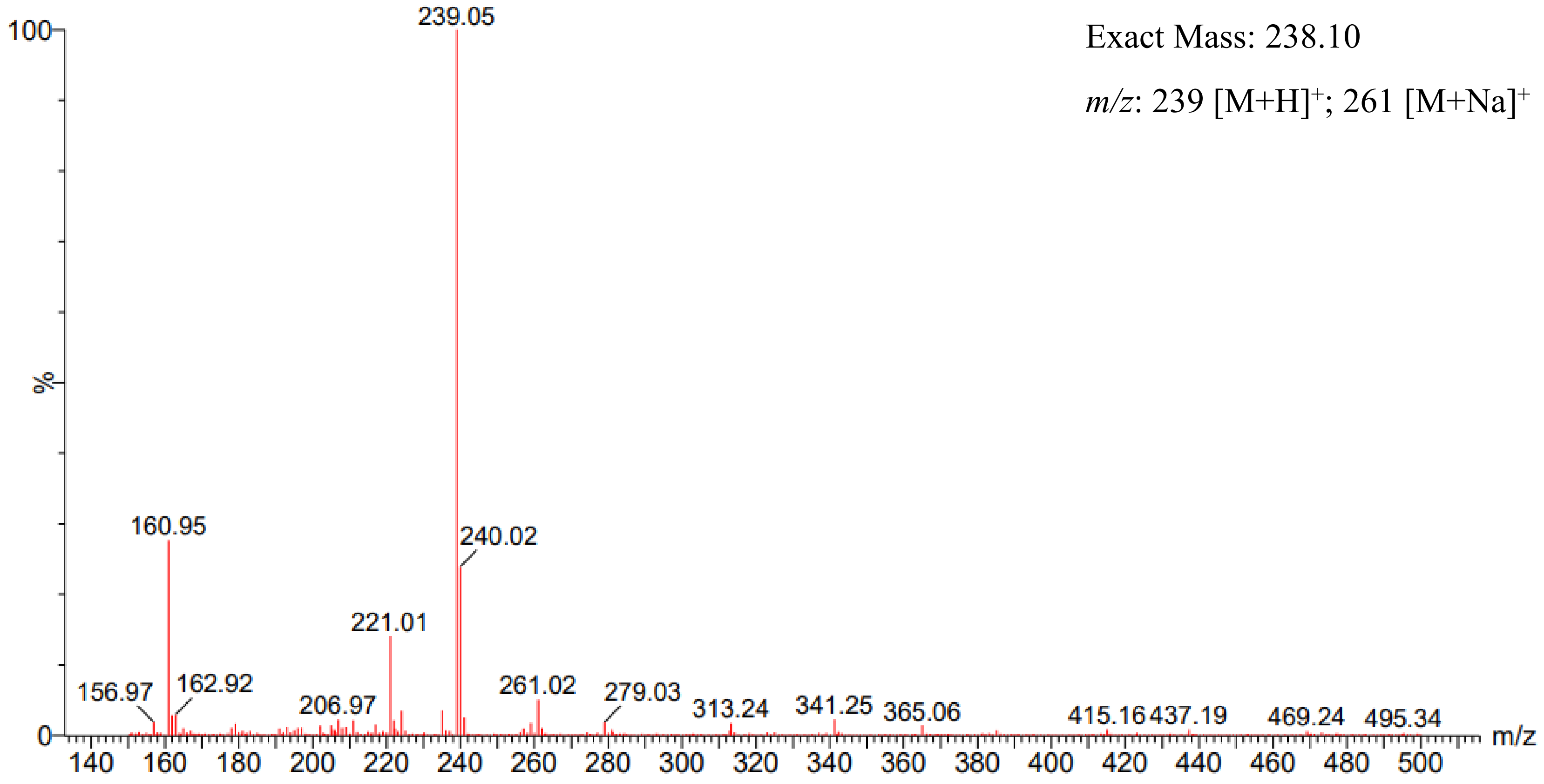

IR Spectrum of **4n**

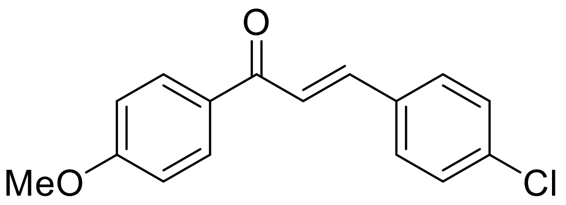

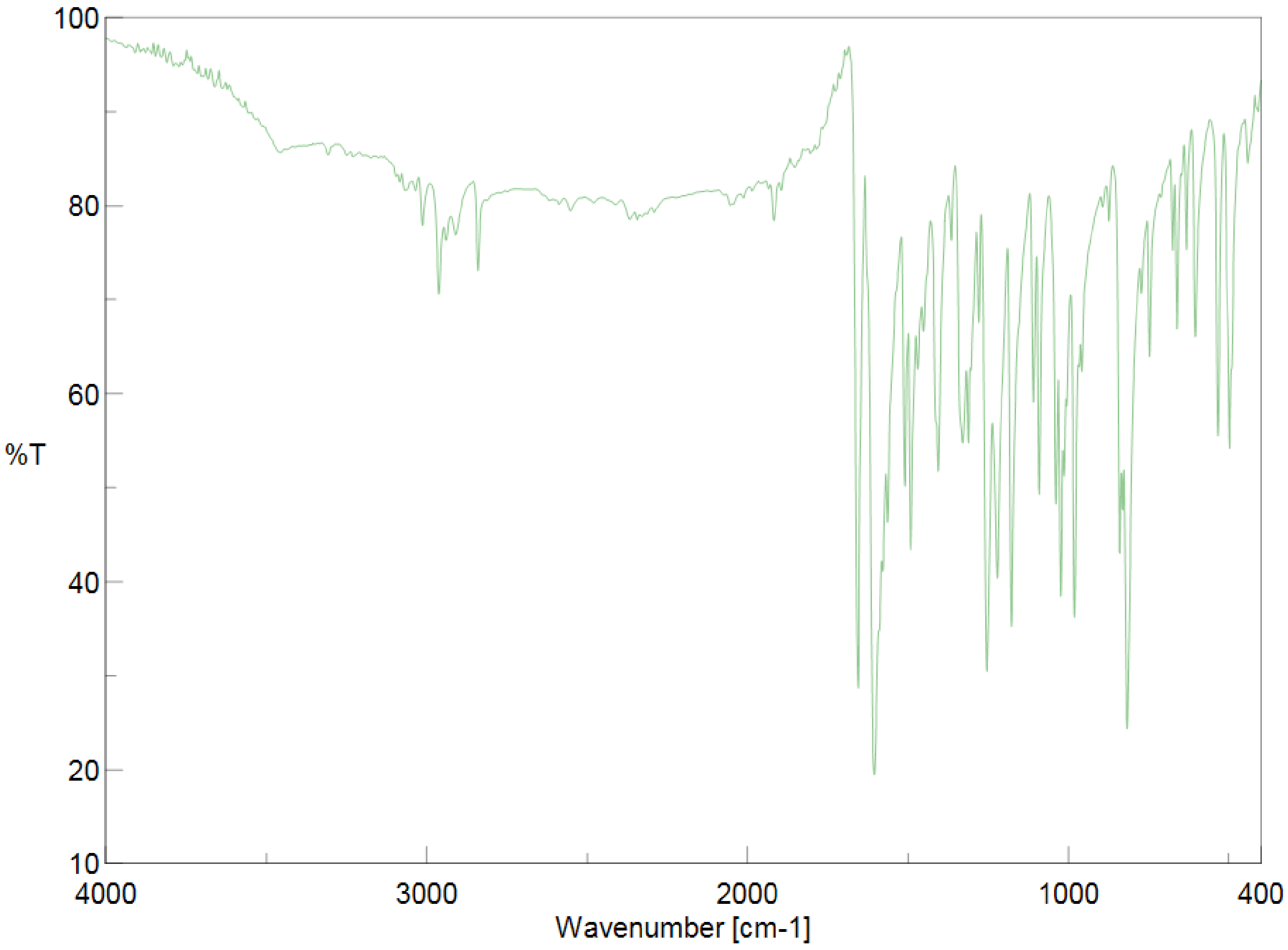

^{1}H-NMR Spectrum of **4n**

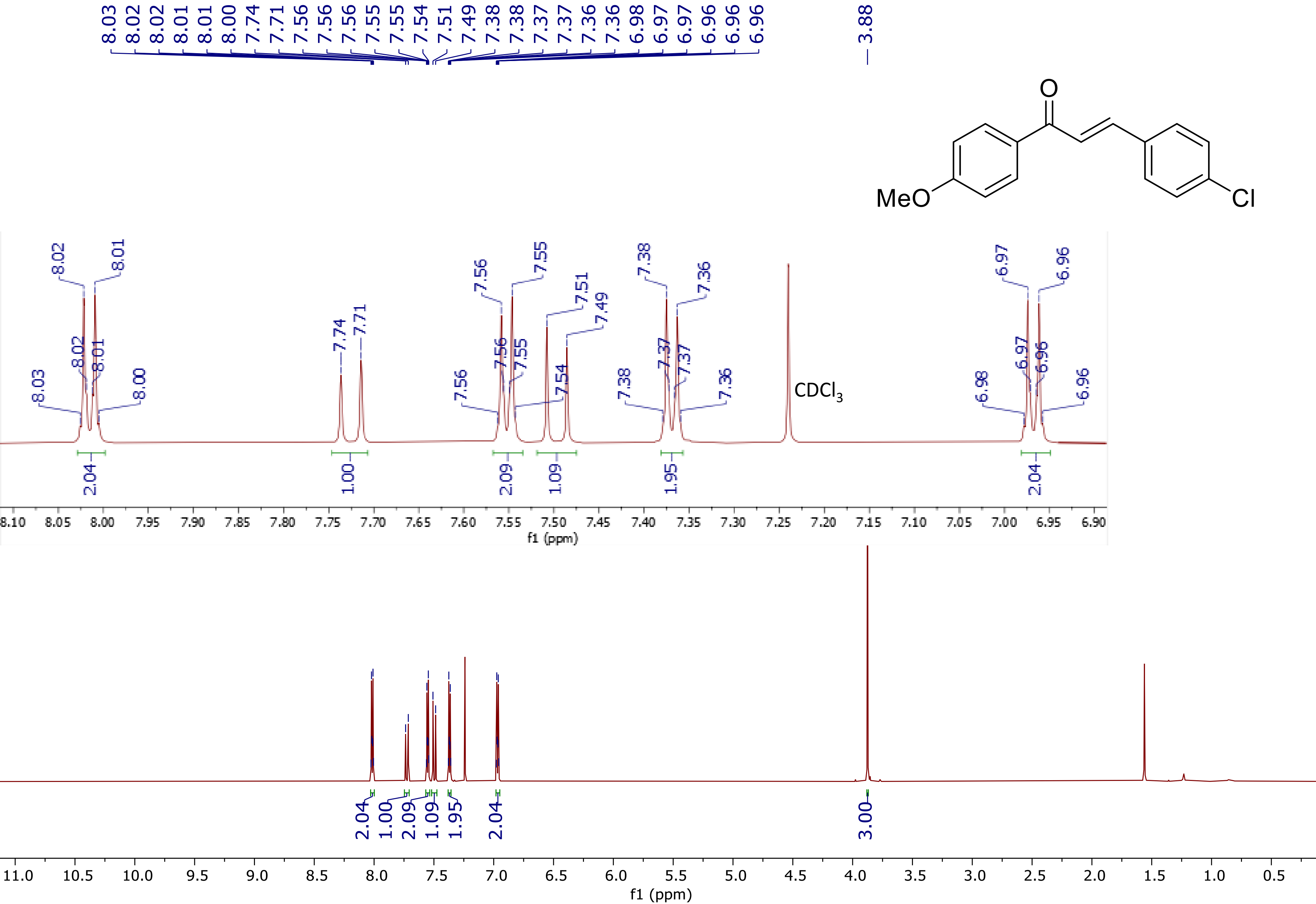

^{13}C-NMR Spectrum of **4n**

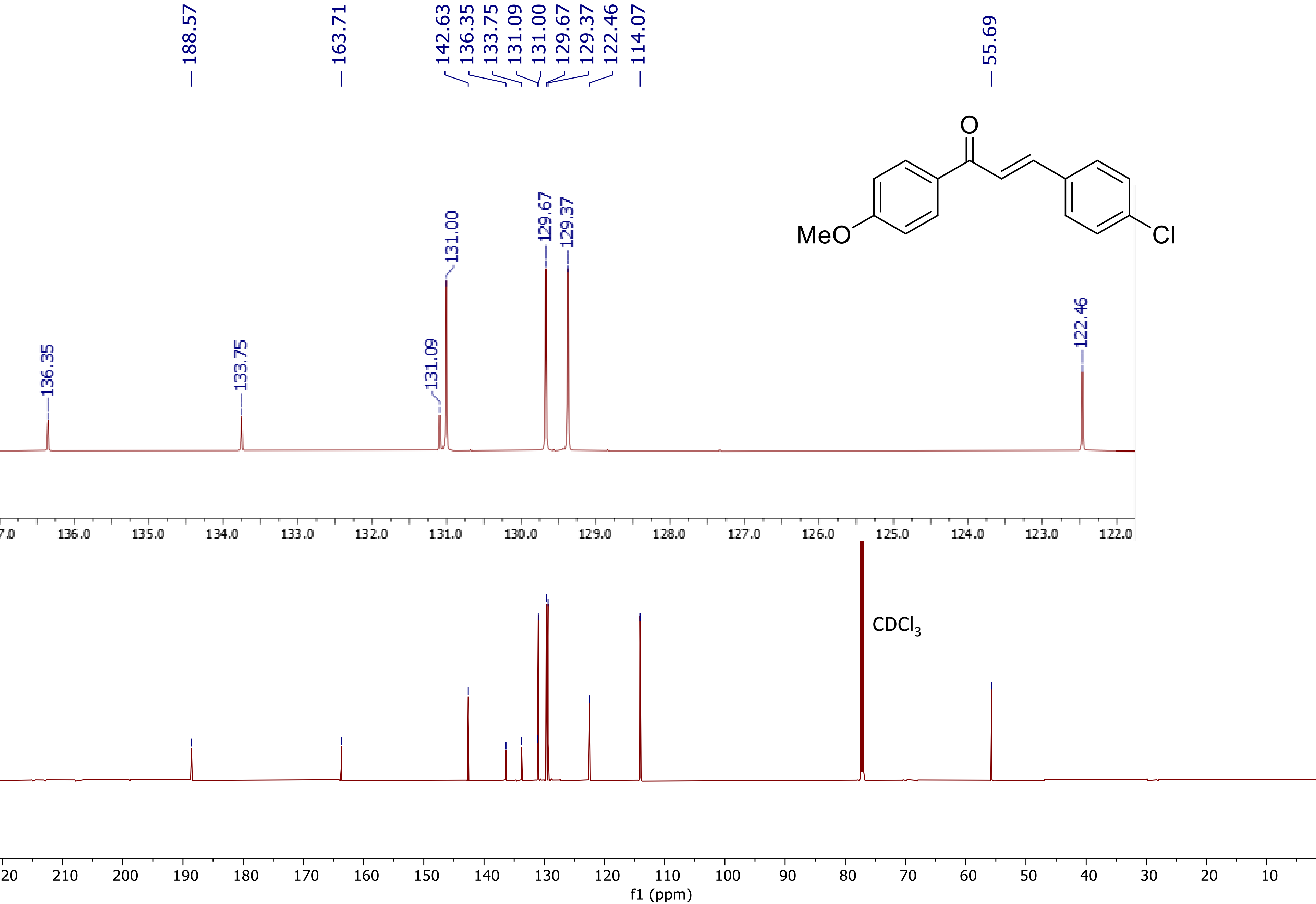

Mass Spectrum of **4n**

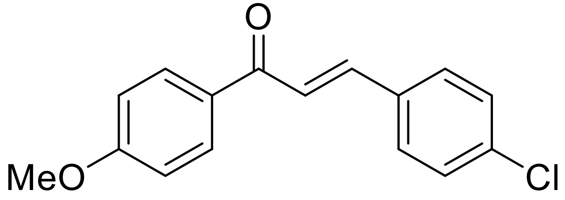

Chemical Formula: $C_{16}H_{13}ClO_2$

Exact Mass: 272.06

m/z: 273 $[M+H]^+$; 295 $[M+Na]^+$

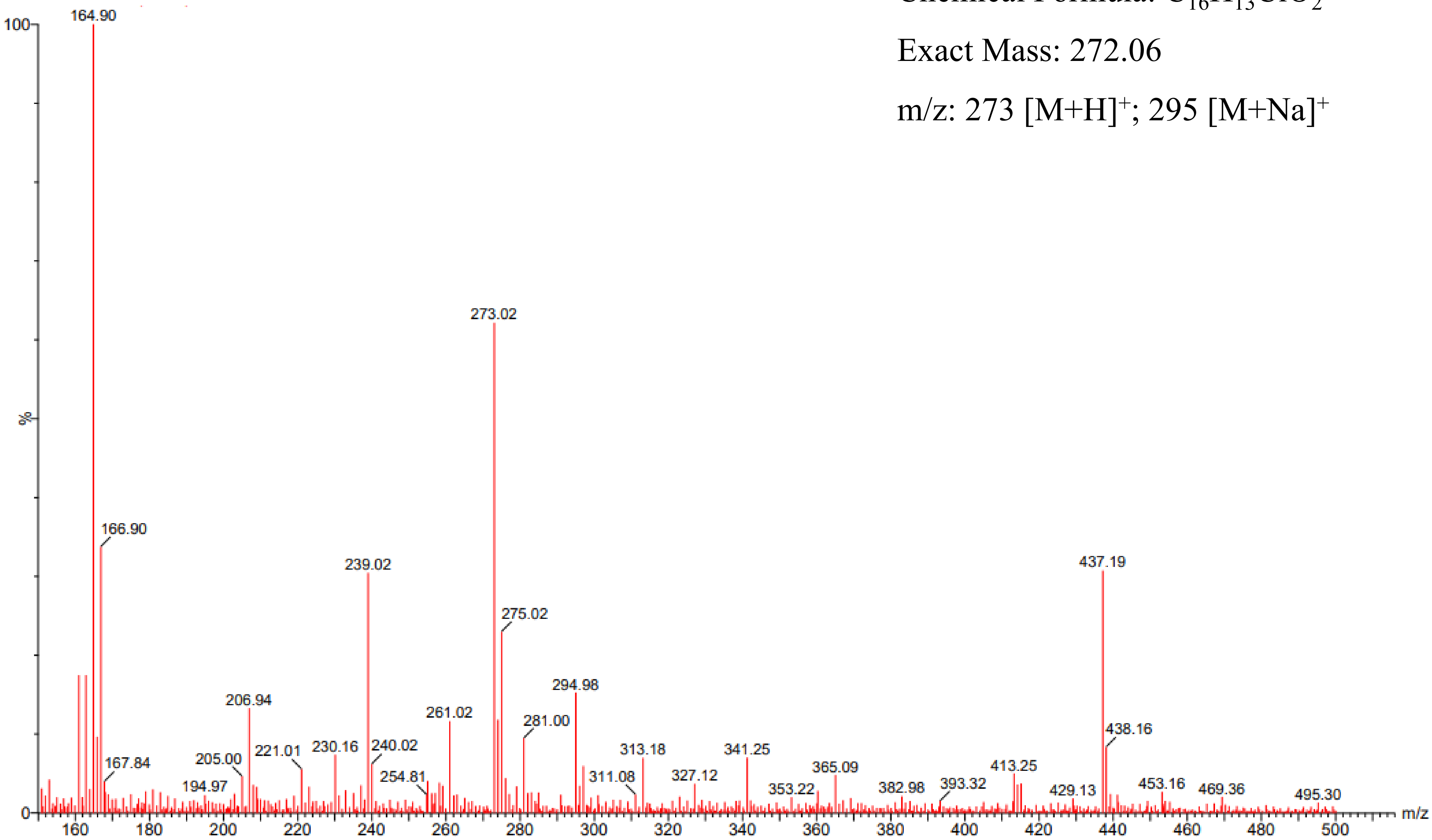

IR Spectrum of **4o**

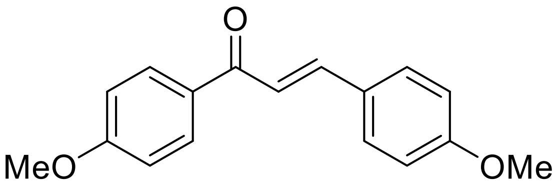

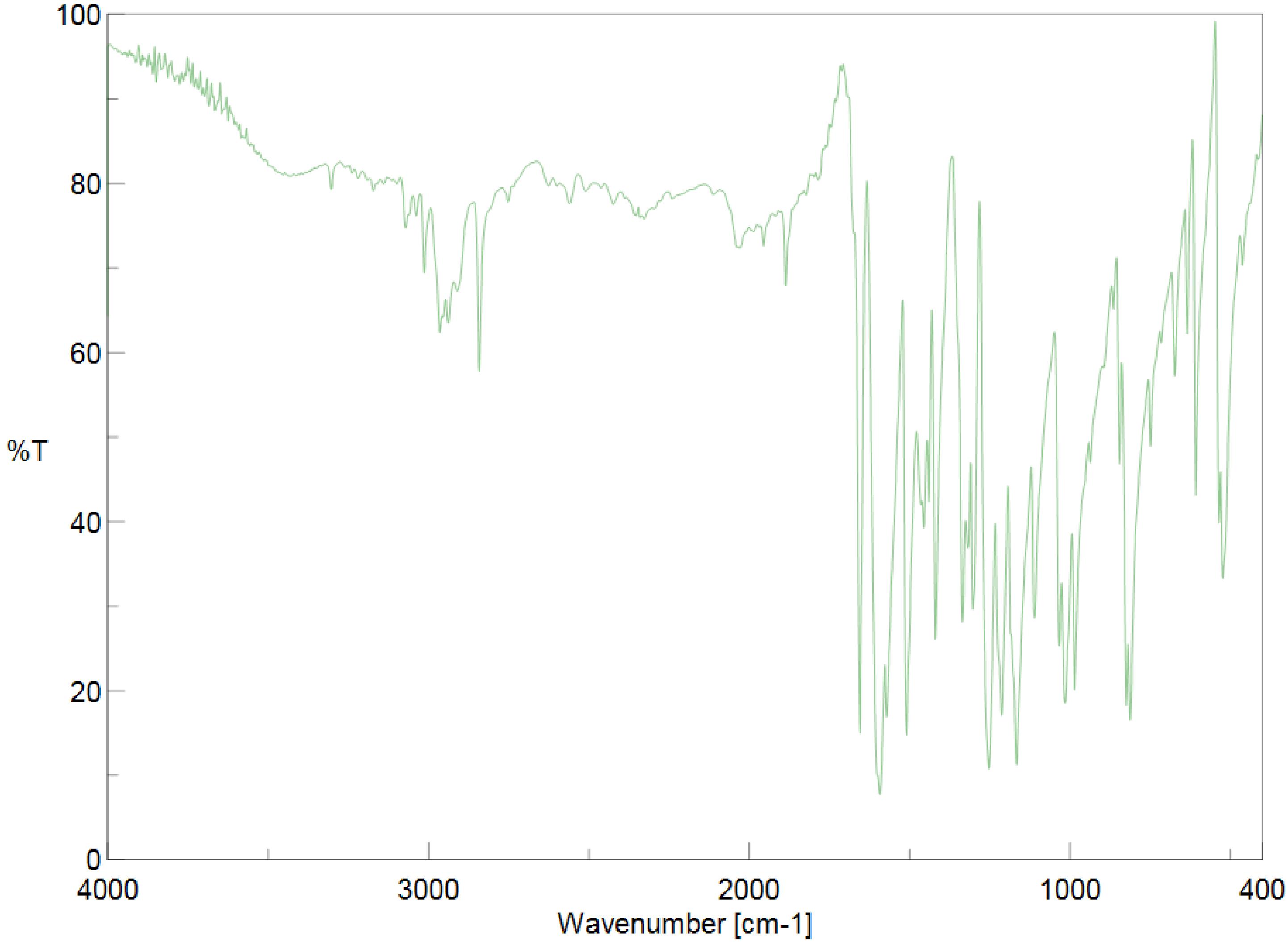

^{1}H-NMR Spectrum of **4o**

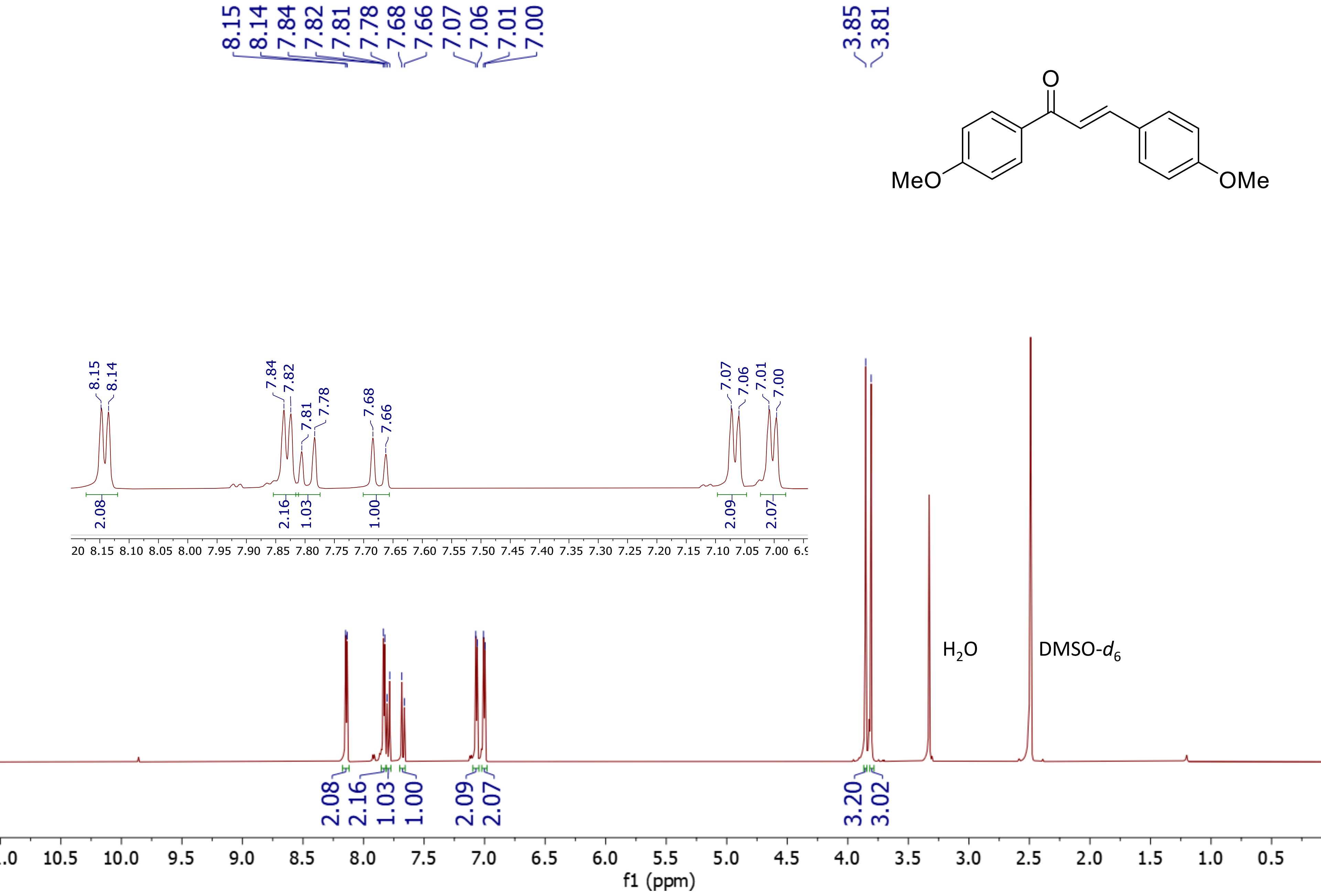

^{13}C-NMR Spectrum of **4o**

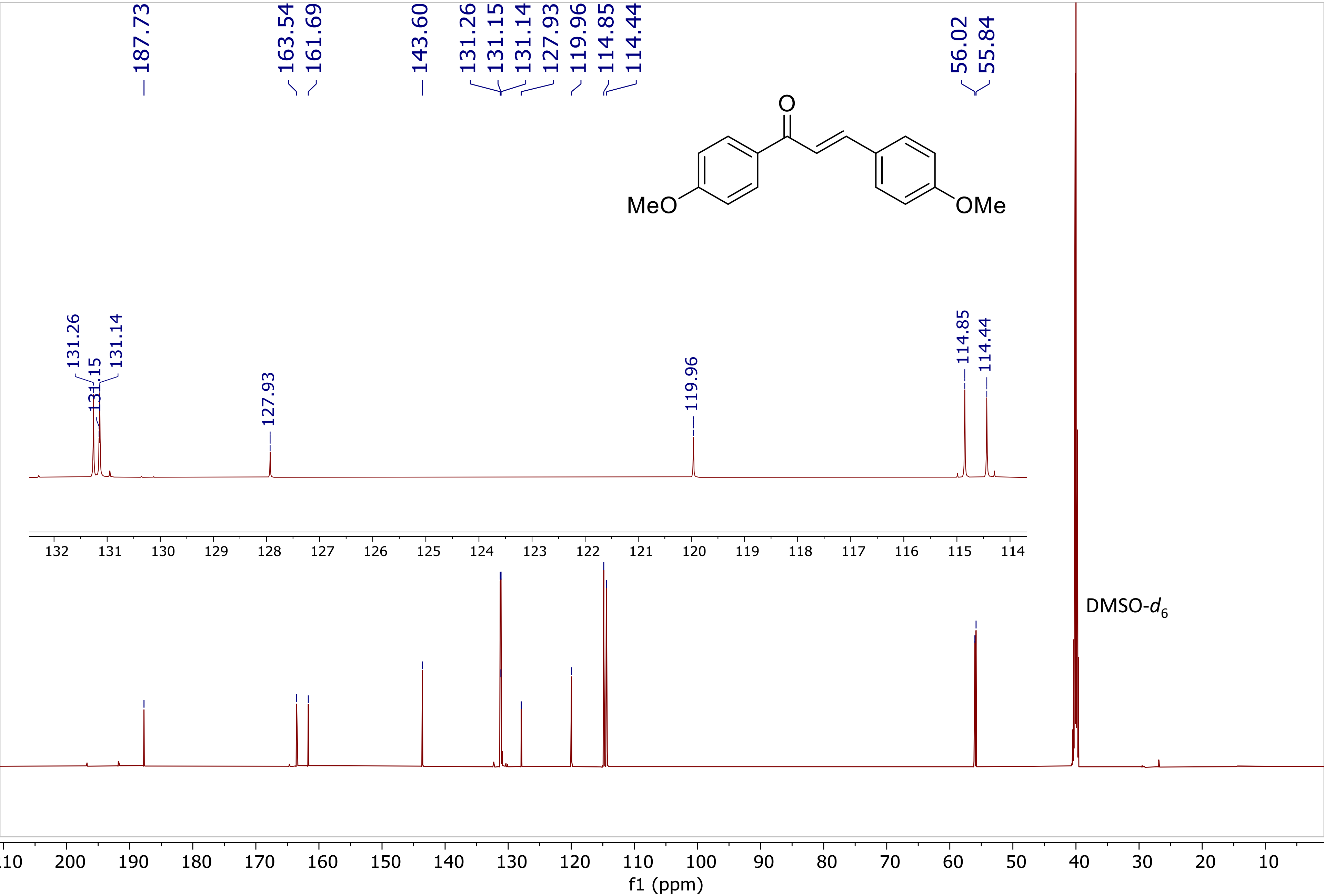

Mass Spectrum of **4o**

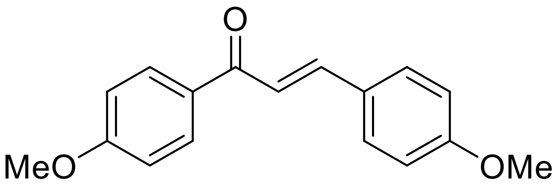

Chemical Formula: $C_{17}H_{16}O_3$

Exact Mass: 268.11

m/z: 269 $[M+H]^+$, 291 $[M+Na]^+$

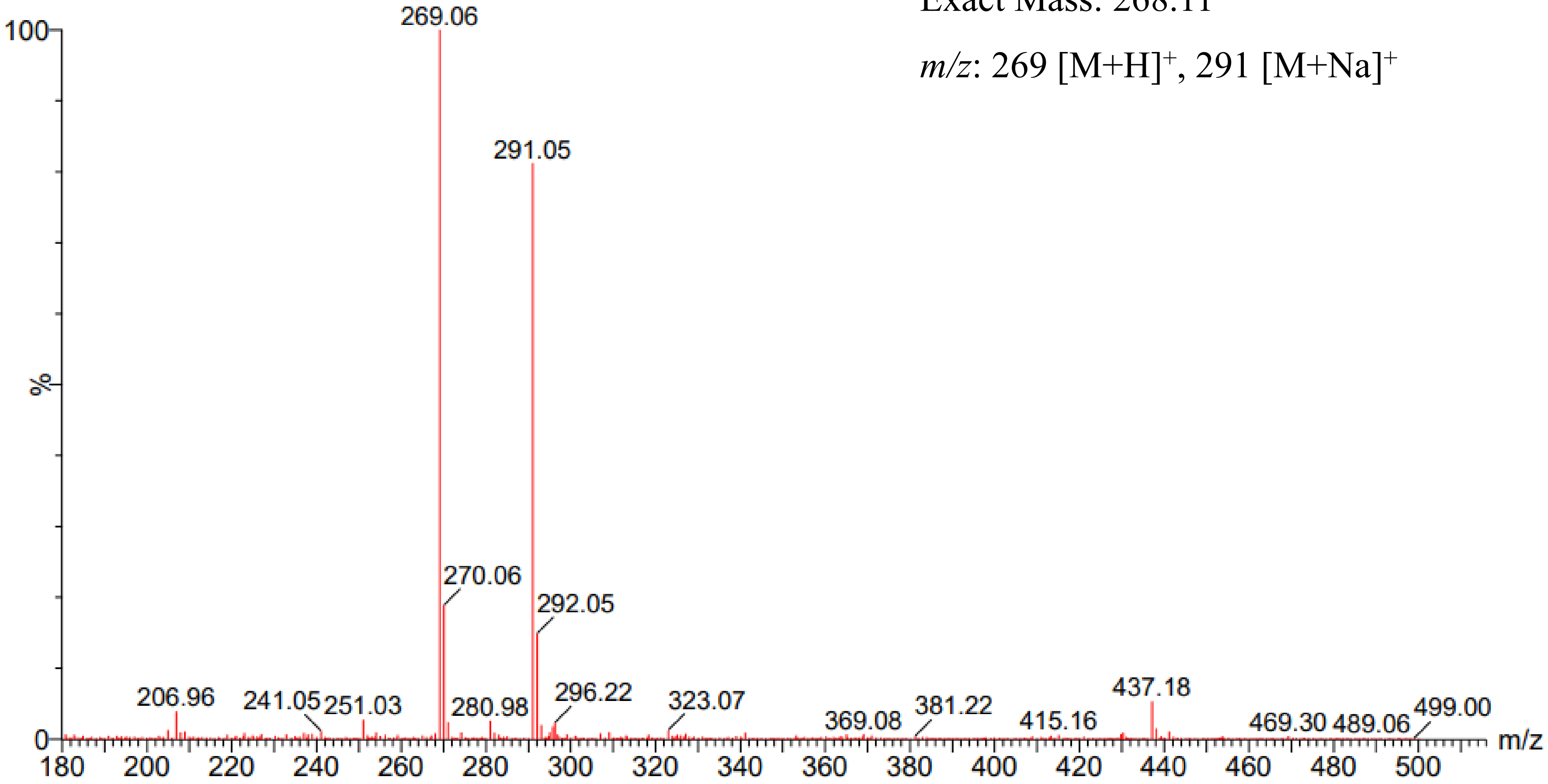

IR Spectrum of **4p**

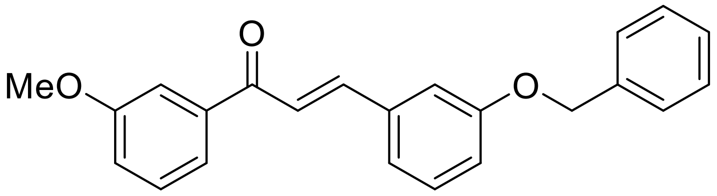

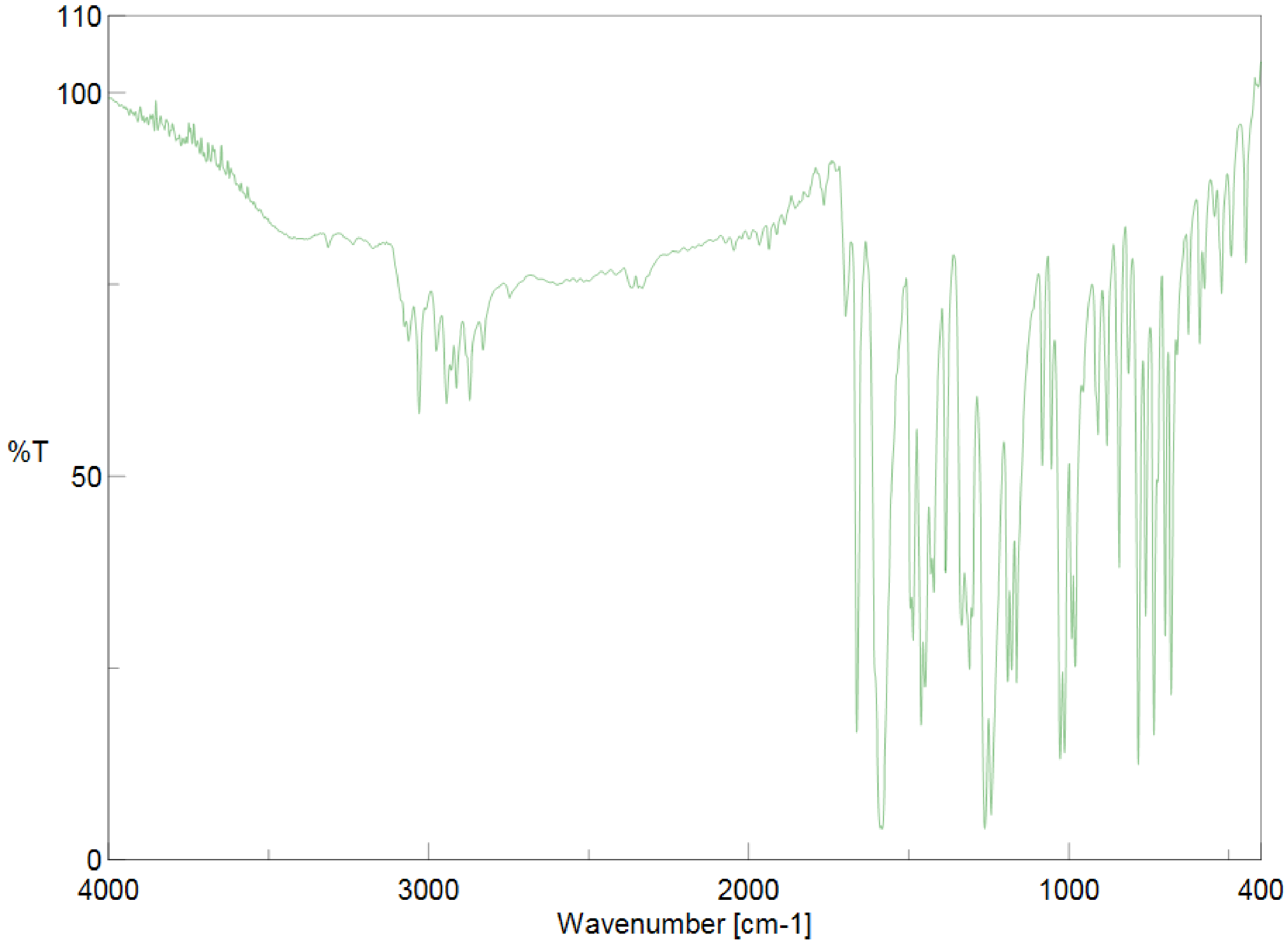

^{1}H-NMR Spectrum of **4p**

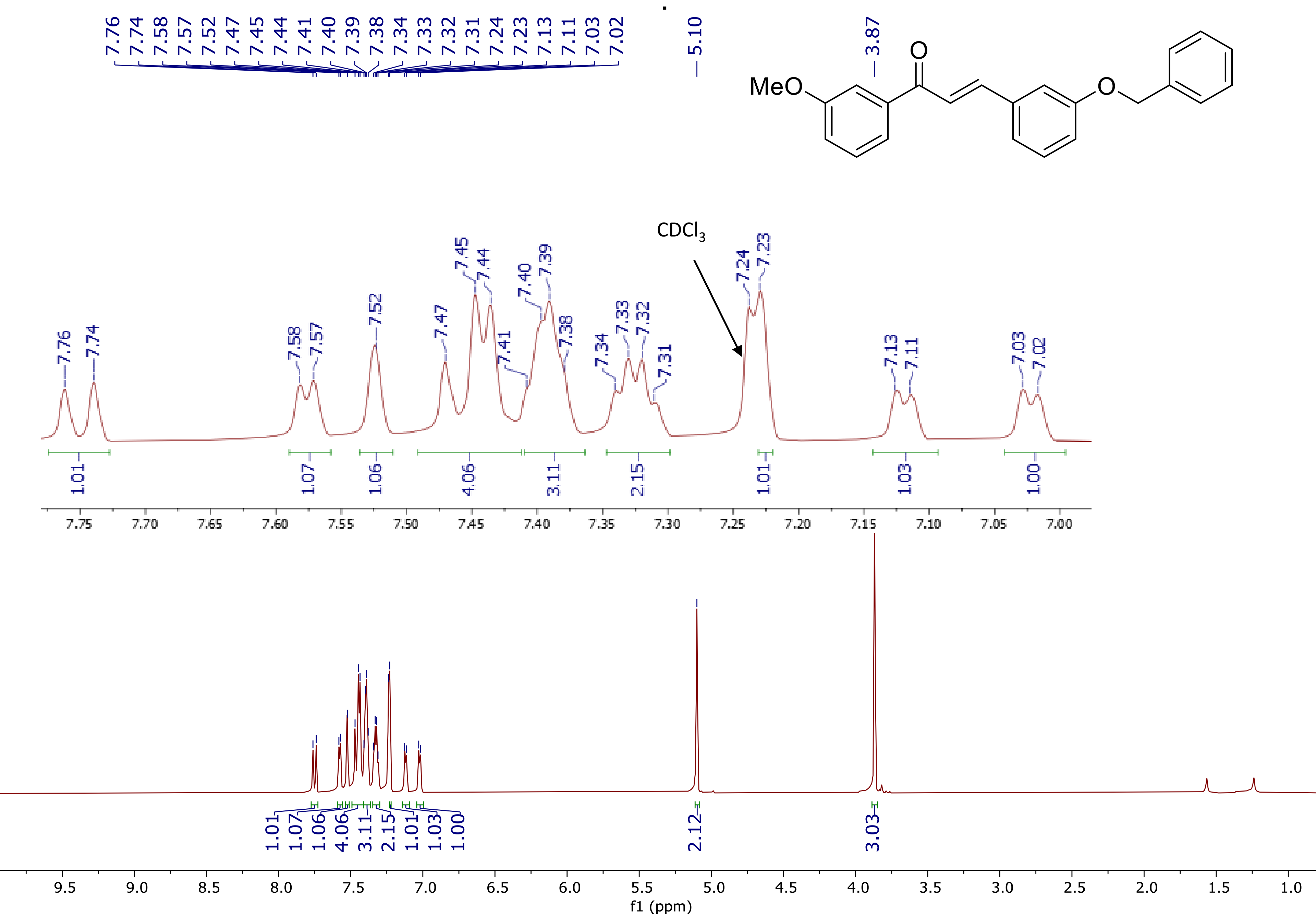

^{13}C-NMR Spectrum of **4p**

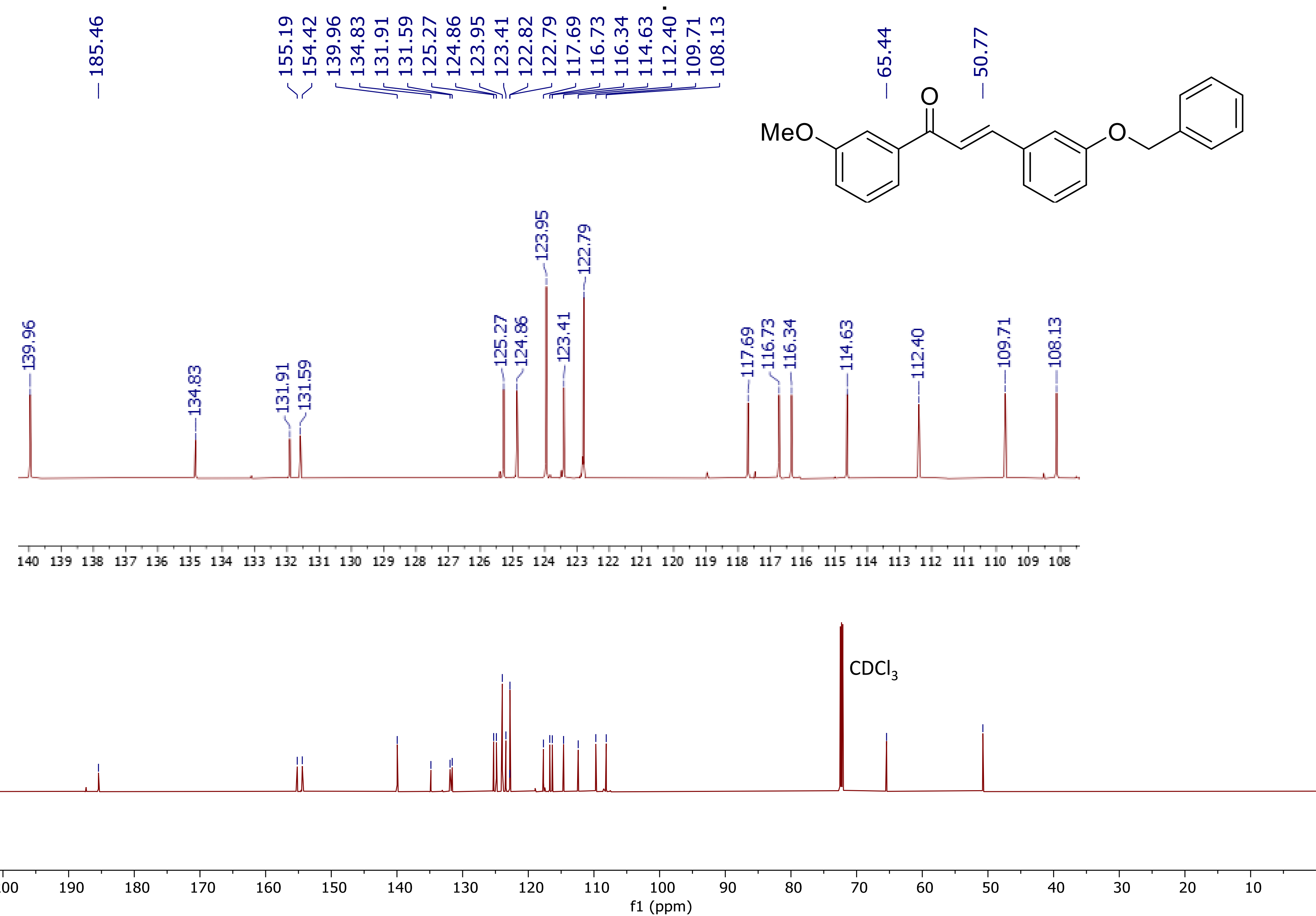

Mass Spectrum of **4p**

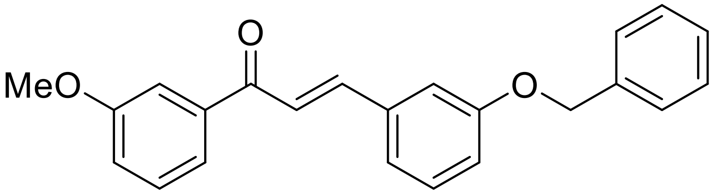

Chemical Formula: $C_{23}H_{20}O_3$

Exact Mass: 344.14

m/z: 345 $[M+H]^+$, 367 $[M+Na]^+$

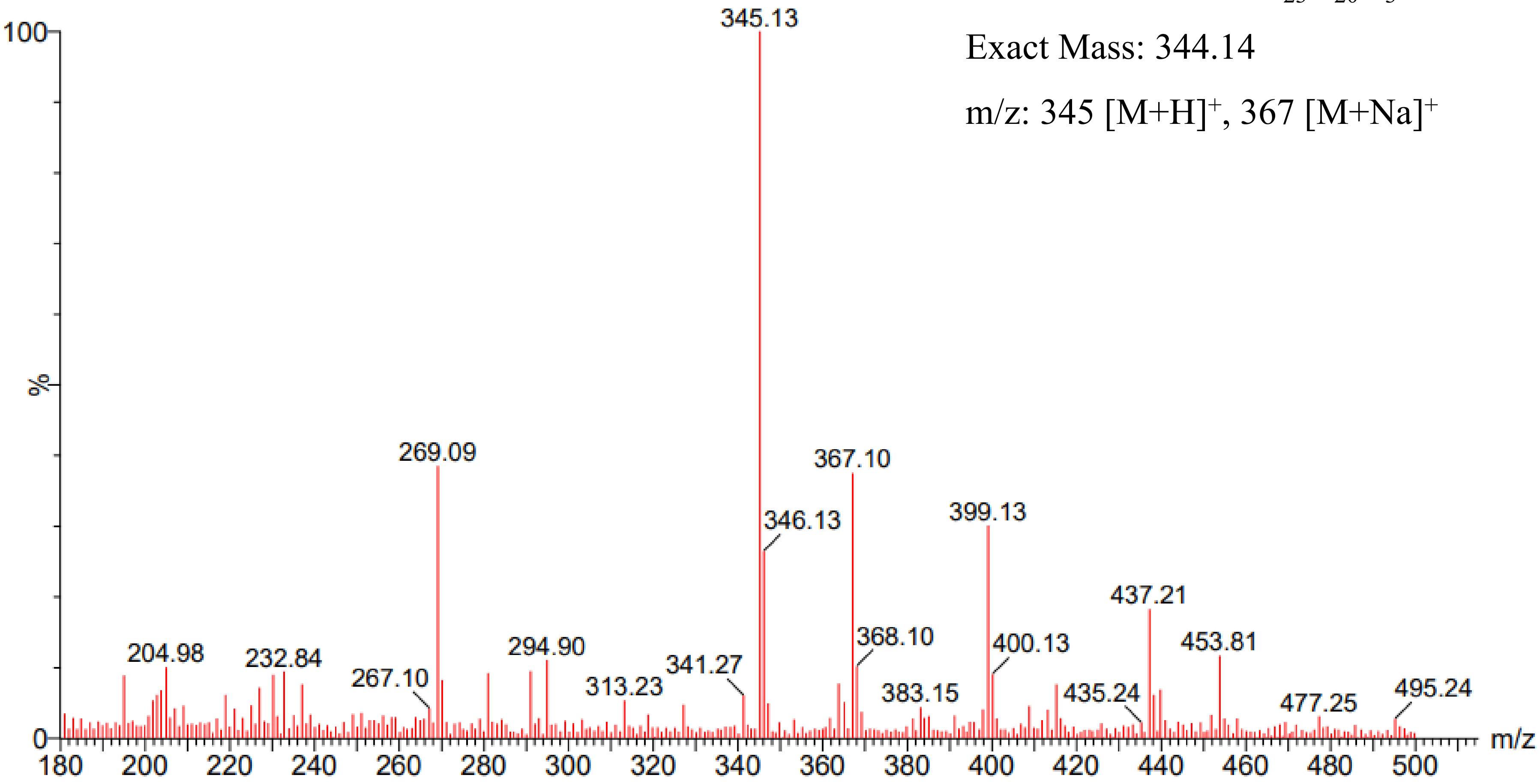

IR Spectrum of **4q**

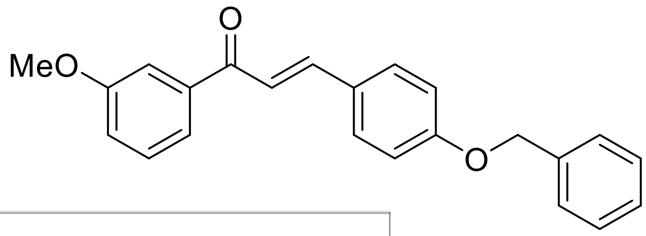

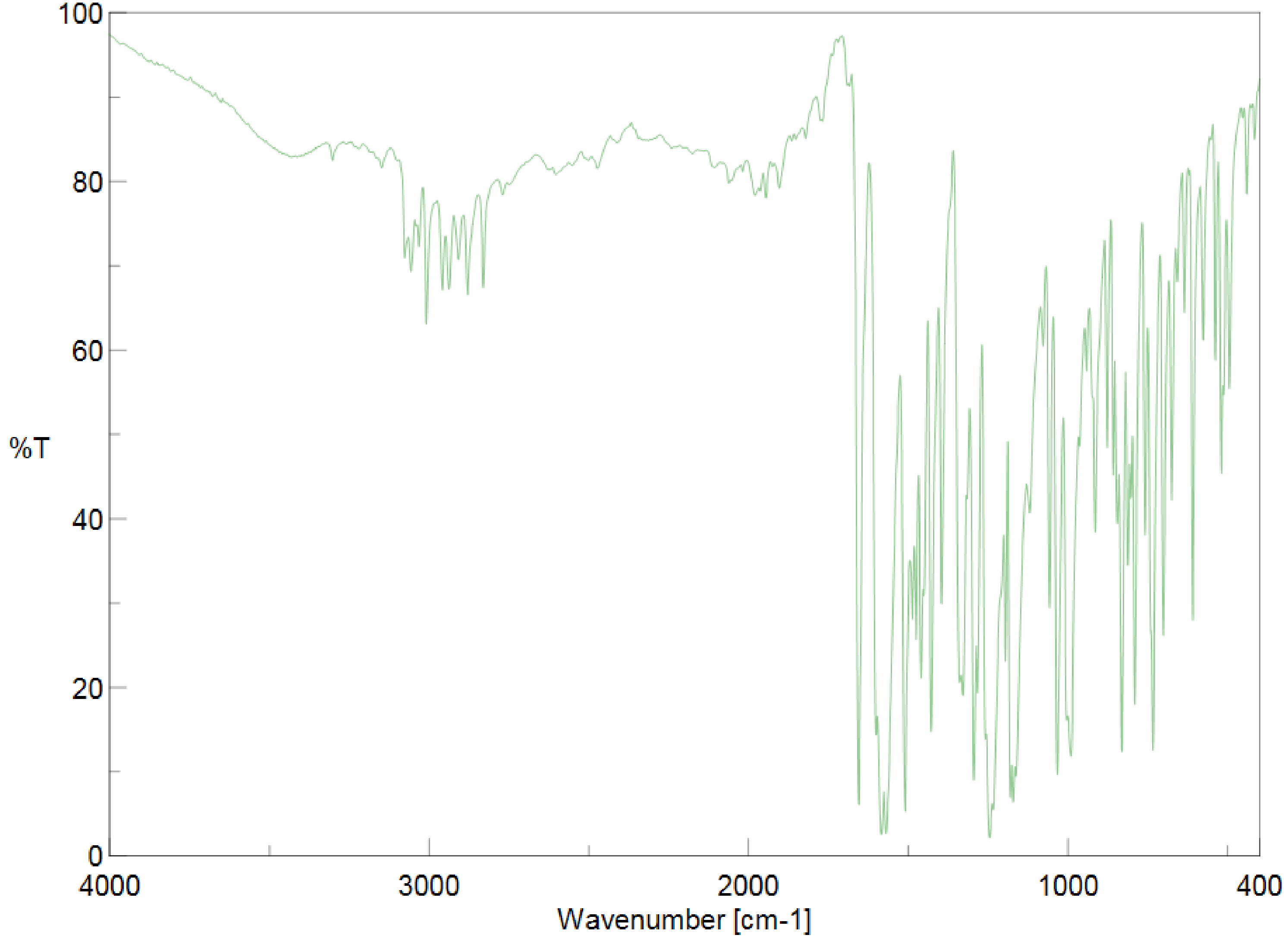

^{1}H-NMR Spectrum of **4q**

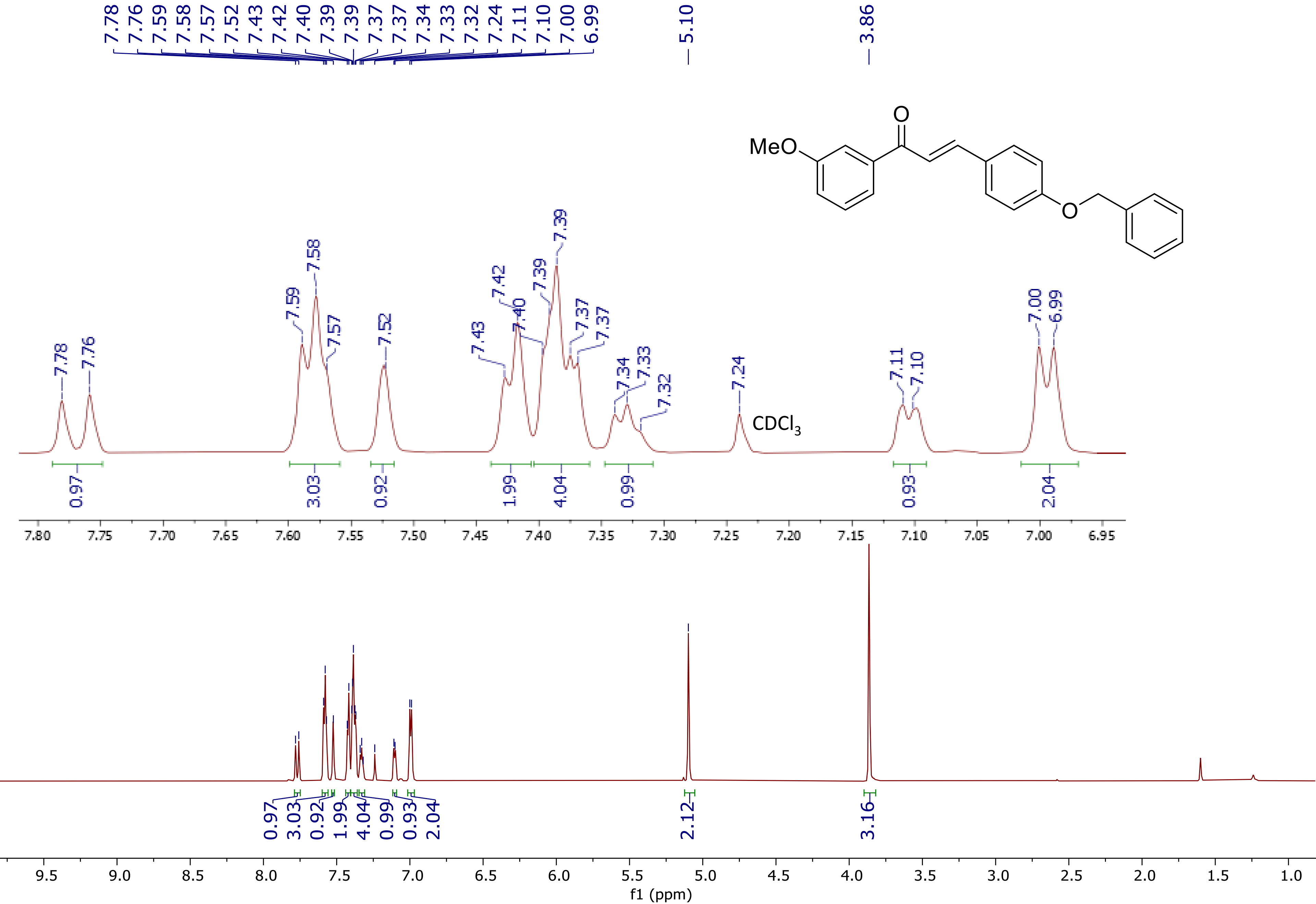

^{13}C-NMR Spectrum of **4q**

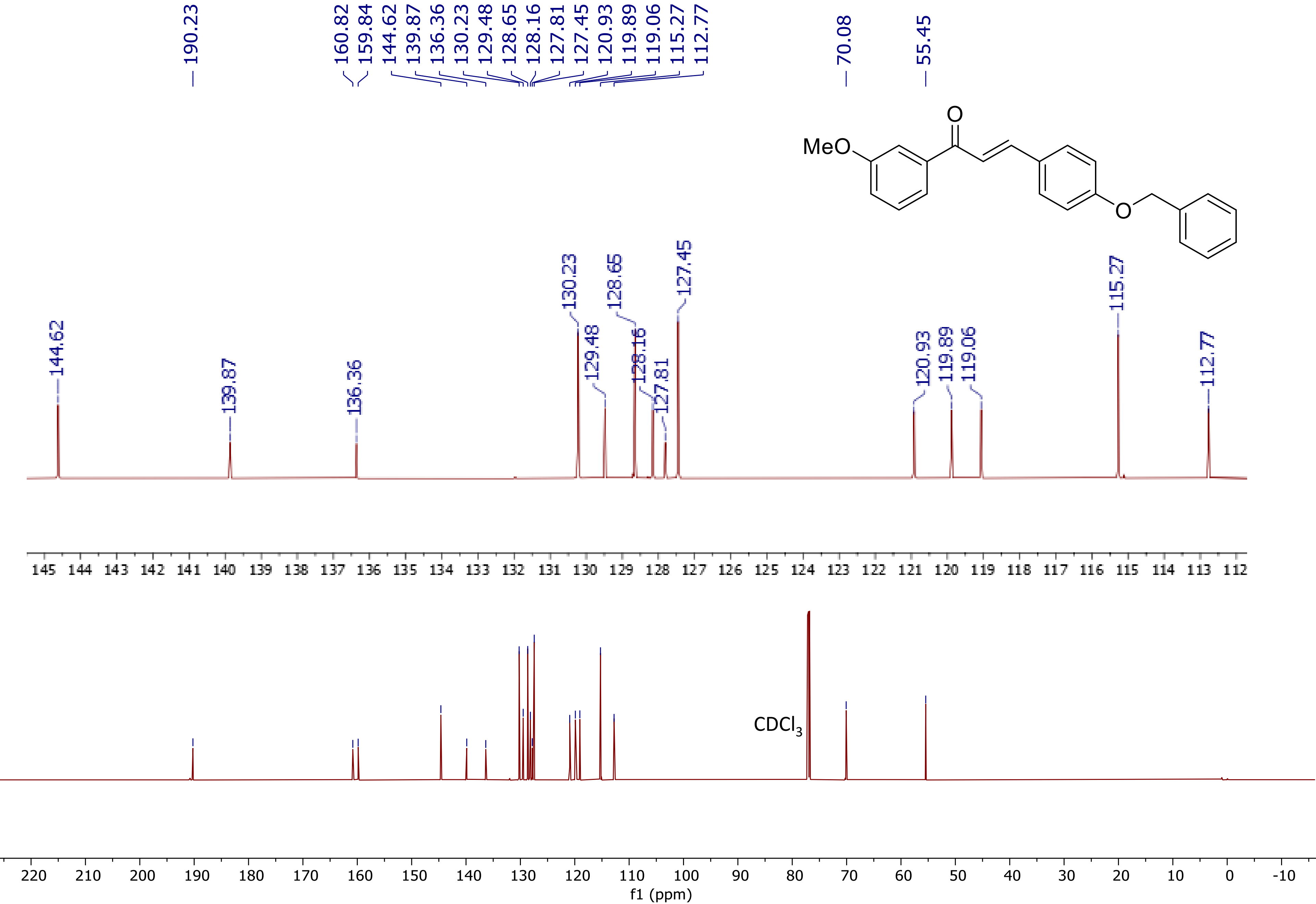

Mass Spectrum of **4q**

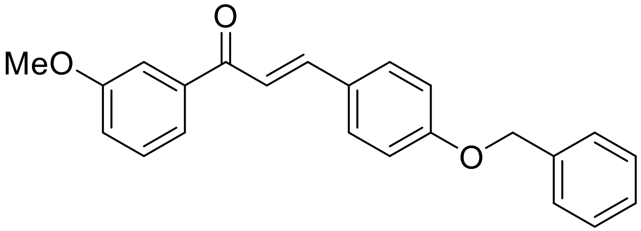

Chemical Formula: $C_{23}H_{20}O_3$

Exact Mass: 344.14

m/z: 345 $[M+H]^+$; 367 $[M+Na]^+$

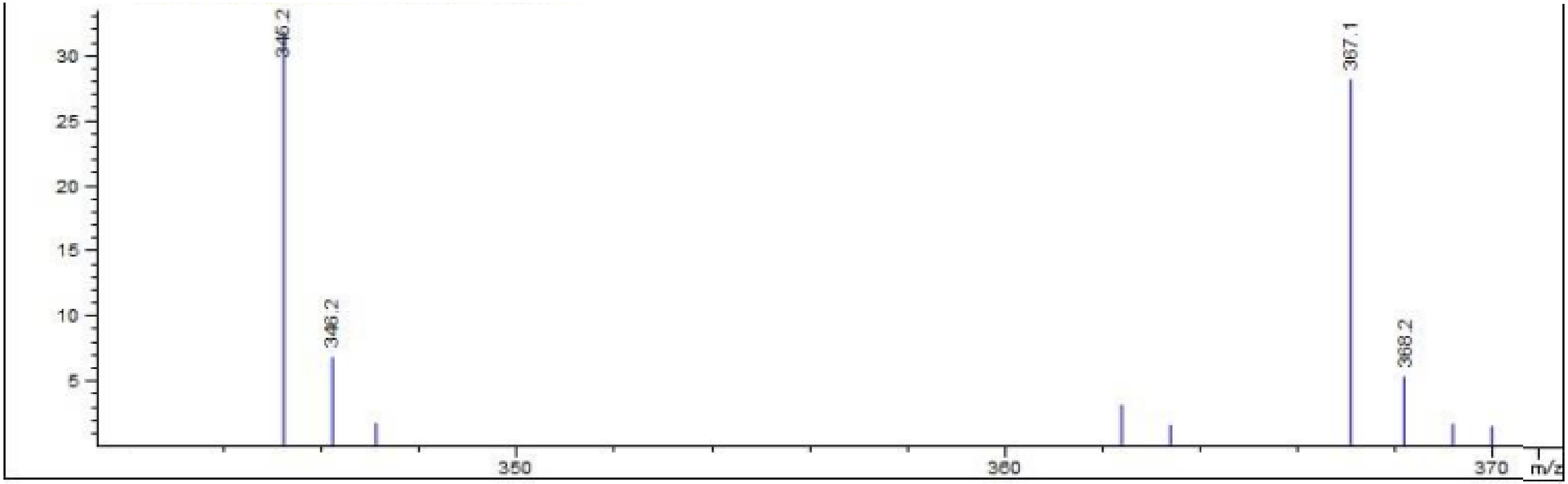

IR Spectrum of **4r**

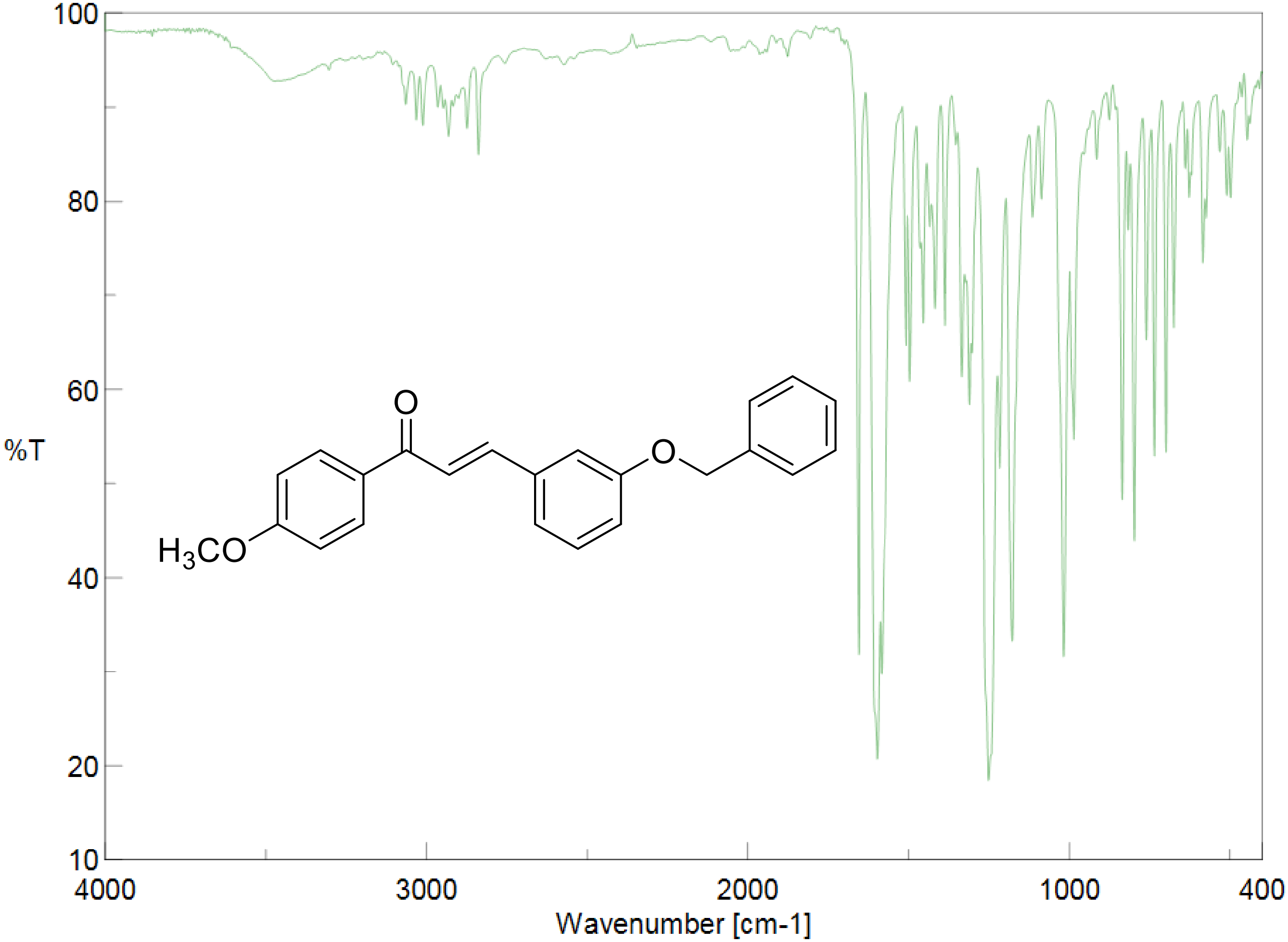

^{1}H-NMR Spectrum of **4r**

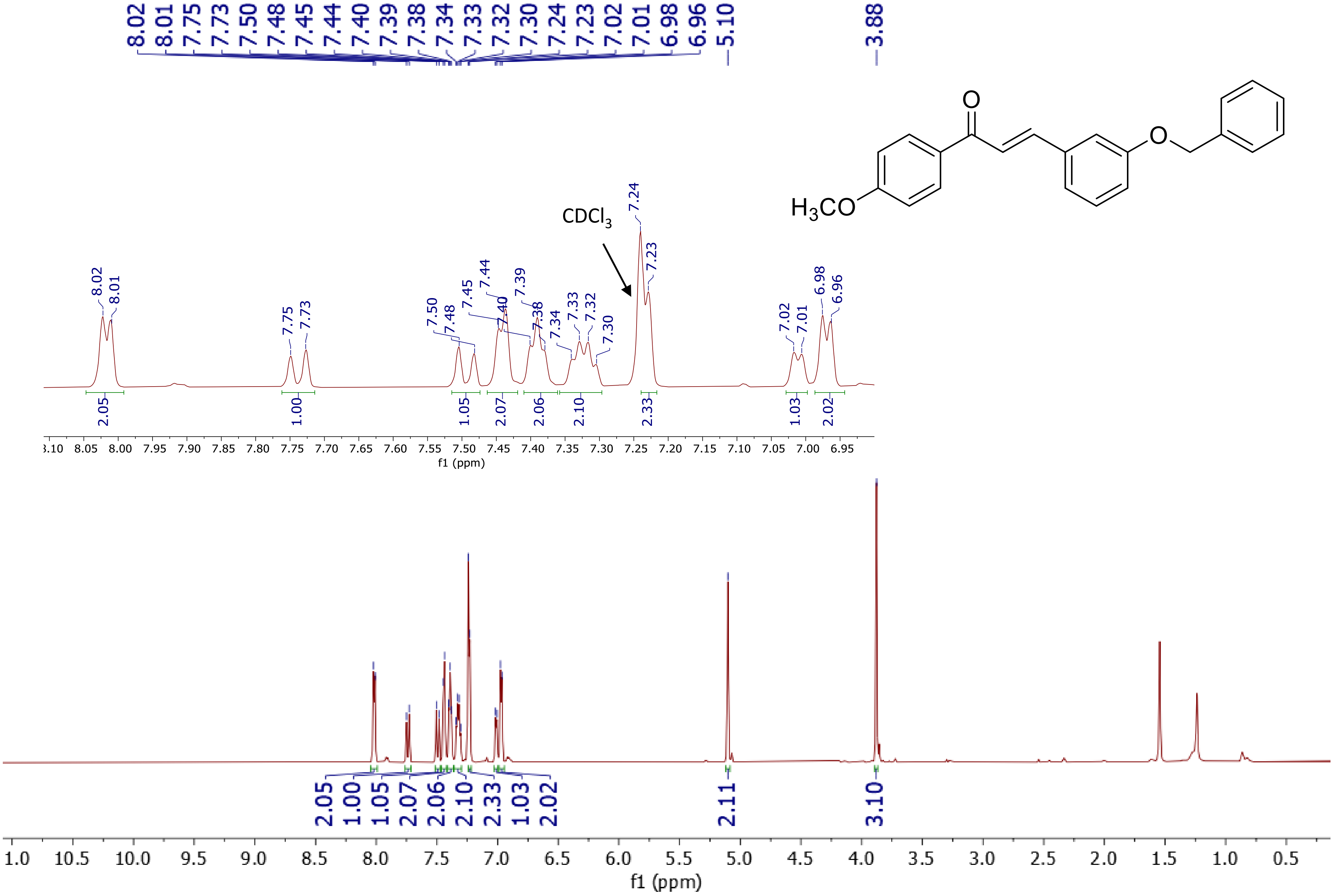

^{13}C-NMR Spectrum of **4r**

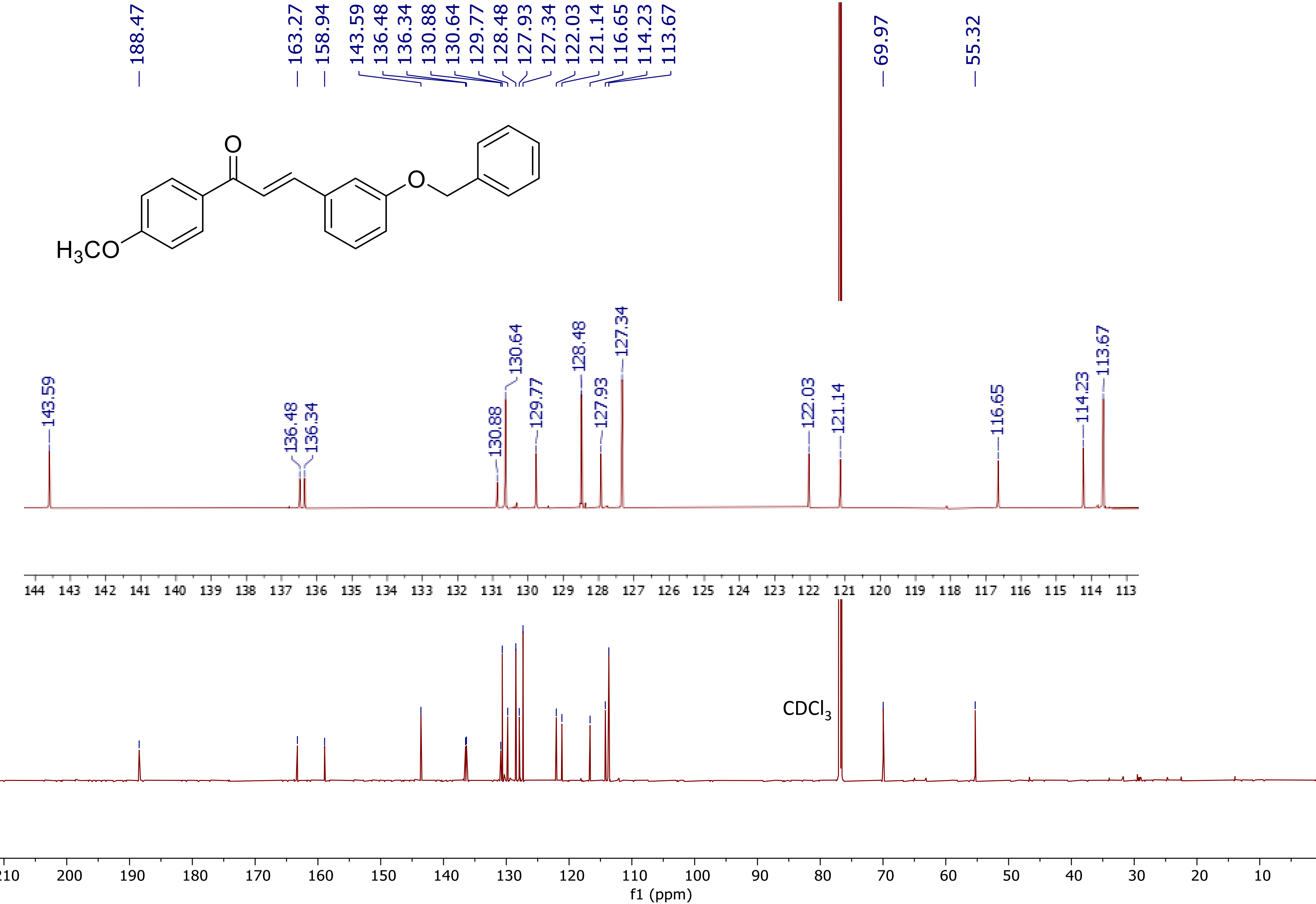

Mass Spectrum of **4r**

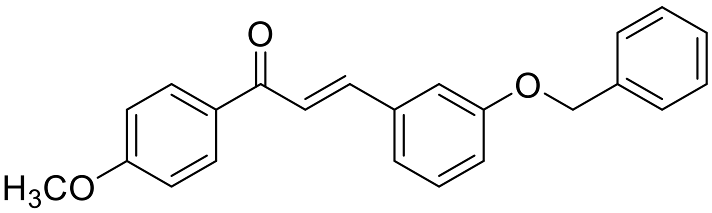

Chemical Formula: $C_{23}H_{20}O_3$

Exact Mass: 344.14

m/z: 345 $[M+H]^+$

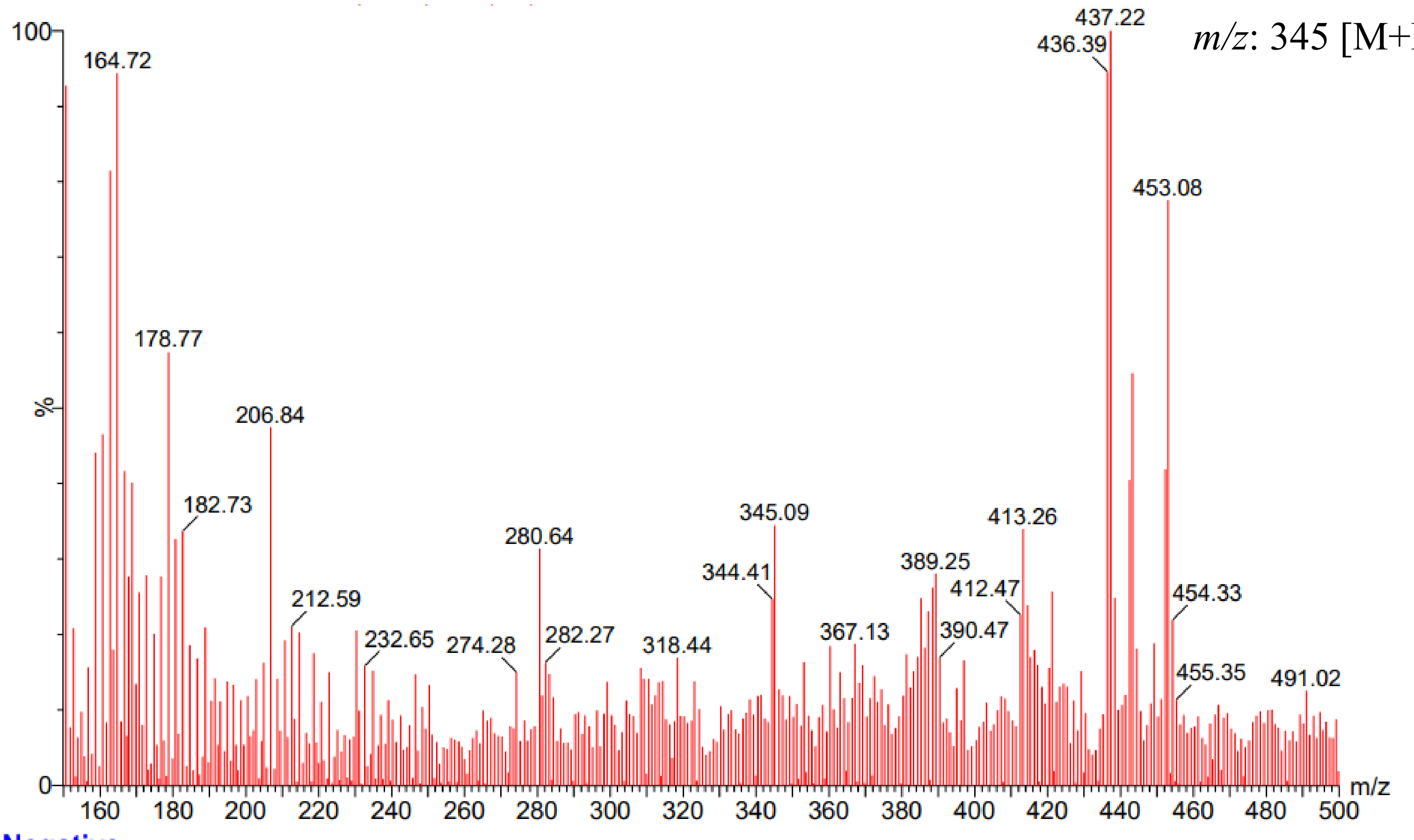

IR Spectrum of **4s**

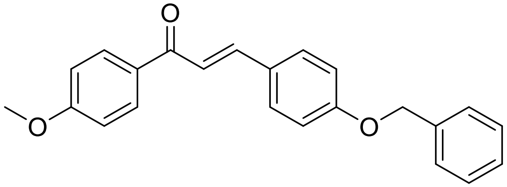

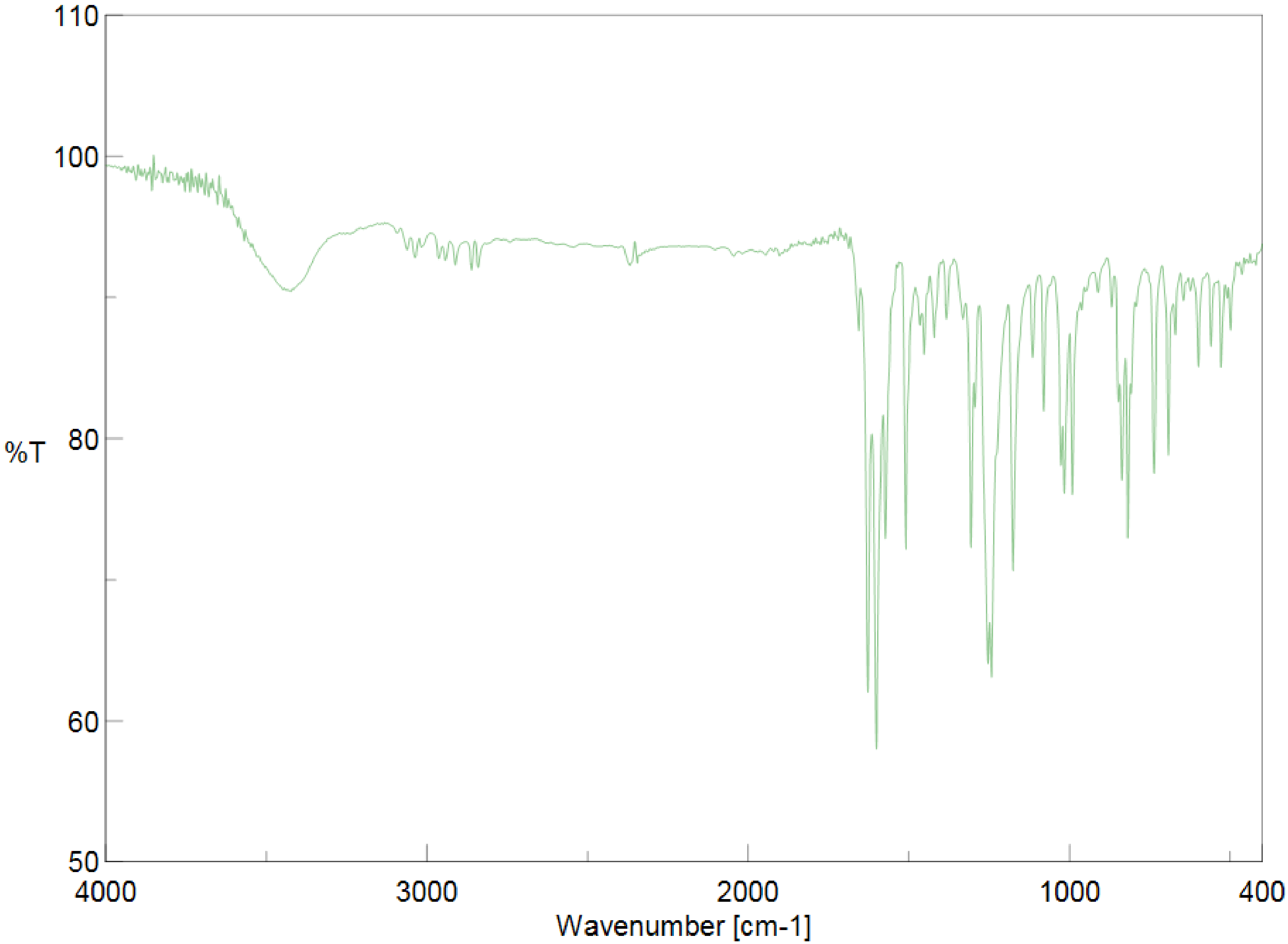

^{1}H-NMR Spectrum of **4s**

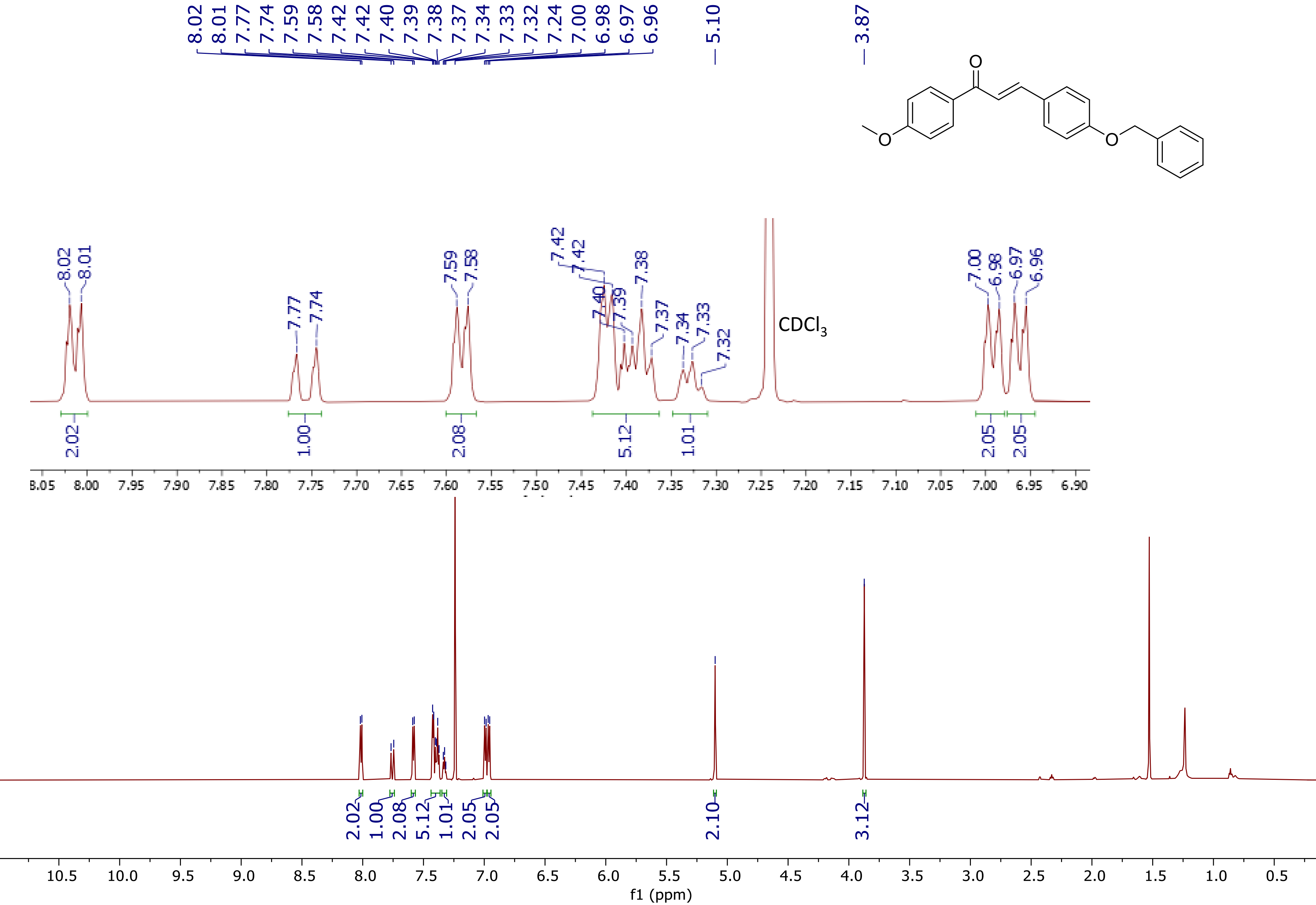

^{13}C-NMR Spectrum of **4s**

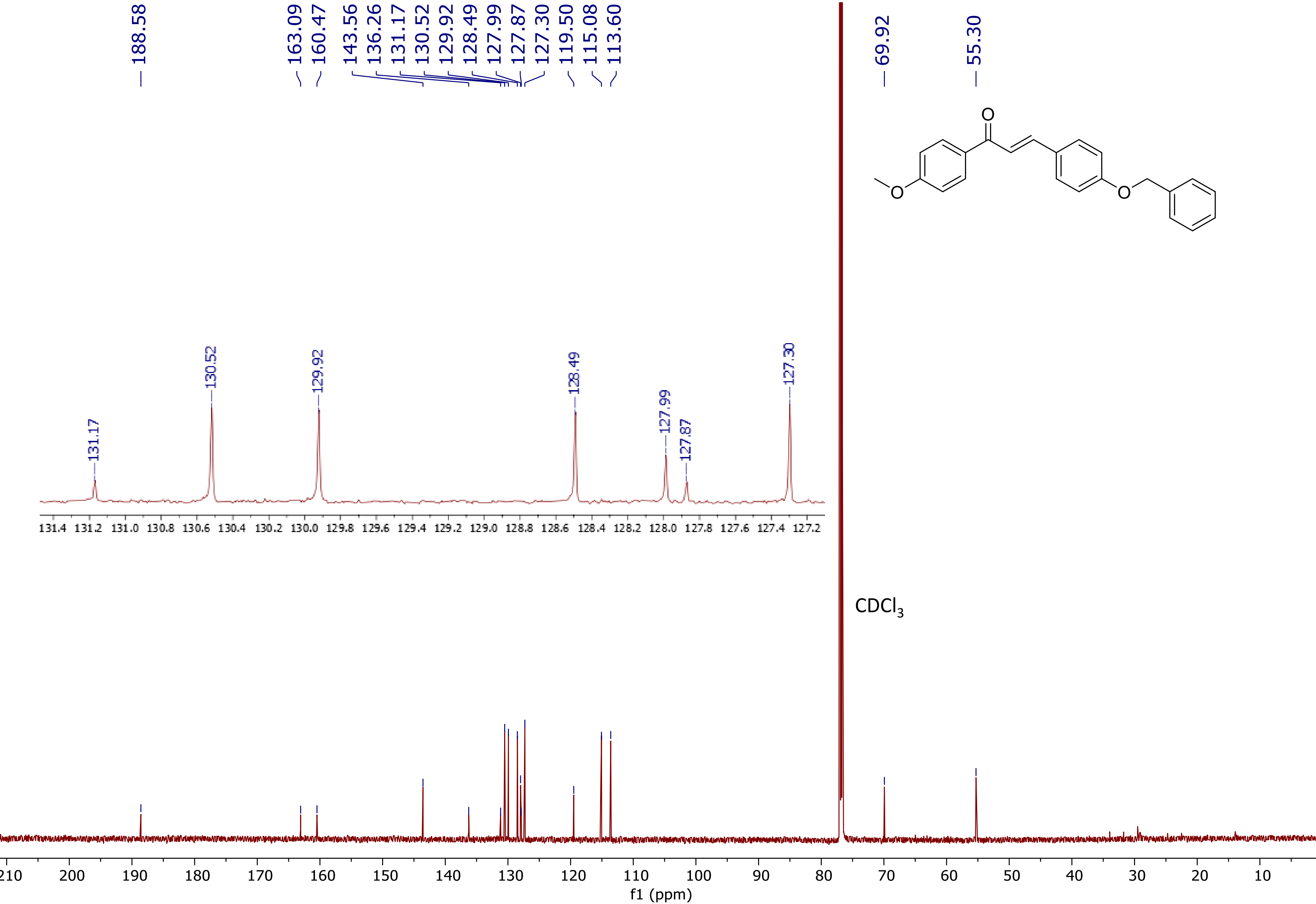

Mass Spectrum of **4s**

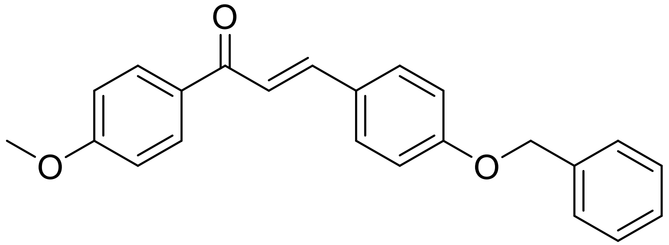

Chemical Formula: $C_{23}H_{20}O_3$

Exact Mass: 344.14

m/z: 345 $[M+H]^+$; 367 $[M+Na]^+$

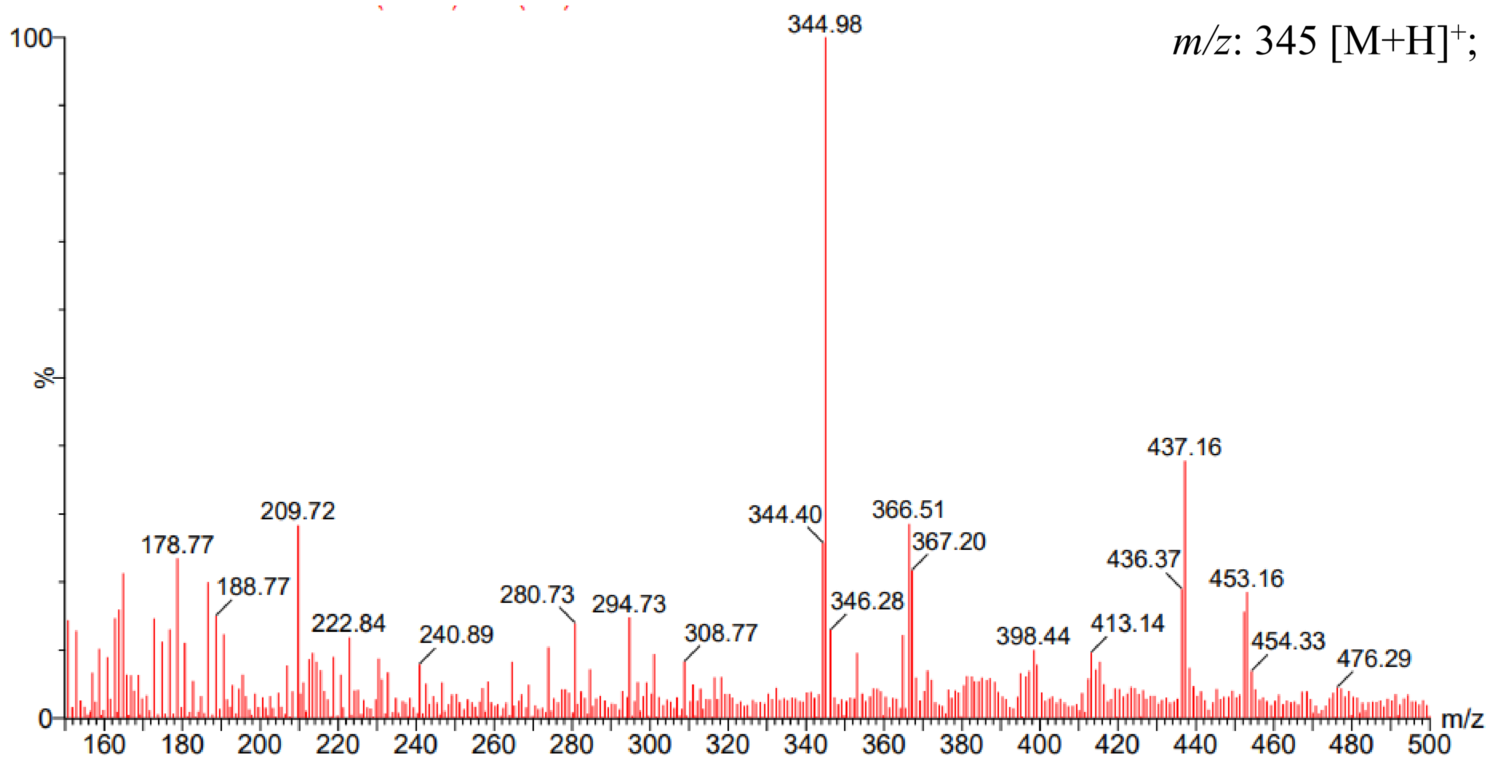

IR Spectrum of **4t**

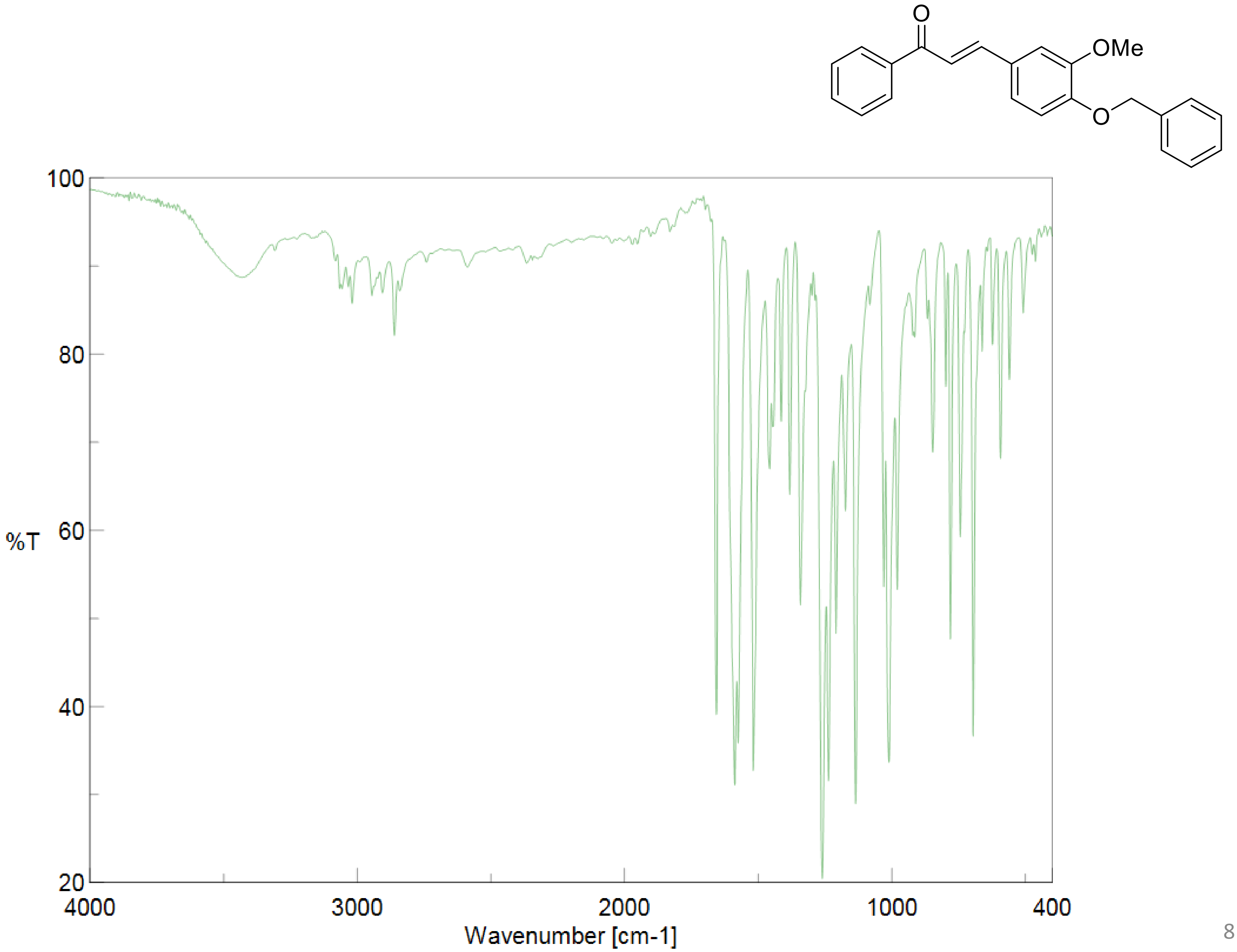

^{1}H-NMR Spectrum of **4t**

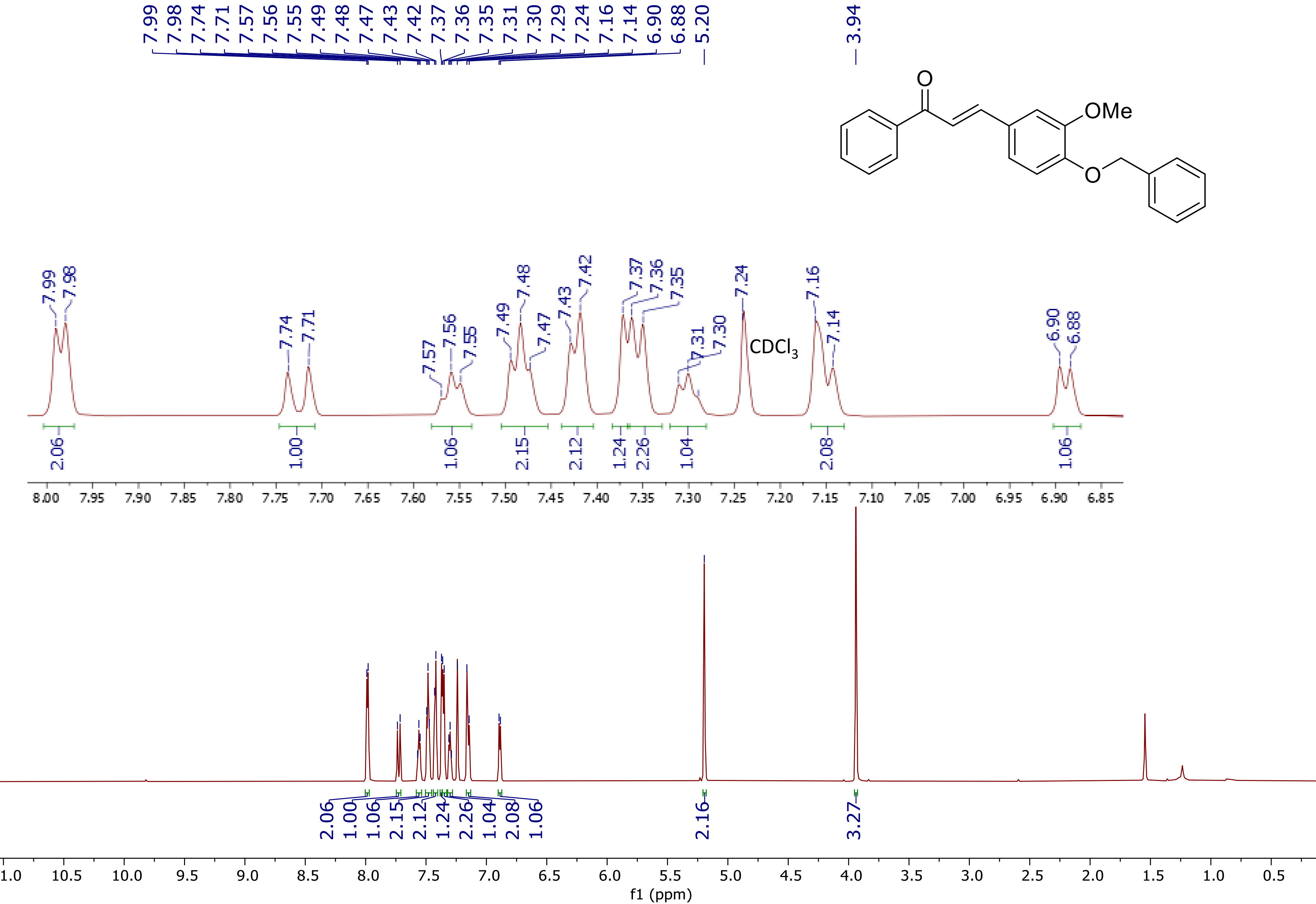

^{13}C-NMR Spectrum of **4t**

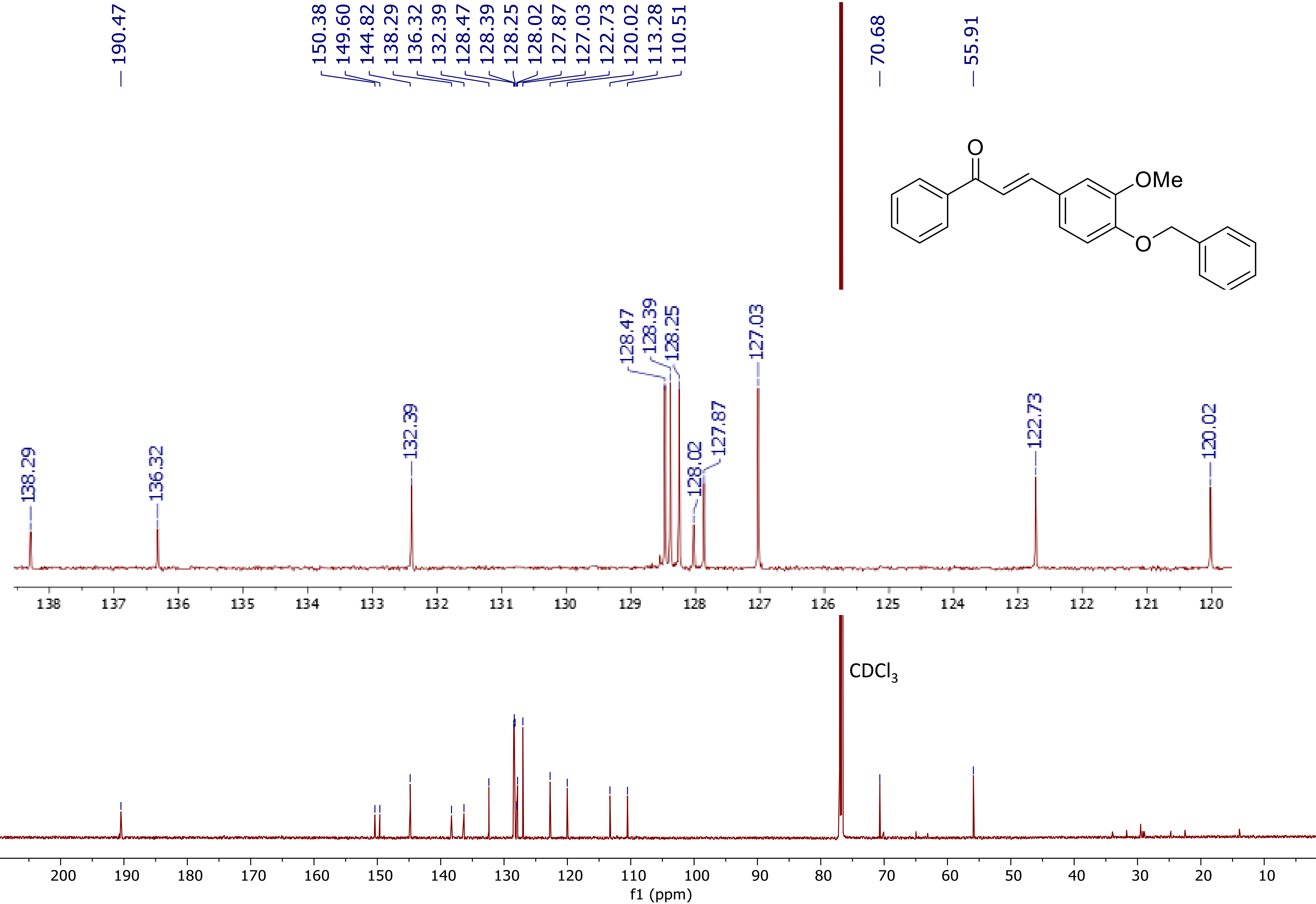

Mass Spectrum of **4t**

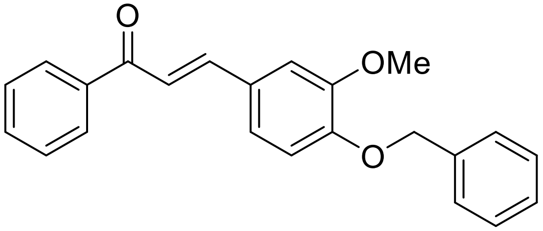

Chemical Formula: $C_{23}H_{20}O_3$

Exact Mass: 344.14

m/z: 345 $[M+H]^+$; 367 $[M+Na]^+$

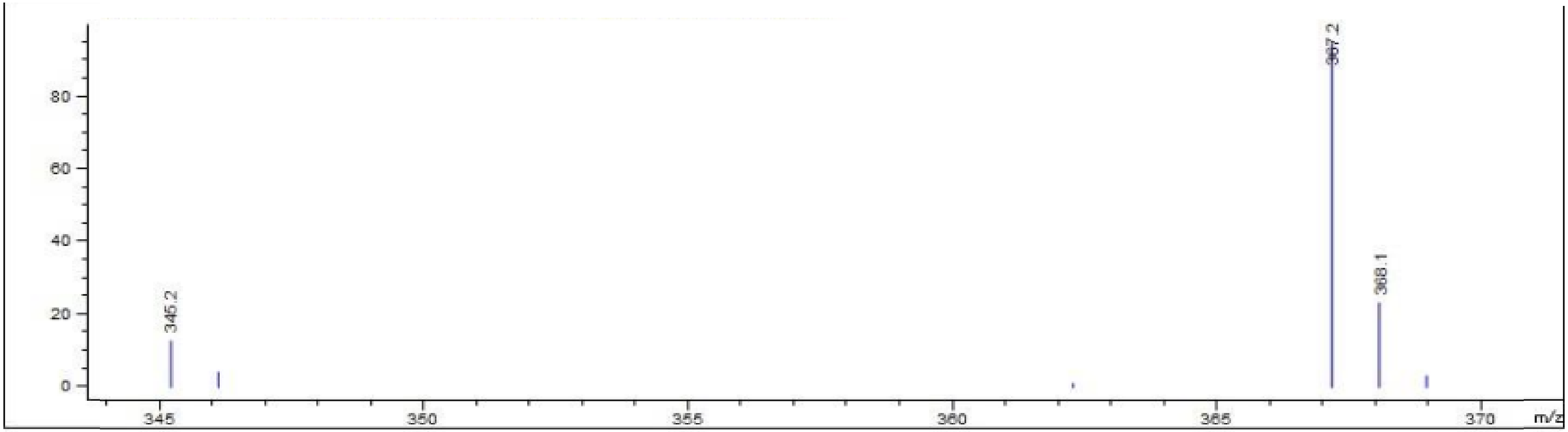

IR Spectrum of **4u**

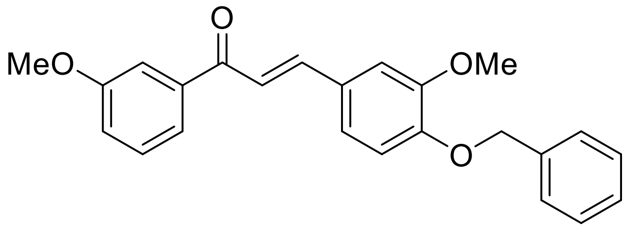

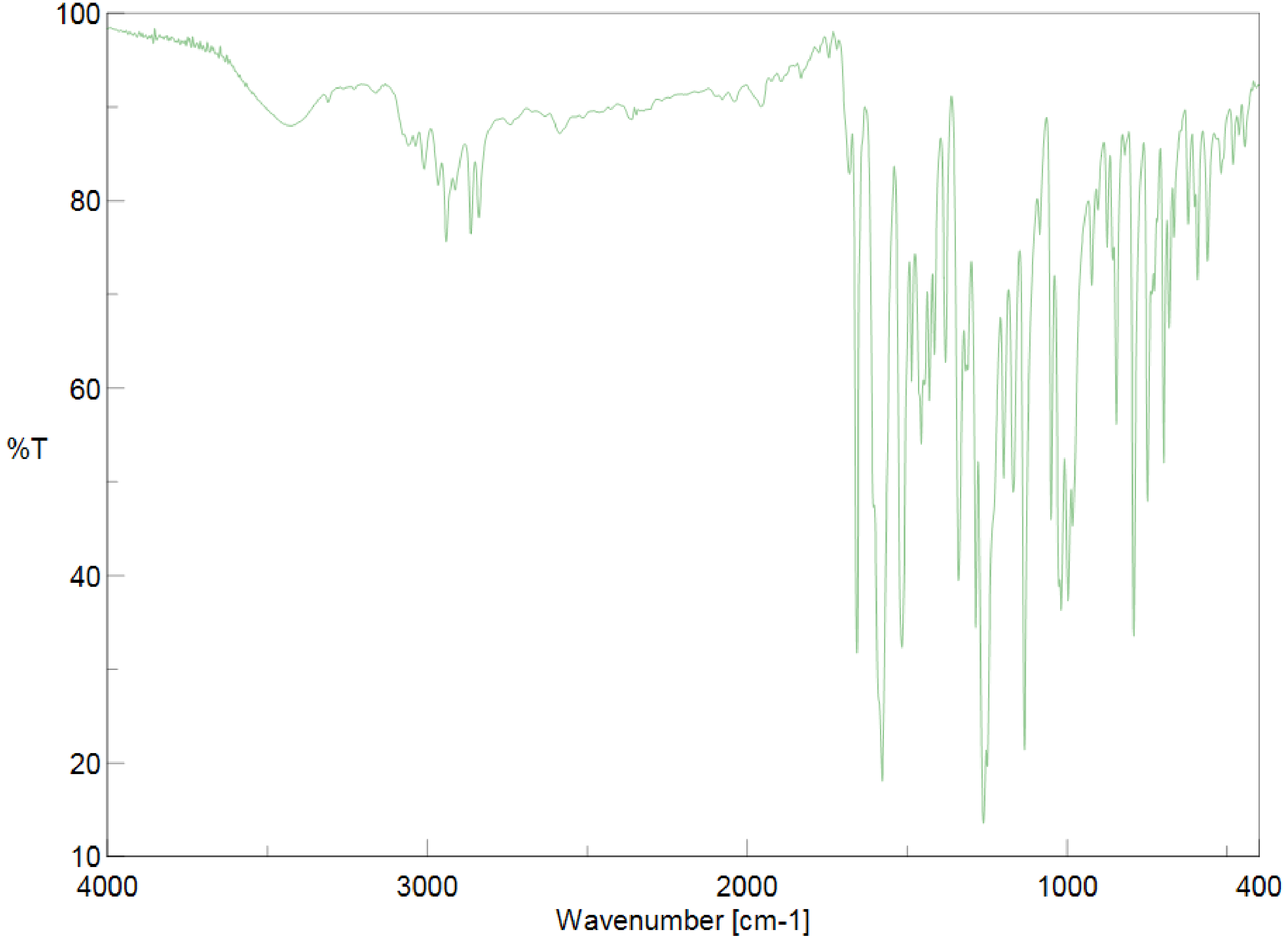

^{1}H-NMR Spectrum of **4u**

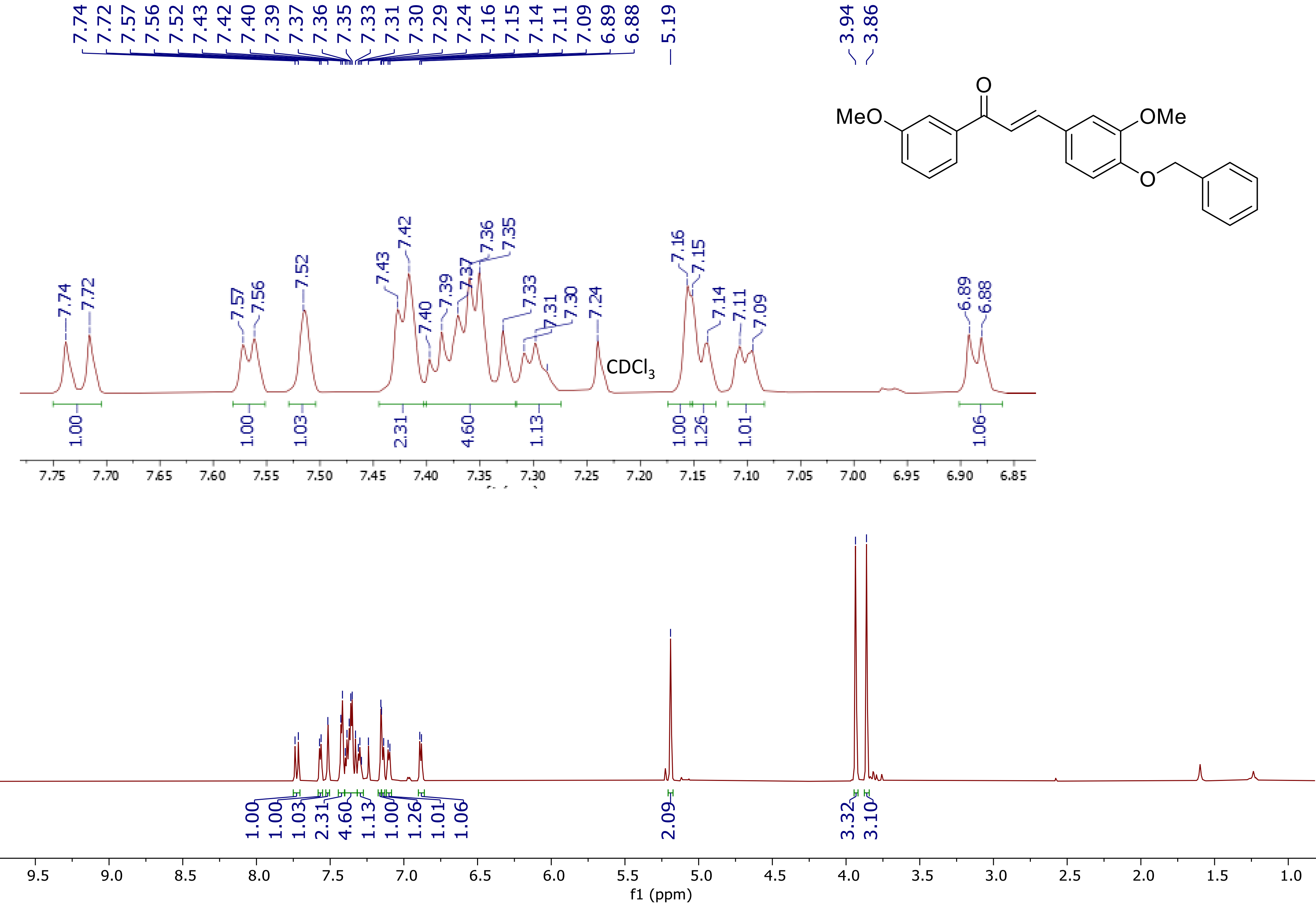

^{13}C-NMR Spectrum of **4u**

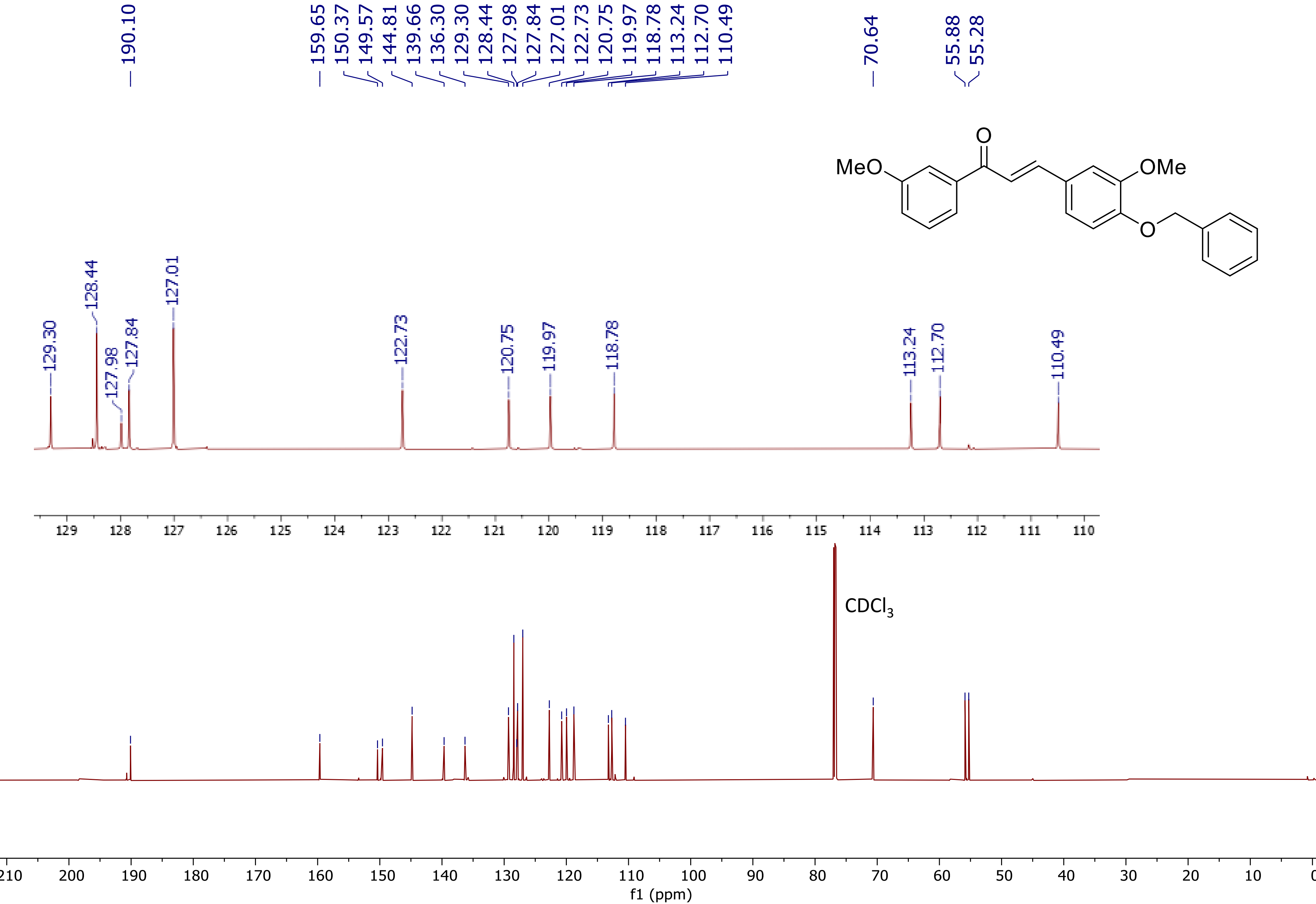

Mass Spectrum of **4u**

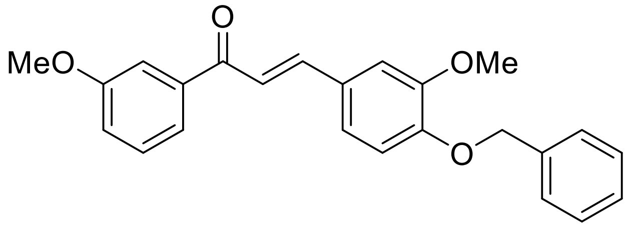

Chemical Formula: $C_{24}H_{22}O_4$

Exact Mass: 374.15

m/z: 375 $[M+H]^+$; 397 $[M+Na]^+$

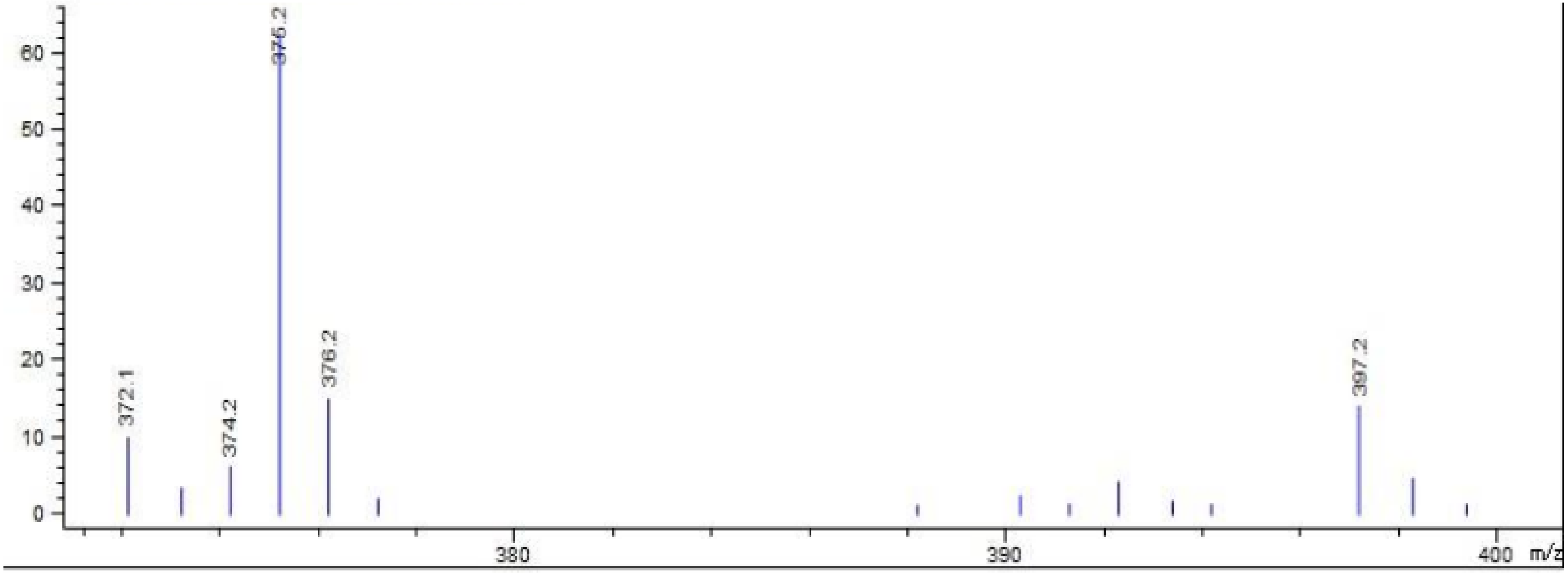

IR Spectrum of **4v**

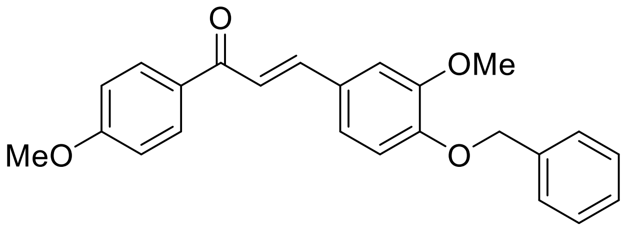

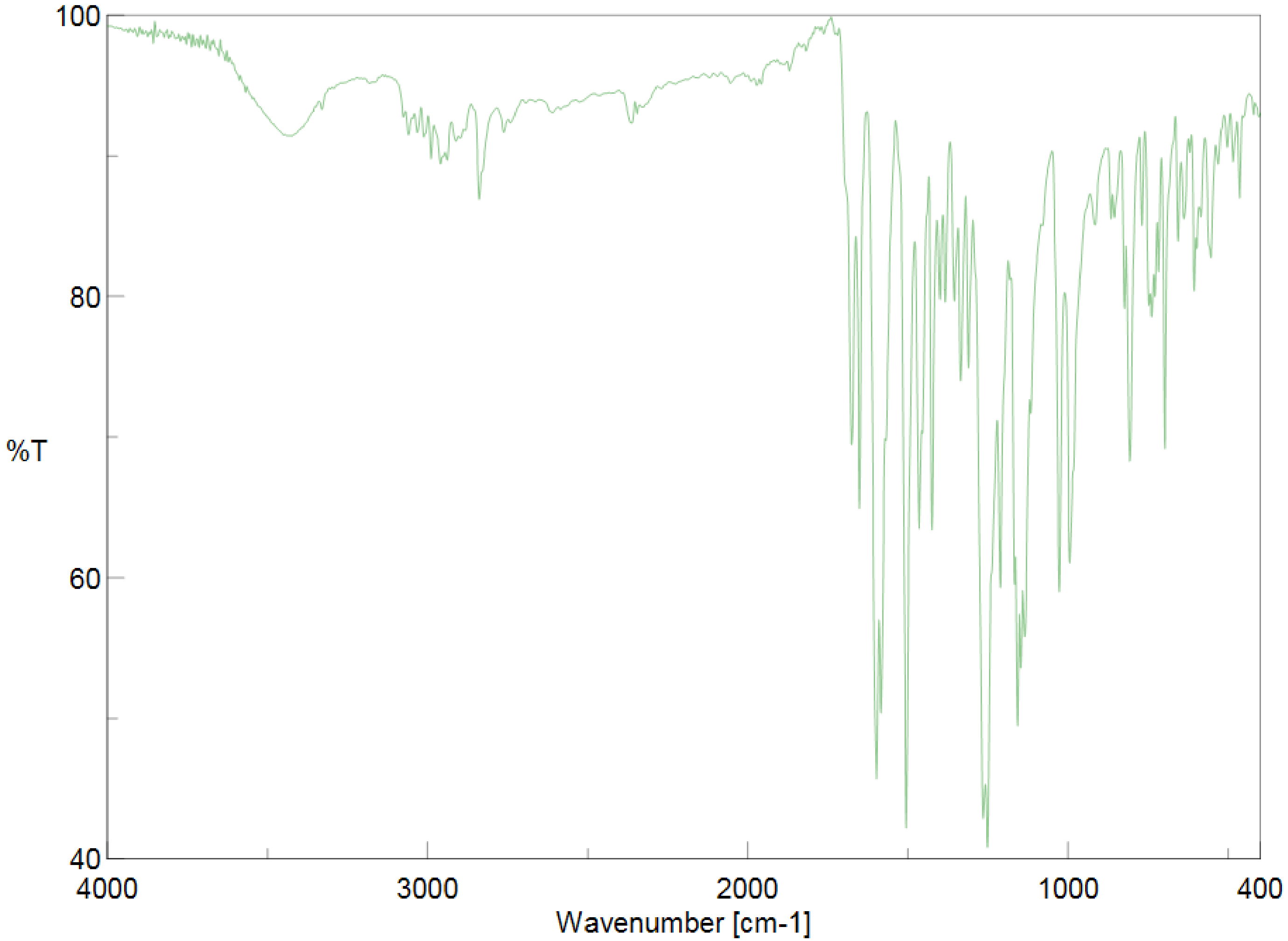

^{1}H-NMR Spectrum of **4v**

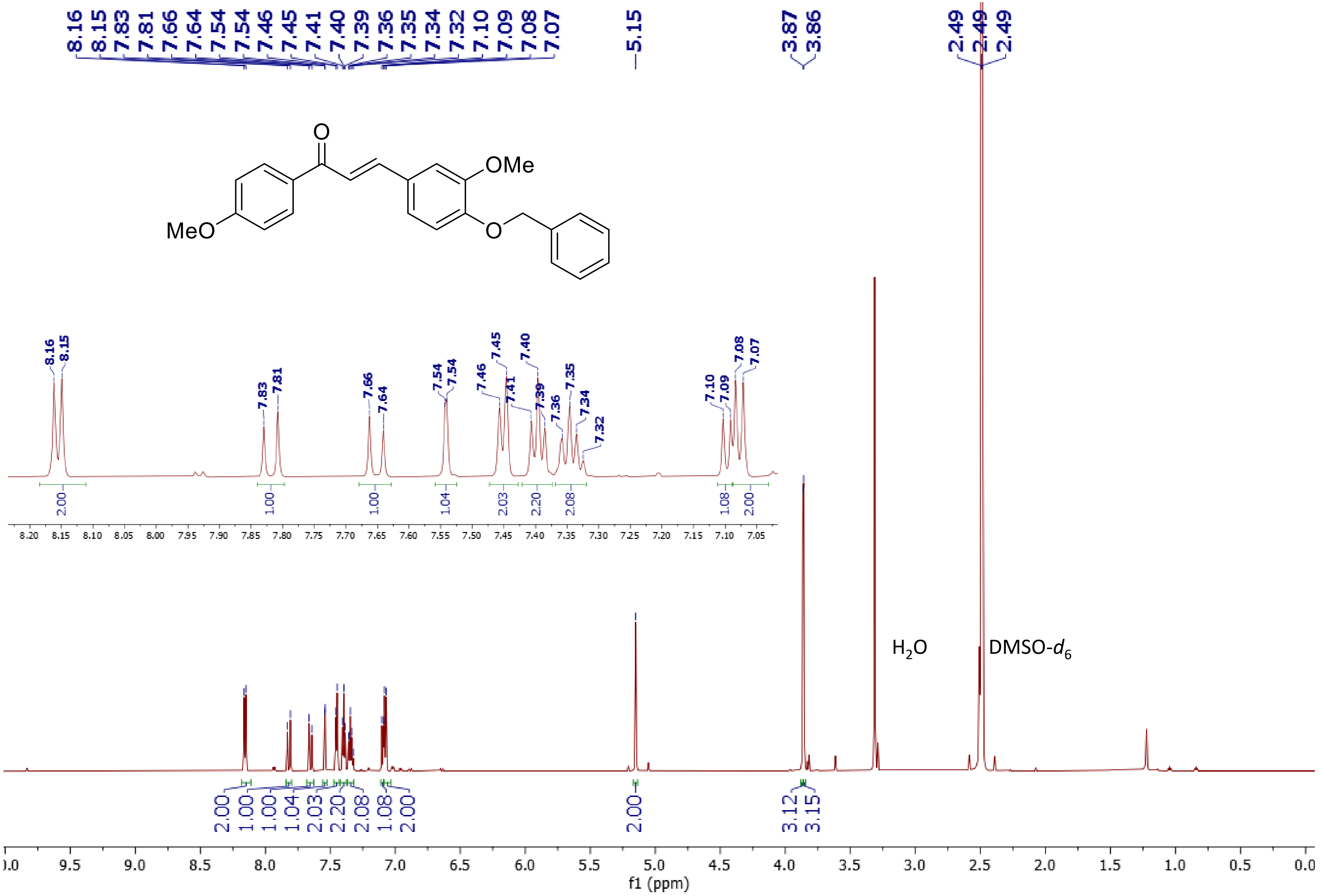

^{13}C-NMR Spectrum of **4v**

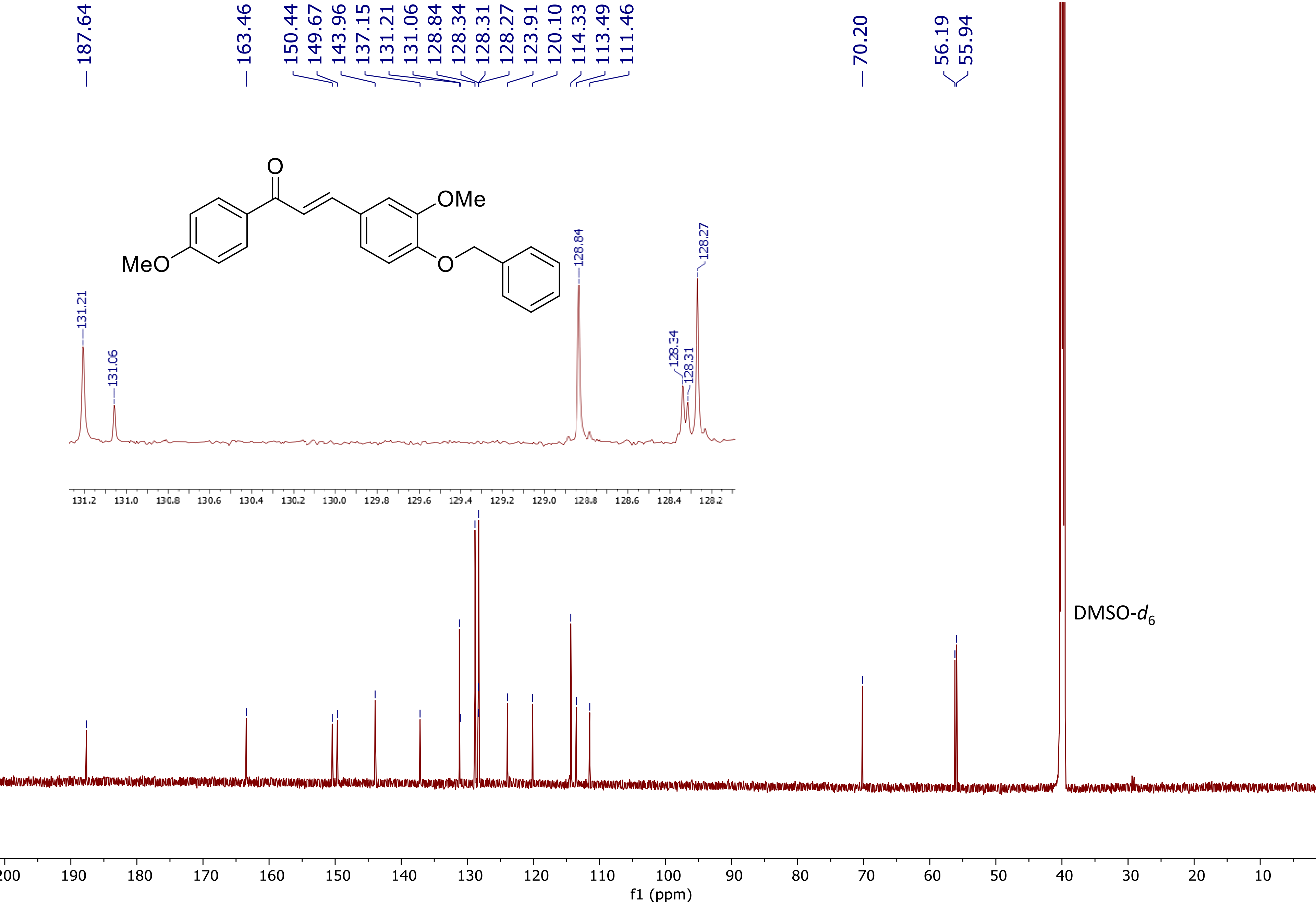

Mass Spectrum of **4v**

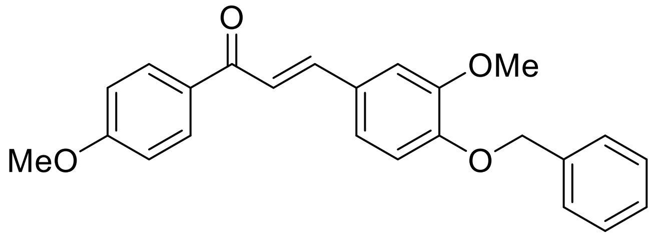

Chemical Formula: $C_{24}H_{22}O_4$

Exact Mass: 374.15

Molecular Weight: 374.44

m/z: 375 $[M+H]^+$; 397 $[M+Na]^+$

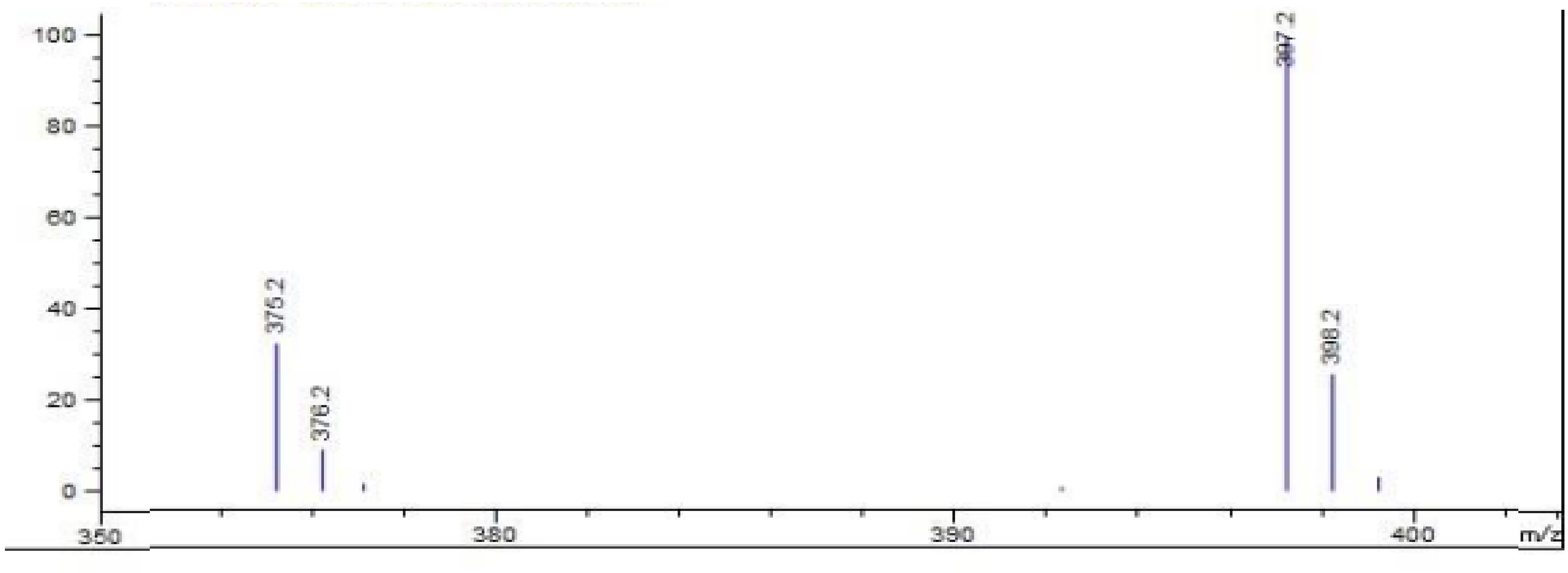

Table S1. Binding affinity, interacting key residues and types of interaction mediated by compound **4q** and Erlotinib with EGFR

Compound	Binding Affinity (kcal/mol)	Hydrophobic interaction	Van der walls interactions	Carbon-Hydrogen Bond
**4q**	-10.0	Leu777, Met766, Phe856, Val726, Ala743, Leu792, Met793, Leu844, Leu718	Arg776, Thr854, Thr790, Leu858, Leu788, Gln791, Gly796	Thr790
**Erlotinib**	-7.3	Leu718, Leu844	Gly796, Ala743, Leu792, Thr790, Phe856, Met766, Leu788, Leu777, Val726, Arg841, Gly719	Gln791, Asp855, Asn842

Table S2. Binding affinity, interacting key residues and types of interaction mediated by compound **4q** and Erlotinib with HER2

Compound	Binding Affinity (kcal/mol)	Hydrophobic interaction	Van der walls interactions	Carbon-hydrogen bond
**4q**	-10.5	Leu785, Leu796, Lys753, Val734, Ala751, Met801, Leu852, Leu726	Met774, Asp863	-
**Erlotinib**	-8.3	Leu785, Leu796, Leu852, Val734, Lys753, Ala751	Asn850	Gln799, Met801,Leu726, Asp863

Table S3. Binding affinity, interacting key residues and types of interaction mediated by compound **4q** and Sorafenib with VEGFR2

Compound	Binding Affinity (kcal/mol)	Hydrophobic interaction	Van der walls interactions	Carbon-hydrogen bond
**4q**	-10.1	Ala866, Val848, Cys919, Leu840, Leu1035, Val916, Val899, Leu1019	Phe918, Glu917,Glu885, Leu889, Phe1047,Asp1046, Val898	His1026
**Sorafenib**	-10.7	Leu1019, Lys868, Phe1047, Val916, Val848, Leu840, Phe918, Leu1035, Ala866, Leu889	Val898, Ile892, Ile888, Val899, Gly922	His1026, Cys1045, Glu917, Lys920

Table S4. Binding affinity, interacting key residues and types of interaction mediated by compound **4q** and Dinaciclib with CDK2

Compound	Binding Affinity (kcal/mol)	Hydrophobic interaction	Van der walls interactions	Carbon-hydrogen bond
**4q**	-8.5	Ile10, Ala31, Val18, Ala144, Leu134, Val64	Lys20, Glu81, His84, Gln85,Asp145, Phe80	Phe82
**Dinaciclib**	-9.1	Ala31,Ile10, Val18, Phe82, Leu134	Phe80, Val64, Ala144, Asp86, Gly11, Gly13, Asp145, Asn132, Gln131, Gln85, His84	Glu81

Table S5. Binding affinity, interacting key residues and types of interaction mediated by compound **4q** and Estrogen (Estradiol) with Estrogen receptor (1A52)

Compound	Binding Affinity (kcal/mol)	Conventional hydrogen bond	Hydrophobic interaction	Van der walls interactions
**4q**	-7.6	Lys529	Ile424, Met421,Leu387, Ala350	Leu428, Phe404, Gly521, Met388, Leu346, Leu391, Leu525
**Estradiol**	-10.6	Glu353, His524	Ile424, Leu346, Leu387, Met388, Leu391, Phe404, Ala350	Leu525, Met343, Met421, Gly521, Leu384, Leu428, Arg394, Leu349

Table S6. Binding affinity, interacting key residues and types of interaction mediated by compound **4q** and Tamoxifen with Estrogen receptor (3ERT)

Compound	Binding Affinity (kcal/mol)	Conventional hydrogen bond	Hydrophobic interaction	Van der walls interactions	Carbon-Hydrogen Bond
**4q**	-9.2	-	Met388, Leu391, Leu525, Leu346, Ala350, Trp383	Ile424, Met343, Leu387, Leu384, Phe404, Met421	Thr347
**Tamoxifen**	-9.8	-	Leu391, Leu428, Met388, Leu346, Leu387, Ala350, Leu525, Met421	Leu349, arg394, phe404, Ile424, His524, Met343, Gly521, Leu384, Thr347	-

Figure S1. IC_{50} of compound **4a** with EGFR

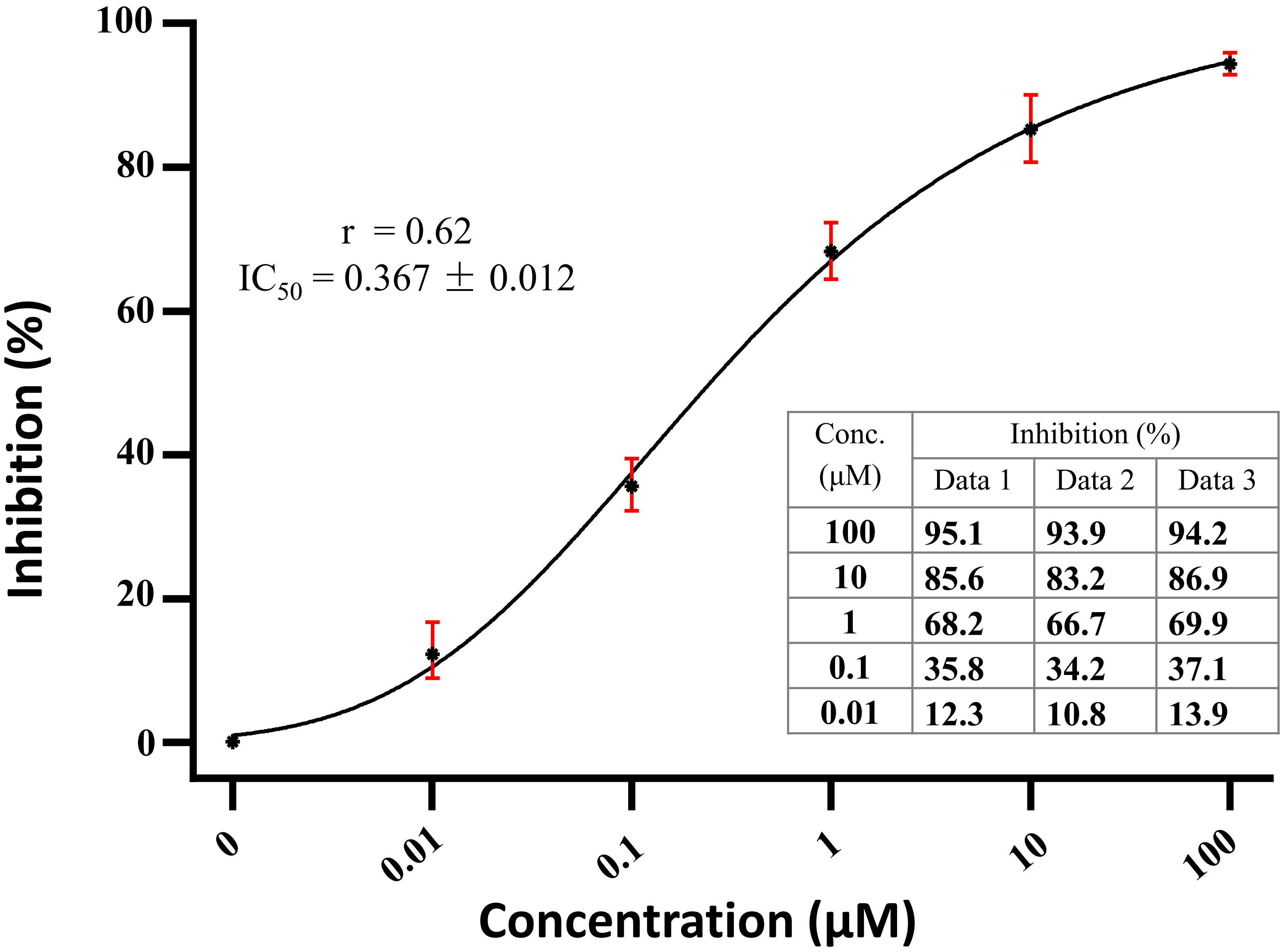

Conc. (μM)	Inhibition (%)		
	Data 1	Data 2	Data 3
**100**	**95.1**	**93.9**	**94.2**
**10**	**85.6**	**83.2**	**86.9**
**1**	**68.2**	**66.7**	**69.9**
**0.1**	**35.8**	**34.2**	**37.1**
**0.01**	**12.3**	**10.8**	**13.9**

Figure S2. IC_{50} of compound **4b** with EGFR

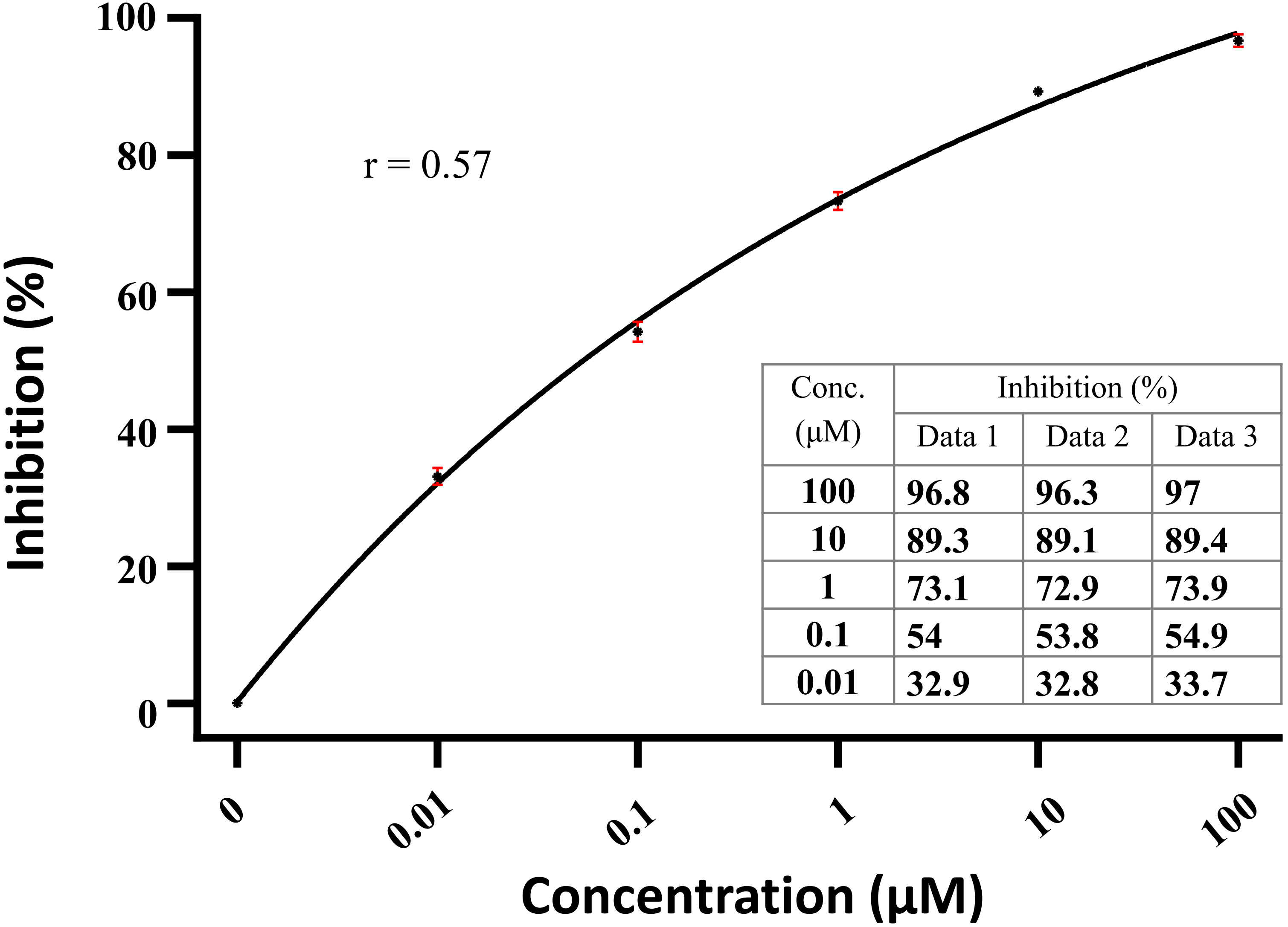

Conc. (µM)	Inhibition (%)		
	Data 1	Data 2	Data 3
**100**	**96.8**	**96.3**	**97**
**10**	**89.3**	**89.1**	**89.4**
**1**	**73.1**	**72.9**	**73.9**
**0.1**	**54**	**53.8**	**54.9**
**0.01**	**32.9**	**32.8**	**33.7**

Figure S3. IC_{50} of compound **4q** with EGFR

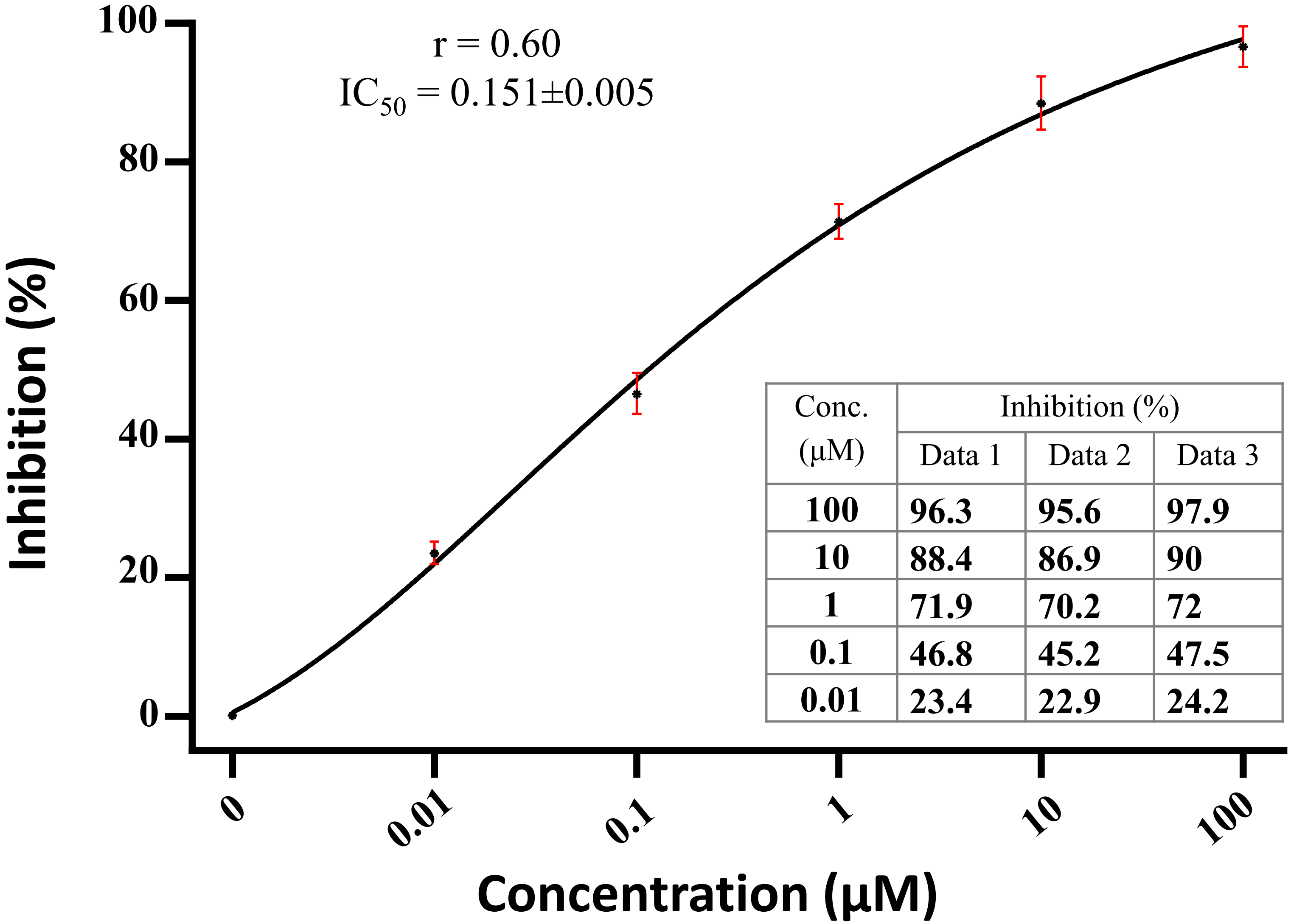

Conc. (μM)	Inhibition (%)		
	Data 1	Data 2	Data 3
**100**	**96.3**	**95.6**	**97.9**
**10**	**88.4**	**86.9**	**90**
**1**	**71.9**	**70.2**	**72**
**0.1**	**46.8**	**45.2**	**47.5**
**0.01**	**23.4**	**22.9**	**24.2**

Figure S4. IC_{50} of compound **4v** with EGFR

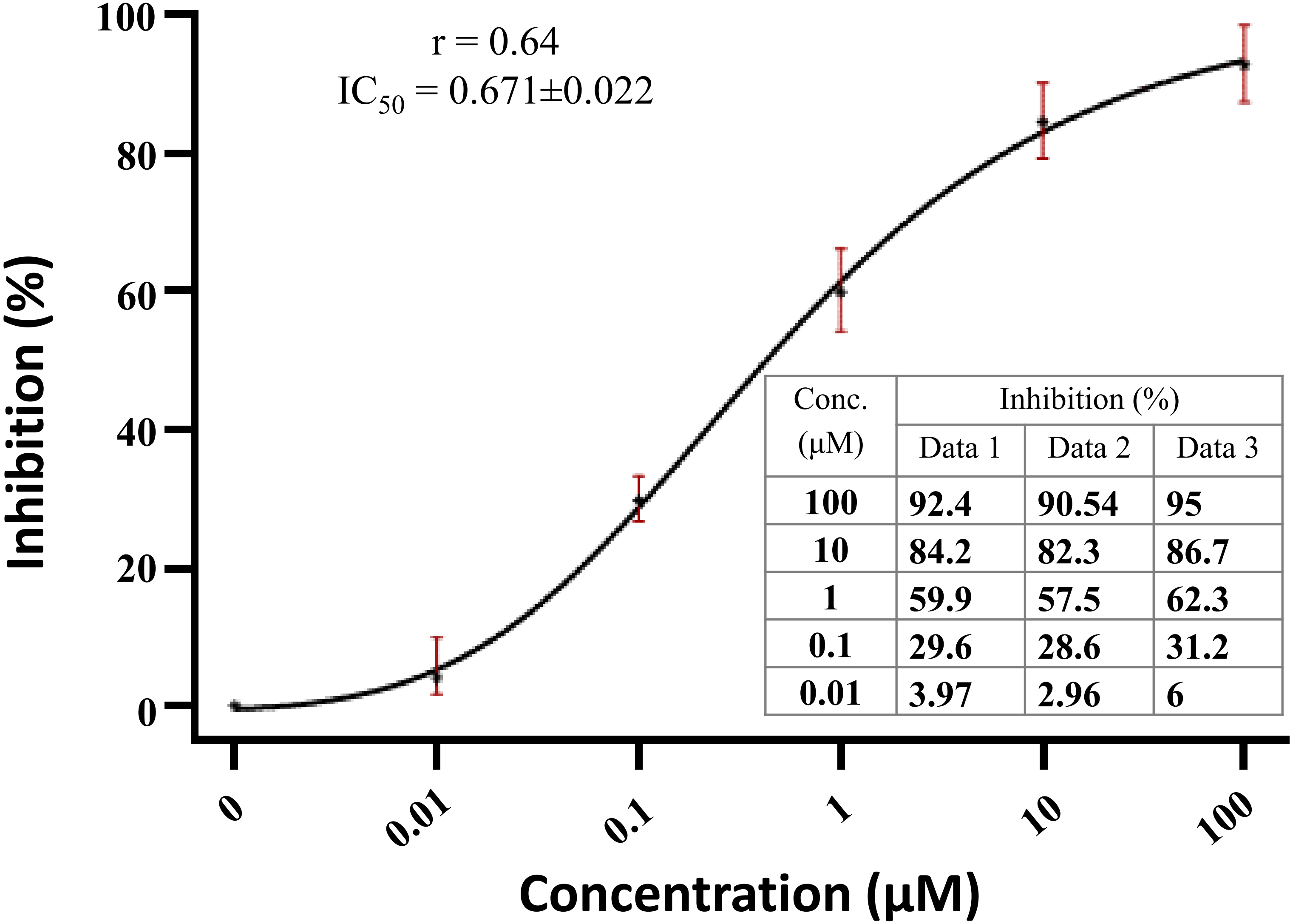

Conc. (μM)	Inhibition (%)		
	Data 1	Data 2	Data 3
**100**	**92.4**	**90.54**	**95**
**10**	**84.2**	**82.3**	**86.7**
**1**	**59.9**	**57.5**	**62.3**
**0.1**	**29.6**	**28.6**	**31.2**
**0.01**	**3.97**	**2.96**	**6**

Figure S5. IC_{50} of compound **erlotinib** with EGFR

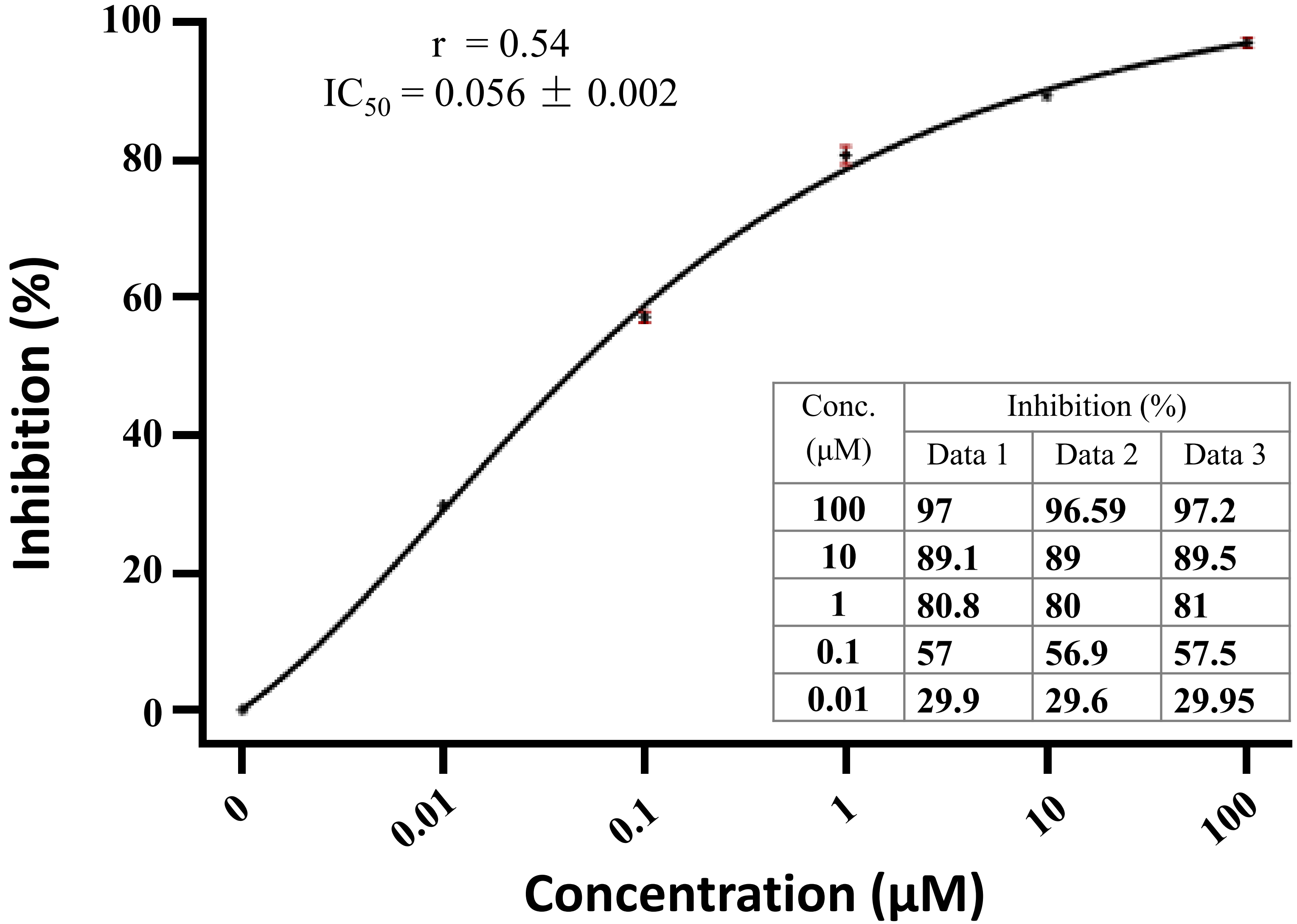

Conc. (μM)	Inhibition (%)		
	Data 1	Data 2	Data 3
**100**	**97**	**96.59**	**97.2**
**10**	**89.1**	**89**	**89.5**
**1**	**80.8**	**80**	**81**
**0.1**	**57**	**56.9**	**57.5**
**0.01**	**29.9**	**29.6**	**29.95**

Figure S6. IC_{50} of compound **4a** with Her2

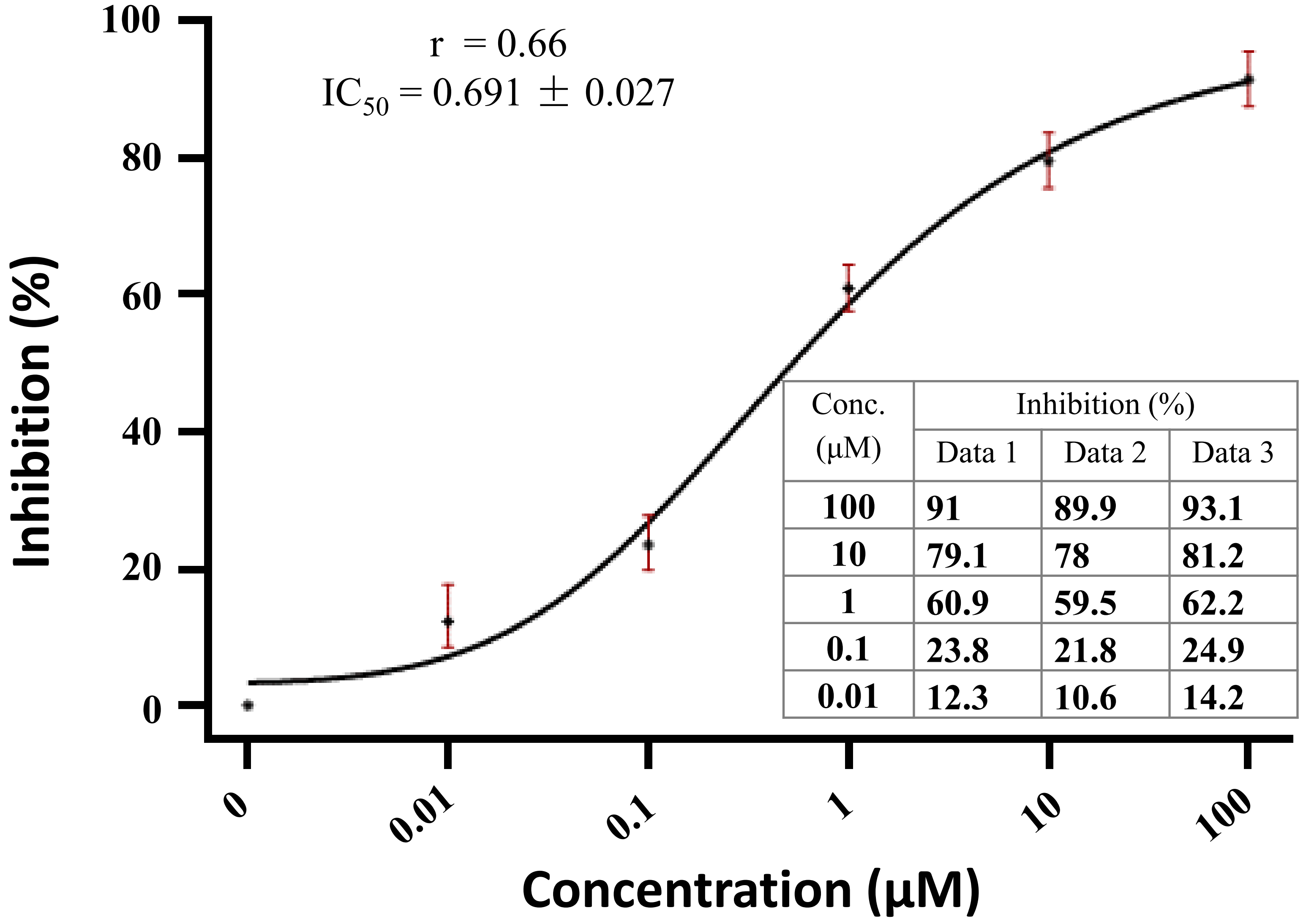

Conc. (μM)	Inhibition (%)		
	Data 1	Data 2	Data 3
100	91	89.9	93.1
10	79.1	78	81.2
1	60.9	59.5	62.2
0.1	23.8	21.8	24.9
0.01	12.3	10.6	14.2

Figure S7. IC_{50} of compound **4b** with Her2

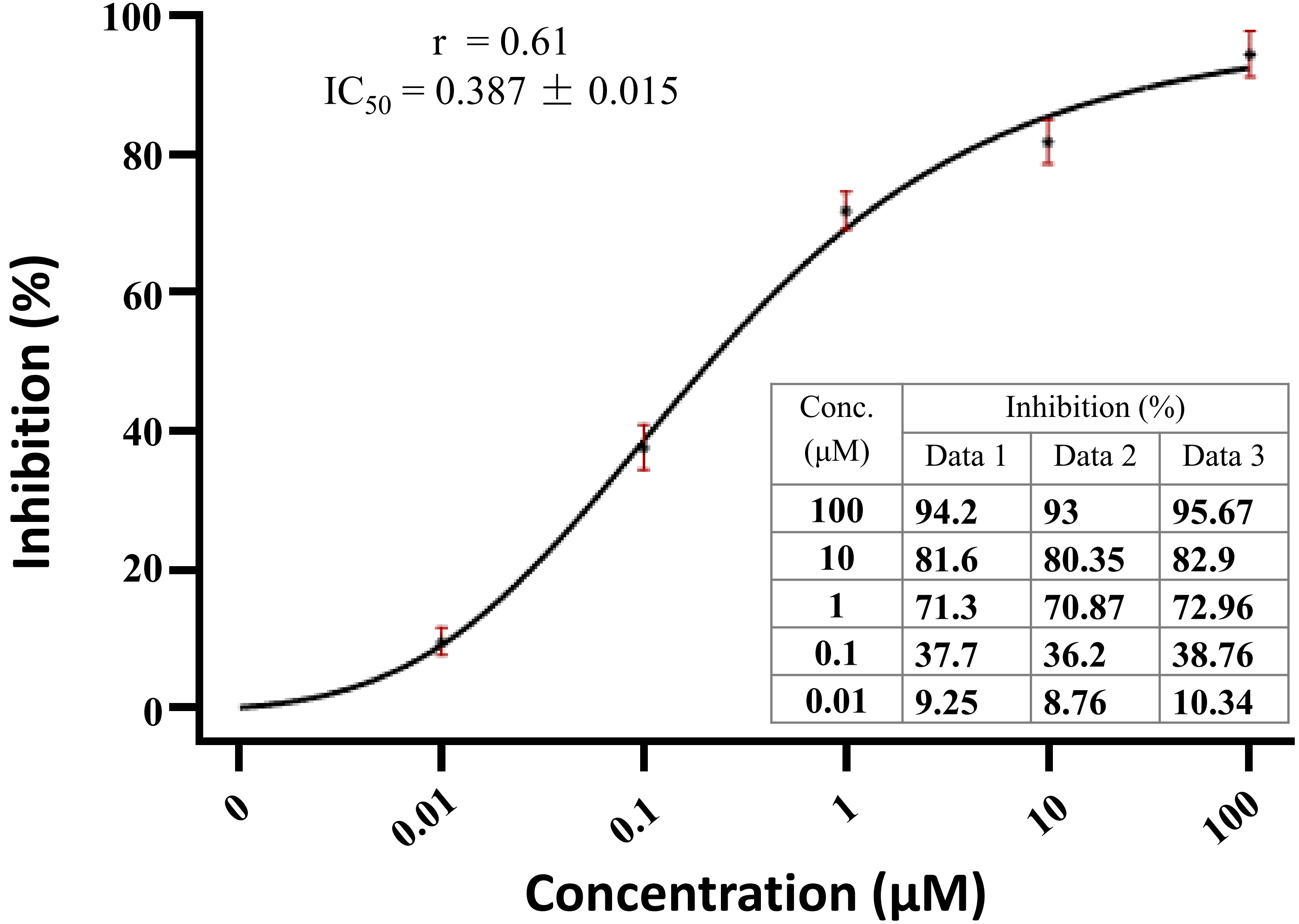

Conc. (μM)	Inhibition (%)		
	Data 1	Data 2	Data 3
**100**	**94.2**	**93**	**95.67**
**10**	**81.6**	**80.35**	**82.9**
**1**	**71.3**	**70.87**	**72.96**
**0.1**	**37.7**	**36.2**	**38.76**
**0.01**	**9.25**	**8.76**	**10.34**

Figure S8. IC_{50} of compound **4q** with Her2

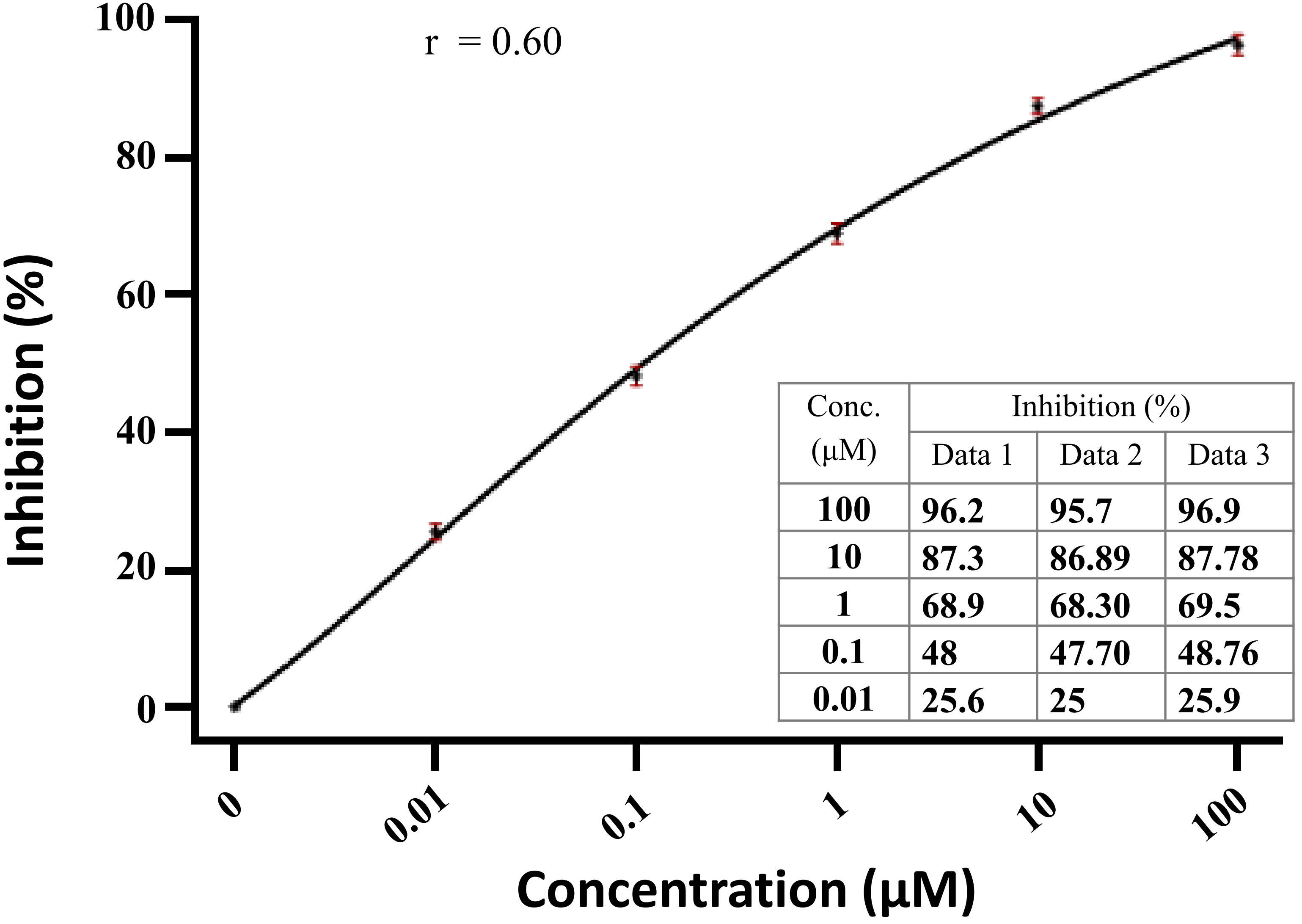

Conc. (µM)	Inhibition (%)		
	Data 1	Data 2	Data 3
**100**	**96.2**	**95.7**	**96.9**
**10**	**87.3**	**86.89**	**87.78**
**1**	**68.9**	**68.30**	**69.5**
**0.1**	**48**	**47.70**	**48.76**
**0.01**	**25.6**	**25**	**25.9**

Figure S9. IC_{50} of compound **4v** with Her2

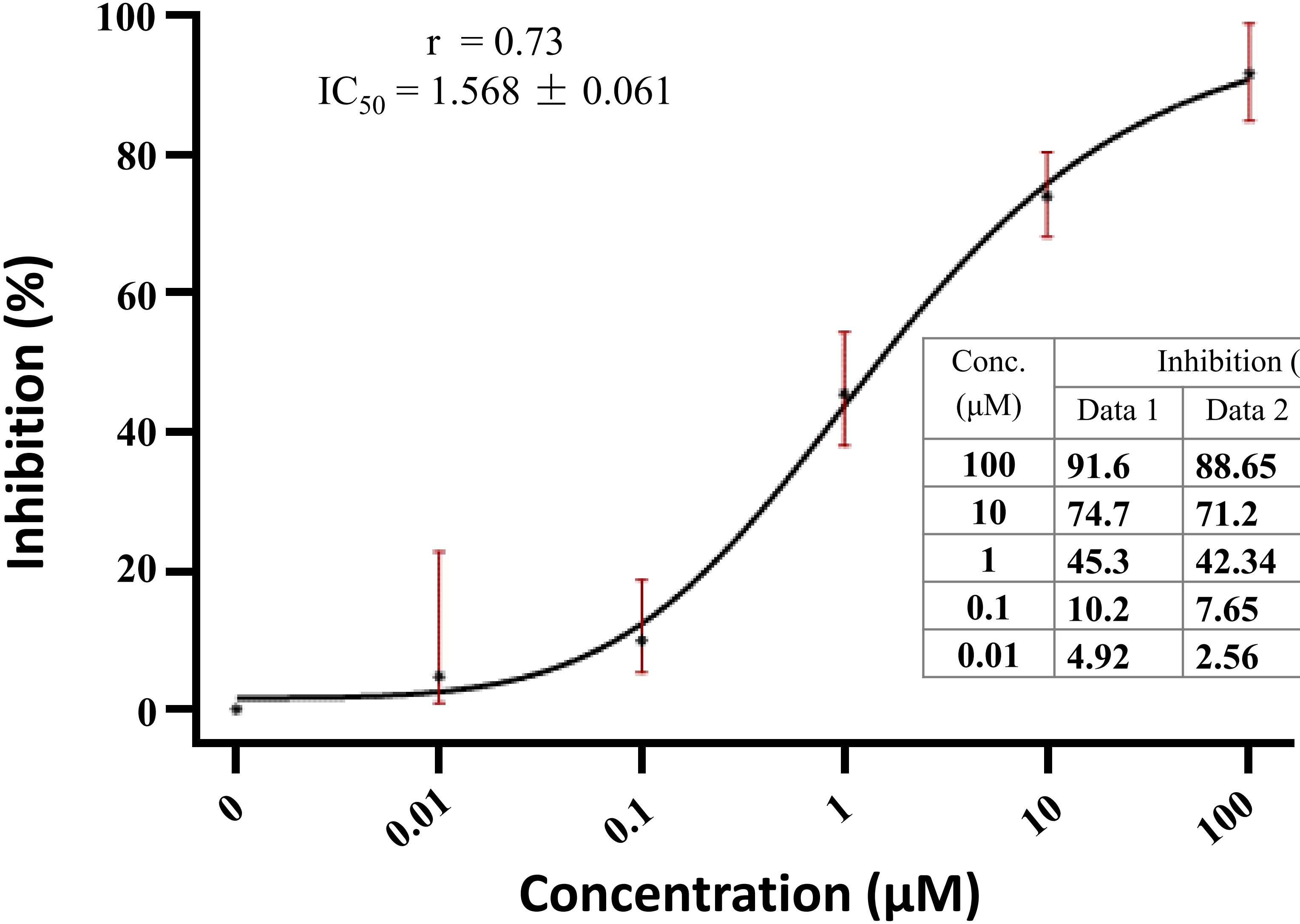

Conc. (μM)	Inhibition (%)		
	Data 1	Data 2	Data 3
**100**	**91.6**	**88.65**	**94.3**
**10**	**74.7**	**71.2**	**75.89**
**1**	**45.3**	**42.34**	**48.9**
**0.1**	**10.2**	**7.65**	**12.67**
**0.01**	**4.92**	**2.56**	**8.92**

Figure S10. IC_{50} of compound **erlotinib** with Her2

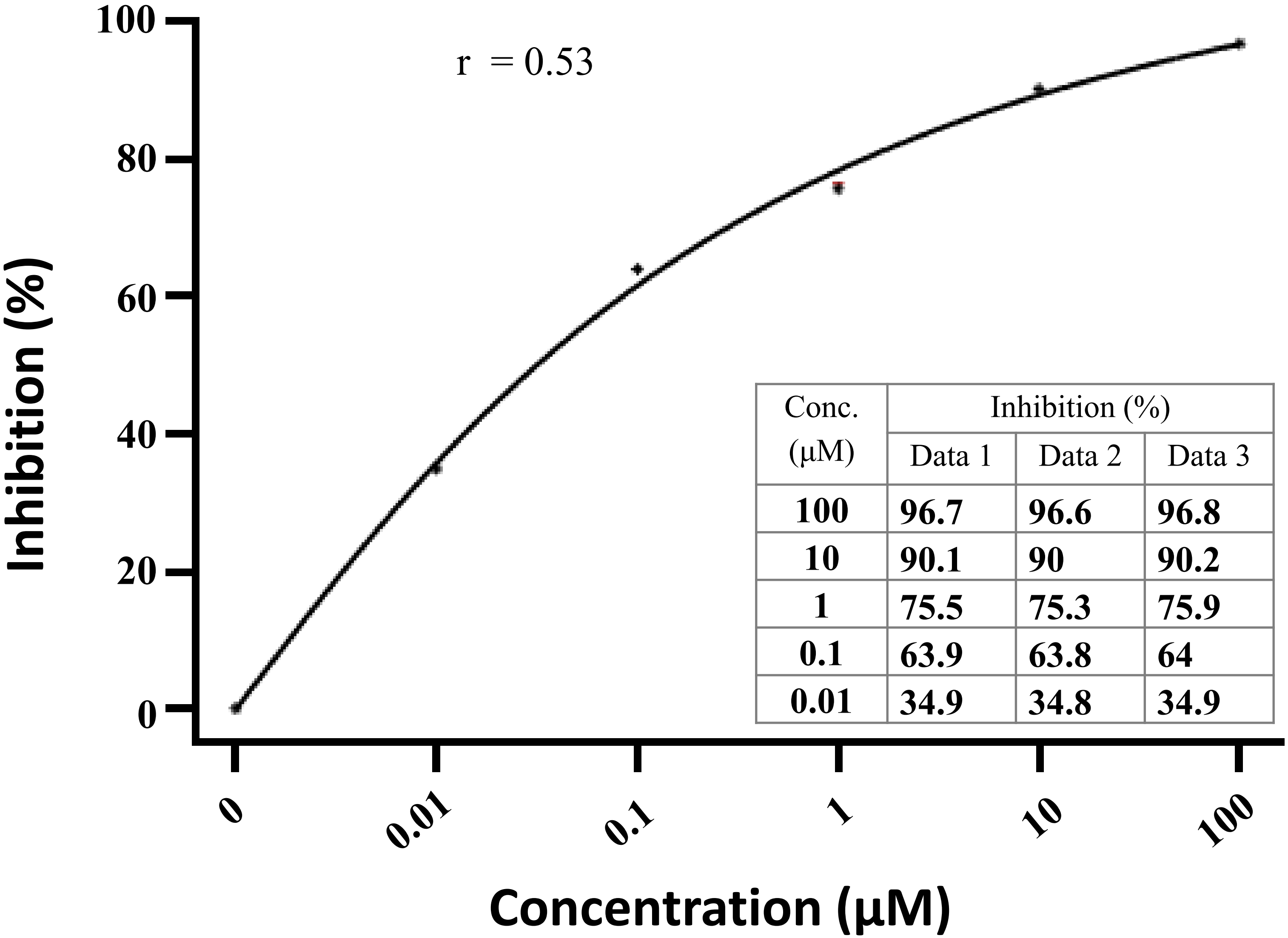

Conc. (µM)	Inhibition (%)		
	Data 1	Data 2	Data 3
**100**	**96.7**	**96.6**	**96.8**
**10**	**90.1**	**90**	**90.2**
**1**	**75.5**	**75.3**	**75.9**
**0.1**	**63.9**	**63.8**	**64**
**0.01**	**34.9**	**34.8**	**34.9**

Figure S11. IC_{50} of compound **4a** with VEGFR2

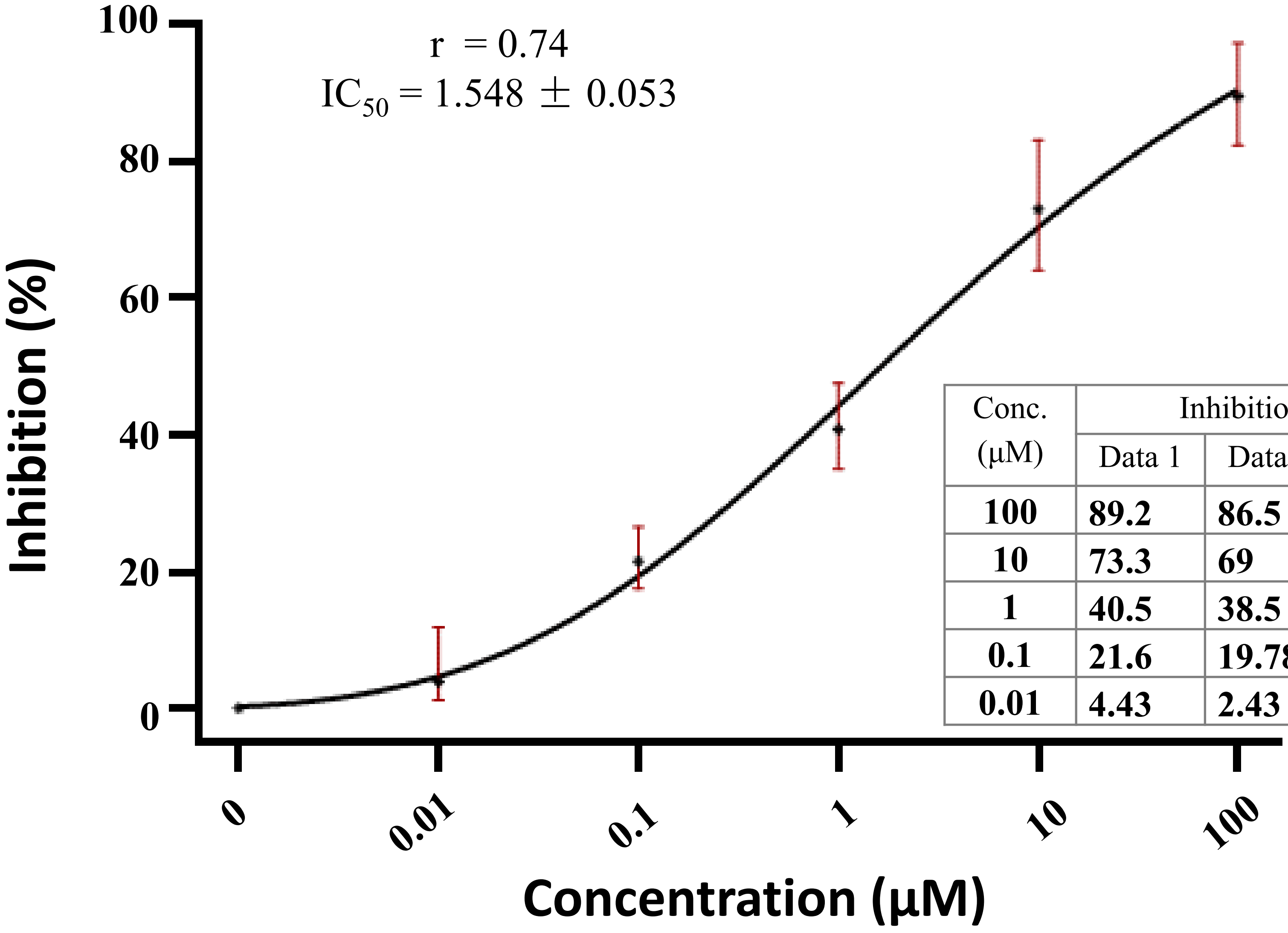

Conc. (µM)	Inhibition (%)		
	Data 1	Data 2	Data 3
**100**	**89.2**	**86.5**	**92.5**
**10**	**73.3**	**69**	**76.6**
**1**	**40.5**	**38.5**	**43.5**
**0.1**	**21.6**	**19.78**	**23.4**
**0.01**	**4.43**	**2.43**	**5.78**

Figure S12. IC_{50} of compound **4b** with VEGFR2

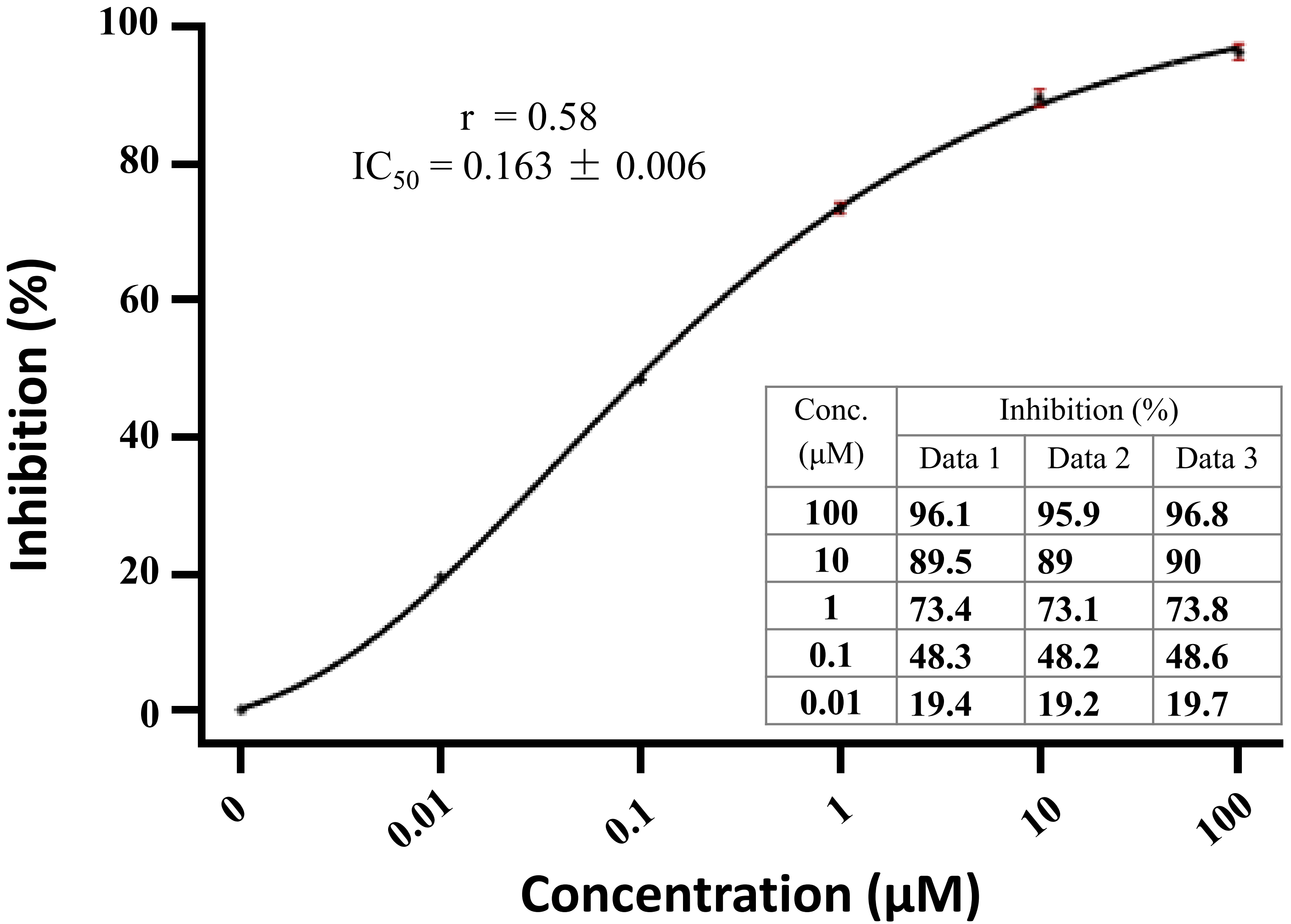

Conc. (μM)	Inhibition (%)		
	Data 1	Data 2	Data 3
**100**	**96.1**	**95.9**	**96.8**
**10**	**89.5**	**89**	**90**
**1**	**73.4**	**73.1**	**73.8**
**0.1**	**48.3**	**48.2**	**48.6**
**0.01**	**19.4**	**19.2**	**19.7**

Figure S13. IC_{50} of compound **4q** with VEGFR2

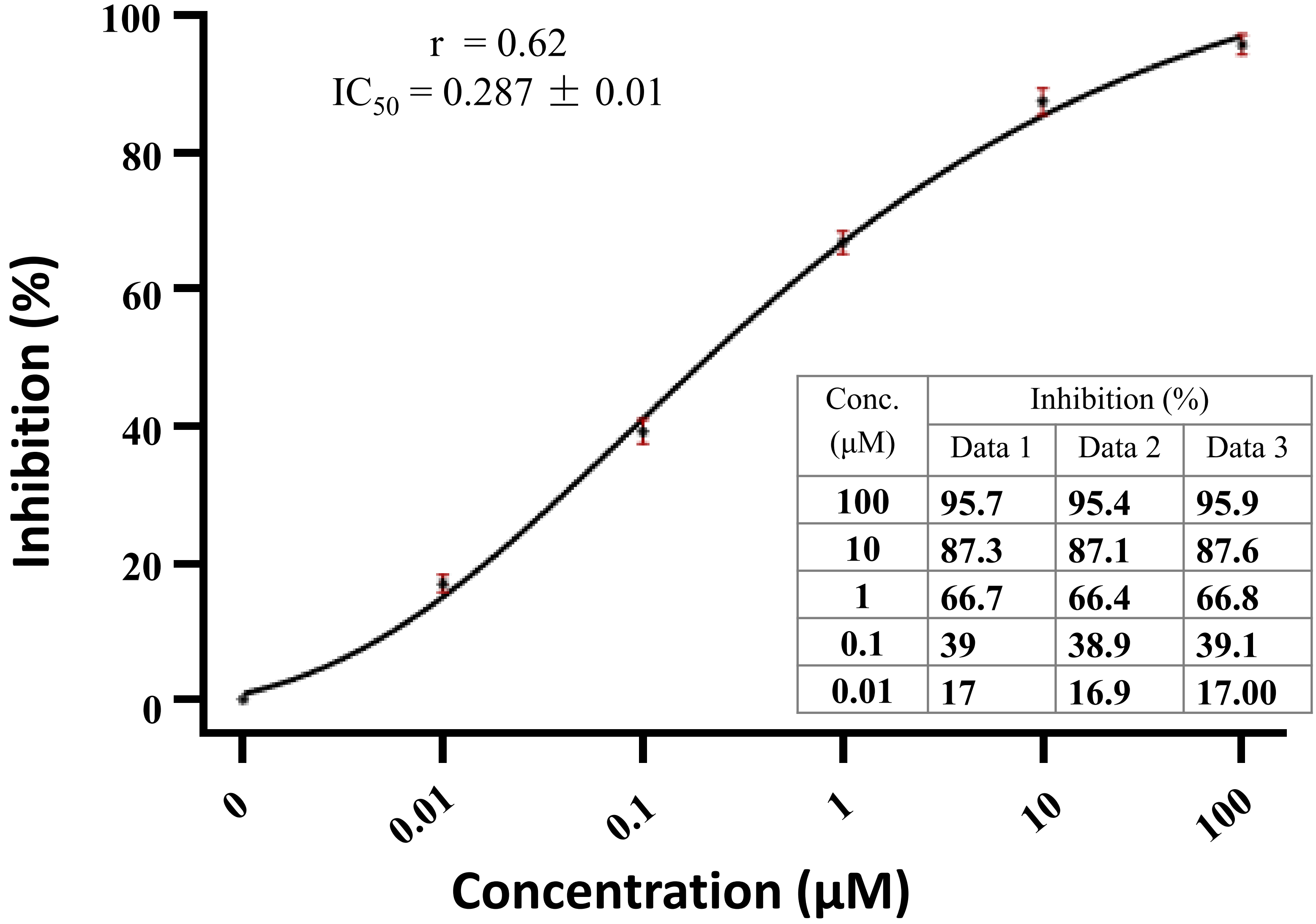

Conc. (μM)	Inhibition (%)		
	Data 1	Data 2	Data 3
**100**	**95.7**	**95.4**	**95.9**
**10**	**87.3**	**87.1**	**87.6**
**1**	**66.7**	**66.4**	**66.8**
**0.1**	**39**	**38.9**	**39.1**
**0.01**	**17**	**16.9**	**17.00**

Figure S14. IC_{50} of compound **4v** with VEGFR2

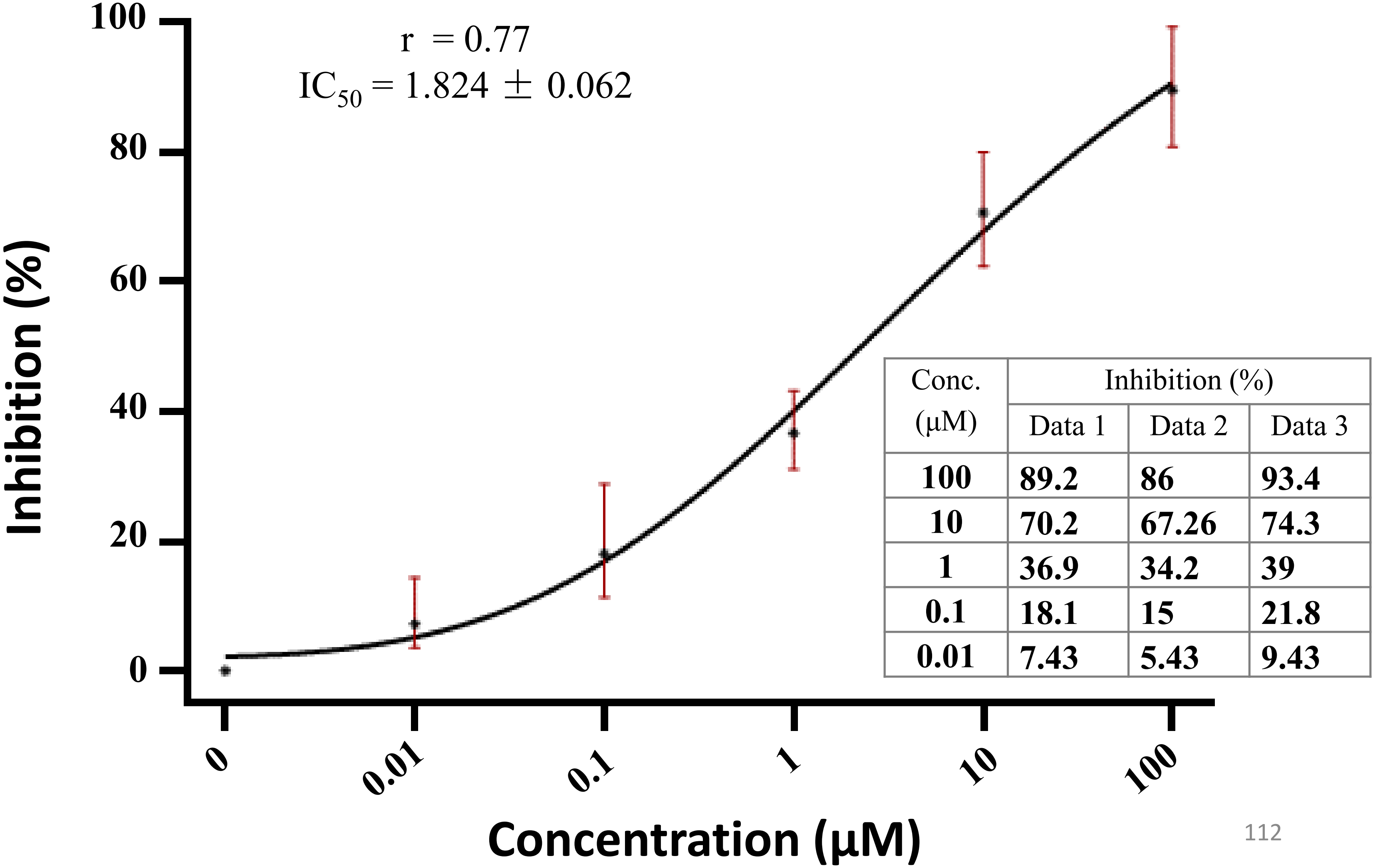

Conc. (µM)	Inhibition (%)		
	Data 1	Data 2	Data 3
**100**	**89.2**	**86**	**93.4**
**10**	**70.2**	**67.26**	**74.3**
**1**	**36.9**	**34.2**	**39**
**0.1**	**18.1**	**15**	**21.8**
**0.01**	**7.43**	**5.43**	**9.43**

Figure S15. IC_{50} of compound **sorafenib** with VEGFR2

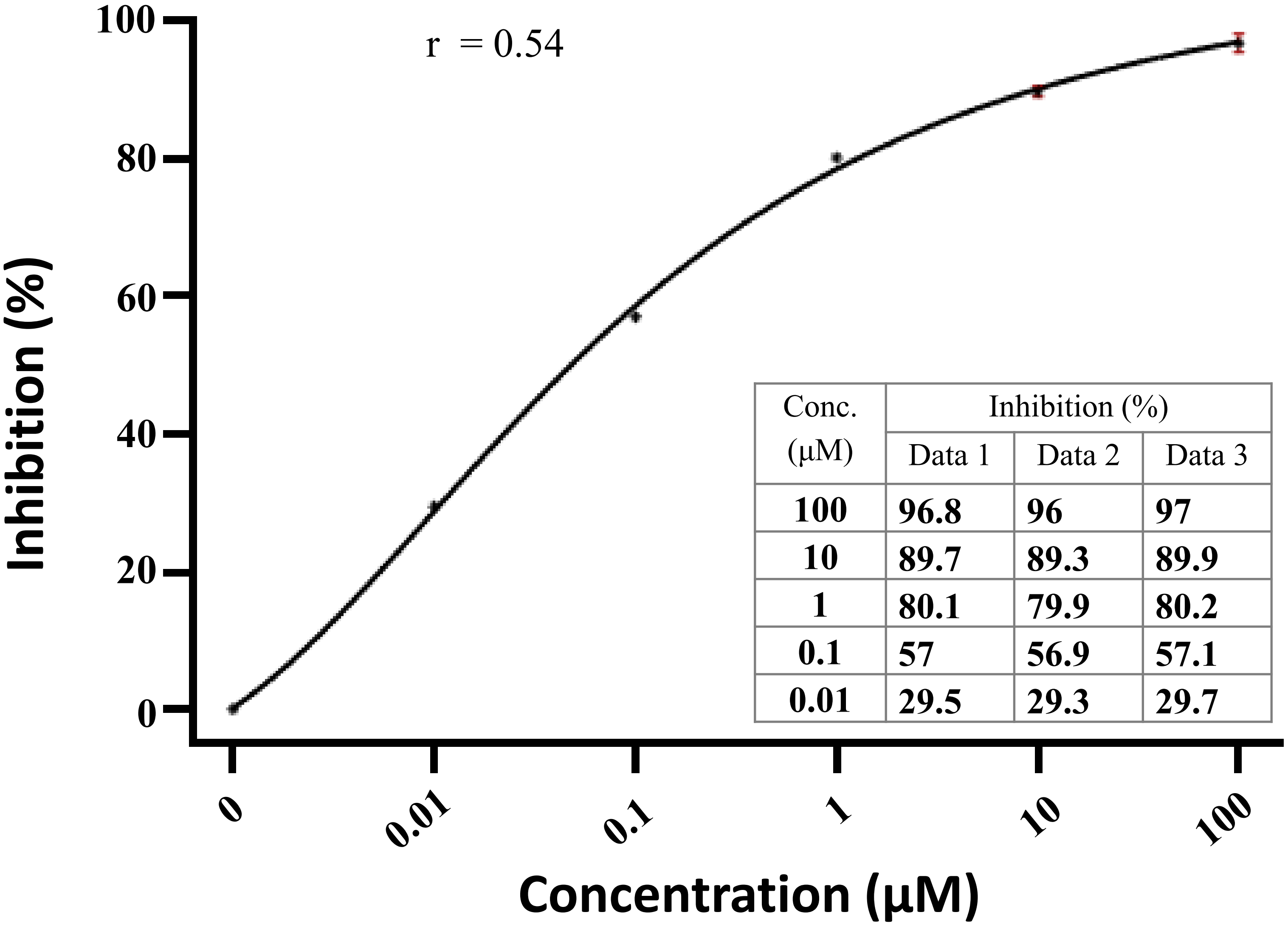

Conc. (µM)	Inhibition (%)		
	Data 1	Data 2	Data 3
**100**	**96.8**	**96**	**97**
**10**	**89.7**	**89.3**	**89.9**
**1**	**80.1**	**79.9**	**80.2**
**0.1**	**57**	**56.9**	**57.1**
**0.01**	**29.5**	**29.3**	**29.7**

Figure S16. IC_{50} of compound **4a** with CDK2

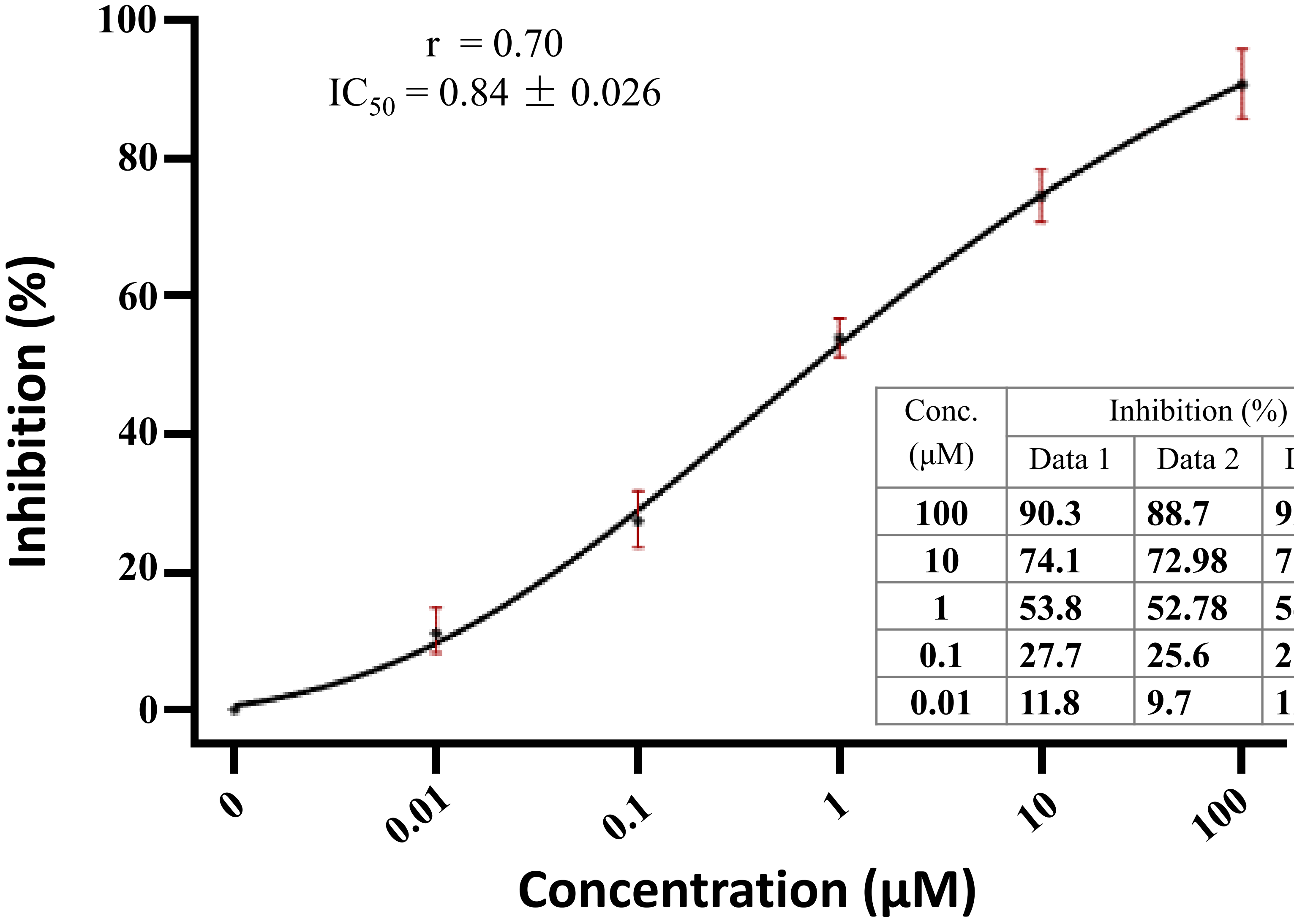

Conc. (µM)	Inhibition (%)		
	Data 1	Data 2	Data 3
**100**	**90.3**	**88.7**	**92.7**
**10**	**74.1**	**72.98**	**76**
**1**	**53.8**	**52.78**	**54**
**0.1**	**27.7**	**25.6**	**28.78**
**0.01**	**11.8**	**9.7**	**12**

Figure S17. IC_{50} of compound **4b** with CDK2

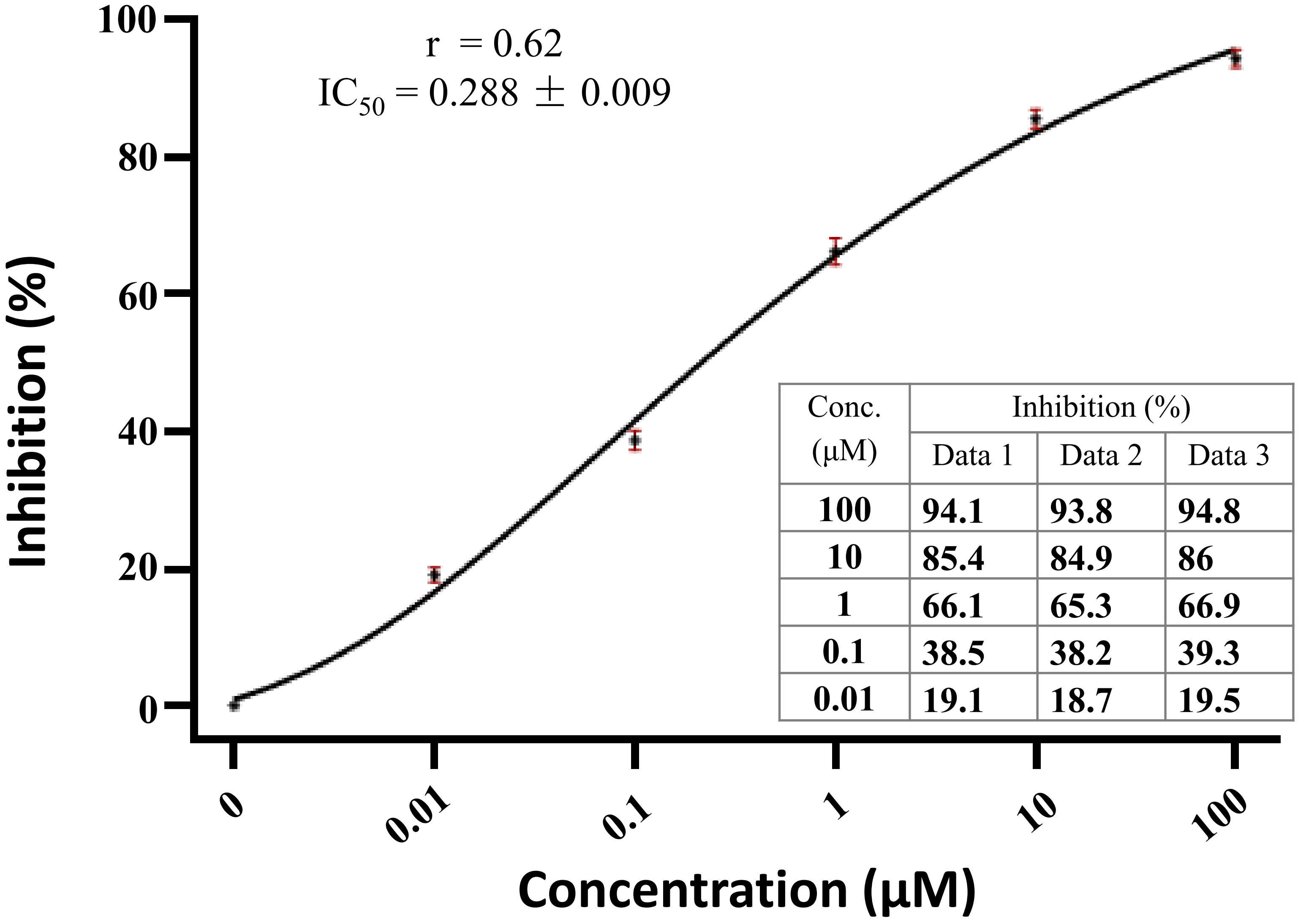

Conc. (µM)	Inhibition (%)		
	Data 1	Data 2	Data 3
**100**	**94.1**	**93.8**	**94.8**
**10**	**85.4**	**84.9**	**86**
**1**	**66.1**	**65.3**	**66.9**
**0.1**	**38.5**	**38.2**	**39.3**
**0.01**	**19.1**	**18.7**	**19.5**

Figure S18. IC_{50} of compound **4q** with CDK2

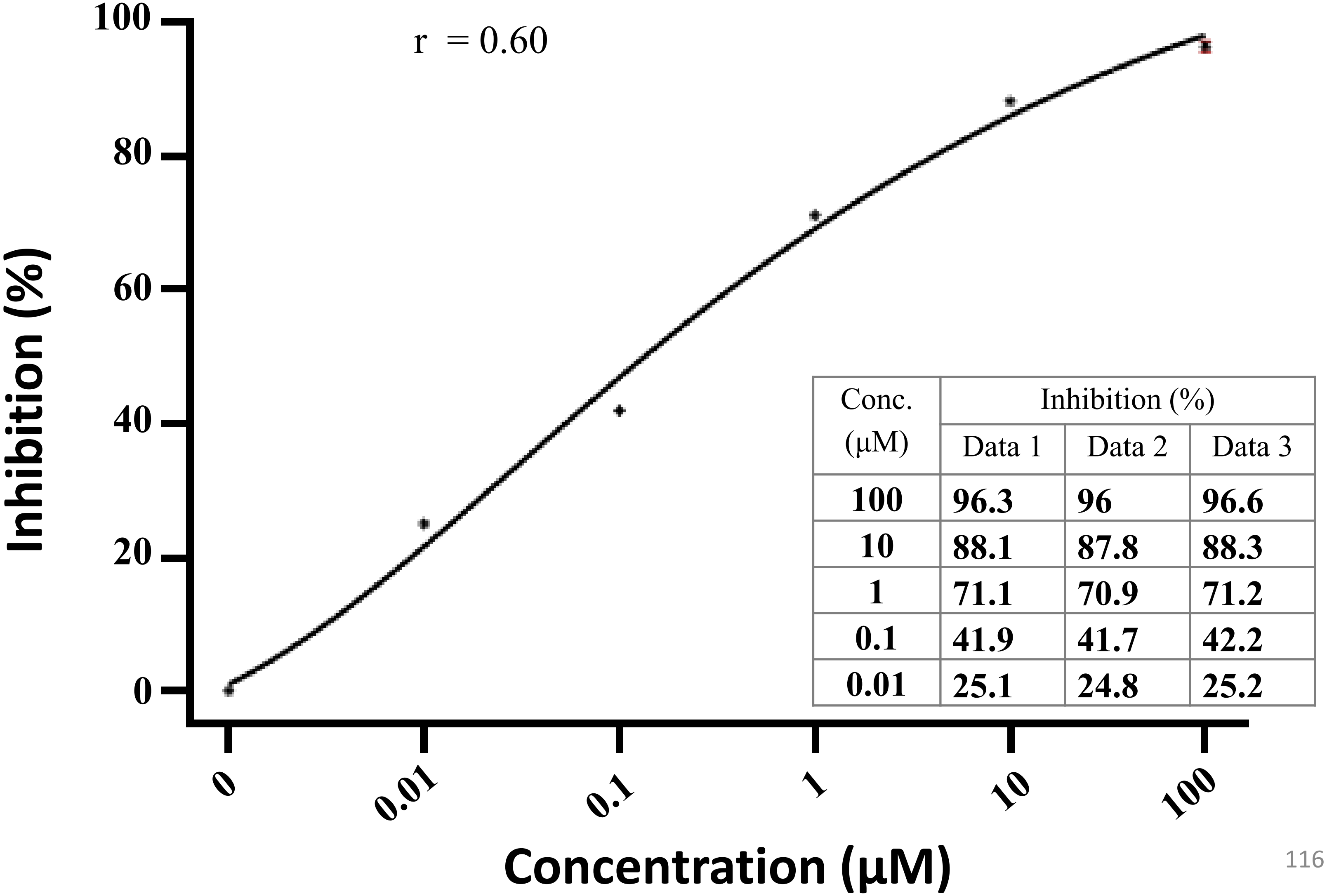

Conc. (µM)	Inhibition (%)		
	Data 1	Data 2	Data 3
**100**	**96.3**	**96**	**96.6**
**10**	**88.1**	**87.8**	**88.3**
**1**	**71.1**	**70.9**	**71.2**
**0.1**	**41.9**	**41.7**	**42.2**
**0.01**	**25.1**	**24.8**	**25.2**

Figure S19. IC_{50} of compound **4v** with CDK2

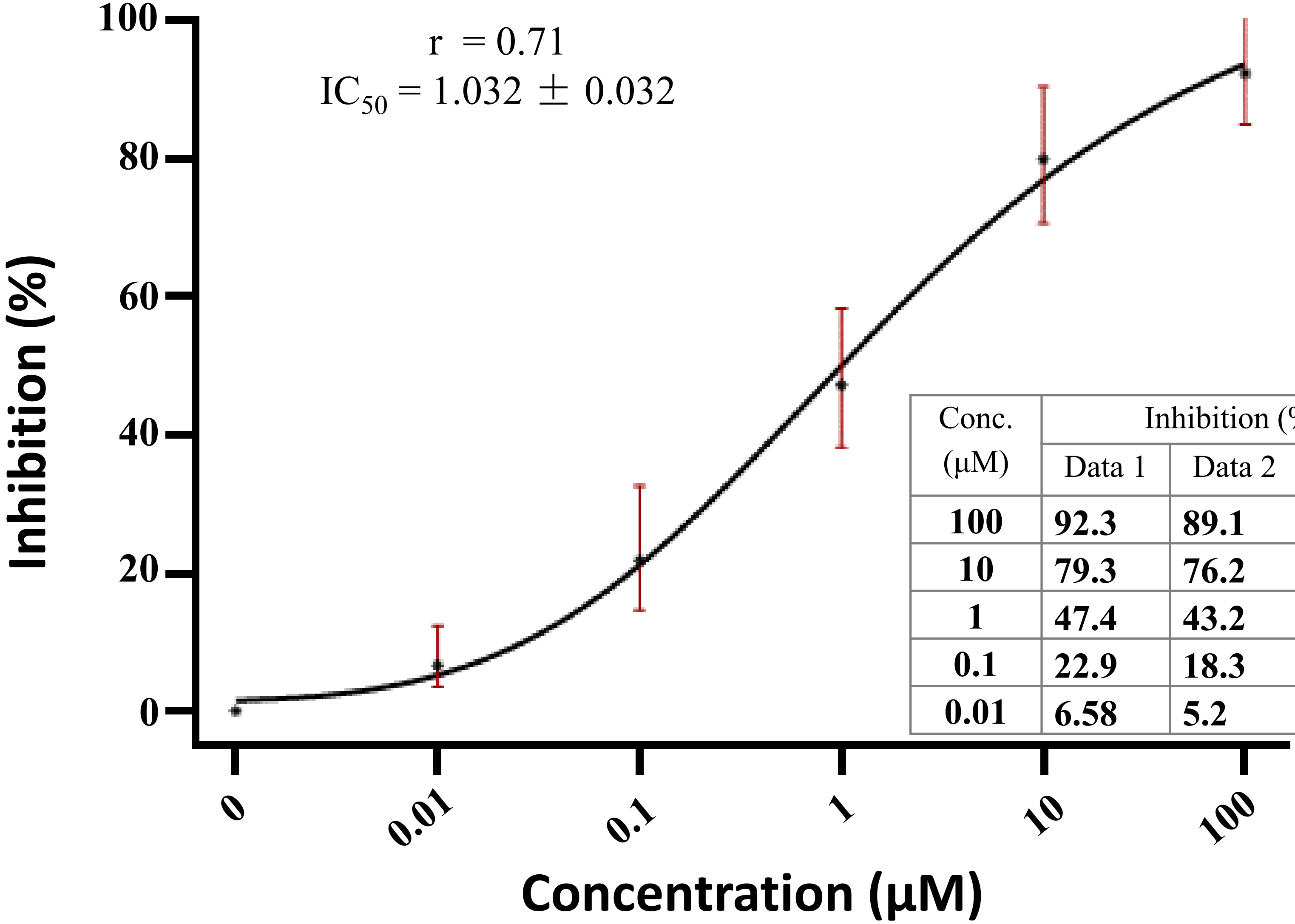

Conc. (μM)	Inhibition (%)		
	Data 1	Data 2	Data 3
**100**	**92.3**	**89.1**	**95.3**
**10**	**79.3**	**76.2**	**84.1**
**1**	**47.4**	**43.2**	**51.2**
**0.1**	**22.9**	**18.3**	**24.97**
**0.01**	**6.58**	**5.2**	**8.6**

Figure S20. IC_{50} of compound **dinaciclib** with CDK2

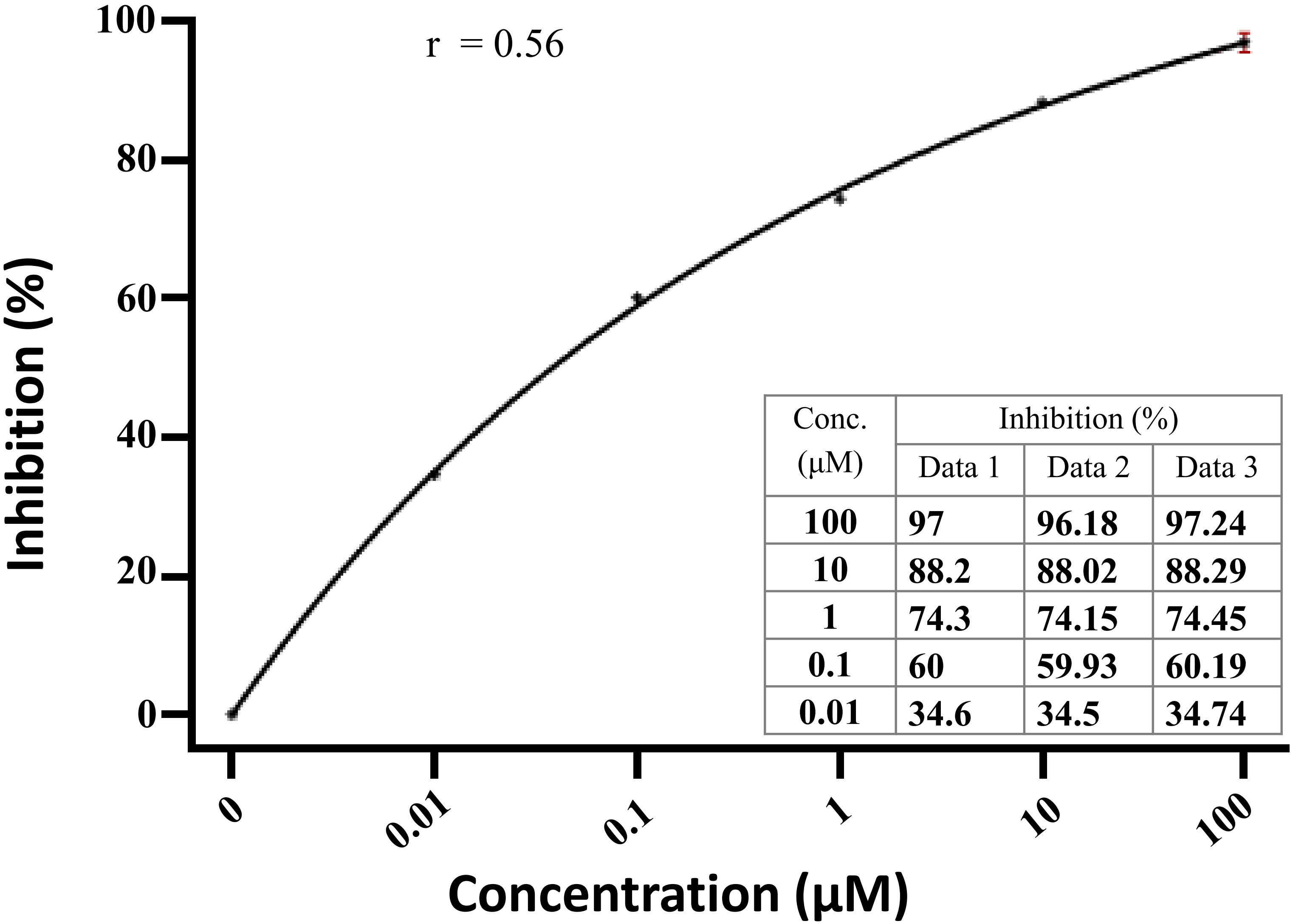

Conc. (μM)	Inhibition (%)		
	Data 1	Data 2	Data 3
**100**	**97**	**96.18**	**97.24**
**10**	**88.2**	**88.02**	**88.29**
**1**	**74.3**	**74.15**	**74.45**
**0.1**	**60**	**59.93**	**60.19**
**0.01**	**34.6**	**34.5**	**34.74**

Figure S21. Compound **4a** with Aromatase

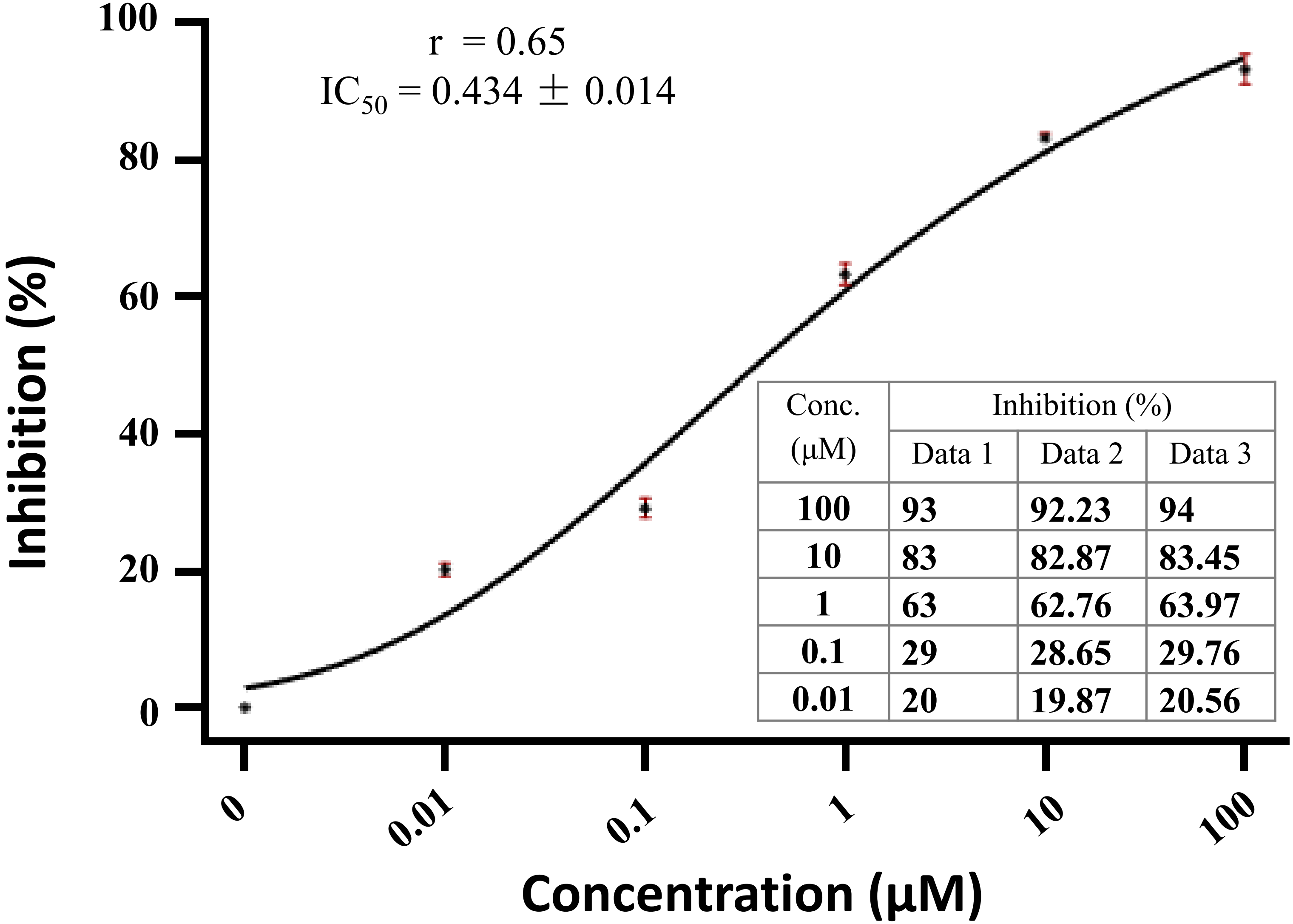

Conc. (µM)	Inhibition (%)		
	Data 1	Data 2	Data 3
**100**	**93**	**92.23**	**94**
**10**	**83**	**82.87**	**83.45**
**1**	**63**	**62.76**	**63.97**
**0.1**	**29**	**28.65**	**29.76**
**0.01**	**20**	**19.87**	**20.56**

Figure S22. Compound **4b** with Aromatase

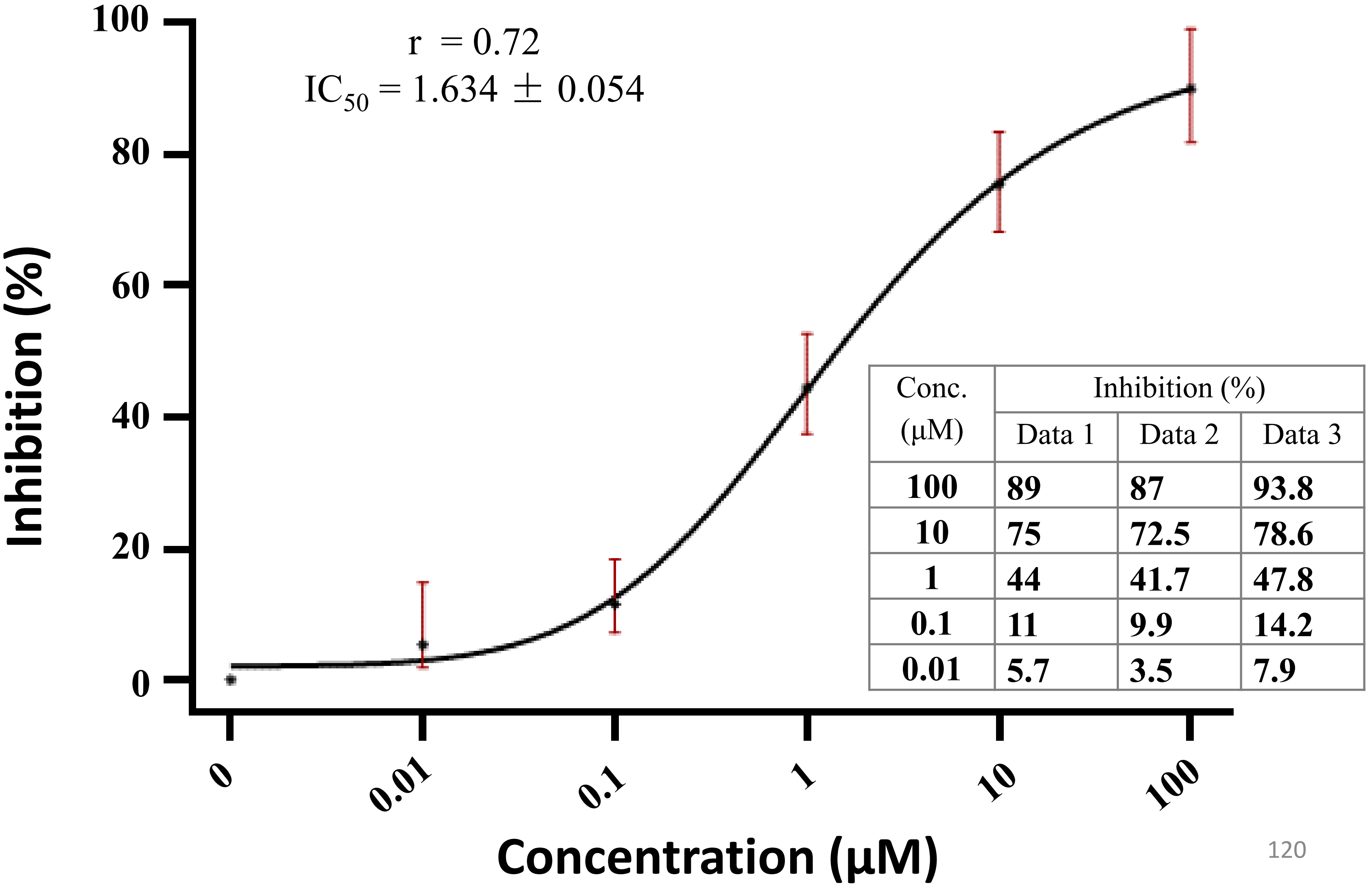

Conc. (μM)	Inhibition (%)		
	Data 1	Data 2	Data 3
**100**	**89**	**87**	**93.8**
**10**	**75**	**72.5**	**78.6**
**1**	**44**	**41.7**	**47.8**
**0.1**	**11**	**9.9**	**14.2**
**0.01**	**5.7**	**3.5**	**7.9**

Figure S23. Compound **4q** with Aromatase

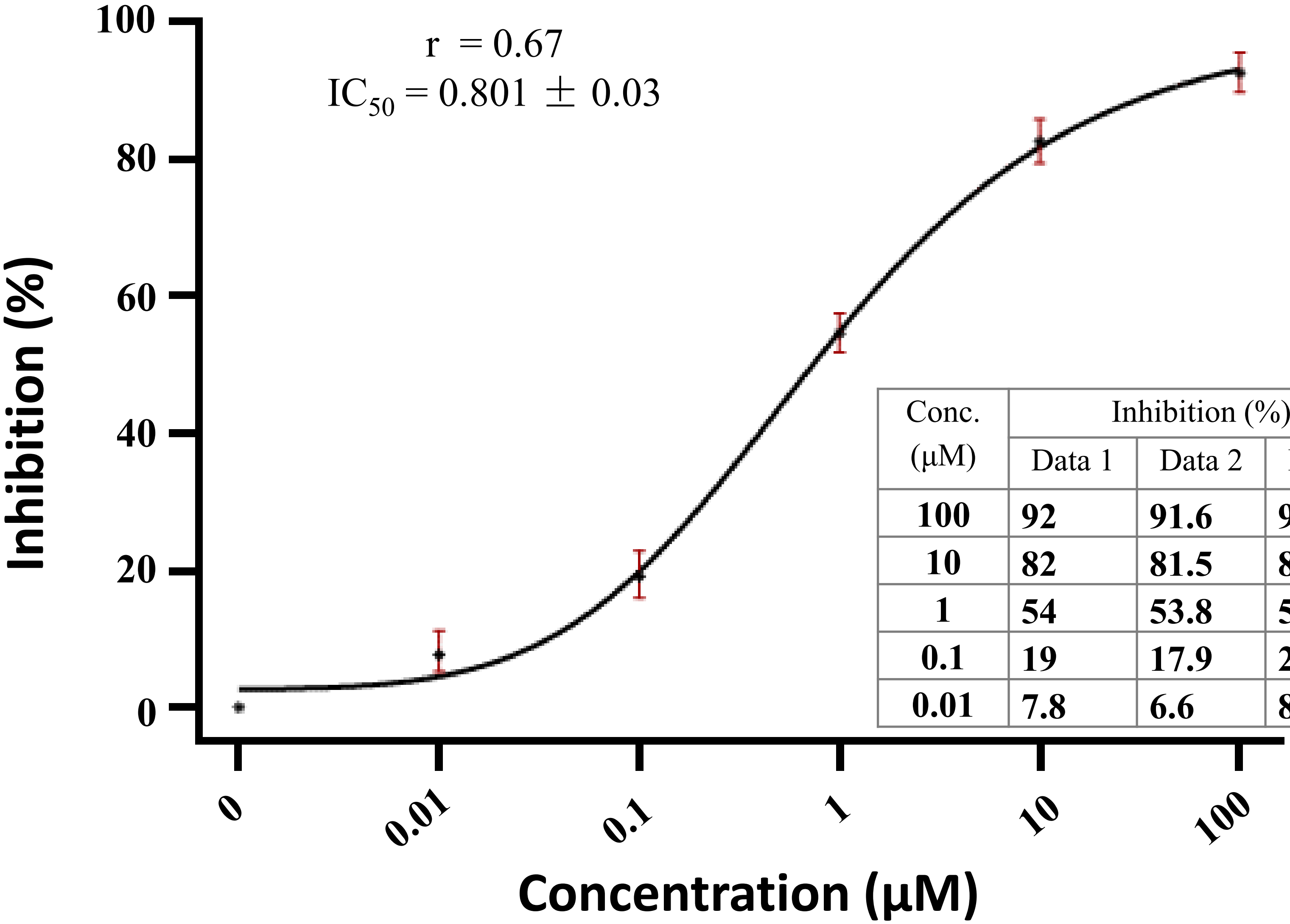

Conc. (µM)	Inhibition (%)		
	Data 1	Data 2	Data 3
**100**	**92**	**91.6**	**93.8**
**10**	**82**	**81.5**	**83.9**
**1**	**54**	**53.8**	**55.9**
**0.1**	**19**	**17.9**	**20.7**
**0.01**	**7.8**	**6.6**	**8.9**

Figure S24. Compound **4v** with Aromatase

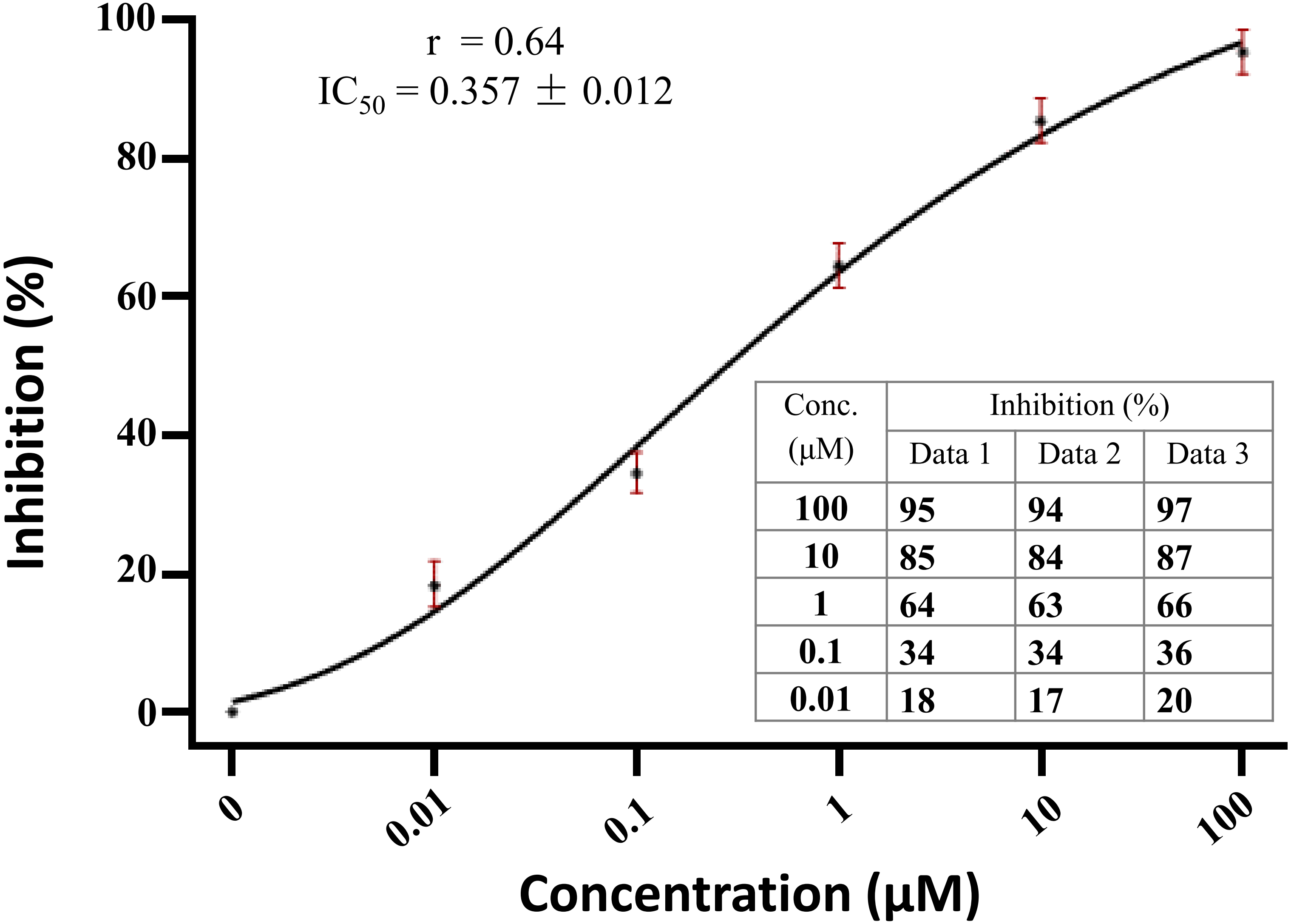

Conc. (µM)	Inhibition (%)		
	Data 1	Data 2	Data 3
**100**	**95**	**94**	**97**
**10**	**85**	**84**	**87**
**1**	**64**	**63**	**66**
**0.1**	**34**	**34**	**36**
**0.01**	**18**	**17**	**20**

Figure S21. Compound **letrozole** with Aromatase

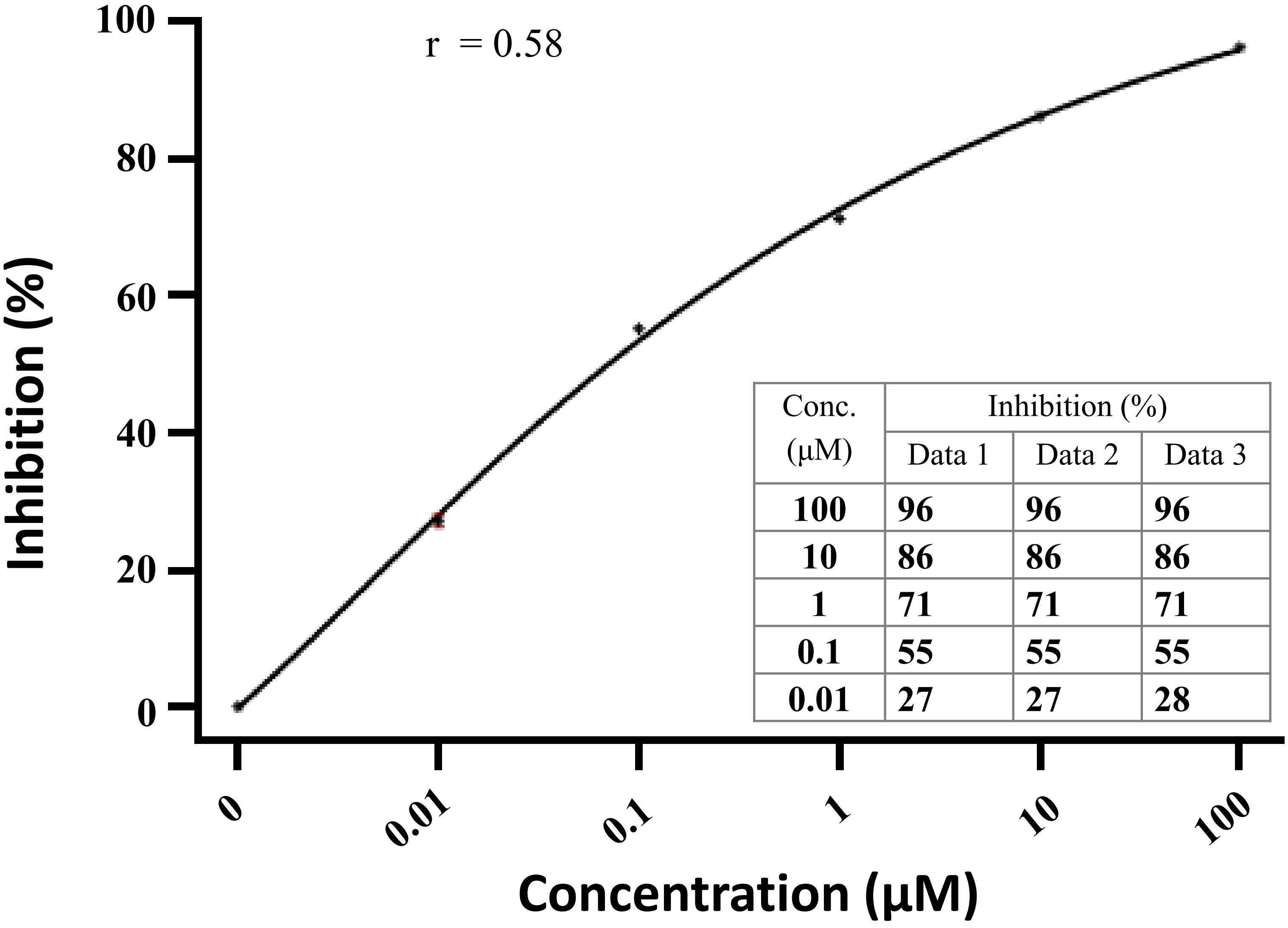

Conc. (µM)	Inhibition (%)		
	Data 1	Data 2	Data 3
**100**	**96**	**96**	**96**
**10**	**86**	**86**	**86**
**1**	**71**	**71**	**71**
**0.1**	**55**	**55**	**55**
**0.01**	**27**	**27**	**28**

Figure S22. Intermolecular interaction between (A) Erlotinib with EGFR (B) Erlotinib with HER2 (C) Dinaciclib with CDK2 (D) Sorafenib with VEGFR2

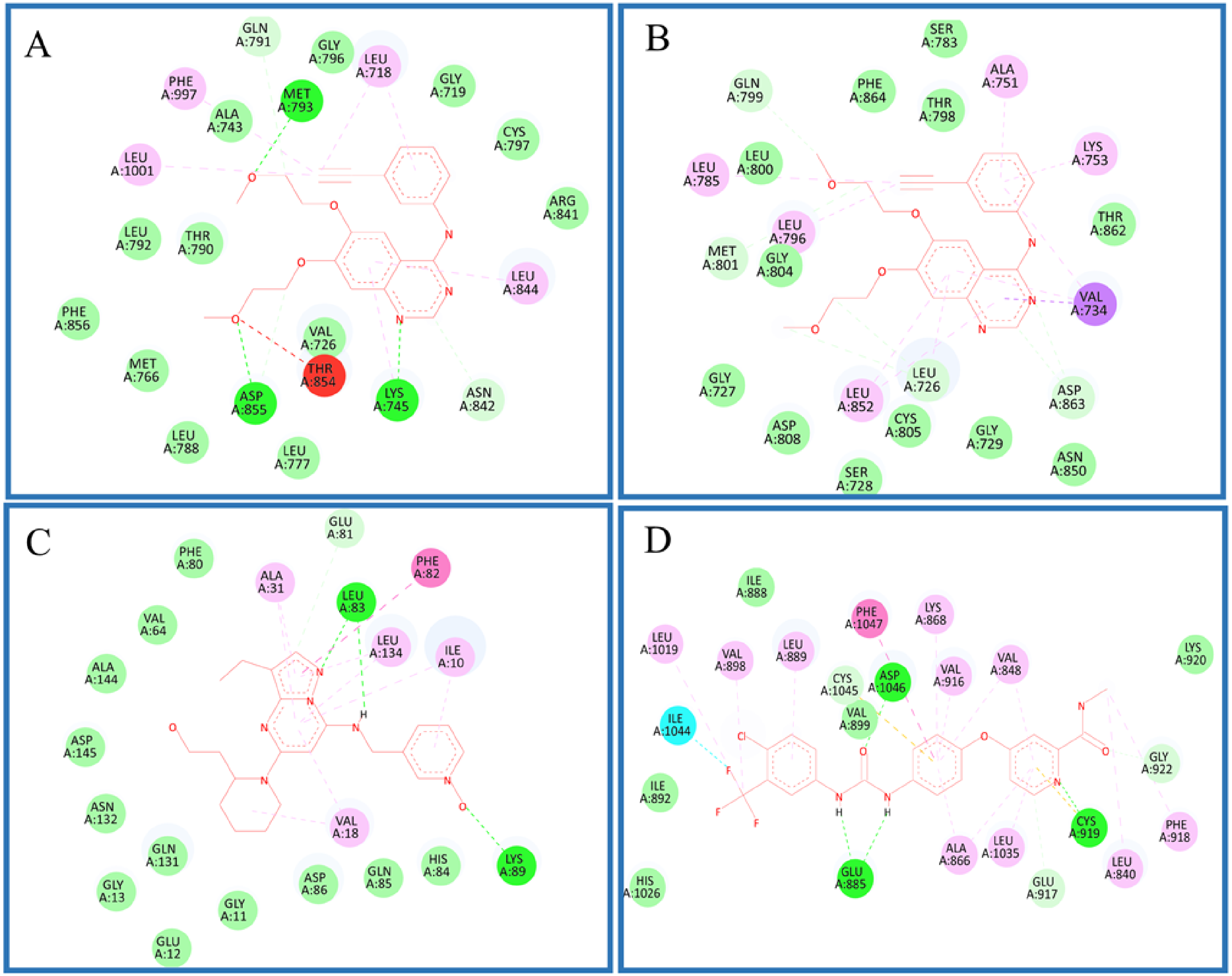

Figure S23. (A) compound **4q** (green) and erlotinib (blue) in the active site of EGFR. (B) compound **4q** (red) and sorafenib (blue) in the active site of VEGFR2

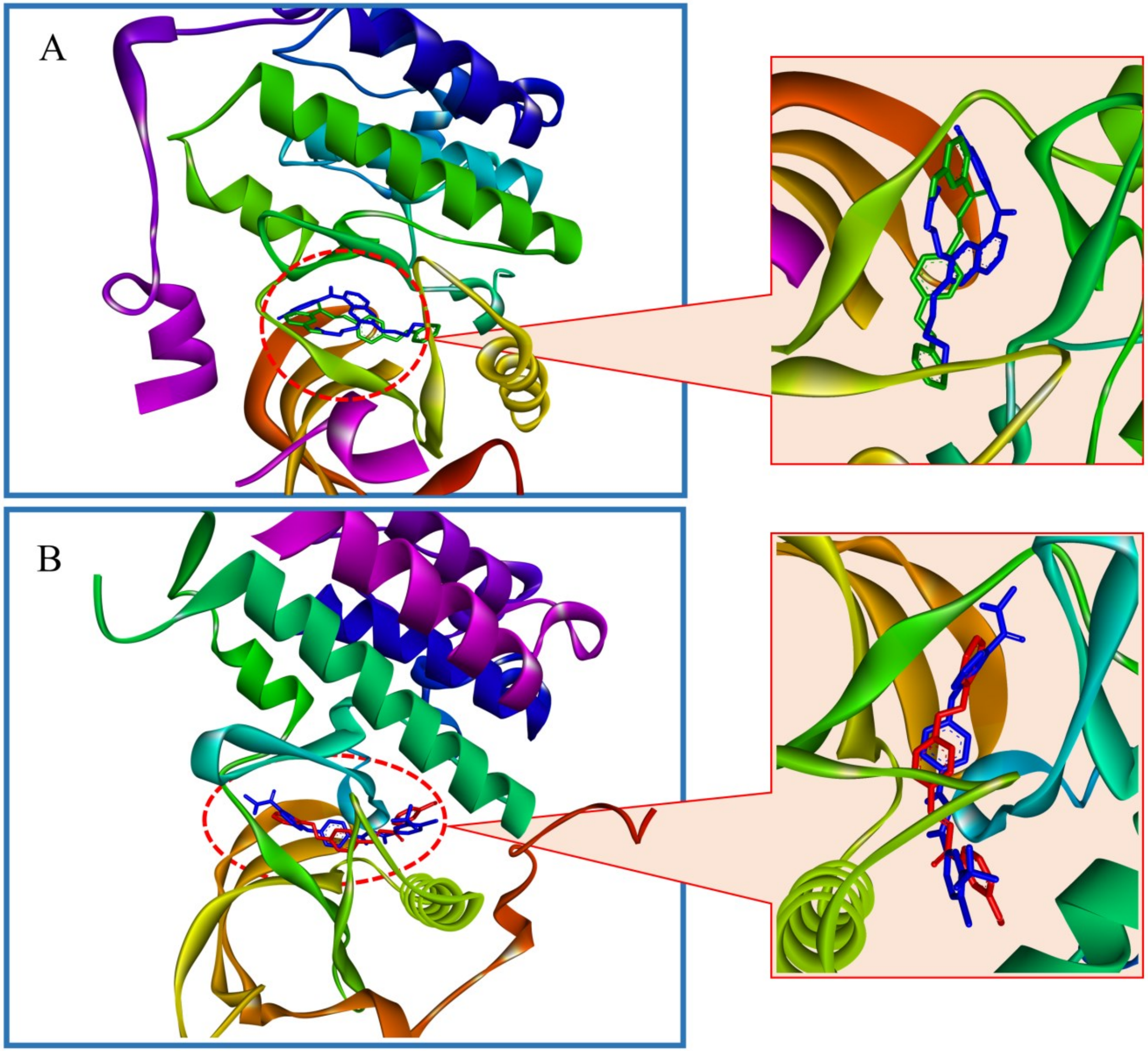

Figure S24. Alignment of (A) compound **4q** (yellow) and dibaciclib (blue) in the active site of CDK2; (B) compound **4q** (green) and erlotinib (blue) in the active site of EGFR

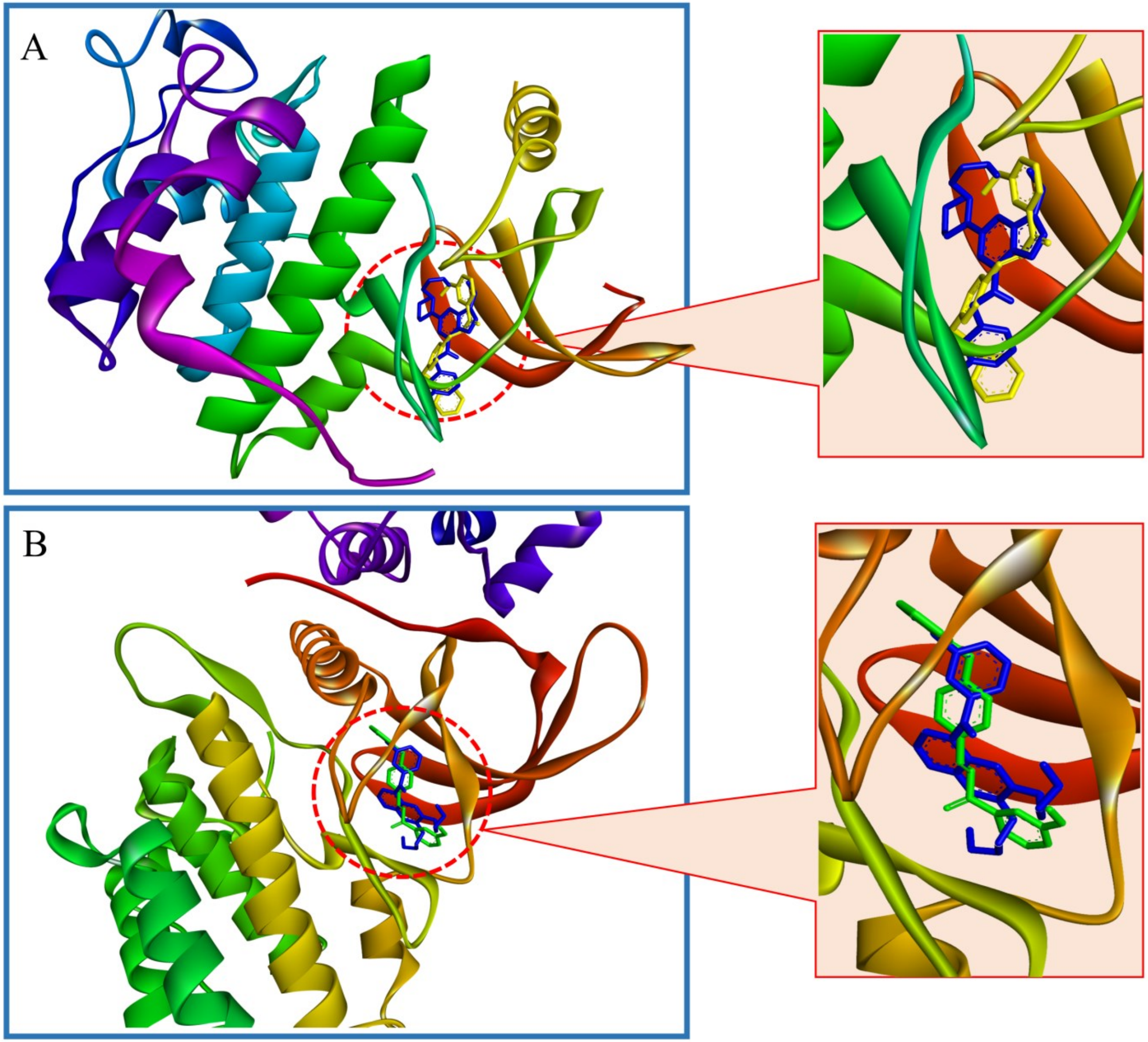

Figure S25. Alignment of (A) compound **4q** (red) and estradiol (blue) in the active site of Estrogen receptor (PDB id= 1a52) ; (B) compound **4q** (green) and tamoxifen (red) in the active site of Estrogen receptor (PDB id = 3ERT). Red dotted circle = Active site

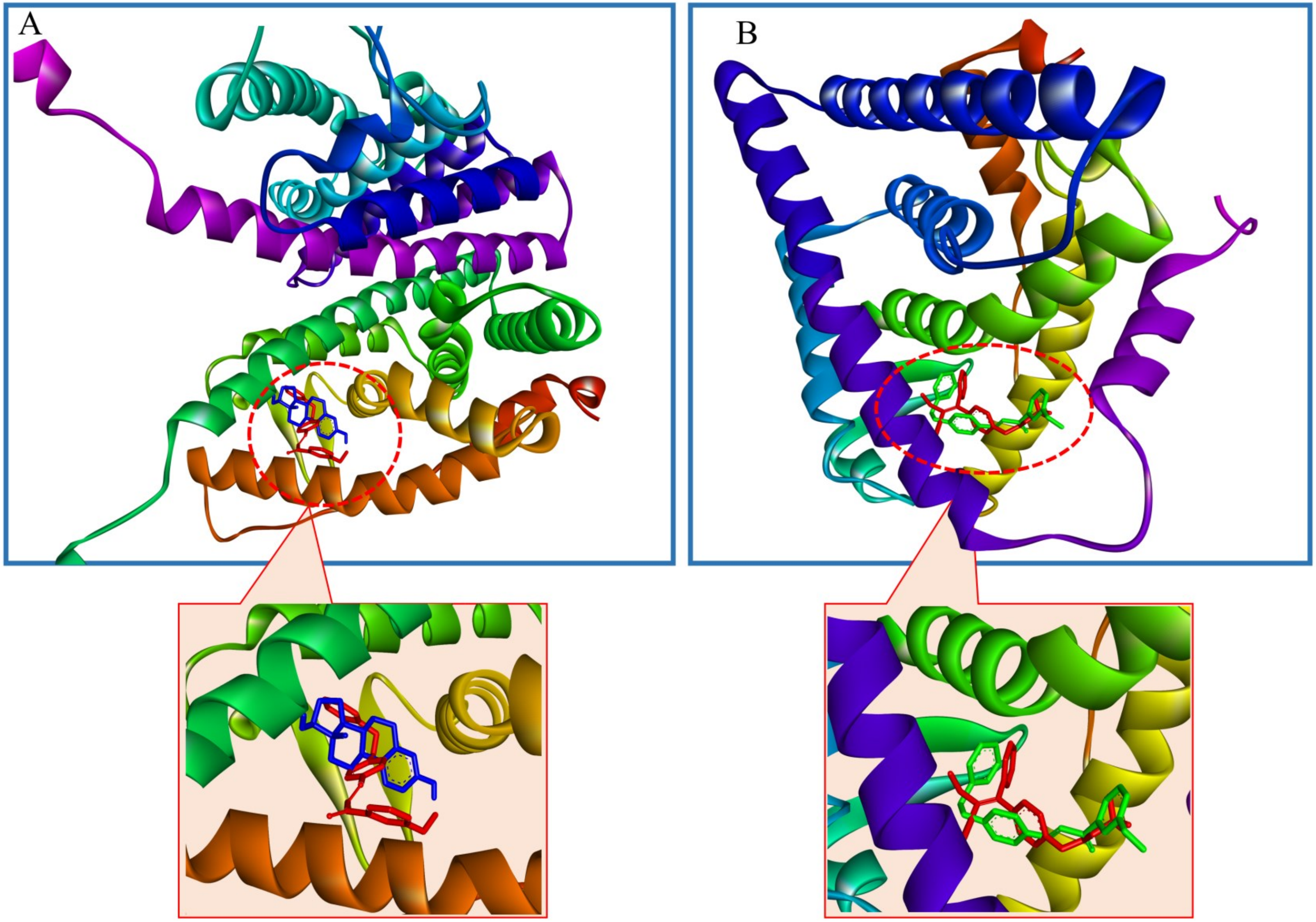

Supplement: Supplementary file 1 [file ijms-26-00833-s001.zip › ijms-3341298-supplementary.pdf]
